# The Genome of the Obligate Intracellular Parasite *Trachipleistophora hominis*: New Insights into Microsporidian Genome Dynamics and Reductive Evolution

**DOI:** 10.1371/journal.ppat.1002979

**Published:** 2012-10-25

**Authors:** Eva Heinz, Tom A. Williams, Sirintra Nakjang, Christophe J. Noël, Daniel C. Swan, Alina V. Goldberg, Simon R. Harris, Thomas Weinmaier, Stephanie Markert, Dörte Becher, Jörg Bernhardt, Tal Dagan, Christian Hacker, John M. Lucocq, Thomas Schweder, Thomas Rattei, Neil Hall, Robert P. Hirt, T. Martin Embley

**Affiliations:** 1 Institute for Cell and Molecular Biosciences, The Medical School, Newcastle University, Newcastle upon Tyne, United Kingdom; 2 Department of Computational Systems Biology, University of Vienna, Vienna, Austria; 3 Institute of Pharmacy, Ernst-Moritz-Arndt-University Greifswald, Greifswald, Germany; 4 Institute of Microbiology, Ernst-Moritz-Arndt-University Greifswald, Greifswald, Germany; 5 Institute for Molecular Evolution, Heinrich Heine University Düsseldorf, Düsseldorf, Germany; 6 School of Medicine, University of St Andrews, St Andrews, Scotland; 7 Department of Functional and Comparative Genomics, School of Biological Sciences, University of Liverpool, Liverpool, United Kingdom; University of California Los Angeles, United States of America

## Abstract

The dynamics of reductive genome evolution for eukaryotes living inside other eukaryotic cells are poorly understood compared to well-studied model systems involving obligate intracellular bacteria. Here we present 8.5 Mb of sequence from the genome of the microsporidian *Trachipleistophora hominis*, isolated from an HIV/AIDS patient, which is an outgroup to the smaller compacted-genome species that primarily inform ideas of evolutionary mode for these enormously successful obligate intracellular parasites. Our data provide detailed information on the gene content, genome architecture and intergenic regions of a larger microsporidian genome, while comparative analyses allowed us to infer genomic features and metabolism of the common ancestor of the species investigated. Gene length reduction and massive loss of metabolic capacity in the common ancestor was accompanied by the evolution of novel microsporidian-specific protein families, whose conservation among microsporidians, against a background of reductive evolution, suggests they may have important functions in their parasitic lifestyle. The ancestor had already lost many metabolic pathways but retained glycolysis and the pentose phosphate pathway to provide cytosolic ATP and reduced coenzymes, and it had a minimal mitochondrion (mitosome) making Fe-S clusters but not ATP. It possessed bacterial-like nucleotide transport proteins as a key innovation for stealing host-generated ATP, the machinery for RNAi, key elements of the early secretory pathway, canonical eukaryotic as well as microsporidian-specific regulatory elements, a diversity of repetitive and transposable elements, and relatively low average gene density. Microsporidian genome evolution thus appears to have proceeded in at least two major steps: an ancestral remodelling of the proteome upon transition to intracellular parasitism that involved reduction but also selective expansion, followed by a secondary compaction of genome architecture in some, but not all, lineages.

## Author Summary

Microsporidians are enormously successful obligate intracellular parasites of animals, including humans. Despite their economic and medical importance, there are major gaps in our understanding of how microsporidians have made the transition from a free-living organism to one that can only complete its life cycle by living inside another cell. We present the larger genome of *Trachipleistophora hominis* isolated from a human patient with HIV/AIDS. Our analyses provide insights into the gene content, genome architecture and intergenic regions of a known opportunistic pathogen, and will facilitate the development of *T. hominis* as a much-needed model species that can also be grown in co-culture. The genome of *T. hominis* has more genes than other microsporidians, it has diverse regulatory motifs, and it contains a variety of transposable elements coupled with the machinery for RNA interference, which may eventually allow experimental down-regulation of *T. hominis* genes. Comparison of the genome of *T. hominis* with other microsporidians allowed us to infer properties of their common ancestor. Our analyses predict an ancestral microsporidian that was already an intracellular parasite with a reduced core proteome but one with a relatively large genome populated with diverse repetitive elements and a complex transcriptional regulatory network.

## Introduction

Microsporidia are a diverse and highly successful group of obligate intracellular parasites affecting many eukaryotic phyla for which relatively few genomes – compared to the approximately 1200 microsporidian species described – are yet available [1]. Microsporidians are dispersed as resistant spores, which is the only life-cycle stage able to survive outside of a host cell. Infection of a new host occurs when the spore germinates and everts a unique polar tube apparatus through which the sporoplasm is injected into the host cell. The introduced sporoplasm undergoes intracellular replication and differentiation eventually producing new spores, which, after host cell lysis, are released to repeat the cycle of infection [2]. Originally described as the causative agents of pébrine, an economically important disease of silkworm, Microsporidia are also important pathogens of honeybees [3]. In recent years, microsporidians have emerged as important human pathogens: they cause chronic diarrhoea in children and the elderly, especially in the developing world, and they infect immunocompromised patients, including those with HIV/AIDS [4].

In addition to their medical and economic importance, Microsporidia have become models for understanding cellular and genomic reduction in eukaryotes [1]. Once thought to be early branching eukaryotes that diverged before the acquisition of the mitochondrion, more recent analyses have established them as a sister group to fungi which have secondarily lost several typical eukaryotic features and simplified others [1], [5]. The first microsporidian genome to be sequenced, *Encephalitozoon cuniculi* [6], revealed an extraordinary degree of genome reduction; it is only 2.9 MB, has high gene-density (1 gene/kb), hardly any repetitive DNA and possesses extremely short intergenic regions, resulting in the overlapping transcription of adjacent genes [7], [8]. Genomic reduction in the closely related *Encephalitozoon intestinalis* is even greater; its genome is only 2.3 Mb [9]. The extreme compaction of *Encephalitozoon* genomes is accompanied by a drastic reduction in coding capacity, with the loss of many genes and pathways required for a free-living lifestyle [6], [9]. However, genome size among the Microsporidia is distributed over at least a 10-fold range from 2.3 to 24 Mb [9], [10] suggesting that compacted genomes and massive gene loss might not be representative of the group as a whole. The small amount of sequence data available from larger microsporidian genomes [3], [10], [11] already suggests that there are lineage-specific variations in coding capacity and genome organisation.

To further investigate the tempo and mode of microsporidian genome evolution we have sequenced and analysed the larger genome of *Trachipleistophora hominis*, an opportunistic pathogen isolated from an HIV/AIDS patient suffering from a progressive severe myositis associated with fever and weight loss [12]. Unlike most microsporidians *T. hominis* can be reliably cultured in the laboratory [13] and hence is more amenable to experimental manipulation [14], [15]. As such, it has the potential to be developed into a much-needed model system for this important group of intracellular parasites, which cannot yet be genetically manipulated. *Trachipleistophora hominis* is also a phylogenetic outgroup to the previously sequenced microsporidian genomes [16], so it is particularly relevant for investigating broader features of microsporidian genome evolution and for inferring common ancestral states. Our comparative analyses reveal that, while the switch to intracellular parasitism was accompanied by a dramatic remodelling of the microsporidian proteome, the extreme genome reduction seen in *Encephalitozoon* spp. is a derived trait of that genus. The ancestral microsporidian was already an intracellular parasite with a greatly reduced core proteome, but it had a genome architecture similar to that of canonical eukaryotes.

## Results/Discussion

### Sequencing and assembly

Spores were isolated from *Trachipleistophora hominis* grown in co-culture with rabbit kidney (RK-13) cells [13] and were extensively purified to provide material for DNA extraction and library construction. A complementary dual approach of 454FLX sequencing for initial assembly at 32.5 fold coverage and high density SOLiD sequencing for improving sequence quality, was used to generate a *T. hominis* assembly comprising 310 scaffolds for a total of 8,498,182 bp. Based upon the total length of assembled 454 reads (378,359,925 bp) and the coverage estimate (32.5×) we estimated a genome size of approximately 11.6 Mb using the method of Carlton et al [17]. However, the accuracy of this estimate is uncertain because this method for calculating genome size is very sensitive to the level of repeat elements and the way in which the data is filtered and assembled [3], [17]. The true size of the *T. hominis* genome may therefore be smaller or larger than 11.6 Mb, and it is possible that an unknown portion of the genome, potentially including genes for proteins that we presently infer to be missing, is not represented in our assembly. Half of the *T. hominis* assembly (N50) was in large scaffolds of greater than 50,285 bp. This compares favourably with the short (N50 2902 bp) contigs of the ∼7.3 Mb *Nosema ceranae* draft assembly [3] and the partial data (∼13.3 Mb) from *Octospora bayeri* where average contig length is only 320 bp [10], and enabled us to perform a detailed analysis of the gene content, genome architecture and intergenic regions of a larger microsporidian genome.

### Genome size and gene density, introns and intergenic regions

**Figure 1:**
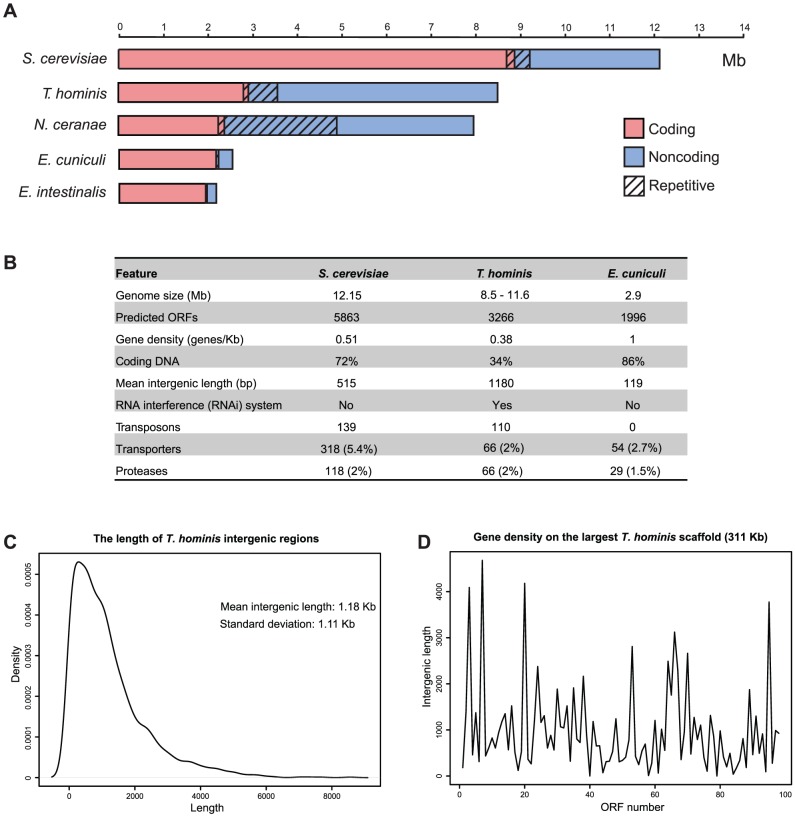
A comparative overview of some features of the *Trachipleistophora hominis* genome. (A) Proportions of coding (red) and non-coding (blue) genomic DNA sequence for *S. cerevisiae*, *T. hominis*, *N. ceranae*, *E. cuniculi* and *E. intestinalis*. The repetitive content, which includes some coding as well as non-coding sequences, is indicated by the hatched overlay. The graph for *E. cuniculi* is based upon the 2.5 Mb of sequence that is available from Genbank and hence is less than the predicted genome size of 2.9 Mb. (B) Comparison of some general features of the genomes of *S. cerevisiae*, *T. hominis* and *E. cuniculi*. Numbers of transposons, transporters and proteases are those that could be identified based on similarity to characterized sequences in model organisms. (C) Density plot showing length variation of intergenic regions in the large *T. hominis* scaffolds. (D) Local length variation in intergenic regions over the largest (#00035, 311,951 bp) *T. hominis* scaffold.

Although our *T. hominis* assembly encodes an estimated 3,266 open reading frames, which is more than previously-sequenced microsporidians (Figure 1, Table S1), the most significant contributor to the difference in genome size between *T. hominis* and the three-times-smaller *E. cuniculi* genome is the difference in gene density, with a mean intergenic DNA length of 1.18 Kb for *T. hominis* and 119 bp for *E. cuniculi* [6]. This demonstrates that proteome reduction and genome compaction are not coupled in Microsporidia: with only 0.38 genes/Kb, *T. hominis* is actually less gene-dense than the free-living model organism *Saccharomyces cerevisiae* (0.51 genes/Kb; Figure 1). Indeed, with only 2.8 Mb of coding DNA in our 8.5 Mb genome assembly, *T. hominis* is a gene-sparse outlier among unicellular eukaryotes in general [18]. Previous small-scale gene surveys from *Brachiola algerae* and *Edhazardia aedis* [19] and the short genomic contigs from *N. ceranae* [3] and *Octospora bayeri* [10] had already suggested that gene density was lower in species with larger genomes, but the availability of much longer contiguous fragments of *T. hominis* sequence enabled us to investigate the size and content of intergenic regions in greater detail. Although the mean intergenic distance for *T. hominis* is 1.18 Kb, there is a large spread around the mean (Figure 1C); some intergenic lengths are similar to those in *E. cuniculi*, but others are much longer. Coding density averaged across each of the 14 largest scaffolds (those greater than 100 Kb in size) in our assembly is remarkably consistent (0.34 genes/Kb; standard deviation 0.05); the wide range of intergenic lengths is the result of local, within-scaffold variation (Figure 1D). Thus, while gene density appears to fluctuate locally over the *T. hominis* genome, we found no evidence for larger-scale regional differences.

The published genomes of microsporidians have relatively few spliceosomal introns and these are often, but not exclusively, in ribosomal protein genes (RPG) [6], [9], [20]. The *T. hominis* genome contains components of the spliceosomal machinery (Figure S1), so we searched for introns in *T. hominis* genes using a conserved microsporidian motif based upon introns in *E. cuniculi* [20] and *N. ceranae* [3]. We identified 78 *T. hominis* ORFs with putative introns (Table S2), but some of these are much longer than validated *E. cuniculi* introns [20] and all will require experimental verification. The true number of *T. hominis* introns may therefore be less than 78; only 36 introns have so far been detected for *E. cuniculi* [6], [20] and only 6 for *N. ceranae* [3]. We detected 3 RPG containing putative introns (Table S2, RPG S23, S26 and L24), one of which, RPG S26 has an intron in *E. cuniculi* [20] but not in *N. ceranae* (Table S3). To further investigate the occurrence of introns in *T. hominis* RPG, we aligned *T. hominis* genes with the intron-containing RPG of *E. cuniculi* and *N. ceranae* and identified two additional *T. hominis* RPG (S17, L39) containing a related motif (Figure S2) at the same position as the introns in *E. cuniculi* and *N. ceranae* RPG. This new motif was used to search the *T. hominis* genome and identified two additional ribosomal proteins (S27 and L44) that were not predicted correctly by the initial ORF annotation. The orthologues of these RPGs in *N. ceranae* and *E. cuniculi* do not contain introns. Thus, while RPG are enriched for introns in *E. cuniculi*, *N. ceranae* and *T. hominis*, the identities of intron-containing RPG are only partially conserved between the three species (Table S3). Introns are also overrepresented in the RPG of *Saccharomyces cerevisiae* and were preferentially retained following whole-genome duplication, indicating ongoing selection for retention of RPG introns [21]. In the case of *Saccharomyces*, it has been suggested that introns persist in ribosomal protein genes because they influence ribosomal protein gene expression as part of an autoregulatory circuit [21]. It is possible that a similar mechanism may underlie the persistence, against a background of reductive evolution, of intron-containing RPG in microsporidians.

**Figure 2:**
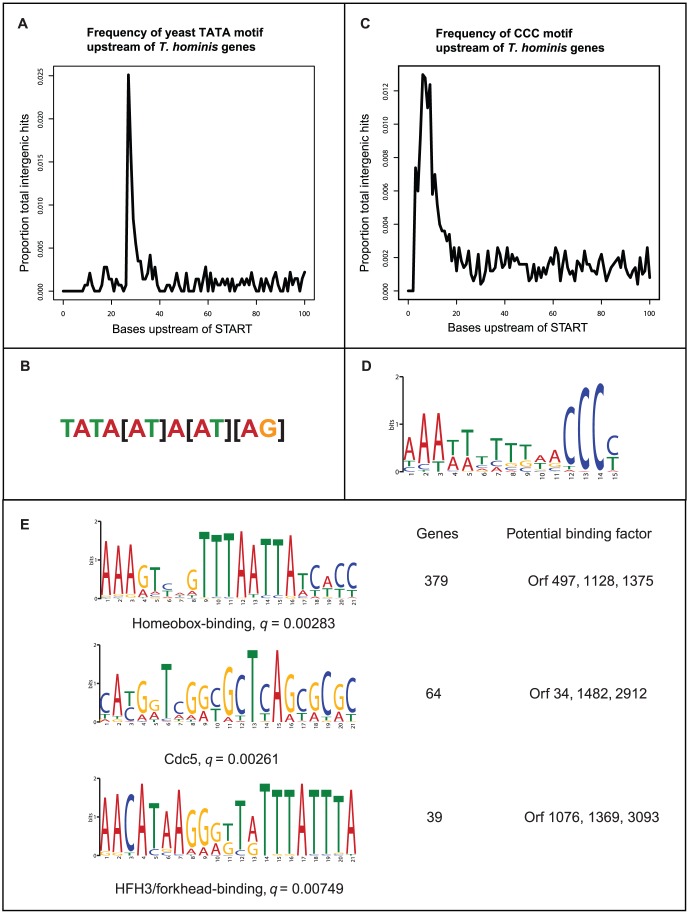
Putative regulatory motifs in the intergenic regions of *T. hominis*. The intergenic regions upstream of *T. hominis* protein coding genes are enriched for both the canonical yeast-type TATA box (A, B) and a microsporidia-specific “CCC” motif (C, D). (E) The longer scaffolds of the *T. hominis* assembly also enabled us to robustly identify additional enriched motifs upstream from coding sequences (Figure S3, Table S4, S5). These included sequences with significant similarity to binding sites for Lim1-like homeobox-binding and fork head-containing transcription factors, as well as the cell cycle regulator Cdc5. The q-value for a match between a motif identified in *T. hominis* and the database consensus motif is a p-value that has been corrected for multiple testing by the False Discovery Rate method.

The intergenic regions of eukaryotes typically contain regulatory motifs that control the expression of adjacent genes. The short intergenic spacers of *E. cuniculi* lack canonical eukaryotic regulatory motifs [3], [7], although they are enriched for a microsporidia-specific “CCC” motif [22]. A yeast-like TATA box was identified upstream of 194 *T. hominis* genes, and the microsporidia-specific “CCC” motif upstream of 977 genes (Figure 2A–D, Table S4). The positions of these regulatory motifs relative to the start codon are strongly conserved between *T. hominis* and *N. ceranae* [3] (Figure 2A–B), suggesting that their common ancestor possessed these core motifs. The shift in *E. cuniculi* [22] of regulatory motifs into upstream genes thus appears to be the result of secondary reduction and is not representative of the Microsporidia as a whole. We also identified 40 additional candidate regulatory motifs upstream from the start codons of 774 *T. hominis* genes (Figure S3, Tables S4 and S5). Six of these motifs are similar to known transcription factor binding motifs (Table S5) and homologues of the corresponding transcription factors for three of them were identified in the *T. hominis* genome (Figure 2E). The remaining 34 motifs (Figure S3, Table S5) appear to be *T. hominis*-specific, providing evidence of a complex transcriptional regulatory network in this species.

### Transposable elements (TE) and RNA interference (RNAi)

**Figure 3:**
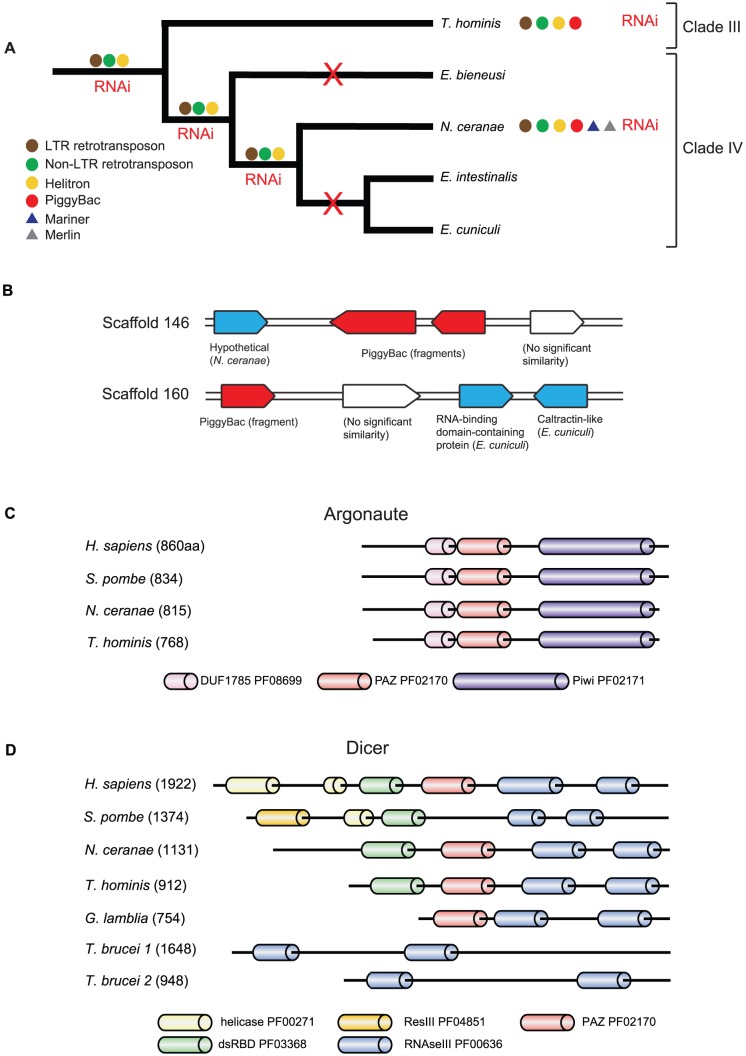
Transposable elements and the RNAi machinery in *T. hominis* and other microsporidians. (A) Comparison of the TE content of *T. hominis* and *N. ceranae* suggests that the genome of the microsporidian ancestor contained several distinct families of transposons, which then appear to have been independently lost in some lineages (Table S6, Figures S4 to S7). Merlin transposons were only detected in the highly repetitive *N. ceranae* genome. (B) The genome of *T. hominis* contains PiggyBac DNA transposons which appear to have originated by LGT from a close relative of the ant *Harpegnathos saltator* (Figure S7), and thus independently from those detected in *N. ceranae*. The integration of the PiggyBac elements into the *T. hominis* genome was confirmed using PCR. (C, D) Domain conservation in the *T. hominis* and *N. ceranae* RNAi proteins Argonaute and Dicer. The domain architecture of Argonaute (C) and Dicer (D) were inferred using a pHMMER search of the respective sequences with default parameters. To identify more divergent domains in the Dicer homologues from parasites, the sequences of *N. ceranae*, *T. hominis*, *T. brucei* (1 and 2), and *G. lamblia* were investigated by searching the Pfam dataset from the conserved domain database at NCBI with an expected threshold of 100 [131]. This identified the second N-terminal RNAse III domains of *T. brucei* Dicer 1 and Dicer 2, as well as an additional PAZ (Piwi, Argonaut and Zwille) domain for *T. hominis*. The Argonaute domain architecture is highly conserved among species, but Dicer is more variable and both microsporidian sequences as well as those of *G. lamblia* and *T. brucei* lack domains typically present in other organisms.

A notable observation when the genome of *E. cuniculi* became available was the lack of transposable elements (TEs), which can make up a large fraction of eukaryotic genomes [23]. Subsequent analyses of the genome of *Encephalitozoon intestinalis* [9] and partial genome data for *Enterocytozoon bieneusi* [11] also failed to identify any TEs. By contrast, TEs were detected in the genome of *Nosema ceranae* [3] and they have also been reported for the silkworm parasite *Nosema bombycis* [24], the opportunistic human pathogen *Vittaforma corneae* [25] and the fish parasite *Spraguea lophii* [26]. We identified 110 ORFs that are predicted to be encoded by TEs in the genome of *T. hominis* (Table S6). Phylogenetic analysis of the proteins associated with helitron (Figure S4) and non-LTR elements (Figure S5) in *T. hominis* and *Nosema ceranae*, and with LTR-elements in *T. hominis*, *N. ceranae* and *Nosema bombycis* (Figure S6), suggests that all three types of TE were present in the common ancestor of these species (Figure 3A). The phylogeny of non-LTR elements in *T. hominis* (Figure S5) also suggests that they have undergone recent expansion in this lineage, although the presence of frameshifts in their coding sequences suggests that they are no longer active. *N. ceranae* encodes mariner elements, and we found some evidence of relict mariner elements on the *T. hominis* genome, but without any protein-coding sequence from *T. hominis* we were unable to test the hypothesis that these elements were inherited from the common microsporidian ancestor, as opposed to being acquired independently. Both *N. ceranae* and *T. hominis* also contain some highly derived sequences that are similar to hAT transposons.

The distribution of TEs was mapped onto a schematic tree (Figure 3A) based upon ribosomal DNA phylogeny for microsporidians [16]. *Trachipleistophora hominis* is a member of clade III in the reference phylogeny and is an outgroup to clade IV which contains *Encephalitozoon, Enterocytozoon and Nosema* [16]. The distribution of TEs on the reference phylogeny (Figure 3A), coupled with the apparent monophyly of coding sequences (Figures S4, S5, S6), suggests that *Enterocytozoon bieneusi* and *Encephalitozoon* spp. have independently purged their genomes of TEs. PiggyBac elements were also detected in *T. hominis* and *N. ceranae* [3] but they do not cluster together and therefore appear to be independent acquisitions, rather than the products of vertical inheritance from a common microsporidian ancestor. Indeed, based upon its phylogeny (Figure S7), the *T. hominis* piggyBac element appears to have originated by recent lateral gene transfer (LGT) from an ant or a close relative (Figure 3B). Consistent with this observation, it has previously been speculated that the natural host for *T. hominis* is an insect; *T. hominis* can infect mosquitos in the lab [27] and is closely related to species of the genus *Vavraia*, which are insect parasites [28], [29].

The occurrence of TEs in *T. hominis* and *N. ceranae* is coupled with the presence of key proteins of the RNAi machinery (Figures 3C and 3D), consistent with a role for microsporidian RNAi in transposon defence [30], [31]. The domain architecture of microsporidian Argonaute proteins (Figure 3C) is conserved compared to yeast and humans, whereas Dicer proteins appear to be reduced (Figure 3D). However, loss of conserved domains has already been reported for the functional Dicer proteins of *Giardia lamblia* [32] and *Trypanosoma brucei* [33]. Moreover, *N. ceranae* genes were shown to be down-regulated after adding the respective RNAi to the sucrose solution fed to *N. ceranae*-infected bees [34]. The discovery of genes for the RNAi machinery in *T. hominis* – Argonaute was also identified in the spore proteomics data (Table S19) – raises the exciting prospect of using RNAi to down regulate *T. hominis* genes and eventually of developing it into a model system which, unlike *N. ceranae* and most other species, can be easily grown in co-culture with mammalian cells.

### Microsporidian proteome evolution

As an outgroup to the microsporidians for which most sequence data is available, the predicted proteome of *T. hominis* provides an opportunity to infer the gene content of the common ancestor of these species. The annotated predicted proteomes of *T. hominis*, *E. cuniculi*, *E. intestinalis*, *Enterocytozoon bieneusi*, *N. ceranae* and two opisthokont [35] outgroups (*S. cerevisiae* and *Homo sapiens*) were investigated for the presence of protein (Pfam [36]) domains, and patterns of presence and absence were mapped onto a schematic phylogeny (Figure S8) using Dollo parsimony [37]. Dollo parsimony makes the assumption that when a complex character is lost during the evolution of a particular lineage it cannot be regained. It has proved useful for reconstructing the evolution of the gene repertoire of eukaryotic organisms because although multiple, independent losses of a gene in different lineages are common, multiple gains of the same gene in different lineages are not (not withstanding LGT – see later) [37].

The major loss (1123 cases) of Pfam domains mapped to the ancestral microsporidian branch (Figure S8), and corresponds to a dramatic reduction in metabolic pathways and typical eukaryotic features [2], [38] upon the transition of Microsporidia to intracellular parasitism. Additional major losses of Pfam domains, but relatively few gains, were mapped to each internal branch, suggesting further lineage-specific reduction in domain diversity (Figure S8). One surprising result of these analyses was the observation that, uniquely among the microsporidians analysed (Figure S8), *Enterocytozoon bieneusi* appeared to have gained a large (69) number of Pfam domains.

We analysed the 74 *E. bieneusi* proteins containing the 69 Pfam domains using BLASTP, and found that they share highest similarity to diverse bacterial sequences (Table S7). We also investigated the codon usage for each species investigated, measuring the frequency of optimal (F_OP_) codons [39] for each genome. The F_OP_ plots (Figure S9) for *T. hominis* and the other microsporidians (except *E. bieneusi*) and for *Saccharomyces cerevisae* had a single peak, suggesting that optimal codon usage is relatively homogeneous for each genome. By contrast, the F_OP_ plot for *E. bieneusi* was broader and had two major peaks. We measured the F_OP_ scores for the 74 genes in the 69 Pfams and found that they had significantly lower F_OP_ scores (P<2.2e-16) than the remainder of the *E. bieneusi* genes analyzed, suggesting that at least some of the observed heterogeneity in codon usage was due to these genes. There are at least two possible explanations for these results. The 74 genes found in the *E. bienuesi* genome sequence data might be the result of multiple LGTs from diverse bacteria, but the few published examples of LGTs affecting microsporidians [40], [41] and the small number of LGTs we detected for *T. hominis* in our genome-wide screen (see later), suggest that LGT affecting microsporidians is relatively rare. An alternative and perhaps simpler explanation, is that the *E. bieneusi* assembly is contaminated with bacterial sequences. This appears possible because the purified *E. bieneusi* spores used for DNA extraction were isolated directly from fecal samples from an infected patient [11], as this microsporidian cannot yet be cultured in the laboratory.

**Figure 4:**
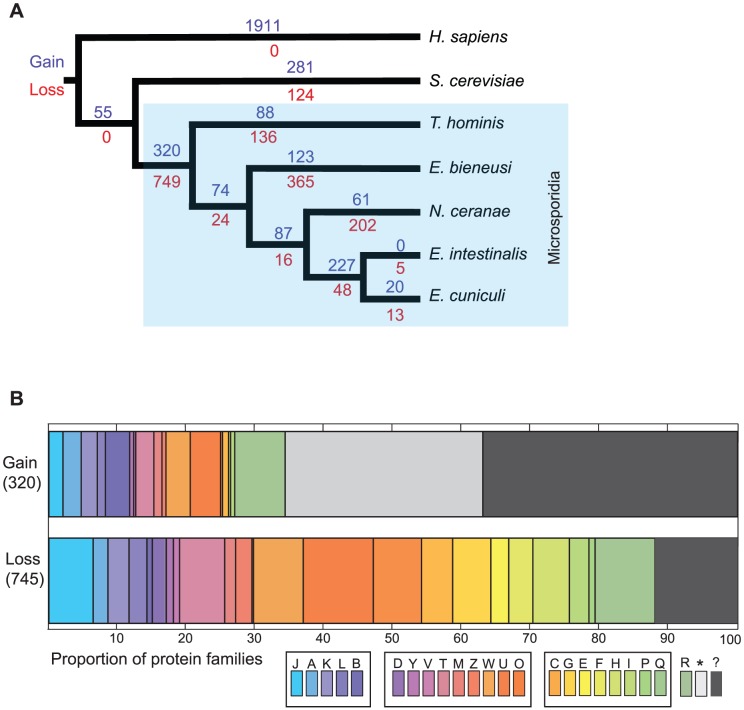
Gain and loss of protein families during microsporidian genome evolution. Homologous protein families were identified using MCL clustering of similar sequences, as determined by PHMMER searches (E<0.01). To prevent the clustering of otherwise unrelated proteins that share a common functional domain, we required that significantly similar sequence pairs align over at least 50% of their length. (A) Gain and loss events inferred using Dollo parsimony were mapped onto a cladogram derived from an 18S rRNA phylogeny [16]. The numbers on the cladogram show the number of protein families lost (red) or gained (blue). (B) Functional classification of protein families gained or lost in the microsporidian ancestor using the COG functional categories, with the three broad divisions of informational genes, cellular processes and metabolism delineated. The microsporidian losses are distributed across COG categories, with a particular impact on cellular processes and metabolism, whereas most of the gains are of unknown function. Key: J, translation, ribosomal structure and biogenesis; A, RNA processing and modification; K, transcription; L, replication, recombination and repair; B, chromatin structure and dynamics; D, cell cycle control, cell division, chromosomal partitioning; Y, nuclear structure; V, defence mechanisms; T, signal transduction; M, cell wall/membrane/envelope biosynthesis; N, cell motility; Z, cytoskeleton; W, extracellular structures; U, intracellular trafficking, secretion, vesicular transport; O, posttranslational modification, protein turnover, chaperones; C, energy production and conversion; transport and metabolism of: G, carbohydrate; E, amino acids; F, nucleotides; H, coenzymes; I, lipids; P, inorganic ions; Q, secondary metabolites. R – general function prediction only; *, no whole-protein functional assignment, but contains a known functional region identified by HHsearch [44] (Table S8); ?, no functional region identified using HHsearch.

Because the construction of Pfam domains is biased towards proteins from model organisms, we also identified protein families *de novo* using an MCL clustering algorithm [42] and sensitive profile hidden Markov models (HMMER ver. 3.0) [43] for sequence similarity searches. This also allowed us to investigate the distribution and conservation of the many hypothetical or uncharacterised gene families found in microsporidian genomes. In these analyses the potential contamination of the *E. bieneusi* data set was less of a problem as it mainly comprised single sequences, and our cluster analysis was focused on protein families i.e. those containing two or more sequences in one or more lineages. This approach also excluded short and potentially unreliable singleton hypothetical ORFs that are common artefacts in genome sequencing projects. Gain and loss of clusters was mapped onto the schematic phylogeny as before (Figure 4A), and clusters were also classified according to their COG functional annotation (Figure 4B). The greatest loss (749) of protein families again occurred at the base of the Microsporidia and particularly affected genes in the broad categories of metabolism and cellular processes (Figure 4B). However, the *de novo* clustering analysis also demonstrates that microsporidian genome evolution is much more dynamic than was revealed by the Pfam-based analysis (Figure S8), particularly in terms of the lineage specific gain of potentially novel protein families. For example, we inferred the gain of 88 protein families on the branch leading to *T. hominis* compared to only 5 Pfam domains gained (Figure S8). We also inferred the gain of 320 protein families in *T. hominis* and at least one other microsporidian species. These families, which are mainly of unknown function, were potentially present in the common ancestor of the microsporidians analysed (Figure 4A).

The 320 families detected may represent protein families that: 1) are microsporidian inventions; 2) were present in the common opisthokont ancestor [35] of the species investigated, but were secondarily lost from the outgroups used; 3) have diverged so far from their homologues in *S. cerevisiae* or *H. sapiens* as to be undetected in our searches; 4) were acquired by LGT at the base of the microsporidian radiation. To distinguish between these possibilities, and to extend the scope of our searches, we used BLASTP and the more sensitive HHsearch [44] to detect and characterize putative homologues of the 320 families in the public sequence databases (Figure S8). We identified potential homologues in prokaryotes or eukaryotes, including some in *Saccharomyces cerevisae* and *Homo sapiens*, for 227 families (Table S8). These included the nucleotide transporter proteins (NTT) that allow Microsporidia to steal ATP from their host cells [45]; a key innovation facilitating their parasitic lifestyle that is shared with a number of intracellular bacterial pathogens including species of *Chlamydia* and *Rickettsia* [46].

The remaining 93 protein families (Table S9, 21 of which are also represented in the spore proteomics data in Table S19) may represent microsporidian-specific innovations or proteins that have diverged beyond detectable similarity. Ten of the families contain ORFs with 2 or more predicted transmembrane domains (TMD); these are likely integral membrane proteins and some may be transporters (Table S9). Of the 93 families, 35 are conserved in all of the microsporidians investigated and 65 are conserved in all species investigated except *E. bieneusi*, although there are only partial genome sequence data available for this species [11]. Thus, along with massive gene loss and reductive evolution, the remodelling of the microsporidian proteome appears to have involved substantial gain of potentially novel parasite-specific functions.

**Figure 5:**
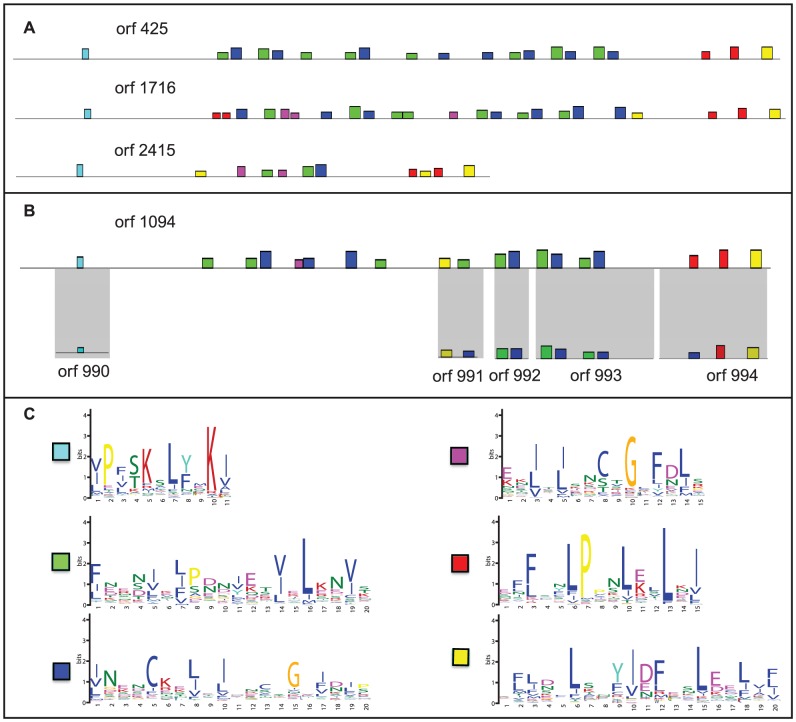
Characterization of a *T. hominis*-specific family of leucine-rich repeat (LRR) containing proteins. (A) Representative family members showing the characteristic pattern of LRR motifs. (B) Some shorter members of this family appear to be fragments derived from larger ORFs by pseudogenization, as indicated by patterns of sequence similarity and synteny. The height of the coloured boxes indicates the p-value of the motif hit assigned by the MEME suite [103]. (C) The LRR motifs that form the building blocks of this protein family, which, at 117 members, is the largest identified in the *T. hominis* genome.

Our analyses also identified 88 gene families, containing 371 ORFs, that were present in *T. hominis*, but not in the other microsporidians or the two outgroups (Figure 4A). A broader-based search identified distant homologues for a few of these ORFs in the databases, but many more are from TEs and most are hypothetical proteins of unknown function (Table S10). Most microsporidian-specific families are very small: median family size is two members among families that are present in single microsporidian genomes (data not shown). However, a small number of extreme expansions have occurred in individual genomes; the largest of these is a unique gene family found only in *T. hominis* (Table S10). This family has 117 ORFs (Table S10) containing motifs that are highly enriched in leucines (or amino acids known to replace leucine [47]), suggesting that this is an expanded *T. hominis*-specific family with a divergent set of leucine-rich repeats (LRRs) [47] (Figure 5). We found both full-length and apparently fragmented family members (Figure 5), consistent with an ongoing process of pseudogenization. Enrichment of LRR and other repetitive proteins is a feature of the genomes of diverse bacterial, fungal and protozoan pathogens, and these proteins can have important roles in host-pathogen interactions [48], [49]. Further study of these proteins may thus provide insights into the poorly understood interactions between *T. hominis* and host cells.

### Gene content and metabolism of *Trachipleistophora hominis*

Although with 3266 predicted ORFs (including 110 TEs), the *T. hominis* assembly has more putative protein coding sequences than the 1997 ORFs identified for *E. cuniculi*, most (72%) of the additional *T. hominis* ORFs are hypothetical, implying that the major changes to the microsporidian core proteome occurred in the ancestor of the group. Hence, in contrast to the diversity of genome sizes and architectures among the microsporidians analysed (Figure 1), there appears to be much less variation in metabolic capacity (Table S11 and S12). The genes detected for *T. hominis* were mapped onto the KEGG pathways to provide an overview of *T. hominis* metabolism and pathways (Figure S1).

The reduction in protein length first reported for *E. cuniculi* [6] relative to orthologous proteins in *S. cerevisiae* is shared with *T. hominis* (P<10^−15^, paired Mann-Whitney test). It has been suggested [6] that this reduction in protein length reflects the loss of protein interaction surfaces that are no longer needed in a smaller proteome; consistent with this prediction, our analyses indicate that the loss of some proteins and the reduction in length of others were coupled in the ancestral microsporidian. Together with the reduction in client protein diversity, we also detected less chaperone diversity in *T. hominis* and other microsporidian genomes compared to human and yeast (Table S13). Thus microsporidians have fewer Hsp70 and Hsp40 homologues, and all of them appear to have lost the important mitochondrial chaperone Cpn60/Hsp60 and its co-chaperone Cpn10/Hsp10. By contrast, all of the microsporidians investigated have retained all eight paralogous subunits of the cytosolic TCP-1 ring complex (TRiC/CCT) that are found in yeast (Figure S10, Table S13). The TRiC/CCT complex assists the folding of a broad range of eukaryotic proteins including actin and tubulin, but it also interacts with the septin ring complex that is important for cytokinesis, the nuclear pore complex, and protein degradation pathways [50]. In yeast, the protein prefoldin functions as a co-chaperone of TriC/CCT in mediating chaperone-substrate interactions [51], but we did not detect homologues of prefoldin among microsporidians. This suggests that microsporidian TriC/CCT functions independently of a co-chaperone, or that an alternative chaperone has evolved to assist TriC/CCT function. Like microsporidia, intracellular bacterial pathogens also tend to lose proteins and biochemical pathways when they can rely on their hosts to supply them with the substrates that they need [52]. The bacterial proteins that are retained are highly derived and are susceptible to misfolding, so there is typically a large investment in increased chaperone expression to preserve protein functionality [52], [53]. The retention of a complete TriC/CCT complex (Figure S13), in contrast to the significant losses in other chaperone families, suggests that it may play an important role in maintaining the stability of the similarly highly derived microsporidian proteins.

A variety of proteins and protein complexes involved in ER-Golgi transport within the proximal part of the secretory pathway were detected in *T. hominis* and the other microsporidian genomes. Comparison with the yeast ER-Golgi machinery revealed that coated vesicle trafficking via COPII export and COPI mediated retrieval and associated mechanisms have all been retained (Figure S1), as are a diversity of guanine nucleotide exchange factors/ARFGAPs that regulate this part of the secretory pathway. Our analyses are consistent with electron microscopic observations of coated buds found in relation to the tubulovesicular elements of Golgi-like organelles in *Paranosema* spp. [54]. Taken together these data emphasise the fundamental importance for eukaryotes: even highly reduced ones, of the ER-Golgi trafficking machinery. By contrast, we did not detect a wide range of endocytic and autophagic pathway genes, consistent with simplification or loss of these pathways.

Core carbon metabolism in *T. hominis* (Figure S1) appears to mainly comprise the interconnected pathways for glycolysis, the pentose phosphate pathway, and trehalose biosynthesis and catabolism. With the exception of *E. bieneusi*, which appears to have lost all three pathways [11], [55], these pathways are also strongly conserved in the other microsporidians (Table S11). We also detected genes (Table S12) for an alternative oxidase and glycerol-3-phosphate dehydrogenase that may function to oxidise cytosolic NADH and hence regenerate the NAD^+^ that is needed to maintain glycolysis [56], [57]. These core pathways could provide cytosolic ATP, the reduced coenzymes NADH and NADPH needed for cellular reductive metabolism and biosynthesis, as well as substrates that can feed into other pathways (Figure S1), including glycerophospholipid biosynthesis, amino sugar and nucleotide metabolism.

Like many parasites [58], *T. hominis* has lost the ATP-expensive pathways for the *de novo* biosynthesis of inosine 5′-phosphate (IMP) and for uridine 5′-phosphate (UMP); the starting points for the biosynthesis of purines and pyrimidines for DNA and RNA biosynthesis (Figure S1). To compensate, *T. hominis* has a range of salvage pathway enzymes to make IMP and UMP and other nucleotides and nucleosides, given starting substrates such as ATP, CTP or GTP (see below).

**Figure 6:**
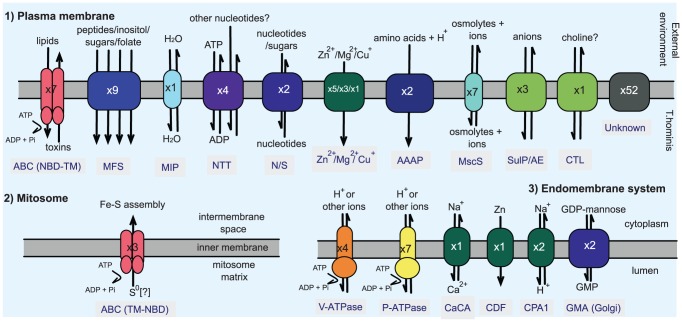
Schematic overview of the transporter repertoire of *T. hominis*. An overview of predicted *T. hominis* transporters with their possible locations in (1) the plasma membrane, (2) the mitosome, and (3) other endomembranes. The number of predicted proteins of each type is indicated in the icons; the predicted transport substrate (s) are also shown. Details of the predicted enzymes (EC numbers and descriptions) as well as the transporters (TC numbers and descriptions) are provided in the Tables S15 and S8, respectively.

The loss of genes means that many *T. hominis* metabolic pathways (Figure S1) are missing or incomplete, so any substrates it still needs, but cannot make, must be imported from infected host cells. We therefore investigated the diversity of *T. hominis* transport proteins. We identified 66 proteins in *T. hominis* with putative homology to 22 previously characterized transporter families [59] (Figure 6, Figure S11, Tables S14 and S15). This represents a reduction in the number and diversity of transporters compared to yeast (Table S15) [60]; which contains 318 transporters assigned to 50 different families (Figure 1), reflecting the greater diversity of yeast metabolism. In many eukaryotes, the major facilitator superfamily (MFS) and ATP binding cassette (ABC) transporters (Table S14) are present as multiple subfamilies with a broad range of substrate specificities [61], [62]. By contrast, *T. hominis* has retained only putative sugar-, folate- and peptide-transporting MFS transporters (Table S14, Figure S12), and only two groups of ABC transporters: including those which are typically found in mitochondria (ABCB and ABCG [63], [64]; Figure S13). Comparison of the predicted transporters for *T. hominis* with other microsporidians (Figure S11, Table S14) suggests that microsporidians retain a similar repertoire of core transport proteins.

The cellular locations of the putative *T. hominis* transporters were inferred based upon their similarity to transporters from model organisms: this information is summarised in Figure 6 and Table S14. Based upon these data and the functional annotation of homologues from model organisms, it appears that the transporters can complement the reduced metabolism and capacity for biosynthesis of *T. hominis*. Thus, putative peptide (MFS), amino acid (AAAP) and folate transporters (MFS), together with an apparently complete *T. hominis* proteasome complex (Figure S1) for amino acid recycling, and enzymes able to interconvert between some amino acids, can potentially compensate for the loss of *de novo* amino acid biosynthesis and other metabolic deficiencies (Figure S1). For example, given imported glutamate and cysteine, *T. hominis* can make glutathione, which, coupled with glutathione reductases and peroxidases, thioredoxin reductases and a superoxide dismutase, appear to make up the main *T. hominis* cellular reductant and detoxifying systems (Figure S1, Table S12). With imported choline and ethanolamine *T. hominis* can make the important phospholipids phosphatidylethanolamine, phosphatidylserine and phosphatidylcholine (glycerophospholipid metabolism, Figure S1). The *T. hominis* homologues of the *E. cuniculi* surface-located NTT transport proteins [45] could potentially import host ATP for energy, and ATP and other nucleotides needed for purine and pyrimidine biosynthesis. Thus, although active transport of GTP and GDP by the *E. cuniculi* NTT transport proteins has not been demonstrated, they do act as competitor substrates reducing ATP transport by the NTT proteins when they are expressed in *E. coli* [45].

In addition to homologues of known eukaryotic transport proteins, we also identified 52 *T. hominis* hypothetical proteins with two or more TMD typical of transporters (Table S14), which may further expand the transporter repertoire of *T. hominis*; 10 of these are conserved across the sequenced microsporidian genomes suggesting they may be of general importance for microsporidian biology. Two of these hypothetical proteins were identified in the spore proteomics data (Table S19).

Proteases often function as important virulence factors in pathogenic organisms, attacking host defence proteins directly [65] or interfering with their transcription [66]. We identified 66 putative proteases in *T. hominis* (Table S16), 24 of which do not have homologues in *E. cuniculi*. Based on the presence of predicted signal peptides, a number of these proteases may be secreted, and may represent candidate effector proteins (Table S16). One of the *T. hominis*-specific proteins, a candidate LGT, is a putative endomembrane metalloprotease of the M79 family [67] and it shares the seven TMD with its bacterial homologues (Figure S14). This protein is most similar to a protease from the opportunistic pathogen *Staphylococcus hominis* (Figure S14, Table S17), which provides resistance against bacteriocin-mediated attacks from other bacteria [68].

Although the metabolism of protist parasites such as *Entamoeba histolytica* [69], *Leishmania major* [70], *Trypanosoma brucei* [71] and *Trichomonas vaginalis* [17] has been significantly affected by LGT, this does not appear to be the case for *T. hominis*. Beyond the piggyBac element, the M79 metalloprotease and the functionally important nucleotide transport proteins already discussed, we identified weak evidence for only two additional candidate LGTs of bacterial origin: a cytidylate kinase (Table S12), and an asparagine synthetase A gene; the latter is found, among eukaryotes, almost exclusively in parasites (Table S18). The relative paucity of LGTs affecting microsporidians may reflect the barriers to transfer posed by their impermeable spores and obligate intracellular lifestyle.

### Are microsporidians derived from fungi or their sister group?

The evolutionary relationship of Microsporidia to fungi, either as sister group or internal branch within the fungal radiation, has been extensively debated over the years [1]. The main difficulties for inferring microsporidian relationships stem from their highly divergent molecular sequences and the challenges these represent for reliable phylogenetic analyses [1], [72]. As a consequence, there has been a search for different types of data that might help resolve the controversy. Thus, a specific relationship of microsporidians to zygomycete fungi has been suggested [73] based upon the apparent conservation of synteny in *E. cuniculi* and *E. bieneusi*, for three genes found at the sex-determining locus of zygomycetes. The genes involved are a high mobility group (HMG) transcription factor flanked by a triose-phosphate transporter (TPT) and an RNA helicase (Hel). However, the hypothesis that microsporidia and zygomycetes are specifically related was challenged by analyses [74] showing that the TPT and Hel genes of zygomycetes and Microsporidia were not orthologous, suggesting that the observed synteny was the result of convergence. We used the *E. cuniculi* HMG, TPT and Hel proteins to search the predicted proteomes of *T. hominis* and *N. ceranae* to identify homologous sequences in these genomes. *Trachipleistophora hominis* encodes one HMG and TPT gene; these are the orthologues of the relevant sequences in *E. cuniculi*. Although *T. hominis* encodes multiple helicase homologues, only one of these is orthologous with the relevant *E. cuniculi* sequence, as confirmed by reciprocal best BLAST hits and our helicase phylogeny (Figure S15). The *T. hominis* and *N. ceranae* sequences formed part of a microsporidian clade that, in agreement with Koestler and Ebersberger [74], is not specifically related to zygomycetes (Figure S15). In addition, the *T. hominis* HMG, TPT and Hel genes (orf_470, 1491 and 432, respectively) are located on different scaffolds, several open reading frames (4, 7, and 5, respectively) away from the closest scaffold edge, and thus they are not syntenic. In summary, the new data from *T. hominis* provide no support for the hypothesis [73] that microsporidians originate from within the fungal radiation as the specific relatives of zygomycetes.

### A minimal mitochondrion: the *T. hominis* mitosome

When the *Encephalitozoon cuniculi* genome was published [6], it was apparent that very few mitochondrial pathways were conserved in this species; indeed at that time it was still not clear whether microsporidians actually retained a mitochondrial organelle. A tiny double membrane bounded remnant mitochondrion (now generally called a mitosome) was discovered in *T. hominis* [14] by immunolocalisation of the mitochondrial Hsp70 and ulstrastructural cell imaging, and the same approach was subsequently used to identify the *E. cuniculi* version [45]. Based upon published electron micrographs [75] it seems that other microsporidians also contain structures that closely resemble the mitosomes of *T. hominis* and *E. cuniculi*, although their identities as homologous structures still needs to be confirmed. We analysed the *T. hominis* genome for pathways that are typically found in mitochondria, but like *E. cuniculi*, it appears that *T. hominis* has lost almost all of the functions of canonical mitochondria including those for ATP generation and cofactor recycling. The only pathway that is strongly conserved in the genomes of *T. hominis* and *E. cuniculi* [6] is for mitochondrial iron-sulphur cluster biosynthesis; key proteins of which have already been localized to the mitosomes of *E. cuniculi* and *T. hominis* [15]. These data suggest that Fe-S cluster biosynthesis is a major metabolic function of the mitosomes of both species.

In model eukaryotes, the mitochondrial pathway for Fe-S cluster biosynthesis is also needed to make essential cytosolic and nuclear Fe-S cluster-containing proteins, including Rad3 and Rli1 and DNA polymerase [76], [77]; proteins that are encoded by the *T. hominis* genome. Three *T. hominis* proteins show high similarity to the yeast Atm1 ABC transporter (Table S8), which provides an essential link between mitochondrial and cytosolic Fe-S cluster biosynthesis [76], further supporting a role for the *T. hominis* mitosome in this pathway. By contrast, the complete absence of *T. hominis* genes for members of the mitochondrial carrier family, which support important steps in mitochondrial Fe-S cluster biosynthesis of model eukaryotes [76], raises important questions about how the substrates (NADH, cysteine and iron) needed for mitosomal Fe-S biosynthesis are imported into *T. hominis* mitosomes.

Consistent with the apparent absence of electron transport chain components in the *T. hominis* genome, we found no evidence for a mitochondrial (mitosomal) genome in our assembly. All of the proteins in *T. hominis* mitosomes [14], [15] must therefore be synthesised in the cytosol and targeted to, and imported into, the organelle. We detected components of a minimal mitosomal protein import machinery for *T. hominis* comparable to that described for *E. cuniculi* [78], comprising core components of the outer membrane translocase (Tom40, Tom70, Sam50), inner membrane translocase (Tim50, a single divergent Tim17/22/23 homologue) and the PAM motor complex (Hsp70, Pam18, Jac1), needed to complete protein import and refolding [79]. We did not detect any components of the mitochondrial intermembrane space import and assembly (MIA) pathway or any intermembrane space chaperonins. Nor did we detect a *T. hominis* homologue of the Imp1/2 processing peptidase used by yeast for import of substrates like G3PDH to the inner membrane [80]. A single homologue of Imp1/2 has previously been detected for the microsporidian *Antonospora* (*Paranosema*) *locustae* [80] and in this case G3PDH does appear to be localised to its mitosomes [81]. *Antonospora* (formerly *Paranosema*) *locustae* also has a mitosomal alternative oxidase [81] that may function with the G3PDH to regenerate NAD+ for the cytosol. The *T. hominis* alternative oxidase carries an N-terminal targeting signal that can guide import into *Saccharomyces cerevisae* mitochondria [57], but the native protein has not been localized in *T. hominis*. We did not detect subunits of the mitochondrial processing peptidase (MPP) typically used to process N-terminal targeting signals [82], and the few proteins that have experimentally verified as located to the *T. hominis* mitosome appear to lack such signals [14], [15]. The complete loss of the MPP would represent an additional level of loss compared to the reduced mitochondrial homologues of *Giardia* (mitosomes) and *Trichomonas* (hydrogenosomes), which have retained one or both MPP subunits, respectively [82], [83].

### Proteomics analysis of *T. hominis* spores

**Figure 7:**
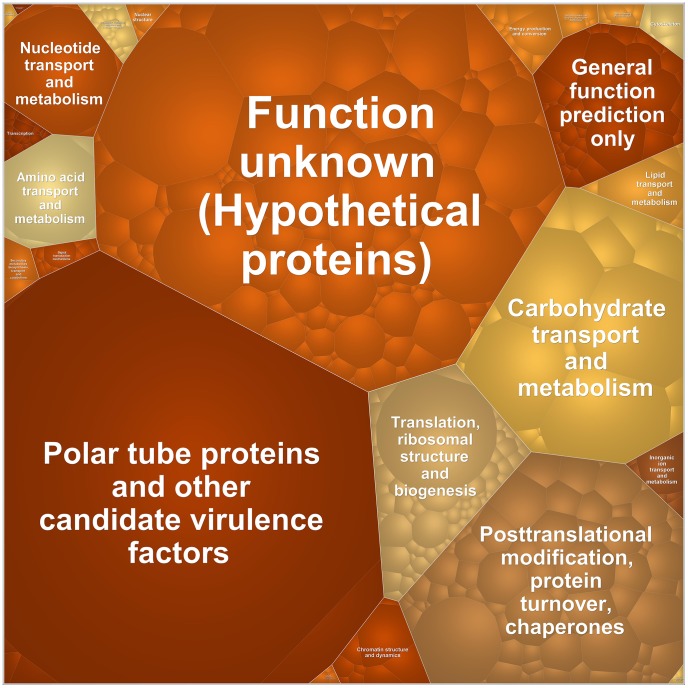
Major functional categories of proteins detected in the proteomics analysis of *T. hominis* spores. Voronoi treemap [132] illustrating the major COG categories, complemented with an ad hoc category for putative *T. hominis* “virulence factors”, detected in the spore proteomics data. Each functional category and individual protein, for a total of 484 proteins (Table S19), are represented by delineated cells. The area of the different cells is proportional to the relative abundance of the proteins according to spectral counts. The locus tag and protein annotation for each individual protein are illustrated in the Voronoi treemap shown in Figure S16. Protein annotation, including predicted signal peptides and transmembrane domains, semi-quantitative spectral counts and presence of homologues of the 484 proteins in the other 5 microsporidians analysed, are provided in Table S19.

The only microsporidian life cycle stage able to survive outside of another eukaryotic host cell are resistant spores that act as both dispersal and survival stages [2]. To investigate the *T. hominis* spore proteome, we collected spores from the culture medium of multiple *T. hominis*-rabbit kidney cell co-cultures over a 3-month period, and, after extensive purification to remove host cell material, subjected the accumulated spores to proteomics analysis. Our analyses identified 484 *T. hominis* proteins (Table S19), representing approximately 15% of the predicted ORFs for *T. hominis*. In terms of the relative abundance of spectral counts, the data were dominated by polar tube proteins and by hypothetical proteins, some of the most strongly represented of which appear to be unique to *T. hominis* (Figures 7 and S16, Table S19). Some COG functional classes were overrepresented in the proteomics data when compared to their representation in the *T. hominis* genome using a hypergeometric test; these classes are illustrated in the Voronoi treemap (Figure 7, Table S19). As might be expected for a non-replicative life cycle stage, proteins involved in cell cycle control, cell division and chromosome partitioning, and in DNA replication, recombination and repair were underrepresented in the spore proteomics data (Table S19). Notably, the types of proteins detected in the *T. hominis* spore proteome are very similar to the 177 proteins detected in the proteomics study of *Encephalitozoon cuniculi* late sporogonal stages [84], suggesting the two studies provide a useful, albeit incomplete, snapshot of typical spore protein content.

The polar tube of microsporidian spores is a highly specialised extrusion apparatus that acts as the conduit through which the microsporidian sporoplasm can pass into a new host cell [85]. It is thus a key component of the microsporidian infection apparatus. In *E. cuniculi*, three polar tube proteins have been characterised [85], [86], two of which – PTP3 and PTP2 – were found in the *T. hominis* proteomics data. We did not detect a *T. hominis* homologue of PTP1 in our genome annotation. This could be due to the *T. hominis* assembly being incomplete, or because the *T. hominis* PTP1 homologue was too divergent to be recognised; polar tube proteins are notoriously difficult to identify based solely on sequence similarity [3], [86]. For example, in *N. ceranae*, putative PTP1 and PTP2 genes were identified based upon a combination of features including their close physical juxtaposition to each other, the presence of a predicted signal peptide, and their similar length and predicted amino acid compositions relative to other PTP1/PTP2 proteins [3]. In our case, the *T. hominis* PTP2 gene was located at the end of a scaffold (scaffold #00193) so its genomic context is incomplete, and none of the annotated *T. hominis* proteins had the combination of length, signal peptide and amino acid composition sufficient to be a strong candidate for PTP1. At least two other proteins detected in the proteomics data may be relevant to the infection process. Tetraspanins mediate a wide variety of membrane associated functions [87] and among fungi are involved in ascospore germination in *Podospora anserina* [88], and in the invasion of host cells by the fungal plant pathogen *Magnaporthe grisea* [89]. Subtilisin-like proteases are important for the release of *Plasmodium falciparum* from erythrocytes [90] and two subtilisin-like proteases have already been shown to be upregulated in *E. cuniculi* during sporont differentiation [91]. The presence of tetraspanin and a subtilisin-like protease in *T. hominis* spores, and the conservation of both types of protein in the genomes of the other microsporidians investigated (Table S19), suggests that these proteins may also have important roles in microsporidian physiology and infection.

**Figure 8:**
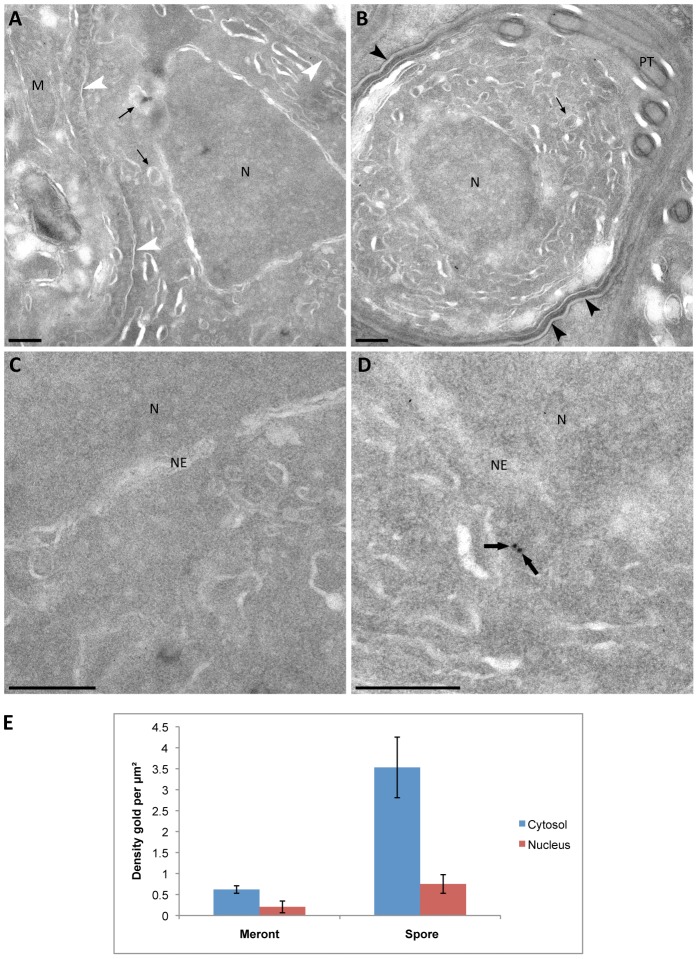
Quantitative immunoloelectron microscopy of the glycolytic enzyme phosphoglycerate kinase (PGK-3) in thawed cryosections of *T. hominis* meronts and spores. Cryosections of *T. hominis* labelled with anti-PGK-3 antiserum were sampled with micrographs taken at systematic uniform random (SUR) locations. The micrographs presented in the figure are representative of the quantitative data. (A) *T. hominis* in proliferative meront phase. White arrowheads indicate the plasma membrane of the parasite. (B) *T. hominis* early spore stage. Black arrowheads indicate the forming cell wall. (C) and (D) Enlarged image details from micrographs (A) and (B) respectively, demonstrating the difference in immunolabelling. In enlargement (D), PGK-3 labelling inside the cytosol can be seen (see black arrows). M = mitochondrion of the host rabbit kidney cell; Small arrows = mitosomes; PT = polar tube; N = nucleus; NE = nuclear envelope; bars = 200 nm. (E) Mean densities of PGK-3 gold labelling from three individual experiments. The area of compartments in *T. hominis* meronts and spore stages was estimated using point counting (see Materials and Methods) and counts of 10 nm gold made (n = 44 micrographs for spores, n = 51 micrographs for meronts; mean point counts were 92/experiment and mean gold counts 56/experiment). Error bars indicate the standard error of the mean.

Glycolysis and trehalose catabolism are thought to be important for microsporidian spore survival and germination [2], [56], and, consistent with this hypothesis, neutral trehalase and most of the proteins needed for glycolysis, the alternative oxidase and glycerol-3-phosphate dehydrogenase, were represented in the proteomics data (Figure 7, Figure S16, Table S19). To investigate further, we also quantified the mean densities of gold labelling for phosphoglycerate kinase (PGK-3), in *T. hominis* meronts and spore stages (Figure 8). This enzyme catalyses the first ATP-forming reaction in the “pay-off” stage of glycolysis. There was a clear enrichment of gold particles for PGK-3 in the cytosol of spores compared to the cytosol of meronts (Figure 8), consistent with a role for glycolysis in *T. hominis* spore ATP generation. These findings support previous data for metabolic enzymes of *Antonospora locustae*, which suggest that glycolysis is mainly used to make ATP in spores rather than vegetative cells [81], which rely instead upon surface-located nucleotide transport proteins to steal host ATP rather than make it themselves [45]. Interestingly, we did detect one such nucleotide transport protein in the proteomics data (orf_2860, Table S19); if this protein were expressed on the surface membrane of an invading *T. hominis* sporoplasm it would allow the immediate exploitation of host energy resources.

Homologues of the yeast protein Ynk1 are conserved in all of the microsporidian genomes investigated, and the *T. hominis* protein was strongly represented in the proteomics data (Figure S16, Table S19). This protein, a nucleoside diphosphate kinase (EC 2.7.4.6), has a central role in purine and pyrimidine metabolism, because it can reversibly convert a broad range of nucleotide diphosphates and deoxynucleotide diphosphates into the corresponding nucleotide triphosphates and deoxynucleotide triphosphates, for DNA, RNA and cofactor biosynthesis (Figure S1). Proteins that function in post-translational modification, protein turnover and chaperonins, were also strongly represented in the *T. hominis* spore proteomics (Figure 7, Table S19), suggesting that protein quality control and cellular detoxification are important spore processes. We detected components of the *T. hominis* proteasome for protein degradation, and cyclophylin, chaperonins of the Hsp70 family and TriC/CCT complex needed for ensuring reliable protein folding (Table S19). The overrepresentation of glutaredoxins, glutathione and thioredoxin metabolising enzymes in the *T. hominis* spore data, as well as in the published *E. cuniculi* proteomics data [84], suggests that these cellular defence systems play an important role in maintaining spore homeostasis and viability.

### Conclusions

Microsporidians are a large and successful group of obligate intracellular parasites, but, despite their importance and diversity with respect to known hosts, developmental cycle, and genome size, relatively few genomes are available, and the vast majority of species have never been cultivated. The limited sample of microsporidian genome diversity means that there are major gaps in our understanding of how microsporidians have made the transition from a free-living lifestyle to an obligate intracellular one. We have now analysed the gene content, genome architecture and intergenic regions of the larger genome of *T. hominis*, a microsporidian that was isolated from a HIV/AIDS patient [13]. Our data provide detailed insights into the genome and potential biology of *T. hominis* as a known opportunistic pathogen, and will help facilitate the development of this species as a model microsporidian: it is one of the few species that can be easily co-cultured and manipulated in the lab [14], [15], [45]. In particular, the discovery of the RNAi machinery raises the exciting prospect of down-regulating genes in *T. hominis*, and hence of developing a much needed genetic system for microsporidian parasites.

As an outgroup to previously sequenced microsporidians, the *T. hominis* genome allows a more precise identification of ancestral and derived features in the evolution of microsporidians more generally. Our analyses predict an ancestral microsporidian that was already an intracellular parasite, but one with a relatively large genome populated with diverse repetitive elements and a complex transcriptional regulatory network. The drastic remodelling of the ancestral microsporidian proteome involved the extensive loss of protein families but also the gain of new ones; many of these new protein families have since been maintained against a background of reductive evolution. This conservation suggests that these proteins are worthwhile targets for investigating aspects of microsporidian biology that are still poorly understood, including the infection process itself, the interplay between parasite and host metabolism, and the manipulation of host organelles [1]. Comparing the genomic features of *T. hominis* with other microsporidians clearly demonstrates that the reductive evolution of coding capacity and genome architecture were not coupled. The major changes to the proteome occurred in the common ancestor of Microsporidia and were contemporaneous with the gain of the nucleotide transport proteins that are used to steal host-generated ATP [45] and are intimately associated with the microsporidian intracellular parasitic lifestyle. By contrast, the reduction in intergenic regions and the loss of canonical eukaryotic regulatory features occurred later, and only in some lineages. Any notion that intracellular parasitism inevitably leads to tiny compacted genomes is not supported by our analyses; although drastic gene loss, simplified cell structures and reduced metabolism appear common to all microsporidians, the genome of *T. hominis* is actually less gene dense than yeast.

## Materials and Methods

### Organism growth and DNA isolation

*Trachipleistophora hominis* [13] was grown in RK-13 cells at 37°C in Dulbecco's Modified Eagle Medium (DMEM), containing Kanamycin 100 ug/ml, Penicillin 100 ug/ml, Streptomycin 100 ug/ml, and Fungizone 1 ug/ml. Spores were harvested and pooled over several months; DNA was extracted from approximately 1.25E+10 spores. To obtain spores, RK-13 cells infected with *T. hominis* were washed, incubated in double distilled H_2_0 overnight at 4°C, washed to remove host cell debris, and re-suspended in 10 ml of proteinase K buffer (0.5% SDS with proteinase K 250 µg/ml; Roche) and incubated overnight at 37°C. The spores were washed with double distilled H_2_0 and pelleted at 500× g for 5 min to enrich for live spores. The spores were re-suspended in 5 ml of 1× DNAse buffer with 100 units of DNAse (Roche) and incubated for 2 days at 37°C, washed with ddH2O, and re-suspended in cell lysis solution (Puregene Tissue Core A kit, Gentra, Qiagen). Spores were mixed with glass beads (0.1–0.11 mmø, Braun Biotech international GmbH) and bead-beaten for 3×45 sec at 6.5 m/sec (Bio101 FastPrep 120). Beads were removed by a quick spin, and 1.5 µl RNase A solution (100 mg/ml, Qiagen) was added. Tubes were inverted several times, placed on ice for 1 min, protein precipitation solution was then added, mixed vigorously and incubated on ice for 5 min. The samples were then centrifuged at 13,000× g for 3 min, the supernatant transferred to a new tube, and isopropanol carefully added. The tubes were repeatedly inverted to mix and centrifuged at 13,000× g for 5 min at 4°C, and the resulting pellet was washed with 70% ethanol once. The DNA was dried at room temperature and rehydrated with DNA hydration solution (Puregene Tissue Core A, Gentra, Qiagen). The genomic DNA was screened for potential contamination by PCR, details for all primers are given in Table S17.

### Sequencing and assembly

For the 454 sequencing, 2 µg of DNA was sheared to 500 bp by nebulisation (following the manufacturer's protocol; Roche) and was sequenced on half a picotitre plate. To generate a paired-end library, 3 µg of DNA was sheared using a HydroShear (DIGILAB) to 3 kb and gel extracted. For the SOLiD library, 100 ng of DNA was sheared to an average length of 150 bp using a Covaris S200 following the manufacturers protocol. The 454 sequencing was performed on a Roche 454 FLX with titanium chemistry according to the manufacturers instructions, and resulted in a total of 378,359,925 bases in 1,748,948 reads of combined single and paired-end read data and with an average length of 216 bp. SOLiD sequencing was performed on the ABI-SOLiD version 3.5, resulting in 83,005,155 reads of an average length of 50 bp, and 43,400,944 reads were of sufficient quality after filtering with the csfasta quality filter (Applied Biosystems). Mapping of SOLiD reads onto the assembled 454 data was performed using the software package Nesoni 0.28 (Monash University Victorian Bioinformatics Consortium, http://bioinformatics.net.au/software.nesoni.shtml), using Shrimp [92] as the aligner. The consensus sequences were called from Nesoni, and after evaluating different parameters and manual inspection; we used a read depth of 10 and purity of 0.6 without ambiguity codes. The genome sequence data and corresponding gene annotations reported in this paper have been submitted to the NCBI GenBank database (NCBI BioProject: 84343).

### ORF annotations and characteristics

Gene prediction was performed using the Genemark suite, and initial gene annotation was performed automatically using the PEDANT annotation software with default parameters [93] and further information was derived from predictions with InterProScan version 4.7 [94] with Pfam release 24.0 [36], SignalP 3.0 [95] and TMHMM 2.0 [96]. Gene lengths based on amino acids were compared between orthologous pairs in *S. cerevisiae*, *E. cuniculi* and *T. hominis* by a Wilcoxon Rank-Sum test. Codon usage was investigated by the programs CUSP and CAI from the EMBOSS package [97] as well as an in-house Python script.

### Splicing machinery and introns

Introns were identified by manually inspecting the coding sequences of *T. hominis* genes whose orthologues in *E. cuniculi* or *N. ceranae* contain introns (Table S18). We then built an intron consensus motif by combining the results of recent analyses in *E. cuniculi* [20] and *N. ceranae* [3] with our manual annotation of introns based on *E. cuniculi* or *N. ceranae* orthologues. This motif was used to scan the *T. hominis* genome, identifying an additional set of introns that were missed in the initial survey (Table S19 and Figure S16).

### Lateral gene transfer (LGT)

Phylogenetic trees for all *T. hominis* protein sequences were calculated using an adapted version of the PhyloGenie pipeline [98] with a maximum threshold of 0.9 on genus level. Trees were calculated using RAxML [99] with the JTT [100]+GAMMA model. 100 bootstrap replicates were performed using the fast bootstrap option of RAxML. Trees with unusual taxonomic relationships (indicating LGT) were selected using the phat.jar tool implemented in PhyloGenie and investigated further.

### Repeats and transposable elements

Identification of repeats was performed using RepeatModeler 1.0.5 (http://www.repeatmasker.org/RepeatModeler.html) on both the concatenated DNA sequence as well as individual scaffolds with the default settings, except for requiring putative new repeat families to be represented by at least 15 homologous members, in order to reduce the possibility of false positives. Searches for known repeats were performed with RepeatMasker 3.2.9 (http://www.repeatmasker.org/) by the integrated program cross_match against the custom set of repetitive families as well as the full set of eukaryotic repetitive families from the curated RepBase database [101] using the *T. hominis* DNA (concatenated as well as individual scaffolds) as input and with default settings. To identify putative TE proteins in the genome, RepeatMasker hits overlapping with ORFs were identified. These DNA-level analyses were supplemented with PHMMER searches to identify predicted proteins with homology to previously described TE proteins. Since many TE-encoded proteins are fragments, we performed two rounds of tree-building: First, all sets of homologous TE proteins from the *T. hominis* genome were included in the trees, then, the longest protein from each of these clusters was chosen as a representative for the final round, in order to maximize the number of alignable positions for inferring the tree.

### Genome structure

100 bp upstream of predicted ORF start codons were searched with the canonical pattern TATA[AT]A[AT][AG] based on the *S. cerevisiae* sequence [102] and CCC motif based on the *N. ceranae* sequence. A search with the MEME program [103] was performed using the default settings and a maximal number of 40 motifs to be reported, and subsequently analysed with TOMTOM [104] against the databases JASPAR, TRANSFAC and UNIPROBE using the default settings.

### Clustering analyses

For *de novo* protein cluster analysis we used the proteomes of *Homo sapiens* (20,245 sequences), *Saccharomyces cerevisiae* S288c (5,863 sequences), *Nosema ceranae* BRL01 (2,060 sequences), *Enterocytozoon bieneusi* H348 (3,632 sequences), *E. intestinalis* ATCC 50506 (1,833 sequences), *E. cuniculi* GB-M1 (1,996 sequences) and *Trachipleistophora hominis* (3,266 sequences, this study). The human proteome dataset was derived as the isoform-free version from Uniprot (http://www.uniprot.org/) whereas the other sequences were downloaded from RefSeq [105]; all downloads were performed on the January 2011 release.

Clustering was performed using Markov Clustering (MCL [42]), and PHMMER ([43]; HMMER version 3.0) was used for sequence similarity searches. The input values were e-values from PHMMER from all-against-all searches with an e-value cut-off of <0.01 and a pairwise-alignment length cut-off of 0.5. The input protein pairs were clustered into protein families using the MCL algorithm by setting the inflation parameter (I) from 1.0 to 10.0 in increments of 0.2. To investigate which inflation parameter produced the best clustering result, the F-measurement [106] was used for comparing the results against a reference clustering. The reference clusters included 15 well-characterized proteins with different levels of conservation across the Microsporidia, *S. cerevisiae* and *H. sapiens*. These included the broadly conserved proteins DNA polymerase *alpha* and *delta*, RNA polymerase, nucleoporins (Nup) 170, members of the TRiC/CCT chaperone family, pyruvate kinase, glycerol-3-phosphate dehydrogenases, phosphoacetyl-glucosamine mutase, superoxide dismutase 2, and iron sulphur cluster assembly protein Isd11. Reference protein families that had no homologues in microsporidians included Nup84, Nup188, and Nup192. We also included polar tube protein 2 (PTP2) in the reference set as a representative of a microsporidian-specific protein family. Based on these analyses, we chose an inflation rate of I = 1.5, which yielded an F-measure of 0.915. This setting produced protein clusters that were most similar to the pre-defined clusters of characterised protein families given in the reference set.

Microsporidian sequences are often highly divergent, and the MCL clustering produced many microsporidian-specific protein families that showed significant sequence similarity to clusters containing only yeast and human sequences. We therefore used HMM profiles built from the microsporidian-specific clusters to perform sensitive similarity searches against the non-microsporidian singletons and clusters, and merged clusters when a unique HMMER hit (with E<1E-05, determined by manual inspection) was established between the microsporidian-only clusters to the non-microsporidian clusters or singletons.

To infer putative functions for the identified clusters, we used the COG annotation of the human or yeast homologue present in the clusters. In cases were a cluster did not contain a human or yeast sequences, all of the cluster members were searched with BLASTP against all proteins in the COG database with an e-value cutoff of ≤0.01. Clusters were assigned a functional COG category if at least 2 members in the cluster hit the same COG. In cases where no COG hit was obtained for clusters inferred to be present in the ancestor of the microsporidians analysed, we tried to infer putative functions using the highly sensitive HHsearch [44]. This was done with default settings and by searching against protein profiles from COG, KOG, CDD, Pfam, Superfamily, SMART, SCOP, PDB, and TIGRfams [36], [107]–[114]; any functional annotation was added based upon on an e-value cutoff ≤0.01 and a probability of >90%.

### Gain and loss of clusters during microsporidian evolution

To investigate the loss and gain of gene families with Pfam annotation we used the Dollo parsimony approach implemented in the Count package [115], [116] to generate a profile of presence or absence of Pfam domains for each taxon over a reference tree [16]. All of the *de novo* protein clusters obtained using the MCL clustering approach described above, were then analysed in the same way to generate profiles of presence or absence of clusters over the same reference tree. Microsporidian-specific gene families were identified as those clusters where no member had a BLASTP hit to any non-microsporidian sequence at an e-value cutoff ≤0.01, when searched against the NCBI RefSeq database.

### Codon usage analysis

Codon usage was measured using the frequency of optimal (F_OP_) codons [39] using R (http://www.r-project.org). Most of the species investigated produced single peaks in F_OP_ plots suggesting a relatively homogeneous codon usage (Figure S7). However, the F_OP_ plot for *E. bieneusi* (Figure S7) was much broader and had two major peaks. To investigate the possible reasons for this distribution, we used BLASTP to search for homologues of the 74 protein sequences containing 69 Pfam domains, that were unique to *E. bieneusi* in the Dollo parsimony analysis (Figure S8). We also analysed their optimal codon usage and compared this to the codon usage of the *E. bieneusi* genome as a whole using a nonparametric Wilcoxon Rank-Sum test.

### Reconstruction of *T. hominis* metabolism; functional classification of proteins and assignment of enzyme classification (EC) numbers

Enzyme classification (EC) numbers were obtained from the automatic annotation in the PEDANT database [93] and combined with HMMER searches of the PRIAM enzyme profiles (release 10. 08. 2010 [117]) with a cutoff e-value of <1E-05. We also used SHARKhunt version 1.0 [118] with shark profiles available for the June 2009 version of PRIAM and the default settings using the DNA sequences as input with a cutoff e-value of <1E-05. Only hits with an ORF region of at least 50 amino acids were assigned EC numbers. The results of these analyses were used to build a *T. hominis* metabolic enzyme database in Pathway Tools [119], with manual curation to take into consideration the degree of completeness of each metabolic pathway. To map the enzymes and proteins identified for *T. hominis* onto KEGG pathways [120] we used BLASTP searches of the *T. hominis* translated ORFs against sequences in the KEGG database [120], with a cutoff e-value of 1E-05. To generate KEGG maps for *T. hominis*, an in-house Python script was used to access the KEGG API version 6.2 (http://www.genome.jp/kegg/soap/).

### Identification of *T. hominis* transporters

To identify potential *T. hominis* transporters, our intial screen focused on proteins with at least two transmembrane domains (TMD) predicted by TMHMM [96], or one TMD but no signal peptide predicted by SignalP [95] were inspected manually. As the highly divergent TMD of parasites are sometimes not recognized by TMHMM, but can be identified in the TMHMM graphical output, additional TMD were assigned to a few *T. hominis* proteins. All proteins with four or more assigned putative TMD were then analysed further. To predict substrate specificities, all putative *T. hominis* transporters were searched using BLASTP for functionally characterised homologues stored in the NCBI nr database, including annotated human or yeast proteins and complemented with InterProScan searches. We also searched the transporter classification database TCDB [59] to obtain information about putative substrates and to classify the transporters according to the TC classification system (Table S8 [59]).

### Single-gene phylogenetic analyses

Phylogenetic analyses based on maximum likelihood were performed with RaxML [99] and PhyML [121] whereas Bayesian analyses were performed using Phylobayes [122] or p4 [123]. Details on the model parameters and run settings are given in the respective Figure legends. To reduce the impact of amino acid composition sequence heterogeneity identified in our alignments, Dayhoff recoding was used [124] combined with the node-discrete compositional heterogeneity (NDCH) model [123], which allows composition to change over the tree. A ChiSquare test as described in [123] was used to test for model fit; if necessary, additional base-composition vectors were included until the model adequately fit the data with respect to compositional heterogeneity.

### Proteomics

*T. hominis* was grown in RK-13 cells and spores harvested from culture media over several months, storing samples in PBS at −20°C. Pooled samples were incubated overnight in sterile double distilled water (ddH_2_0) at 4°C to lyse RK-13 cells and then centrifuged at 2,400× g for 5 min. The supernatant was removed and spores and cell debris were resuspended in 5 ml of PBS, transferred carefully onto a 25∶75 solution of Percoll∶PBS and centrifuged at 900× g for 30 min at 4°C. The pellet was washed twice with PBS and re-suspended in 9 ml of PBS containing 0.05% saponin and 0.05% Triton X-100. The suspension was passed 5 times through a syringe (25G gauge) and subjected to a second Percoll gradient [125]. After centrifugation at 45,000× g for 15 min, two visible bands close to the bottom of the tube were observed: the upper band was enriched with spores. The percoll-purified spores were washed in PBS and broken by bead-beating in tubes containing 400 mg of glass beads (Glasperlen 0.1–0.11 mmØ, B. Braun Biotech international GmbH). The samples were bead-beaten (×3) for 45 sec at speed 6.0 in a Bio101 FastPrep machine in a cold room with cooling of the sample on ice in between. The beads were pelleted at 1,000× g for 1 minute and the supernatant, containing the spore protein raw extract (including soluble proteins as well as fragments of the spore envelope and cellular membranes), was frozen at −80°C until proteomics analysis.

Approximately 20 µg of the protein extract, in a sample buffer containing 2.5% β-mercaptoethanol as a reducing agent, was separated using 1D SDS PAGE and stained with Coomassie Brilliant Blue. The minigel lane was cut into 10 equal pieces (fractions), which were subjected to in-gel digestion [126]. Gel pieces were washed (200 mM NH_4_HCO_3_, 30% acetonitrile) and dried in a vacuum centrifuge before overnight digestion with 2 µg/ml Trypsin solution (Promega) at 37°C. Peptides were eluted with deionized water in an ultrasonic bath for 15 min. The peptide mix was separated by Nano HPLC (Easy-nLCII HPLC system, Thermo Fisher Scientific) followed by MS/MS analysis in an LTQ Orbitrap Velos mass spectrometer (Thermo Fisher Scientific) as described in detail by Elsholz et al. [127]. The MS analyses were performed twice to provide duplicate spectral data (n = 2) for all sample fractions. All of the spectra obtained for the spore proteomics experiments are provided as mzxml files at the Tranche database (https://proteomecommons.org/tranche/), one of the major repository database for raw and meta proteomics data [128] and can be downloaded directly using the following url: https://proteomecommons.org/dataset.jsp?i=A9goHT9L5X88EFSpcYVOQtT2SN%2FcbLYeCMKFL6RqWx%2F8N5WSY2ql5R0ormjK6DvYH3gRUoRY0UgrVsRNOVMYvT8rRVYAAAAAAAAMhg%3D%3D.

For protein identification, tandem mass spectra were extracted using Sorcerer v3.5 (Sage-N Research). All MS/MS samples were analyzed using the Sorcerer-Sequest software (Thermo-Finnigan, version v.27, rev. 11) applying the following search parameters: peptide tolerance, 10 ppm; tolerance for fragment ions, 1 amu; b- and y-ion series; oxidation of methionine (15.99 Da) was considered as variable modification (max. three modifications per peptide). The search was performed against a combined target-decoy database containing the *T. hominis* protein sequences obtained in this study, the *Oryctolagus cuniculus* protein sequences (http://www.ncbi.nlm.nih.gov/bioproject/12819), and common contaminants, such as keratin. An additional search against all available NCBI protein databases was performed to identify spectra that originated from potential additional eukaryotic and prokaryotic contaminants and to verify the purity of our spore preparations. Validation of MS/MS based peptide and protein identifications was performed with Scaffold V3.1.2 (http://www.proteomesoftware.com/Proteome_software_prod_Scaffold3_download-main.html). Peptide identifications were accepted if they passed the following SEQUEST filter: XCorr for doubly charged peptides 2.2, for triply charged peptides 3.3 and for quadruply charged peptides 3.8; Cn score was set to 0.1. To visualize the relative abundances of the identified proteins and their respective COG functional categories, Voronoi treemaps were constructed as described [127] using averaged (n = 2) and normalized spectral counts (Scaffold's “quantitative value”).

### Electron microscopy

Monolayer RK cells (RK-13) were infected with *T. hominis* and grown to near confluency. The cells were fixed in 0.5% glutaraldehyde in 0.2 M PIPES buffer (pH 7.2) for 15 min at room temperature and scraped from the dish and pelleted (15 min at 16.000× g); washed three times with buffer and cryoprotected in 2.3 M sucrose in PBS overnight at 4°C. Small fragments of the cell pellet were then plunge-frozen in liquid nitrogen and 80 nm thick sections were cut at −100°C (EM FC7 ultracryomicrotome; Leica, Vienna, Austria). Sections were mounted on carbon/pioloform-coated EM copper grids (Agar Scientific, Stansted, UK) and stored in drops of 1∶1 pre-mixed 2.1 M sucrose/2% w/v methylcellulose. Prior to labelling, grids were washed in ice-cold distilled water (×3) followed by PBS at room temperature. The sections were then incubated in 0.5% fish skin gelatin (Sigma Aldrich, Poole, UK) in PBS, and labelled using rabbit antiserum against PGK-3 followed by 10 nm protein-A gold and contrasted using 2% w/v methylcellulose/3% w/v uranyl acetate. For quantification, labelled sections were sampled systematic uniform random (SUR; [129], [130]) in three individual experiments by taking 23–39 micrographs per sample with a JEOL 1200 transmission electron microscope on Ditabis imaging plates (DITABIS Digital Biomedical Imaging Systems AG, Pforzheim, Germany) at a nominal magnification of 40 or 50 K. Tiff files of micrographs were further analysed using Adobe Photoshop CS4. Randomly placed square lattice grids were placed on each micrograph and used to estimate the areas of interest (cytosol and nucleus of *T. hominis* meronts and spore stages respectively) by point counting (grid spacing 262 nm at 40 K and 205 nm at 50 K for the nucleus and 655 nm at 40 K and 205 nm at 50 K for the cytosol which yielded a total of 51–360 points per experimental condition). *T. hominis* meronts could be identified as single or multinucleated cells growing in the RK cells. Spore stages (including earlier sporont stages as well as later sporoblasts and fully matured spores as described by Hollister et al. 1996) could be identified based on the presence of a discernible cell wall and/or the formation of the polar tube as well as the surrounding parasitophorous vesicle. Cytosol was defined as any area lying between the plasma membrane, internal organelles and outer nuclear envelope; the nucleus was defined as any area limited by the inner nuclear envelope membrane. Gold particles lying over profiles of internal organelles such as the polar tube or the lamellar polaroplast were not included in the quantification.

## Supporting Information

Figure S1 KEGG maps for *Trachipleistophora hominis*. The KEGG maps were generated based on the curated annotation of the *T. hominis* genome. The results from enzyme annotation, profile-profile search and pairwise sequence similarity searches were used to annotate putative genes on the KEGG maps. Yellow boxes indicate the presence of a respective *T. hominis* protein; red letters indicate manually annotated EC numbers, blue letters indicate proteins assigned by BLAST searches (cut off ≤1E-05) against all proteins in the KEGG database, and violet letters indicate manual annotation following the HHSearch analyses of the clusters inferred to be present in the common microsporidian ancestor.
(PDF)

Figure S2 Sequences of putative introns in selected *T. hominis* genes. Predicted 5′ and 3′ splice sites (5′SS and 3′SS, respectively), the branch point (pbA) as well as the trinucleotide threshold area (3 nt) are indicated. The relaxed regular expression GTA[AG]GT[ATGC]+?TAATT[ATGC]{0,4}AG was used to identify putative *T. hominis* introns, following a comparison of *T. hominis* genes with intron-containing genes from *N. ceranae* and *E. cuniculi* (See also Tables S2 and S3). Scavenger is an mRNA decapping enzyme.
(PDF)

Figure S3 Identification of 40 potential regulatory motifs in the non-coding regions of the *T. hominis* genome. Details on the motifs and their similarity to known motifs are given in Tables S4 and S5.
(PDF)

Figure S4 Relationships of helitron sequences from *T. hominis* and *N. ceranae* to those from other eukaryotes. The weakly supported tree favours a common origin for *N. ceranae* and *T. hominis* helitron elements, suggesting that helitrons were present in their common microsporidian ancestor, but does not confidently identify their closest relatives among other eukaryotic helitrons. The tree shown is from a Bayesian analysis of Dayhoff-recoded amino acid sequences performed using p4 and the node discrete compositional heterogeneity model ([123], NDCH) with two base composition vectors needed to fit the data; support values are Bayesian posterior probabilities. The tree was calculated over 2 million generations using the ‘auto-tune’ setting, and compositional fit was tested as previously described ([123]. The accession number (gi) or the *T. hominis* ORF locus tag are given for each sequence in the tree.
(PDF)

Figure S5 Relationships of non-LTR sequences from *T. hominis* and *N. ceranae* to those from other eukaryotes. The tree strongly supports the monophyly of *N. ceranae* and *T. hominis* non-LTR elements, suggesting that these elements were present in their common microsporidian ancestor. The topology also demonstrates that expansion of non-LTR elements has occurred in *T. hominis*. The tree shown is from a Bayesian analysis of Dayhoff-recoded amino acid sequences performed with p4 using the NDCH model with two base composition vectors. Further details of the analysis are given in the legend to Figure S4. The accession number (gi) or the *T. hominis* ORF locus tag are given for each sequence in the tree.
(PDF)

Figure S6 Relationships of microsporidian LTR sequences to those from other eukaryotes. The tree strongly supports the monophyly of *N. ceranae*, *N. bombycis* and *T. hominis* LTR elements, suggesting that these elements were present in their common microsporidian ancestor. The tree shown is from a Bayesian analysis of Dayhoff-recoded amino acid sequences performed with p4 using the NDCH model with two base composition vectors. Further details of the analysis are given in the legend to Figure S4. The accession number (gi) or the *T. hominis* ORF locus tag are given for each sequence in the tree.
(PDF)

Figure S7 Phylogenetic analysis provides evidence for LGT of a piggyBac transposon between an ant and *T. hominis*. The tree suggests that LGT of a piggyBac transposon has occurred between the ancestors of the ant *Harpegnathos saltator* and *T. hominis*. The tree shown is from a Bayesian analysis of Dayhoff-recoded amino acid sequences performed with p4 using the NDCH model with an additional base composition vector. Further details of the analysis are given in the legend to Figure S4. The accession number (gi) or the *T. hominis* ORF locus tag are given for each sequence in the tree.
(PDF)

Figure S8 Gain and loss of Pfam domains during microsporidian evolution. Gain and loss of Pfam domains were plotted onto the cladogram using Dollo parsimony. The numbers indicated on the branches show the number of Pfam domains inferred to have been lost (red) or gained (green).
(PDF)

Figure S9 Synonymous codon usage in *S. cerevisiae* and the five sequenced microsporidian genomes. Per-gene codon usage was quantified using the F_OP_ score (frequency of optimal codons) [39], which measures for each gene the proportion of amino acids encoded by the “optimal” (most frequent) codon, determined for the respective amino acid over the whole genome. Choice of synonymous codon usage is characteristic of a genome, often resulting in a unimodal distribution of the F_OP_ score. The biased distribution for *N. ceranae* towards higher F_OP_ scores reflects the reduction of codon usage variation in this genome as a function of very low G+C content (27.2%, vs. 40.3%+/−5.5% standard deviation for the other species [133]). The broad bimodal shape of the F_OP_ distribution for *E. bieneusi* suggests heterogeneity of codon usage for which one possible explanation is contamination of the data (see main text for discussion).
(PDF)

Figure S10 Phylogeny of TriC/CCT (chaperonin) genes in the microsporidia. All of the microsporidians have retained each of the eight TriC/CCT (cytosolic chaperonin) subunits. Support values are given as Bayesian posterior probabilities. The phylogeny was built using the CAT20 model in PhyloBayes.
(PDF)

Figure S11 The number and types of different transport proteins in *T. hominis* and the other microsporidians investigated. The figure shows the different transporter protein families and the number of individual proteins in each family (MCL cluster) for each microsporidian genome investigated. The key for the abbreviated transporter names is provided in Table S14, where details of the manual annotation are also described. The number of proteins within each cluster for each species is indicated by a colour ranging from 1 protein (blue) to 8 proteins (red). An empty box indicates that no family member was detected.
(PDF)

Figure S12 Phylogenetic analysis of microsporidian MFS transporters. Each species has retained at least one member of each MFS subgroup, suggesting that MFS transporters are functionally important for microsporidians. The tree was calculated with p4 on a Dayhoff-recoded dataset using two base composition vectors and were calculated for 2 million generations as described in Figure S4, support values are given as Bayesian posterior probabilities. Putative substrates are indicated; details of the evidence supporting these inferences are given in Table S14.
(PDF)

Figure S13 Phylogenetic analysis of microsporidian ABC transporters. To increase the number of positions available for phylogenetic analyses, the transporters were split into two groups based on their different domain organisations (A) N-terminus - TMD - nucleotide binding domain (NBD) - C-terminus and (B) N-terminus - NBD - TMD - C-terminus. These trees reveal lineage-specific duplications among microsporidians, with an apparent expansion of the transporters with topology (A) in *T. hominis*. The trees were calculated with p4 on a Dayhoff-recoded dataset using 7 base composition vectors and were calculated for 2 million generations as described in Figure S4, support values are given as Bayesian posterior probabilities. Details on similarities to other ABC transporters as well as their potential functions are given in Table S14.
(PDF)

Figure S14 An endomembrane metalloprotease (CAAX-like peptidase) of the M79 family in *T. hominis* was potentially acquired by lateral gene transfer. (A) Protein alignment of the potential LGT orf_3051 with its closest BLAST hits. The three functional motifs for M79 peptidases are highlighted in green. Further details for the sequences displayed are given in Table S17. (B) The predicted transmembrane profile obtained using TMHMM [96] for the *T. hominis* protein is much more similar to the *Staphylococcus hominis* protein than to the eukaryotic sequence from *Arabidopsis thaliana* (Table S17).
(PDF)

Figure S15 Phylogenies of the triosephosphate transporter (TPT) and the RNA helicase (Hel) genes that form part of the syntenic sex locus in Zygomycetes. Zygomycete sex locus sequences in red; microsporidian sequences in blue. The tree topologies for TPT (A) and Hel (B) suggest that the observed synteny between these sequences in zygomycete fungi, *E. cuniculi* and *E. bienieusi* is due to convergence. In the case of both genes, the zygomycete and microsporidian sequences are paralogs, related via a gene duplication that occurred early in eukaryotic evolution, confirming the analyses of Koestler and Ebersberger [74]. Sequences on either side of these duplications are denoted by (1) and (2). The phylogenies are consistent with the observation that these genes are not syntenic in *T. hominis*. The sequences are those used in Koestler and Ebersberger [74], with the addition of the respective *T. hominis* sequences.
(PDF)

Figure S16 The same Voronoi treemap shown in Figure 7 showing the corresponding locus tags and protein annotation of the individual proteins identified. The area of individual cells is proportional to the semi-quantitative spectral counts of each individual protein.
(TIF)

Table S1 An overview of the most complete microsporidian genomes sequenced to date including the sequencing techniques as well as key features of the genomes based on the original papers and our own analyses.
(XLS)

Table S2 List of *T. hominis* ORFs that potentially contain introns.
(XLS)

Table S3 Intron conservation in *T. hominis* and *N. ceranae* orthologues of experimentally confirmed *E. cuniculi* intron-containing genes.
(XLS)

Table S4 Putative regulatory motifs in *T. hominis*. The table includes the number of ORFs located downstream of each motif and the annotation for these ORFs.
(XLS)

Table S5 Putative regulatory motifs detected by MEME in the intergenic regions of *T. hominis*, provided as regular expressions, and putative transcription factors for some motifs (Figure 2).
(XLS)

Table S6 (A) Transposon-encoded open reading frames in the *T. hominis* genome. (B) A comparison of mobile genetic elements identified by our searches in the *T. hominis* and the *N. ceranae* genomes.
(XLS)

Table S7 Analysis of the 74 ORFs containing the 69 Pfam domains inferred as having been gained by *E. bieneusi* based on Dollo parsimony analysis (Figure S8). Many of the 74 ORF appear to be most similar to bacterial sequences and may be contaminants (see main text for discussion).
(XLS)

Table S8 Analysis of the 320 protein families inferred using Dollo parsimony to have been gained in the common ancestor of the Microsporidia (Figure 4).
(XLS)

Table S9 Details of the 93 protein families that may be unique to microsporidia including representation in the spore proteomics data.
(XLS)

Table S10 Details of the 371 ORF in the 88 *T. hominis*-specific gene families.
(XLS)

Table S11 Presence and absence of enzymes in selected metabolic pathways across the Microsporidia. The table is an expanded version of Table S1 in Keeling *et al*. [55].
(XLS)

Table S12 This table lists the manually curated Enzyme Commission (EC) annotation for *T. hominis* proteins, as well as for the manually curated KEGG maps represented in Figure S1.
(XLS)

Table S13 Analysis of the chaperone repertoire of microsporidia. Microsporidia have lost a number of Hsp70 and Hsp40 family members, but have retained the subunits of the eukaryote cytosolic TriC/CCT complex.
(XLS)

Table S14 Putative *T. hominis* and microsporidian transport proteins identified using a variety of approaches and with detailed annotation.
(XLS)

Table S15 Comparison of the transport proteins found in *T. hominis* and *S. cerevisiae*.
(XLS)

Table S16 Putative proteases/peptidases found in *T. hominis*.
(XLS)

Table S17 Sequence similarity searches of orf_3051 against the NCBI nr database (see Figure S14). The *A. fumigatus* sequence lacks the functional M79 family (CAAX-like) peptidase sites and is therefore not included in the alignment figure (Figure S14).
(XLS)

Table S18 The unusual taxonomic distribution of the asnA gene in eukaryotes based on BLASTP searches against the NCBI nr database.
(XLS)

Table S19 The 484 *T. hominis* proteins detected by proteomics of purified spores. Locus tags, annotations and selected structural details including inferred SP and TMD, along with spectral counts and corresponding mean value (n = 2)(semi-quantitative values) for each protein are provided. Details on the settings for the measurements and the database search are described in the Materials and Methods section and the complete MS data are available from the Tranche repository database (see Materials and Methods).
(XLS)

Table S20 Primers used in this study for screening DNA preparations.
(XLS)
