# Supplementary material for: The Genome of the Obligate Intracellular Parasite Trachipleistophora hominis: New Insights into Microsporidian Genome Dynamics and Reductive Evolution
Source: PLoS Pathog. 2012 Oct 25;8(10):e1002979. doi: 10.1371/journal.ppat.1002979 (PMC3486916; doi:10.1371/journal.ppat.1002979)
Supplement: Figure S1 — KEGG maps for Trachipleistophora hominis. The KEGG maps were generated based on the curated annotation of the T. hominis genome. The results from enzyme annotation, profile-profile search and pairwise sequence similarity searches were used to annotate putative genes on the KEGG maps. Yellow boxes indicate the presence of a respective T. hominis protein; red letters indicate manually annotated EC numbers, blue letters indicate proteins assigned by BLAST searches (cut off ≤1E-05) against all proteins in the KEGG database, and violet letters indicate manual annotation following the HHSearch analyses of the clusters inferred to be present in the common microsporidian ancestor. (PDF) [file ppat.1002979.s001.pdf]

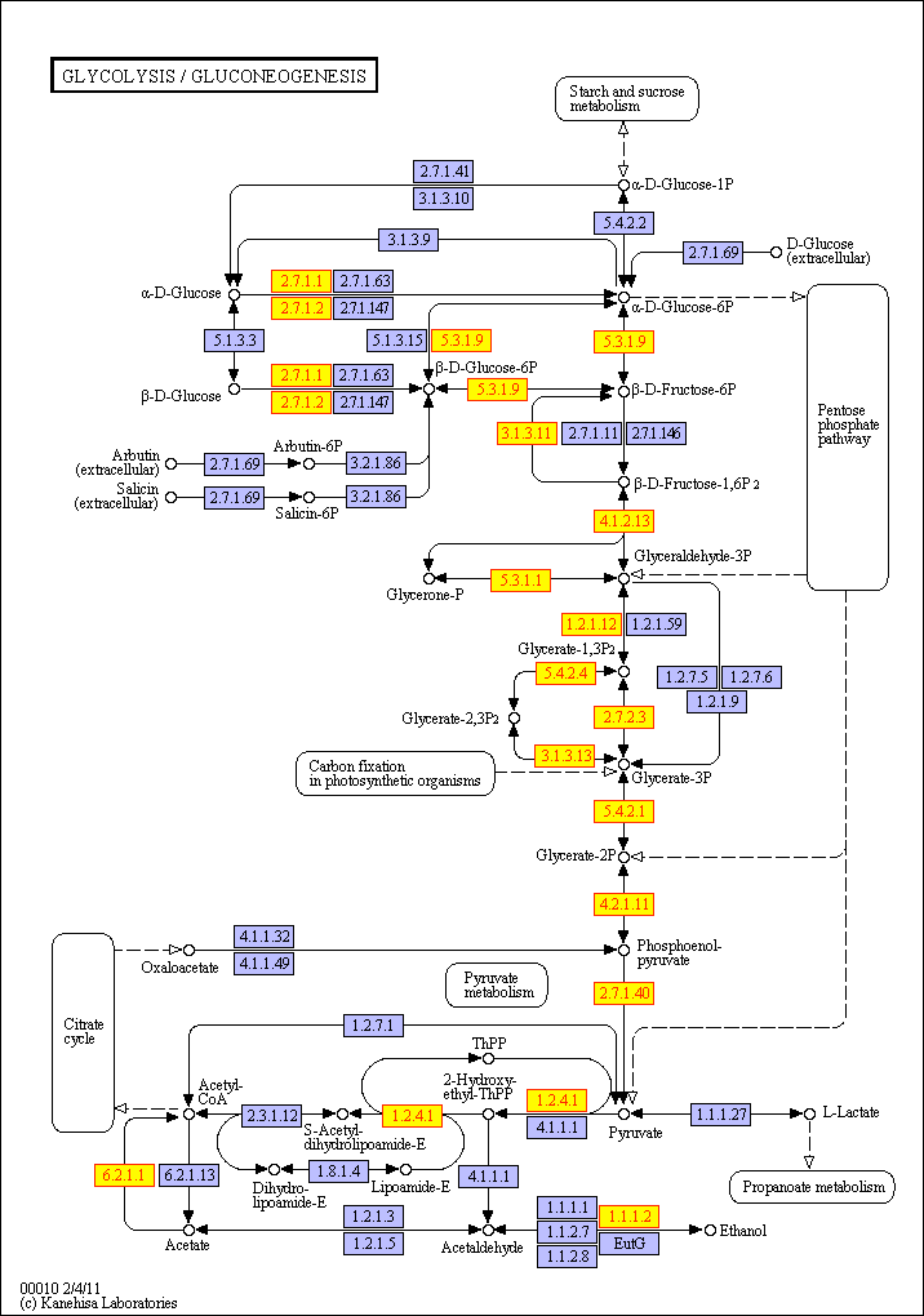

GLYCOLYSIS / GLUCONEOGENESIS
Starch and sucrose metabolism
2.7.1.41
3.1.3.10
α-D-Glucose-1P
5.4.2.2
3.1.3.9
2.7.1.69
D-Glucose (extracellular)
α-D-Glucose
2.7.1.1
2.7.1.63
2.7.1.2
2.7.1.147
α-D-Glucose-6P
5.1.3.3
5.1.3.15
5.3.1.9
β-D-Glucose-6P
β-D-Glucose
β-D-Fructose-6P
3.1.3.11
2.7.1.11
2.7.1.146
Arbutin (extracellular)
Arbutin-6P
2.7.1.69
3.2.1.86
Salicin (extracellular)
Salicin-6P
β-D-Fructose-1,6P2
Pentose phosphate pathway
4.1.2.13
Glyceraldehyde-3P
5.3.1.1
Glycerone-P
1.2.1.12
1.2.1.59
Glycerate-1,3P2
5.4.2.4
1.2.7.5
1.2.7.6
1.2.1.9
Glycerate-2,3P2
2.7.2.3
3.1.3.13
Carbon fixation in photosynthetic organisms
Glycerate-3P
5.4.2.1
Glycerate-2P
4.2.1.11
4.1.1.32
4.1.1.49
Oxaloacetate
Phosphoenol-pyruvate
Citrate cycle
Pyruvate metabolism
2.7.1.40
1.2.7.1
ThPP
2-Hydroxy-ethyl-ThPP
Acetyl-CoA
2.3.1.12
S-Acetyl-dihydrolipoamide-E
1.2.4.1
4.1.1.1
Pyruvate
1.1.1.27
L-Lactate
6.2.1.1
6.2.1.13
Dihydro-lipoamide-E
1.8.1.4
Lipoamide-E
Propanoate metabolism
1.2.1.3
1.2.1.5
1.1.1.1
1.1.1.2
1.1.2.7
EutG
1.1.2.8
Ethanol
Acetate
Acetaldehyde
00010 2/4/11
(c) Kanehisa Laboratories

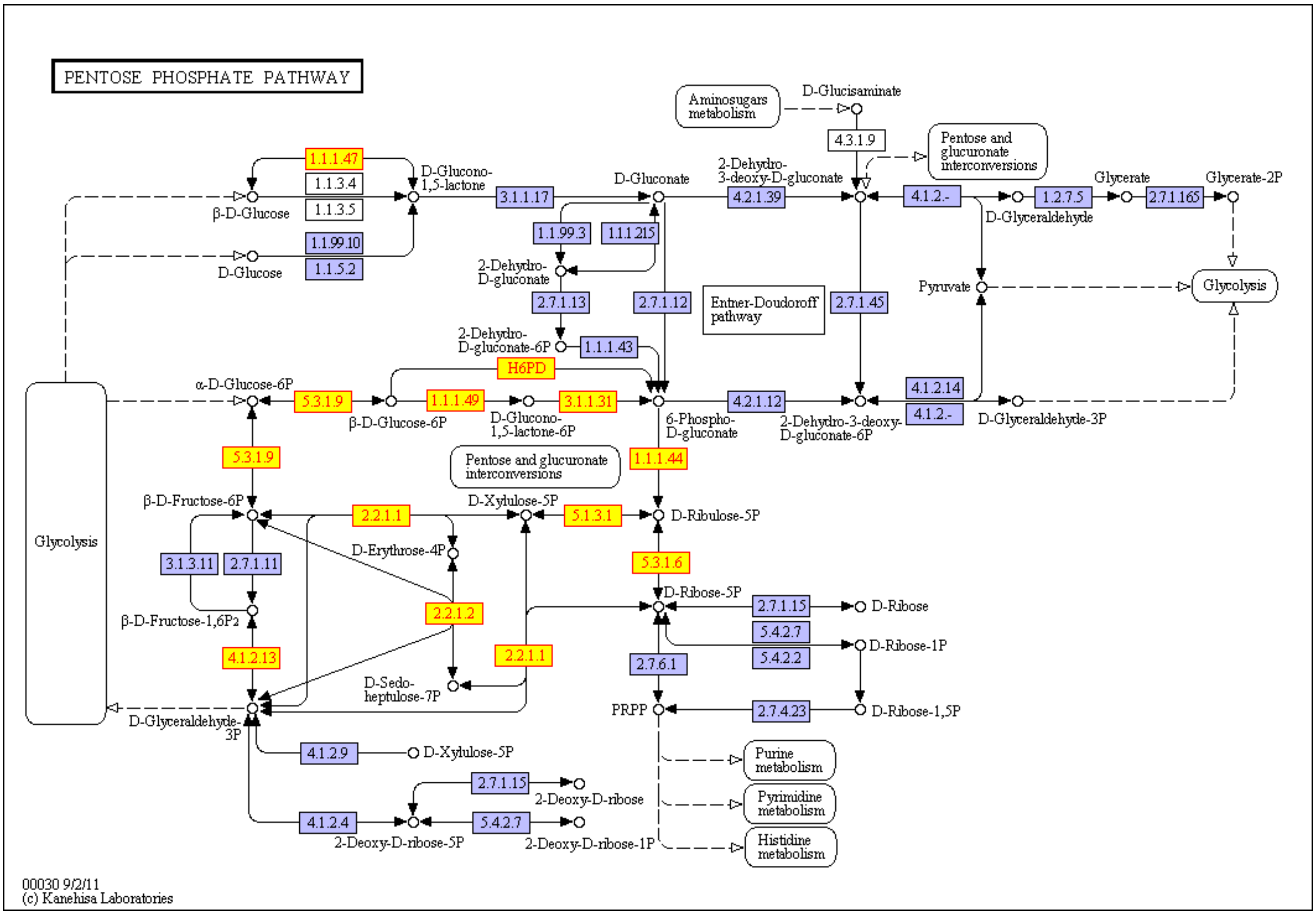
PENTOSE PHOSPHATE PATHWAY
Aminosugars metabolism
D-Glucisaminate
4.3.1.9
Pentose and glucuronate interconversions
1.1.1.47
1.1.3.4
1.1.3.5
1.1.99.10
1.1.5.2
β-D-Glucose
D-Glucose
D-Glucono-1,5-lactone
3.1.1.17
D-Gluconate
2-Dehydro-3-deoxy-D-gluconate
4.2.1.39
4.1.2.-
Glycerate
Glycerate-2P
1.2.7.5
2.7.1.165
D-Glyceraldehyde
1.1.99.3
1.1.1.215
2-Dehydro-D-gluconate
2.7.1.13
2.7.1.12
Entner-Doudoroff pathway
2.7.1.45
Pyruvate
Glycolysis
2-Dehydro-D-gluconate-6P
1.1.1.43
H6PD
α-D-Glucose-6P
5.3.1.9
β-D-Glucose-6P
1.1.1.49
D-Glucono-1,5-lactone-6P
3.1.1.31
6-Phospho-D-gluconate
4.2.1.12
2-Dehydro-3-deoxy-D-gluconate-6P
4.1.2.14
4.1.2.-
D-Glyceraldehyde-3P
Glycolysis
5.3.1.9
Pentose and glucuronate interconversions
1.1.1.44
β-D-Fructose-6P
2.2.1.1
D-Xylulose-5P
5.1.3.1
D-Ribulose-5P
D-Erythrose-4P
5.3.1.6
3.1.3.11
2.7.1.11
β-D-Fructose-1,6P2
2.2.1.2
D-Ribose-5P
2.7.1.15
D-Ribose
5.4.2.7
5.4.2.2
D-Ribose-1P
4.1.2.13
2.2.1.1
2.7.6.1
D-Sedo-heptulose-7P
PRPP
2.7.4.23
D-Ribose-1,5P
D-Glyceraldehyde-3P
4.1.2.9
D-Xylulose-5P
Purine metabolism
2.7.1.15
2-Deoxy-D-ribose
Pyrimidine metabolism
4.1.2.4
5.4.2.7
2-Deoxy-D-ribose-5P
2-Deoxy-D-ribose-1P
Histidine metabolism
00030 9/2/11
(c) Kanehisa Laboratories

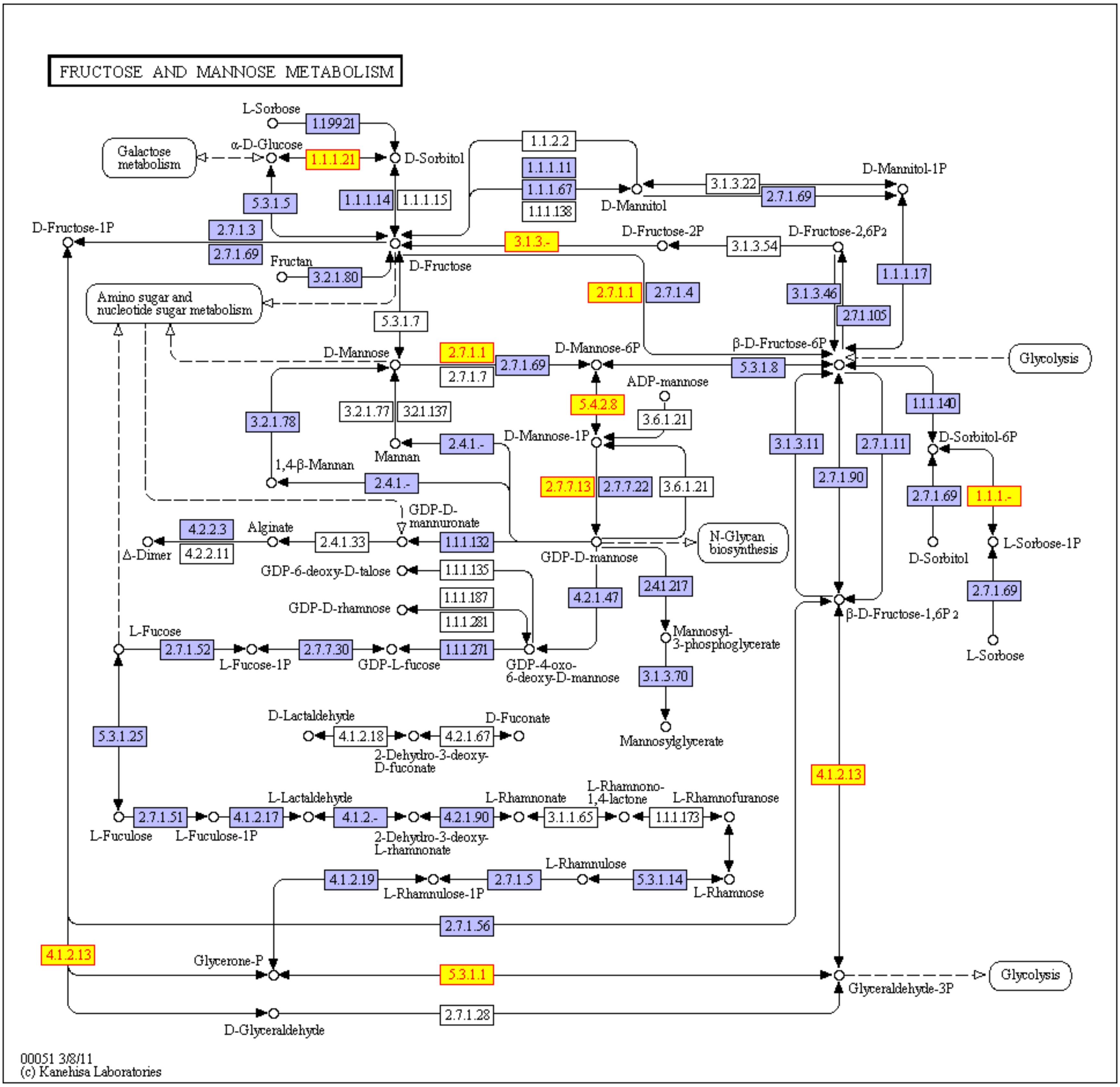
FRUCTOSE AND MANNOSE METABOLISM
L-Sorbose
1.1.99.21
α-D-Glucose
D-Sorbitol
Galactose metabolism
1.1.1.21
1.1.2.2
1.1.1.11
1.1.1.67
1.1.1.138
1.1.1.14
1.1.1.15
5.3.1.5
D-Mannitol
3.1.3.22
2.7.1.69
D-Mannitol-1P
D-Fructose-1P
2.7.1.3
2.7.1.69
3.1.3.-
D-Fructose-2P
3.1.3.54
D-Fructose-2,6P2
Fructan
3.2.1.80
D-Fructose
2.7.1.1
2.7.1.4
3.1.3.46
1.1.1.17
2.7.1.105
Amino sugar and nucleotide sugar metabolism
5.3.1.7
D-Mannose
2.7.1.1
2.7.1.7
2.7.1.69
D-Mannose-6P
5.3.1.8
β-D-Fructose-6P
Glycolysis
3.2.1.78
3.2.1.77
3.2.1.137
5.4.2.8
ADP-mannose
3.6.1.21
1.1.1.140
Mannan
2.4.1.-
D-Mannose-1P
3.1.3.11
2.7.1.11
D-Sorbitol-6P
1,4-β-Mannan
2.4.1.-
2.7.7.13
2.7.7.22
3.6.1.21
2.7.1.90
2.7.1.69
1.1.1.-
GDP-D-mannuronate
4.2.2.3
Alginate
Δ-Dimer
4.2.2.11
2.4.1.33
1.1.1.132
N-Glycan biosynthesis
GDP-D-mannose
D-Sorbitol
L-Sorbose-1P
GDP-6-deoxy-D-talose
1.1.1.135
2.4.1.217
4.2.1.47
1.1.1.187
1.1.1.281
2.7.1.69
GDP-D-rhamnose
β-D-Fructose-1,6P2
L-Fucose
Mannosyl-3-phosphoglycerate
2.7.1.52
2.7.7.30
1.1.1.271
L-Fucose-1P
GDP-L-fucose
GDP-4-oxo-6-deoxy-D-mannose
L-Sorbose
3.1.3.70
5.3.1.25
D-Lactaldehyde
D-Fuconate
4.1.2.18
4.2.1.67
Mannosylglycerate
2-Dehydro-3-deoxy-D-fuconate
4.1.2.13
L-Lactaldehyde
L-Rhamnonate
L-Rhamnono-1,4-lactone
L-Rhamnofuranose
2.7.1.51
4.1.2.17
4.1.2.-
4.2.1.90
3.1.1.65
1.1.1.173
L-Fuculose
L-Fuculose-1P
2-Dehydro-3-deoxy-L-rhamnonate
L-Rhamnulose
4.1.2.19
2.7.1.5
5.3.1.14
L-Rhamnulose-1P
L-Rhamnose
2.7.1.56
4.1.2.13
Glycerone-P
5.3.1.1
Glyceraldehyde-3P
Glycolysis
2.7.1.28
D-Glyceraldehyde
00051 3/8/11
(c) Kanehisa Laboratories

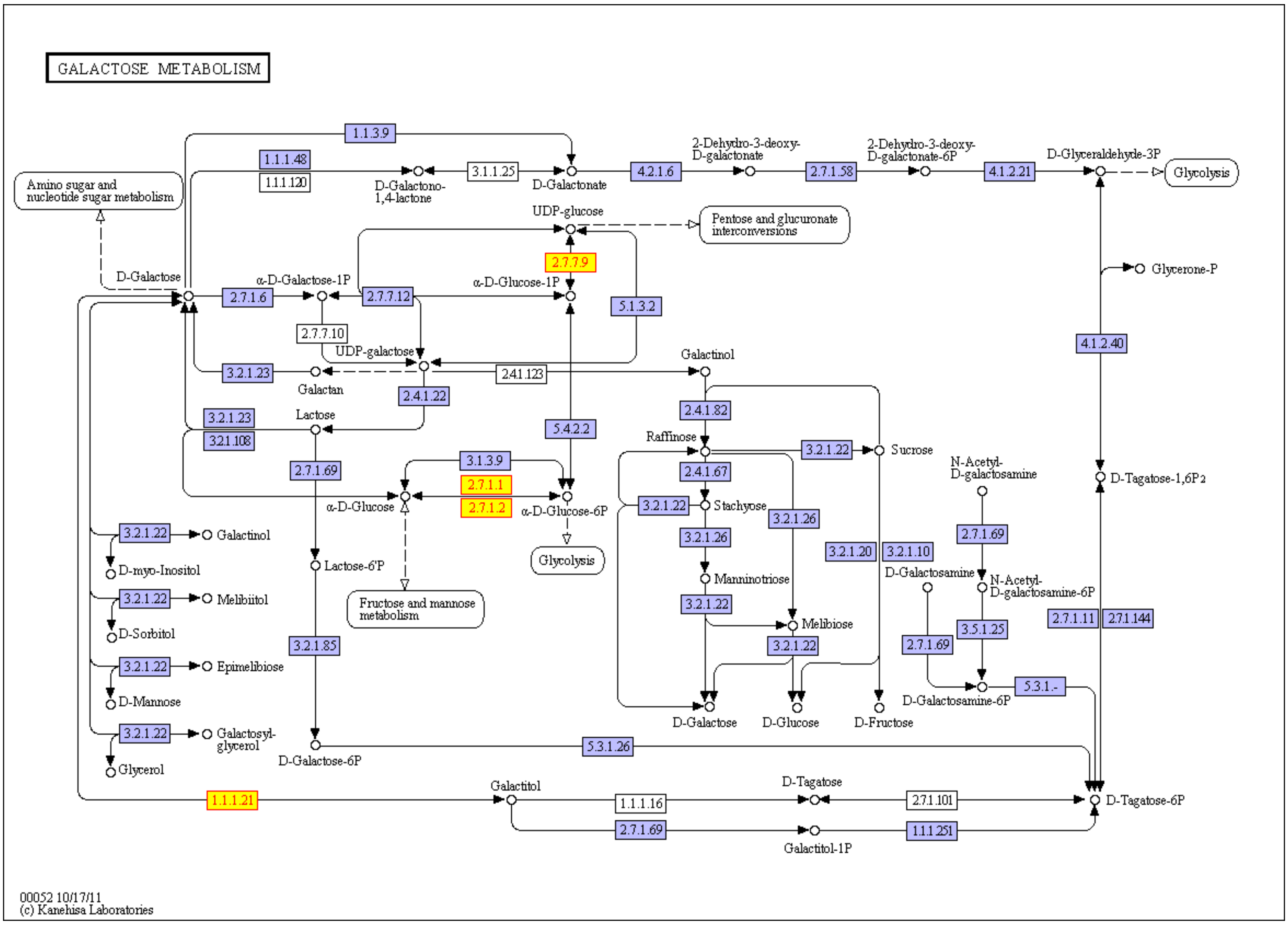
GALACTOSE METABOLISM
1.1.3.9
1.1.1.48
1.1.1.120
3.1.1.25
D-Galactono-1,4-lactone
D-Galactonate
4.2.1.6
2-Dehydro-3-deoxy-D-galactonate
2.7.1.58
2-Dehydro-3-deoxy-D-galactonate-6P
4.1.2.21
D-Glyceraldehyde-3P
Glycolysis
Amino sugar and nucleotide sugar metabolism
UDP-glucose
Pentose and glucuronate interconversions
2.7.7.9
D-Galactose
α-D-Galactose-1P
2.7.1.6
2.7.7.12
α-D-Glucose-1P
5.1.3.2
Glycerone-P
2.7.7.10
UDP-galactose
3.2.1.23
Galactan
2.4.1.123
Galactinol
4.1.2.40
2.4.1.22
5.4.2.2
2.4.1.82
Lactose
3.2.1.23
3.2.1.108
Raffinose
3.2.1.22
Sucrose
3.1.3.9
2.7.1.69
2.7.1.1
2.7.1.2
α-D-Glucose
α-D-Glucose-6P
2.4.1.67
3.2.1.22
Stachyose
3.2.1.26
N-Acetyl-D-galactosamine
D-Tagatose-1,6P2
3.2.1.22
Galactinol
D-myo-Inositol
Lactose-6'P
Glycolysis
3.2.1.26
3.2.1.20
3.2.1.10
2.7.1.69
D-Galactosamine
N-Acetyl-D-galactosamine-6P
3.2.1.22
Melibiitol
D-Sorbitol
Fructose and mannose metabolism
Manninotriose
3.2.1.22
Melibiose
3.5.1.25
2.7.1.11
2.7.1.144
3.2.1.22
Epimelibiose
D-Mannose
3.2.1.85
3.2.1.22
2.7.1.69
5.3.1.-
D-Galactosamine-6P
D-Galactose
D-Glucose
D-Fructose
3.2.1.22
Galactosyl-glycerol
Glycerol
D-Galactose-6P
5.3.1.26
Galactitol
D-Tagatose
1.1.1.21
1.1.1.16
2.7.1.101
D-Tagatose-6P
2.7.1.69
1.1.1.251
Galactitol-1P
00052 10/17/11
(c) Kanehisa Laboratories

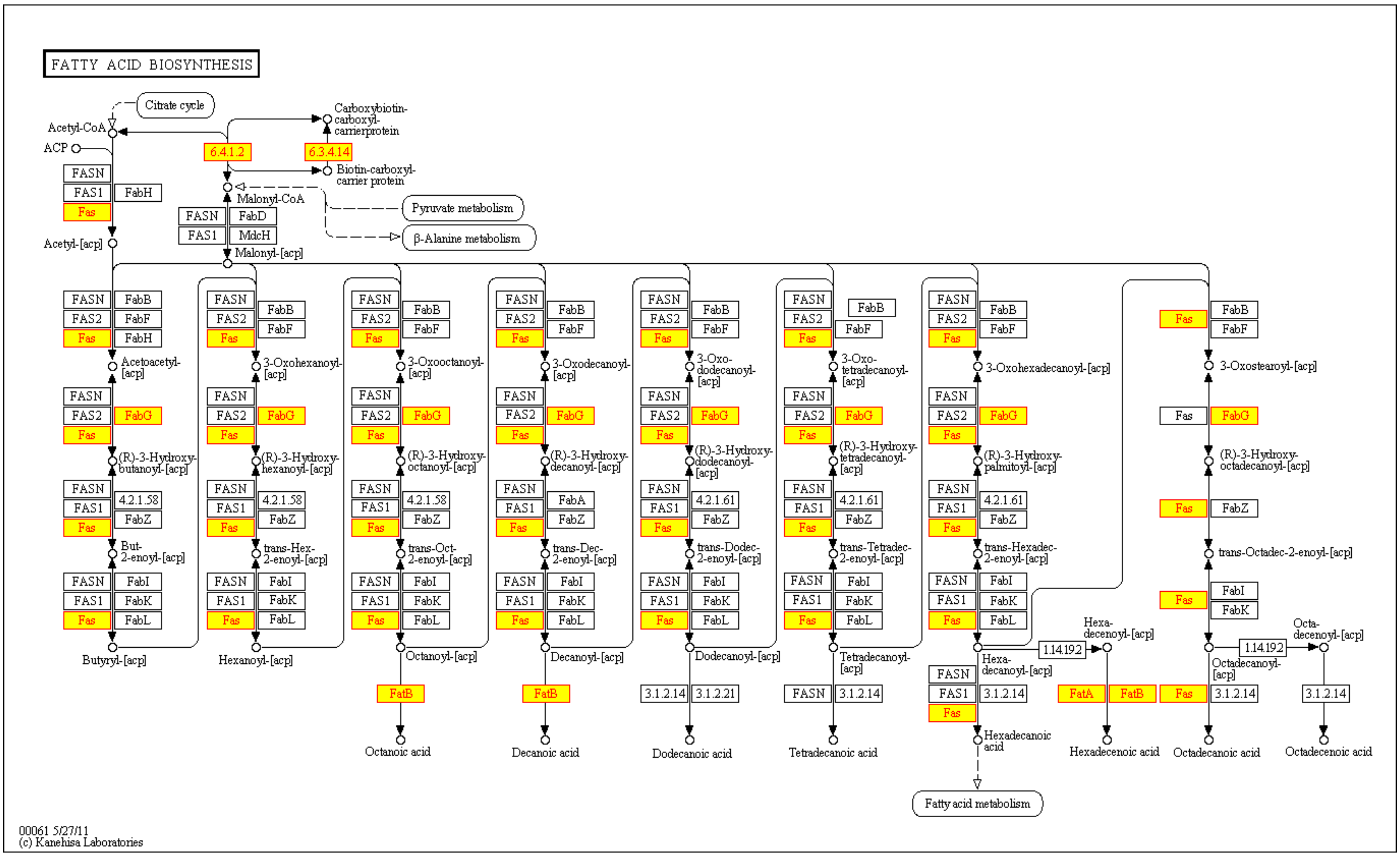
FATTY ACID BIOSYNTHESIS
Citrate cycle
Acetyl-CoA
ACP
Carboxybiotin-carboxyl-carrierprotein
Biotin-carboxyl-carrier protein
6.4.1.2
6.3.4.14
Malonyl-CoA
Pyruvate metabolism
β-Alanine metabolism
FASN
FAS1
FAS2
Fas
FabH
FabD
MdcH
Acetyl-[acp]
Malonyl-[acp]
FabB
FabF
FabG
FabZ
FabA
FabI
FabK
FabL
FatA
FatB
4.2.1.58
4.2.1.61
3.1.2.14
3.1.2.21
1.14.19.2
Acetoacetyl-[acp]
(R)-3-Hydroxy-butanoyl-[acp]
But-2-enoyl-[acp]
Butyryl-[acp]
3-Oxohexanoyl-[acp]
(R)-3-Hydroxy-hexanoyl-[acp]
trans-Hex-2-enoyl-[acp]
Hexanoyl-[acp]
3-Oxooctanoyl-[acp]
(R)-3-Hydroxy-octanoyl-[acp]
trans-Oct-2-enoyl-[acp]
Octanoyl-[acp]
Octanoic acid
3-Oxodecanoyl-[acp]
(R)-3-Hydroxy-decanoyl-[acp]
trans-Dec-2-enoyl-[acp]
Decanoyl-[acp]
Decanoic acid
3-Oxo-dodecanoyl-[acp]
(R)-3-Hydroxy-dodecanoyl-[acp]
trans-Dodec-2-enoyl-[acp]
Dodecanoyl-[acp]
Dodecanoic acid
3-Oxo-tetradecanoyl-[acp]
(R)-3-Hydroxy-tetradecanoyl-[acp]
trans-Tetradec-2-enoyl-[acp]
Tetradecanoyl-[acp]
Tetradecanoic acid
3-Oxohexadecanoyl-[acp]
(R)-3-Hydroxy-palmitoyl-[acp]
trans-Hexadec-2-enoyl-[acp]
Hexa-decanoyl-[acp]
Hexadecanoic acid
Hexa-decenoyl-[acp]
Hexadecenoic acid
Fatty acid metabolism
3-Oxostearoyl-[acp]
(R)-3-Hydroxy-octadecanoyl-[acp]
trans-Octadec-2-enoyl-[acp]
Octadecanoyl-[acp]
Octadecanoic acid
Octa-decenoyl-[acp]
Octadecenoic acid
00061 5/27/11
(c) Kanehisa Laboratories

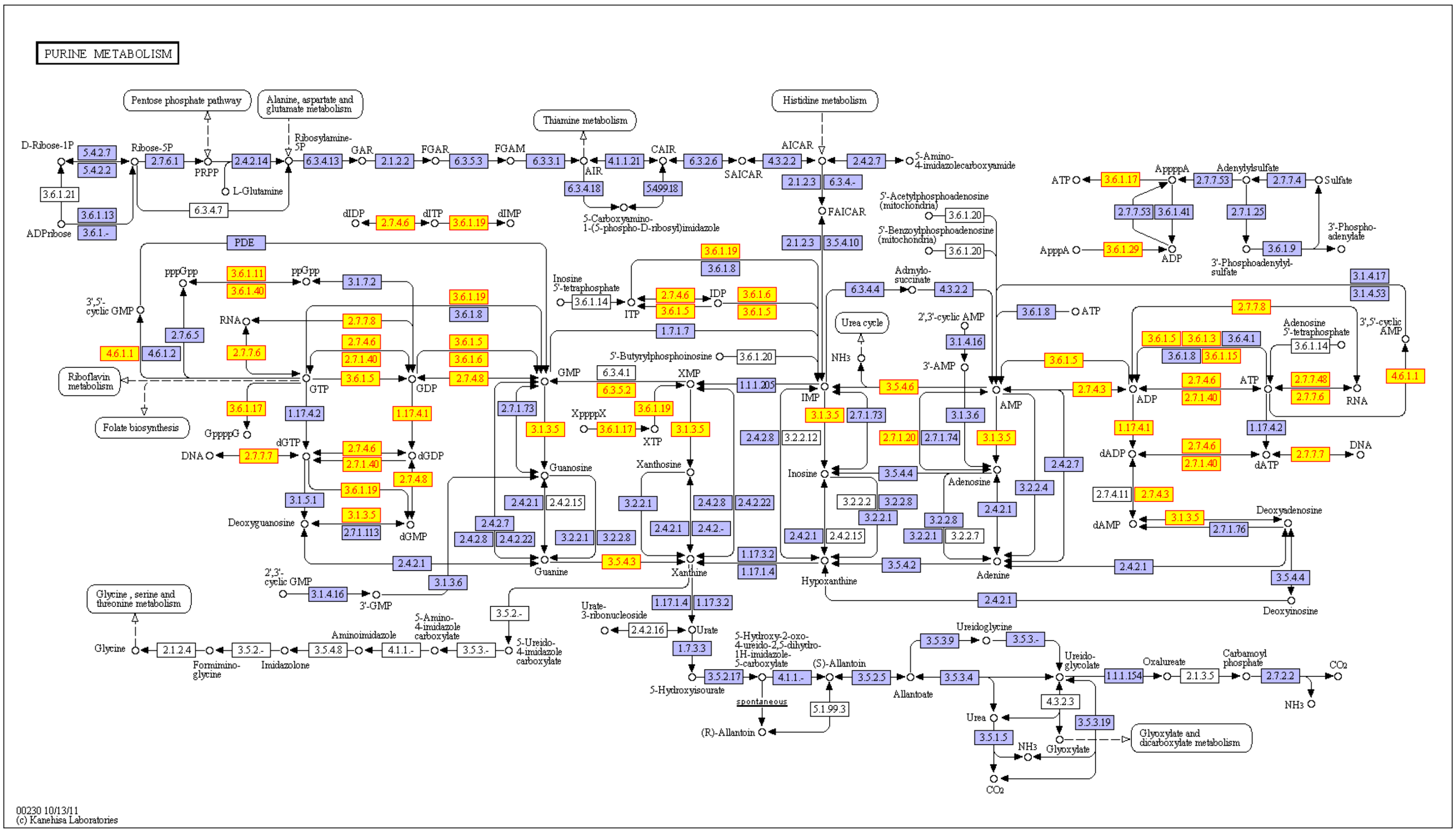
PURINE METABOLISM
Pentose phosphate pathway
Alanine, aspartate and glutamate metabolism
Thiamine metabolism
Histidine metabolism
D-Ribose-1P
5.4.2.7
5.4.2.2
3.6.1.21
3.6.1.13
3.6.1.-
ADPribose
Ribose-5P
2.7.6.1
PRPP
6.3.4.7
2.4.2.14
L-Glutamine
Ribosylamine-5P
6.3.4.13
GAR
2.1.2.2
FGAR
6.3.5.3
FGAM
6.3.3.1
AIR
6.3.4.18
4.1.1.21
CAIR
5.4.99.18
5-Carboxyamino-1-(5-phospho-D-ribosyl)imidazole
6.3.2.6
SAICAR
4.3.2.2
AICAR
2.1.2.3
6.3.4.-
2.4.2.7
5-Amino-4-imidazolecarboxyamide
FAICAR
2.1.2.3
3.5.4.10
dIDP
2.7.4.6
dITP
3.6.1.19
dIMP
PDE
3',5'-cyclic GMP
pppGpp
3.6.1.11
3.6.1.40
ppGpp
3.1.7.2
2.7.6.5
RNA
2.7.7.8
2.7.4.6
2.7.1.40
2.7.7.6
4.6.1.1
4.6.1.2
Riboflavin metabolism
Folate biosynthesis
GTP
3.6.1.5
GDP
3.6.1.17
GppppG
1.17.4.2
1.17.4.1
DNA
2.7.7.7
dGTP
2.7.4.6
2.7.1.40
dGDP
3.6.1.19
2.7.4.8
3.1.5.1
Deoxyguanosine
3.1.3.5
2.7.1.113
dGMP
2.4.2.1
2',3'-cyclic GMP
3.1.4.16
3'-GMP
3.1.3.6
Glycine, serine and threonine metabolism
Glycine
2.1.2.4
Formimino-glycine
3.5.2.-
Imidazolone
3.5.4.8
Aminoimidazole
4.1.1.-
5-Amino-4-imidazole carboxylate
3.5.3.-
5-Ureido-4-imidazole carboxylate
3.5.2.-
3.6.1.19
3.6.1.8
3.6.1.5
3.6.1.6
Inosine 5'-tetraphosphate
3.6.1.14
ITP
2.7.4.6
IDP
3.6.1.6
3.6.1.5
1.7.1.7
5'-Butyrylphosphoinosine
3.6.1.20
GMP
6.3.4.1
6.3.5.2
XMP
1.1.1.205
2.7.1.73
XppppX
3.6.1.17
XTP
3.6.1.19
3.1.3.5
3.1.3.5
Guanosine
Xanthosine
2.4.2.1
2.4.2.15
3.2.2.1
2.4.2.8
2.4.2.22
2.4.2.7
2.4.2.1
2.4.2.-
2.4.2.8
2.4.2.22
3.2.2.1
3.2.2.8
Guanine
3.5.4.3
Xanthine
1.17.3.2
1.17.1.4
1.17.1.4
1.17.3.2
Urate-3-ribonucleoside
2.4.2.16
Urate
1.7.3.3
5-Hydroxy-2-oxo-4-ureido-2,5-dihydro-1H-imidazole-5-carboxylate
5-Hydroxyisourate
3.5.2.17
4.1.1.-
(S)-Allantoin
spontaneous
5.1.99.3
(R)-Allantoin
3.5.2.5
Allantoate
3.5.3.4
3.5.3.9
Ureidoglycine
3.5.3.-
Ureido-glycolate
4.3.2.3
Urea
3.5.1.5
NH3
CO2
Glyoxylate
3.5.3.19
Glyoxylate and dicarboxylate metabolism
1.1.1.154
Oxalureate
2.1.3.5
Carbamoyl phosphate
2.7.2.2
CO2
NH3
IMP
3.1.3.5
2.7.1.73
2.4.2.8
3.2.2.12
Inosine
3.2.2.2
3.2.2.8
3.2.2.1
2.4.2.1
2.4.2.15
Hypoxanthine
3.5.4.2
3.5.4.4
3.5.4.6
NH3
Urea cycle
6.3.4.4
Adenylo-succinate
4.3.2.2
5'-Acetylphosphoadenosine (mitochondria)
3.6.1.20
5'-Benzoylphosphoadenosine (mitochondria)
3.6.1.20
2',3'-cyclic AMP
3.1.4.16
3'-AMP
3.1.3.6
AMP
3.6.1.8
ATP
3.6.1.5
2.7.1.20
2.7.1.74
3.1.3.5
Adenosine
3.2.2.4
2.4.2.7
2.4.2.1
3.2.2.8
3.2.2.1
3.2.2.7
Adenine
2.4.2.1
2.4.2.1
ATP
3.6.1.17
ApppA
2.7.7.53
2.7.7.53
3.6.1.41
ADP
Adenylylsulfate
2.7.7.4
Sulfate
2.7.1.25
ApppA
3.6.1.29
3'-Phosphoadenylyl-sulfate
3.6.1.9
3'-Phospho-adenylate
3.1.4.17
3.1.4.53
2.7.7.8
3.6.1.5
3.6.1.3
3.6.4.1
3.6.1.8
3.6.1.15
Adenosine 5'-tetraphosphate
3.6.1.14
3',5'-cyclic AMP
ADP
2.7.4.3
2.7.4.6
2.7.1.40
ATP
2.7.7.48
2.7.7.6
RNA
4.6.1.1
1.17.4.1
1.17.4.2
dADP
2.7.4.6
2.7.1.40
dATP
2.7.7.7
DNA
2.7.4.11
2.7.4.3
dAMP
3.1.3.5
2.7.1.76
Deoxyadenosine
3.5.4.4
2.4.2.1
Deoxyinosine
00230 10/13/11
(c) Kanehisa Laboratories

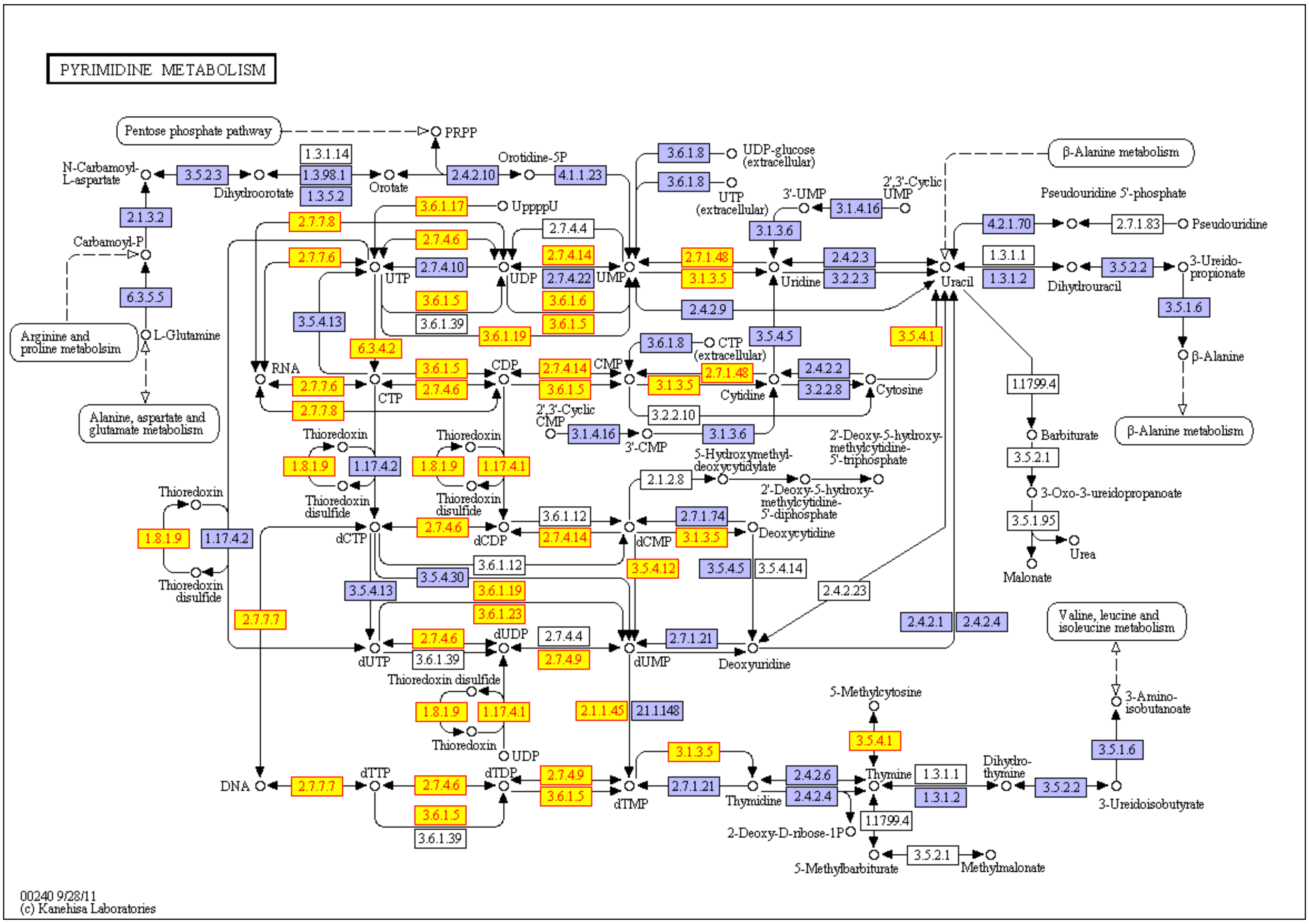
PYRIMIDINE METABOLISM
Pentose phosphate pathway
PRPP
N-Carbamoyl-L-aspartate
Dihydroorotate
Orotate
Orotidine-5P
UDP-glucose (extracellular)
UTP (extracellular)
3'-UMP
2',3'-Cyclic UMP
β-Alanine metabolism
Pseudouridine 5'-phosphate
Pseudouridine
Carbamoyl-P
UppppU
UTP
UDP
UMP
Uridine
Uracil
Dihydrouracil
3-Ureido-propionate
β-Alanine
L-Glutamine
Arginine and proline metabolsim
Alanine, aspartate and glutamate metabolism
RNA
CTP
CDP
CMP
Cytidine
Cytosine
CTP (extracellular)
2',3'-Cyclic CMP
3'-CMP
Barbiturate
3-Oxo-3-ureidopropanoate
Urea
Malonate
Thioredoxin
Thioredoxin disulfide
5-Hydroxymethyl-deoxycytidylate
2'-Deoxy-5-hydroxy-methylcytidine-5'-triphosphate
2'-Deoxy-5-hydroxy-methylcytidine-5'-diphosphate
Deoxycytidine
dCTP
dCDP
dCMP
dUTP
dUDP
dUMP
Deoxyuridine
Valine, leucine and isoleucine metabolism
5-Methylcytosine
3-Amino-isobutanoate
DNA
dTTP
dTDP
dTMP
Thymidine
Thymine
Dihydro-thymine
3-Ureidoisobutyrate
2-Deoxy-D-ribose-1P
5-Methylbarbiturate
Methylmalonate
UDP
00240 9/28/11
(c) Kanehisa Laboratories

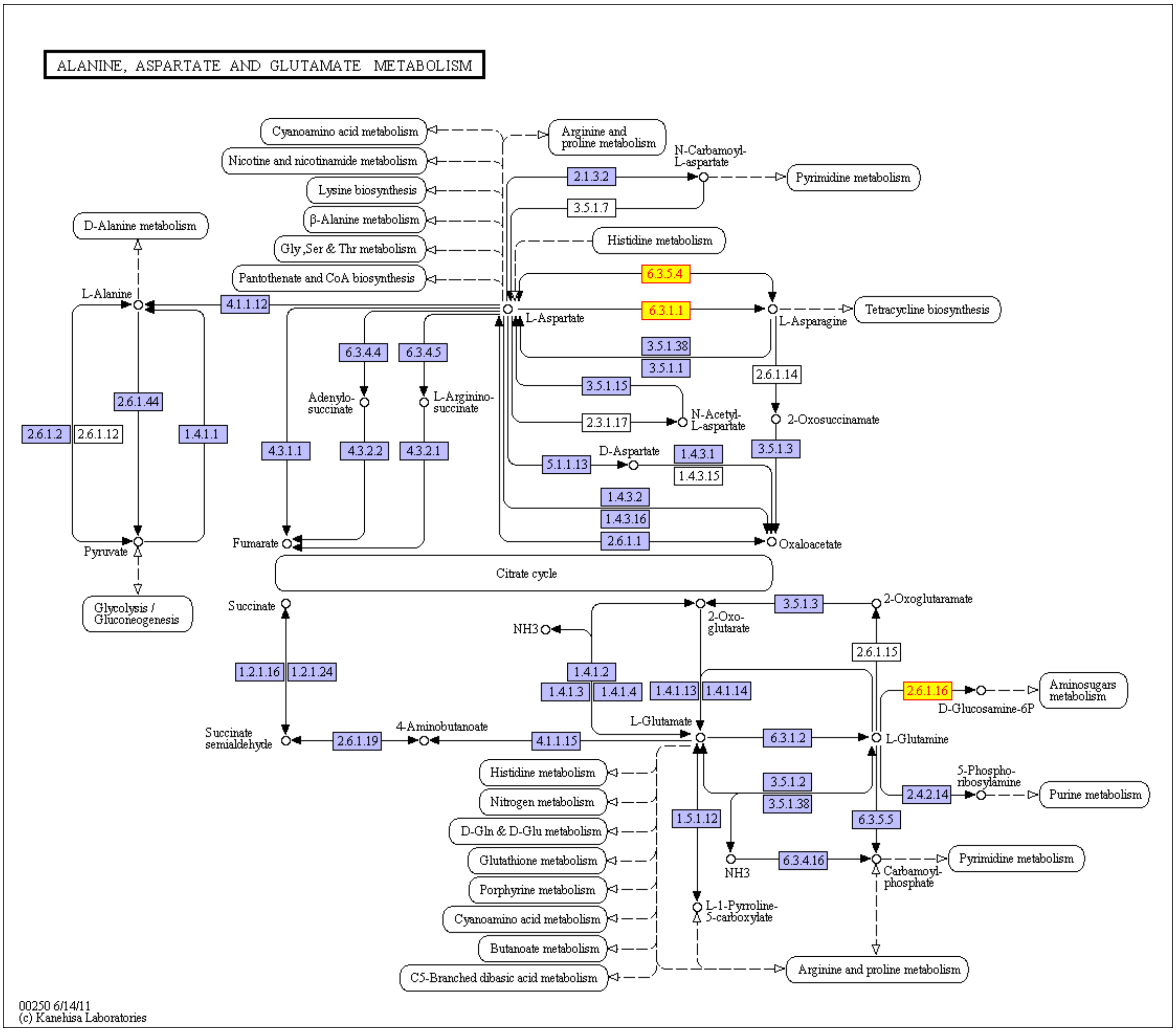
ALANINE, ASPARTATE AND GLUTAMATE METABOLISM
Cyanoamino acid metabolism
Nicotine and nicotinamide metabolism
Lysine biosynthesis
β-Alanine metabolism
Gly, Ser & Thr metabolism
Pantothenate and CoA biosynthesis
Arginine and proline metabolism
N-Carbamoyl-L-aspartate
Pyrimidine metabolism
2.1.3.2
3.5.1.7
Histidine metabolism
D-Alanine metabolism
L-Alanine
4.1.1.12
6.3.5.4
6.3.1.1
L-Aspartate
L-Asparagine
Tetracycline biosynthesis
6.3.4.4
6.3.4.5
3.5.1.38
3.5.1.1
2.6.1.14
2.6.1.44
Adenylo-succinate
L-Arginino-succinate
3.5.1.15
N-Acetyl-L-aspartate
2.3.1.17
2-Oxosuccinamate
2.6.1.2
2.6.1.12
1.4.1.1
4.3.1.1
4.3.2.2
4.3.2.1
3.5.1.3
D-Aspartate
1.4.3.1
5.1.1.13
1.4.3.15
1.4.3.2
1.4.3.16
2.6.1.1
Pyruvate
Fumarate
Oxaloacetate
Citrate cycle
Glycolysis / Gluconeogenesis
Succinate
2-Oxoglutaramate
3.5.1.3
2-Oxo-glutarate
NH3
2.6.1.15
1.2.1.16
1.2.1.24
1.4.1.2
1.4.1.3
1.4.1.4
1.4.1.13
1.4.1.14
2.6.1.16
Aminosugars metabolism
D-Glucosamine-6P
Succinate semialdehyde
2.6.1.19
4-Aminobutanoate
4.1.1.15
L-Glutamate
6.3.1.2
L-Glutamine
5-Phospho-ribosylamine
Histidine metabolism
Nitrogen metabolism
3.5.1.2
3.5.1.38
2.4.2.14
Purine metabolism
D-Gln & D-Glu metabolism
1.5.1.12
6.3.5.5
Glutathione metabolism
NH3
6.3.4.16
Carbamoyl-phosphate
Pyrimidine metabolism
Porphyrine metabolism
L-1-Pyrroline-5-carboxylate
Cyanoamino acid metabolism
Butanoate metabolism
C5-Branched dibasic acid metabolism
Arginine and proline metabolism
00250 6/14/11
(c) Kanehisa Laboratories

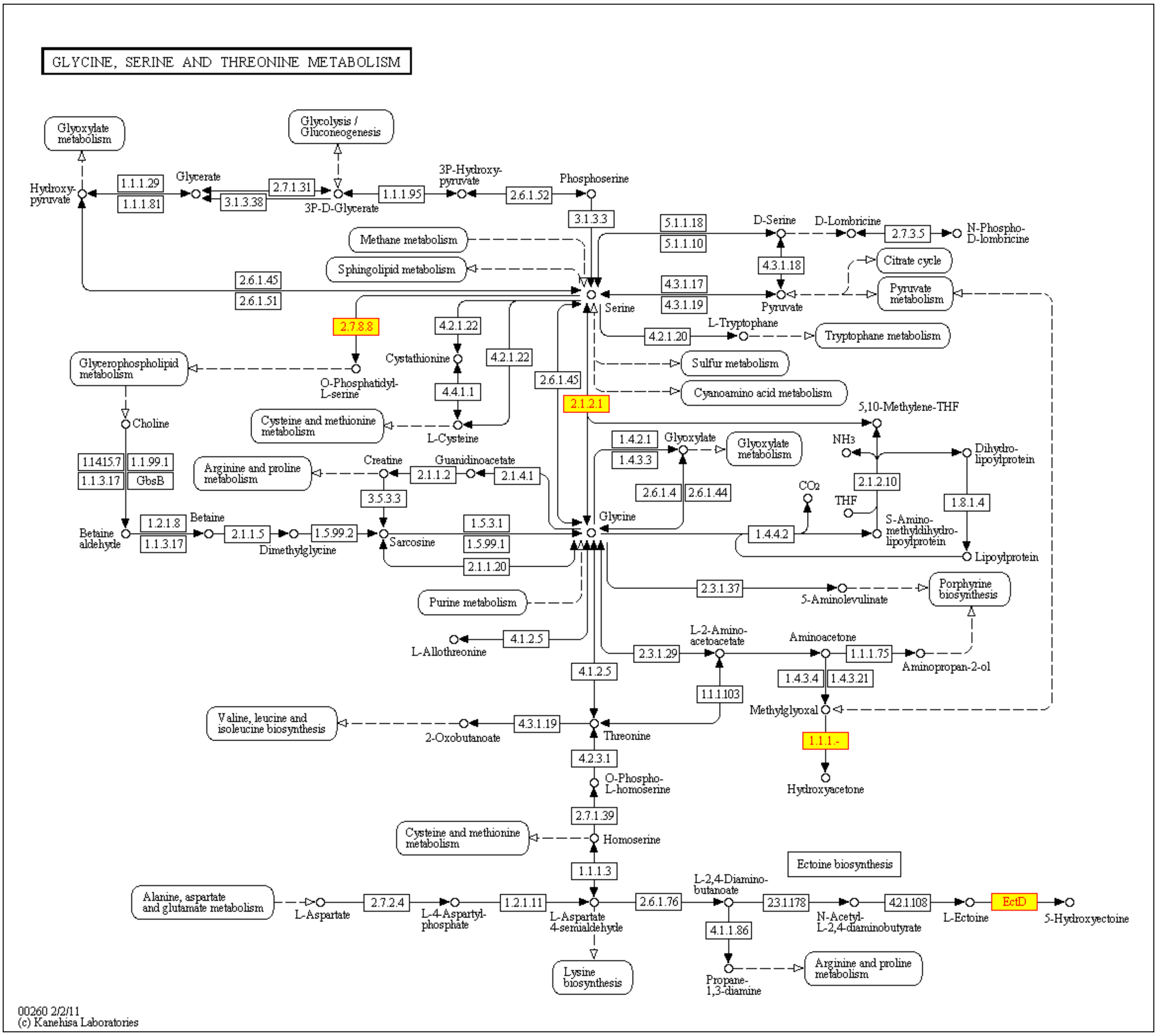
GLYCINE, SERINE AND THREONINE METABOLISM
Glyoxylate metabolism
Glycolysis / Gluconeogenesis
Hydroxy-pyruvate
1.1.1.29
1.1.1.81
Glycerate
2.7.1.31
3.1.3.38
3P-D-Glycerate
1.1.1.95
3P-Hydroxy-pyruvate
2.6.1.52
Phosphoserine
3.1.3.3
5.1.1.18
5.1.1.10
D-Serine
D-Lombricine
2.7.3.5
N-Phospho-D-lombricine
Methane metabolism
Sphingolipid metabolism
4.3.1.18
Citrate cycle
2.6.1.45
2.6.1.51
4.3.1.17
4.3.1.19
Pyruvate
Pyruvate metabolism
Serine
2.7.8.8
4.2.1.22
4.2.1.20
L-Tryptophan
Tryptophan metabolism
Glycerophospholipid metabolism
Cystathionine
4.2.1.22
Sulfur metabolism
O-Phosphatidyl-L-serine
4.4.1.1
2.6.1.45
Cyanoamino acid metabolism
Choline
2.1.2.1
5,10-Methylene-THF
Cysteine and methionine metabolism
L-Cysteine
1.4.2.1
Glyoxylate
Glyoxylate metabolism
NH3
Dihydro-lipoylprotein
1.1.4.15.7
1.1.99.1
1.1.3.17
GbsB
Creatine
Guanidinoacetate
1.4.3.3
Arginine and proline metabolism
2.1.1.2
2.1.4.1
CO2
2.1.2.10
3.5.3.3
2.6.1.4
2.6.1.44
THF
1.8.1.4
Betaine
1.2.1.8
1.5.3.1
Glycine
S-Amino-methyldihydro-lipoylprotein
Betaine aldehyde
1.1.3.17
2.1.1.5
1.5.99.2
Sarcosine
1.5.99.1
1.4.4.2
Dimethylglycine
2.1.1.20
Lipoylprotein
2.3.1.37
5-Aminolevulinate
Porphyrine biosynthesis
Purine metabolism
4.1.2.5
L-Allothreonine
L-2-Amino-acetoacetate
Aminoacetone
2.3.1.29
1.1.1.75
Aminopropan-2-ol
4.1.2.5
1.4.3.4
1.4.3.21
1.1.1.103
Methylglyoxal
Valine, leucine and isoleucine biosynthesis
2-Oxobutanoate
4.3.1.19
Threonine
1.1.1.-
4.2.3.1
Hydroxyacetone
O-Phospho-L-homoserine
2.7.1.39
Cysteine and methionine metabolism
Homoserine
Ectoine biosynthesis
1.1.1.3
Alanine, aspartate and glutamate metabolism
L-Aspartate
2.7.2.4
L-4-Aspartyl-phosphate
1.2.1.11
L-Aspartate 4-semialdehyde
2.6.1.76
L-2,4-Diamino-butanoate
2.3.1.178
N-Acetyl-L-2,4-diaminobutyrate
4.2.1.108
L-Ectoine
EctD
5-Hydroxyectoine
4.1.1.86
Propane-1,3-diamine
Arginine and proline metabolism
Lysine biosynthesis
00260 2/2/11
(c) Kanehisa Laboratories

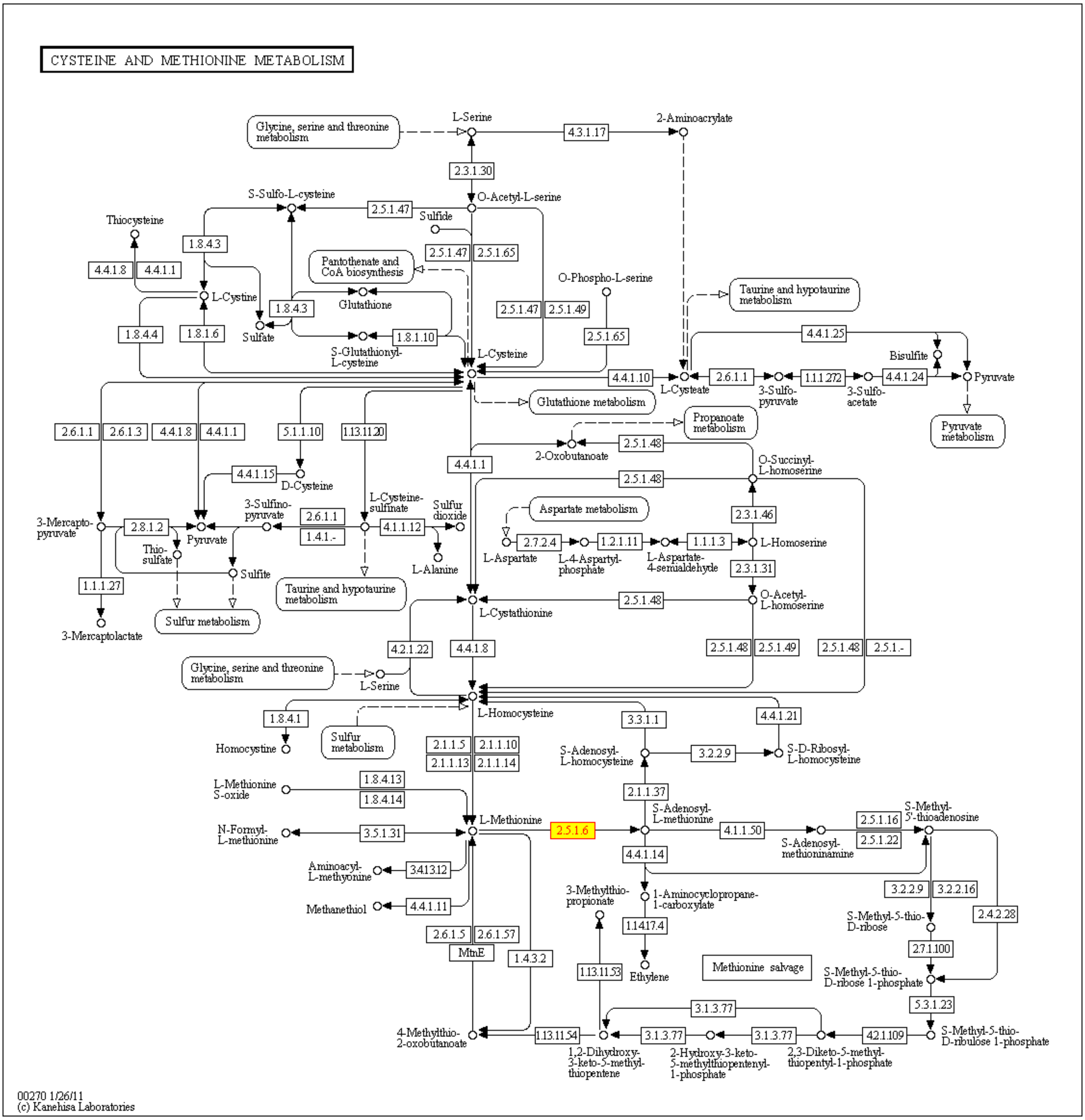

CYSTEINE AND METHIONINE METABOLISM
Glycine, serine and threonine metabolism
L-Serine
4.3.1.17
2-Aminoacrylate
2.3.1.30
O-Acetyl-L-serine
S-Sulfo-L-cysteine
2.5.1.47
Sulfide
Thiocysteine
1.8.4.3
4.4.1.8
4.4.1.1
2.5.1.47
2.5.1.65
Pantothenate and CoA biosynthesis
O-Phospho-L-serine
L-Cystine
1.8.4.3
Glutathione
Taurine and hypotaurine metabolism
2.5.1.47
2.5.1.49
1.8.4.4
1.8.1.6
Sulfate
1.8.1.10
S-Glutathionyl-L-cysteine
2.5.1.65
L-Cysteine
4.4.1.25
Bisulfite
4.4.1.10
L-Cysteate
2.6.1.1
3-Sulfo-pyruvate
1.1.1.272
3-Sulfo-acetate
4.4.1.24
Pyruvate
Glutathione metabolism
Propanoate metabolism
Pyruvate metabolism
2.6.1.1
2.6.1.3
4.4.1.8
4.4.1.1
5.1.1.10
1.13.11.20
2.5.1.48
2-Oxobutanoate
4.4.1.1
O-Succinyl-L-homoserine
4.4.1.15
D-Cysteine
2.5.1.48
3-Sulfino-pyruvate
L-Cysteine-sulfinate
Sulfur dioxide
2.3.1.46
Aspartate metabolism
3-Mercapto-pyruvate
2.8.1.2
Pyruvate
2.6.1.1
1.4.1.-
4.1.1.12
2.7.2.4
1.2.1.11
1.1.1.3
L-Homoserine
Thio-sulfate
Sulfite
L-Alanine
L-Aspartate
L-4-Aspartyl-phosphate
L-Aspartate-4-semialdehyde
2.3.1.31
1.1.1.27
Taurine and hypotaurine metabolism
O-Acetyl-L-homoserine
3-Mercaptolactate
Sulfur metabolism
L-Cystathionine
2.5.1.48
4.2.1.22
4.4.1.8
2.5.1.48
2.5.1.49
2.5.1.48
2.5.1.-
Glycine, serine and threonine metabolism
L-Serine
L-Homocysteine
1.8.4.1
3.3.1.1
4.4.1.21
Sulfur metabolism
Homocystine
2.1.1.5
2.1.1.10
2.1.1.13
2.1.1.14
S-Adenosyl-L-homocysteine
3.2.2.9
S-D-Ribosyl-L-homocysteine
L-Methionine S-oxide
1.8.4.13
1.8.4.14
2.1.1.37
S-Adenosyl-L-methionine
S-Methyl-5'-thioadenosine
L-Methionine
2.5.1.6
4.1.1.50
2.5.1.16
N-Formyl-L-methionine
3.5.1.31
S-Adenosyl-methioninamine
2.5.1.22
4.4.1.14
Aminoacyl-L-methyonine
3.4.13.12
3-Methylthio-propionate
1-Aminocyclopropane-1-carboxylate
3.2.2.9
3.2.2.16
Methanethiol
4.4.1.11
S-Methyl-5-thio-D-ribose
2.4.2.28
1.14.17.4
2.6.1.5
2.6.1.57
2.7.1.100
MtnE
1.4.3.2
1.13.11.53
Ethylene
Methionine salvage
S-Methyl-5-thio-D-ribose 1-phosphate
5.3.1.23
3.1.3.77
4-Methylthio-2-oxobutanoate
1.13.11.54
3.1.3.77
3.1.3.77
4.2.1.109
S-Methyl-5-thio-D-ribulose 1-phosphate
1,2-Dihydroxy-3-keto-5-methyl-thiopentene
2-Hydroxy-3-keto-5-methylthiopentenyl-1-phosphate
2,3-Diketo-5-methyl-thiopentyl-1-phosphate
00270 1/26/11
(c) Kanehisa Laboratories

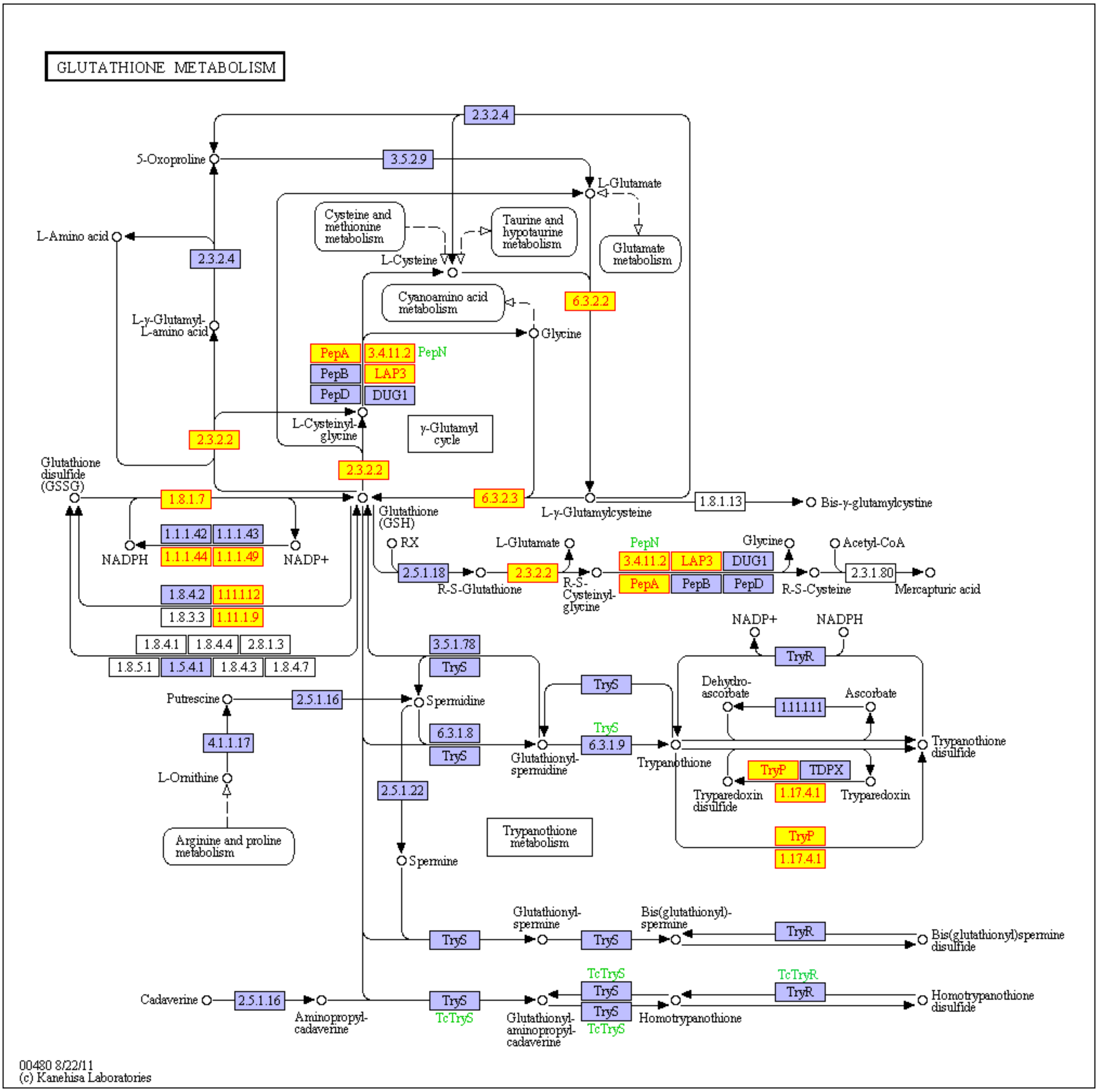
GLUTATHIONE METABOLISM
2.3.2.4
5-Oxoproline
3.5.2.9
L-Glutamate
Cysteine and methionine metabolism
Taurine and hypotaurine metabolism
Glutamate metabolism
L-Amino acid
2.3.2.4
L-Cysteine
Cyanoamino acid metabolism
6.3.2.2
L-γ-Glutamyl-L-amino acid
Glycine
PepA
3.4.11.2
PepN
PepB
LAP3
PepD
DUG1
L-Cysteinyl-glycine
γ-Glutamyl cycle
2.3.2.2
2.3.2.2
Glutathione disulfide (GSSG)
1.8.1.7
6.3.2.3
Glutathione (GSH)
L-γ-Glutamylcysteine
1.8.1.13
Bis-γ-glutamylcystine
NADPH
1.1.1.42
1.1.1.43
NADP+
1.1.1.44
1.1.1.49
RX
L-Glutamate
PepN
Glycine
Acetyl-CoA
2.5.1.18
2.3.2.2
3.4.11.2
LAP3
DUG1
2.3.1.80
R-S-Glutathione
R-S-Cysteinyl-glycine
PepA
PepB
PepD
R-S-Cysteine
Mercapturic acid
1.8.4.2
1.11.1.12
1.8.3.3
1.11.1.9
NADP+
NADPH
1.8.4.1
1.8.4.4
2.8.1.3
3.5.1.78
TryR
1.8.5.1
1.5.4.1
1.8.4.3
1.8.4.7
TryS
TryS
Dehydro-ascorbate
Ascorbate
Putrescine
2.5.1.16
Spermidine
1.11.1.11
4.1.1.17
6.3.1.8
TryS
Trypanothione disulfide
TryS
6.3.1.9
Glutathionyl-spermidine
Trypanothione
TryP
TDPX
L-Ornithine
2.5.1.22
Trypanodoxin disulfide
1.17.4.1
Tryparedoxin
Arginine and proline metabolism
Trypanothione metabolism
TryP
1.17.4.1
Spermine
Glutathionyl-spermine
Bis(glutathionyl)-spermine
TryS
TryS
TryR
Bis(glutathionyl)spermine disulfide
TcTryS
TcTryR
TryS
TryR
Cadaverine
2.5.1.16
TryS
TryS
Homotrypanothione disulfide
Aminopropyl-cadaverine
TcTryS
Glutathionyl-aminopropyl-cadaverine
TcTryS
Homotrypanothione
00480 8/22/11
(c) Kanehisa Laboratories

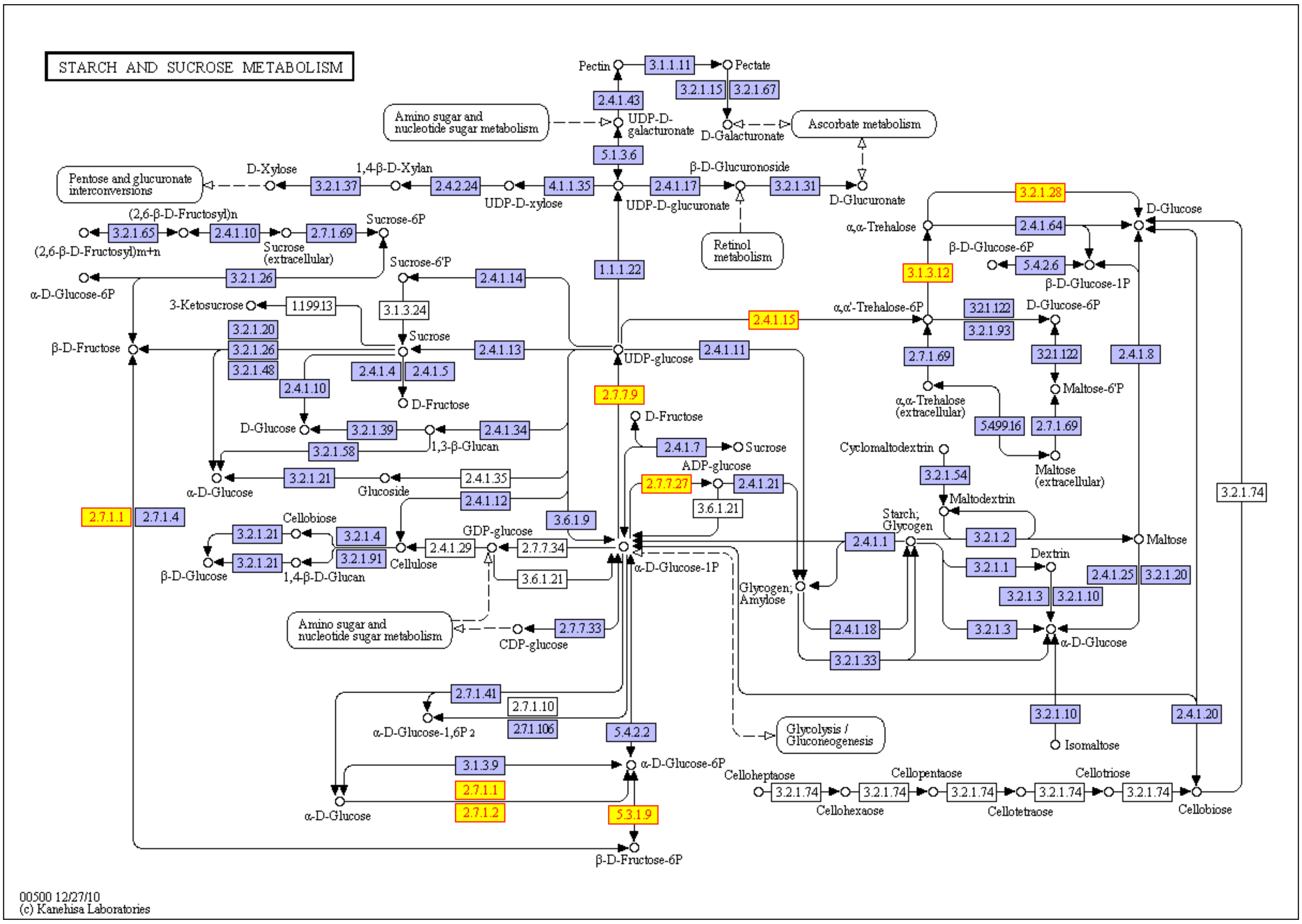

STARCH AND SUCROSE METABOLISM
Pectin
3.1.1.11
Pectate
3.2.1.15
3.2.1.67
2.4.1.43
Amino sugar and nucleotide sugar metabolism
UDP-D-galacturonate
D-Galacturonate
Ascorbate metabolism
5.1.3.6
Pentose and glucuronate interconversions
D-Xylose
3.2.1.37
1,4-β-D-Xylan
2.4.2.24
UDP-D-xylose
4.1.1.35
2.4.1.17
β-D-Glucuronoside
3.2.1.31
D-Glucuronate
UDP-D-glucuronate
Retinol metabolism
3.2.1.28
D-Glucose
α,α-Trehalose
2.4.1.64
β-D-Glucose-6P
5.4.2.6
β-D-Glucose-1P
3.1.3.12
(2,6-β-D-Fructosyl)n
3.2.1.65
2.4.1.10
2.7.1.69
Sucrose-6P
(2,6-β-D-Fructosyl)m+n
Sucrose (extracellular)
Sucrose-6'P
3.2.1.26
α-D-Glucose-6P
2.4.1.14
1.1.1.22
3-Ketosucrose
1.1.99.13
3.1.3.24
Sucrose
3.2.1.20
3.2.1.26
3.2.1.48
β-D-Fructose
2.4.1.13
2.4.1.4
2.4.1.5
D-Fructose
2.4.1.10
UDP-glucose
2.4.1.11
2.4.1.15
α,α'-Trehalose-6P
3.2.1.122
3.2.1.93
D-Glucose-6P
2.7.1.69
3.2.1.122
2.4.1.8
α,α-Trehalose (extracellular)
Maltose-6'P
5.4.99.16
2.7.1.69
Maltose (extracellular)
2.7.7.9
D-Glucose
3.2.1.39
2.4.1.34
1,3-β-Glucan
3.2.1.58
D-Fructose
2.4.1.7
Sucrose
Cyclomaltodextrin
3.2.1.54
Maltodextrin
α-D-Glucose
3.2.1.21
2.4.1.35
Glucoside
2.7.7.27
ADP-glucose
2.4.1.21
3.6.1.21
2.4.1.12
2.7.1.1
2.7.1.4
Cellobiose
3.2.1.21
3.2.1.4
β-D-Glucose
3.2.1.21
3.2.1.91
1,4-β-D-Glucan
Cellulose
GDP-glucose
2.4.1.29
2.7.7.34
3.6.1.9
3.6.1.21
α-D-Glucose-1P
Starch; Glycogen
2.4.1.1
3.2.1.2
Maltose
3.2.1.74
Glycogen; Amylose
3.2.1.1
Dextrin
2.4.1.25
3.2.1.20
3.2.1.3
3.2.1.10
Amino sugar and nucleotide sugar metabolism
CDP-glucose
2.7.7.33
2.4.1.18
3.2.1.3
α-D-Glucose
3.2.1.33
2.7.1.41
2.7.1.10
α-D-Glucose-1,6P2
2.7.1.106
5.4.2.2
Glycolysis / Gluconeogenesis
3.2.1.10
2.4.1.20
Isomaltose
3.1.3.9
α-D-Glucose-6P
Celloheptaose
3.2.1.74
Cellopentaose
3.2.1.74
3.2.1.74
Cellotriose
3.2.1.74
Cellobiose
Cellohexaose
Cellotetraose
2.7.1.1
2.7.1.2
α-D-Glucose
5.3.1.9
β-D-Fructose-6P
00500 12/27/10
(c) Kanehisa Laboratories

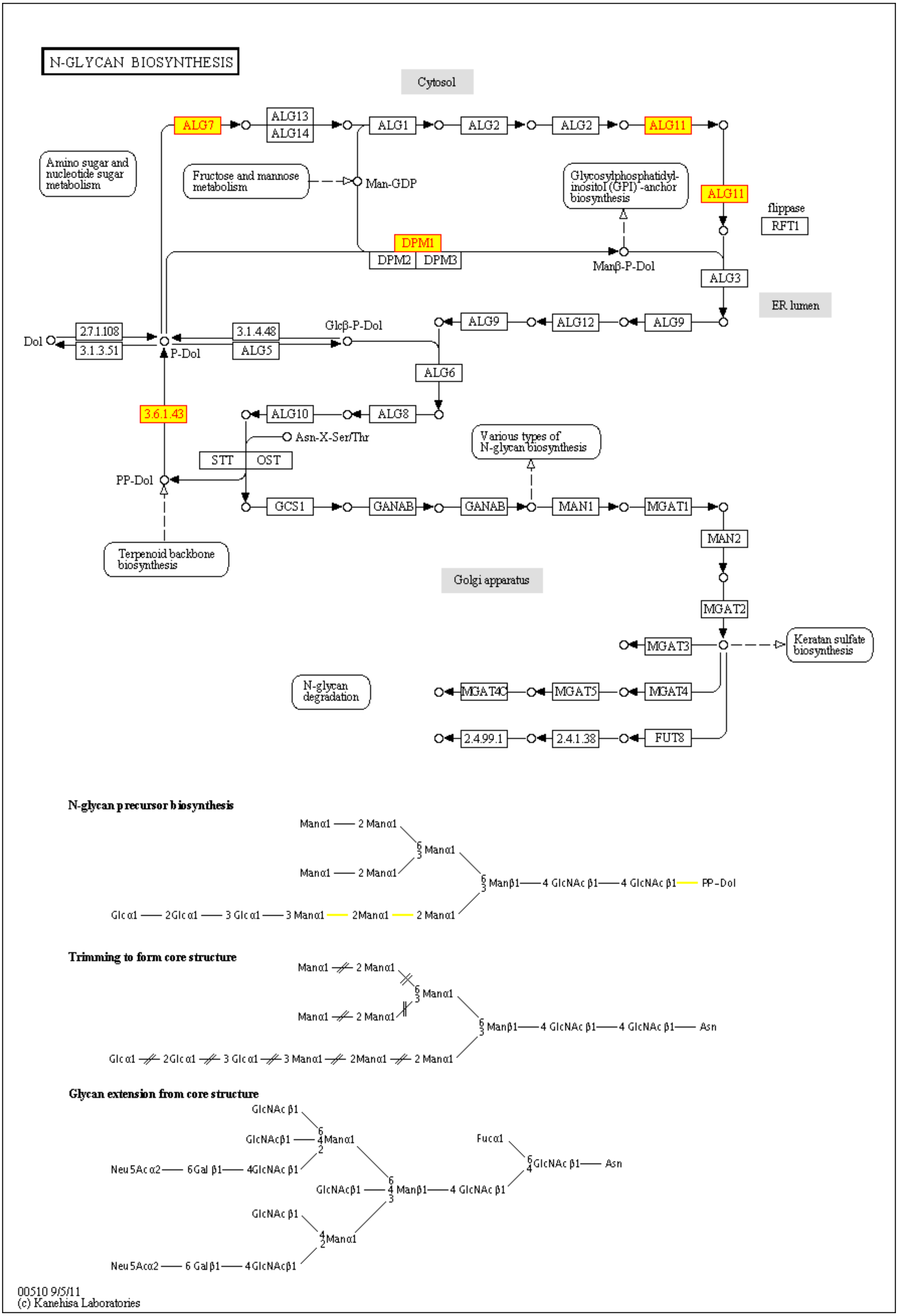

N-GLYCAN BIOSYNTHESIS
Cytosol
ALG7
ALG13
ALG14
ALG1
ALG2
ALG2
ALG11
ALG11
flippase
RFT1
Amino sugar and nucleotide sugar metabolism
Fructose and mannose metabolism
Man-GDP
Glycosylphosphatidyl-inositol (GPI) -anchor biosynthesis
DPM1
DPM2
DPM3
Manβ-P-Dol
ALG3
ER lumen
Dol
2.7.1.108
3.1.3.51
P-Dol
3.1.4.48
ALG5
Glcβ-P-Dol
ALG9
ALG12
ALG9
ALG6
3.6.1.43
ALG10
ALG8
Asn-X-Ser/Thr
STT
OST
Various types of N-glycan biosynthesis
PP-Dol
GCS1
GANAB
GANAB
MAN1
MGAT1
MAN2
Terpenoid backbone biosynthesis
Golgi apparatus
MGAT2
MGAT3
Keratan sulfate biosynthesis
N-glycan degradation
MGAT4C
MGAT5
MGAT4
2.4.99.1
2.4.1.38
FUT8
N-glycan precursor biosynthesis
Manα1 — 2 Manα1
Manα1 — 2 Manα1
6 3 Manα1
6 3 Manβ1 — 4 GlcNAc β1 — 4 GlcNAc β1 — PP-Dol
Glcα1 — 2Glcα1 — 3 Glcα1 — 3 Manα1 — 2Manα1 — 2 Manα1
Trimming to form core structure
Manα1 — 2 Manα1
Manα1 — 2 Manα1
6 3 Manα1
6 3 Manβ1 — 4 GlcNAc β1 — 4 GlcNAc β1 — Asn
Glcα1 — 2Glcα1 — 3 Glcα1 — 3 Manα1 — 2Manα1 — 2 Manα1
Glycan extension from core structure
GlcNAc β1
GlcNAcβ1 — 6 4 2 Manα1
Neu5Acα2 — 6Gal β1 — 4GlcNAc β1
Fucα1
6 4 GlcNAc β1 — Asn
GlcNAcβ1 — 6 4 3 Manβ1 — 4 GlcNAc β1
GlcNAc β1
4 2 Manα1
Neu5Acα2 — 6 Gal β1 — 4GlcNAcβ1
00510 9/5/11
(c) Kanehisa Laboratories

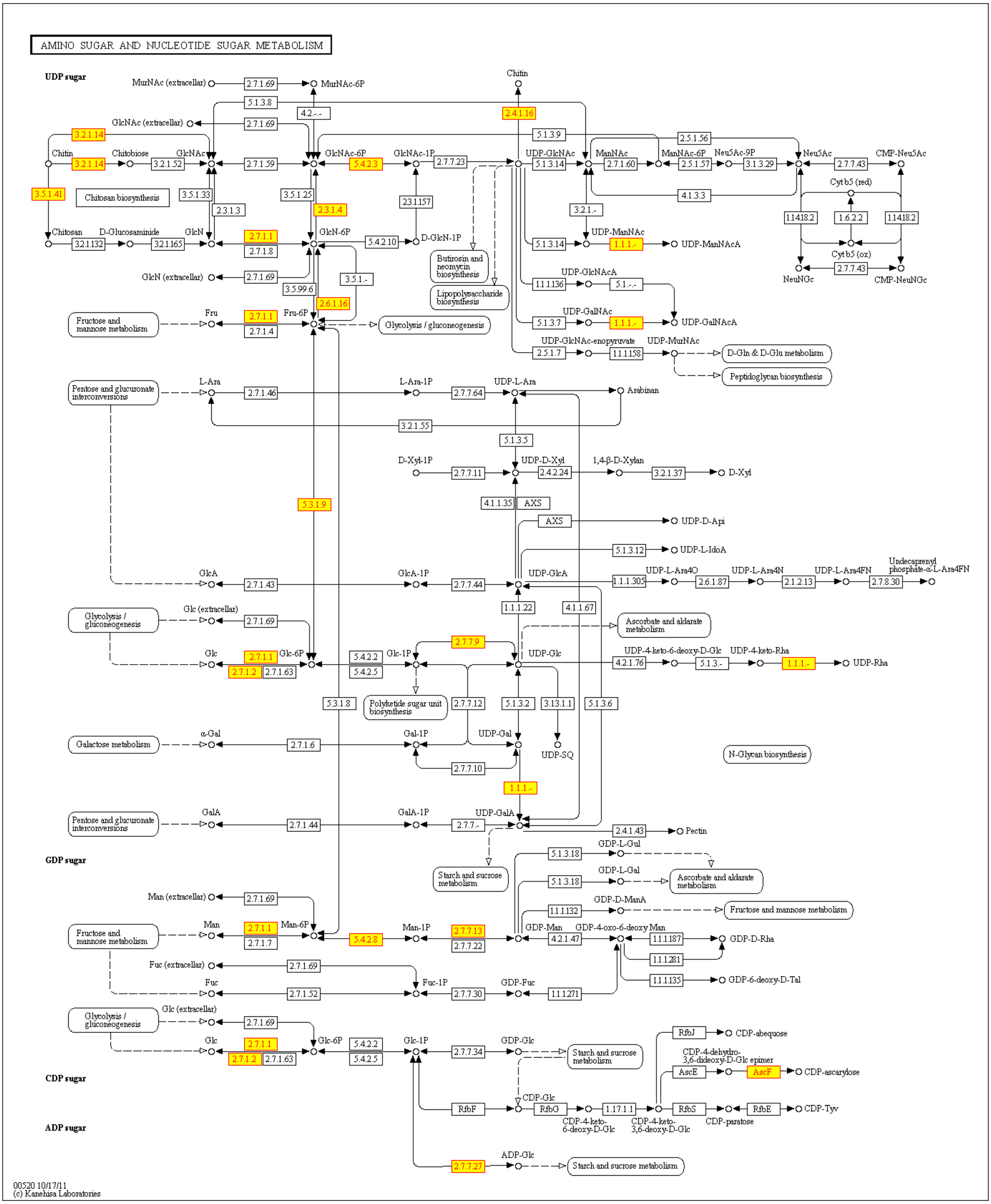

AMINO SUGAR AND NUCLEOTIDE SUGAR METABOLISM
UDP sugar
MurNAc (extracellar)
2.7.1.69
MurNAc-6P
Chitin
5.1.3.8
4.2.-.-
2.4.1.16
GlcNAc (extracellar)
2.7.1.69
3.2.1.14
5.1.3.9
2.5.1.56
Chitin
Chitobiose
GlcNAc
GlcNAc-6P
GlcNAc-1P
UDP-GlcNAc
ManNAc
ManNAc-6P
Neu5Ac-9P
Neu5Ac
CMP-Neu5Ac
3.2.1.14
3.2.1.52
2.7.1.59
5.4.2.3
2.7.7.23
5.1.3.14
2.7.1.60
2.5.1.57
3.1.3.29
2.7.7.43
4.1.3.3
Cyt b5 (red)
3.5.1.41
Chitosan biosynthesis
3.5.1.33
3.5.1.25
2.3.1.4
2.3.1.157
3.2.1.-
1.14.18.2
1.6.2.2
1.14.18.2
2.3.1.3
Chitosan
D-Glucosaminide
GlcN
GlcN-6P
D-GlcN-1P
UDP-ManNAc
3.2.1.132
3.2.1.165
2.7.1.1
2.7.1.8
5.4.2.10
5.1.3.14
1.1.1.-
UDP-ManNAcA
Cyt b5 (ox)
Butirosin and neomycin biosynthesis
UDP-GlcNAcA
GlcN (extracellar)
2.7.1.69
3.5.1.-
2.7.7.43
NeuNGc
CMP-NeuNGc
3.5.99.6
1.1.1.136
5.1.-.-
Lipopolysaccharide biosynthesis
2.6.1.16
UDP-GalNAc
Fru
Fru-6P
Fructose and mannose metabolism
2.7.1.1
2.7.1.4
Glycolysis / gluconeogenesis
5.1.3.7
1.1.1.-
UDP-GalNAcA
UDP-GlcNAc-enolpyruvate
UDP-MurNAc
2.5.1.7
1.1.1.158
D-Gln & D-Glu metabolism
Peptidoglycan biosynthesis
Pentose and glucuronate interconversions
L-Ara
2.7.1.46
L-Ara-1P
2.7.7.64
UDP-L-Ara
Arabinan
3.2.1.55
5.1.3.5
D-Xyl-1P
2.7.7.11
UDP-D-Xyl
2.4.2.24
1,4-β-D-Xylan
3.2.1.37
D-Xyl
5.3.1.9
4.1.1.35
AXS
AXS
UDP-D-Api
5.1.3.12
UDP-L-IdoA
GlcA
2.7.1.43
GlcA-1P
2.7.7.44
UDP-GlcA
1.1.1.305
UDP-L-Ara4O
2.6.1.87
UDP-L-Ara4N
2.1.2.13
UDP-L-Ara4FN
2.7.8.30
Undecaprenyl phosphate-α-L-Ara4FN
1.1.1.22
4.1.1.67
Glycolysis / gluconeogenesis
Glc (extracellar)
2.7.1.69
Ascorbate and aldarate metabolism
Glc
2.7.1.1
2.7.1.2
2.7.1.63
Glc-6P
5.4.2.2
5.4.2.5
Glc-1P
2.7.7.9
UDP-Glc
UDP-4-keto-6-deoxy-D-Glc
UDP-4-keto-Rha
4.2.1.76
5.1.3.-
1.1.1.-
UDP-Rha
5.3.1.8
Polyketide sugar unit biosynthesis
2.7.7.12
5.1.3.2
3.13.1.1
5.1.3.6
Galactose metabolism
α-Gal
2.7.1.6
Gal-1P
UDP-Gal
UDP-SQ
N-Glycan biosynthesis
2.7.7.10
1.1.1.-
Pentose and glucuronate interconversions
GalA
2.7.1.44
GalA-1P
2.7.7.-
UDP-GalA
2.4.1.43
Pectin
GDP sugar
GDP-L-Gul
5.1.3.18
Starch and sucrose metabolism
GDP-L-Gal
5.1.3.18
Ascorbate and aldarate metabolism
GDP-D-ManA
1.1.1.132
Fructose and mannose metabolism
Man (extracellar)
2.7.1.69
Man
Man-6P
Man-1P
2.7.7.13
GDP-Man
GDP-4-oxo-6-deoxy Man
Fructose and mannose metabolism
2.7.1.1
2.7.1.7
5.4.2.8
2.7.7.22
4.2.1.47
1.1.1.187
GDP-D-Rha
1.1.1.281
Fuc (extracellar)
2.7.1.69
Fuc
Fuc-1P
GDP-Fuc
1.1.1.135
GDP-6-deoxy-D-Tal
2.7.1.52
2.7.7.30
1.1.1.271
Glycolysis / gluconeogenesis
Glc (extracellar)
2.7.1.69
Glc
Glc-6P
Glc-1P
GDP-Glc
RfbJ
CDP-abequose
2.7.1.1
5.4.2.2
2.7.7.34
Starch and sucrose metabolism
CDP-4-dehydro-3,6-dideoxy-D-Glc epimer
2.7.1.2
2.7.1.63
5.4.2.5
AscE
AscF
CDP-ascarylose
CDP sugar
CDP-Glc
RfbF
RfbG
1.17.1.1
RfbS
RfbE
CDP-Tyv
CDP-4-keto-6-deoxy-D-Glc
CDP-4-keto-3,6-deoxy-D-Glc
CDP-paratose
ADP sugar
ADP-Glc
2.7.7.27
Starch and sucrose metabolism
00520 10/17/11
(c) Kanehisa Laboratories

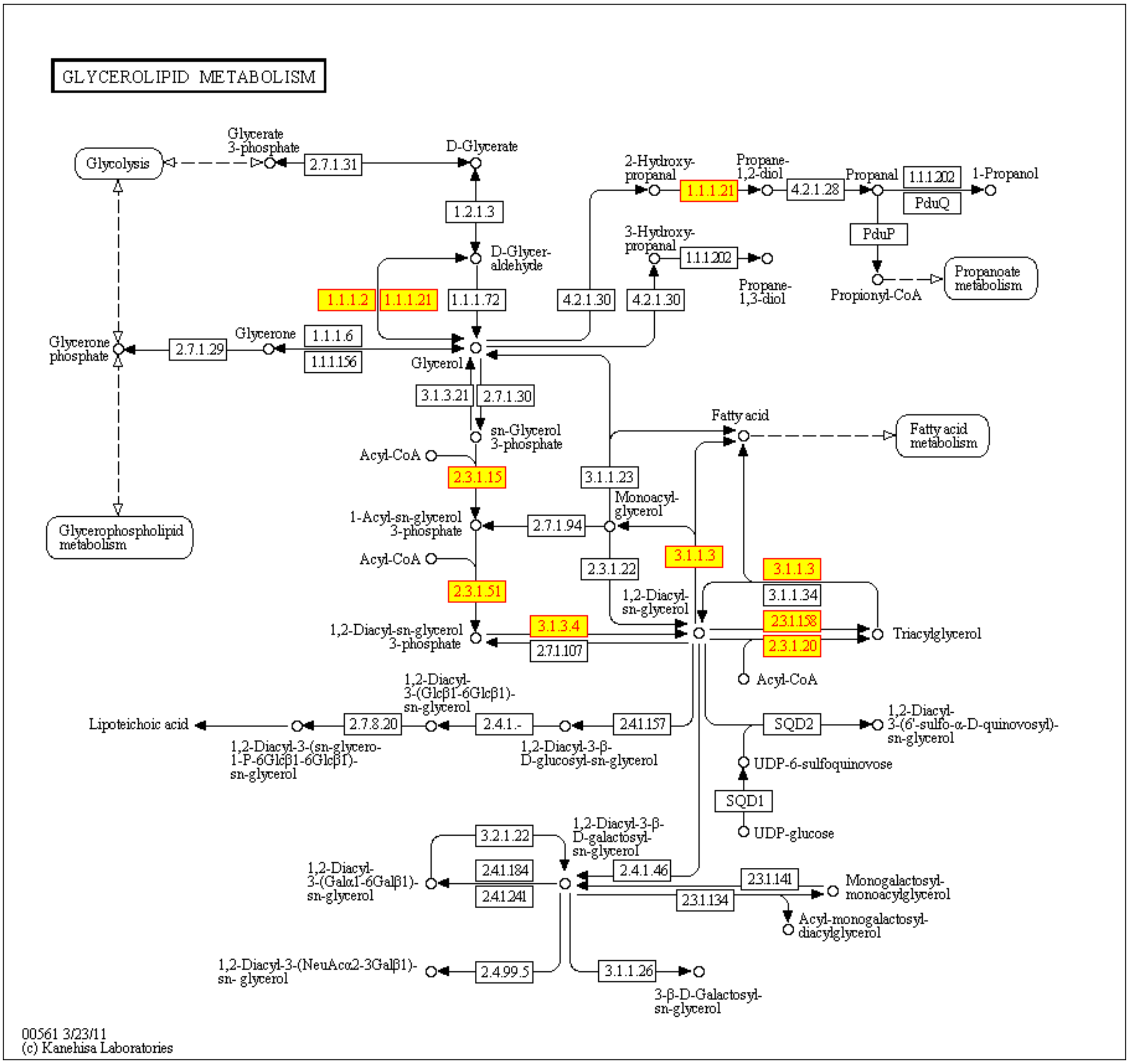
GLYCEROLIPID METABOLISM
Glycolysis
Glycerate 3-phosphate
2.7.1.31
D-Glycerate
1.2.1.3
D-Glyceraldehyde
2-Hydroxy-propanal
1.1.1.21
Propane-1,2-diol
4.2.1.28
Propanal
1.1.1.202
PduQ
1-Propanol
PduP
Propionyl-CoA
Propanoate metabolism
3-Hydroxy-propanal
1.1.1.202
Propane-1,3-diol
1.1.1.2
1.1.1.21
1.1.1.72
4.2.1.30
4.2.1.30
Glycerone phosphate
2.7.1.29
Glycerone
1.1.1.6
1.1.1.156
Glycerol
3.1.3.21
2.7.1.30
sn-Glycerol 3-phosphate
Fatty acid
Fatty acid metabolism
Acyl-CoA
2.3.1.15
3.1.1.23
Monoacylglycerol
1-Acyl-sn-glycerol 3-phosphate
2.7.1.94
Glycerophospholipid metabolism
Acyl-CoA
3.1.1.3
3.1.1.3
2.3.1.22
2.3.1.51
3.1.1.34
1,2-Diacyl-sn-glycerol
2.3.1.158
1,2-Diacyl-sn-glycerol 3-phosphate
3.1.3.4
2.7.1.107
Triacylglycerol
2.3.1.20
Acyl-CoA
1,2-Diacyl-3-(Glcβ1-6Glcβ1)-sn-glycerol
Lipoteichoic acid
2.7.8.20
2.4.1.-
2.4.1.157
SQD2
1,2-Diacyl-3-(6'-sulfo-α-D-quinovosyl)-sn-glycerol
1,2-Diacyl-3-(sn-glycero-1-P-6Glcβ1-6Glcβ1)-sn-glycerol
1,2-Diacyl-3-β-D-glucosyl-sn-glycerol
UDP-6-sulfoquinovose
SQD1
UDP-glucose
3.2.1.22
1,2-Diacyl-3-β-D-galactosyl-sn-glycerol
1,2-Diacyl-3-(Galα1-6Galβ1)-sn-glycerol
2.4.1.184
2.4.1.241
2.4.1.46
2.3.1.141
Monogalactosyl-monoacylglycerol
2.3.1.134
Acyl-monogalactosyl-diacylglycerol
1,2-Diacyl-3-(NeuAcα2-3Galβ1)-sn-glycerol
2.4.99.5
3.1.1.26
3-β-D-Galactosyl-sn-glycerol
00561 3/23/11
(c) Kanehisa Laboratories

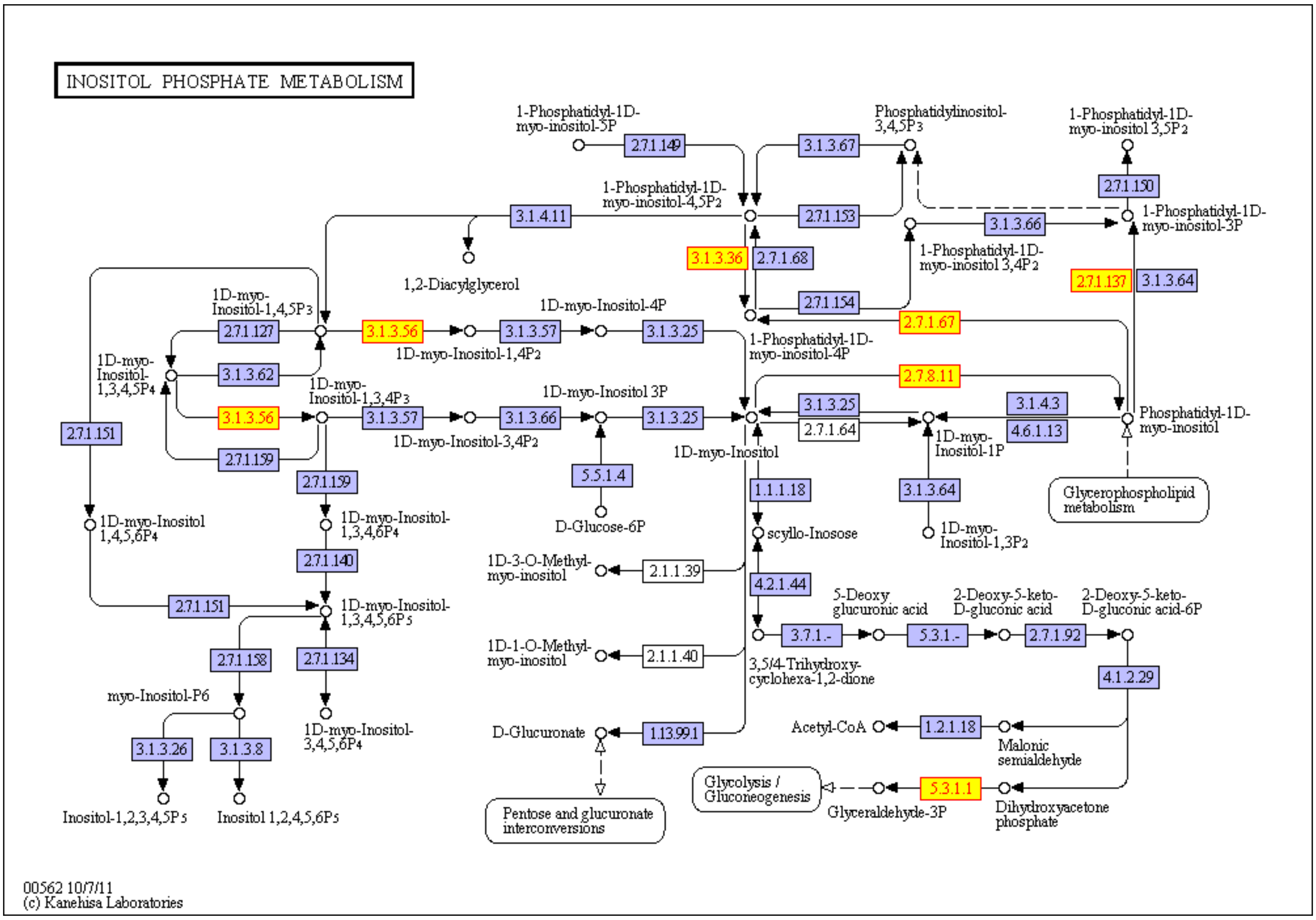
INOSITOL PHOSPHATE METABOLISM
1-Phosphatidyl-1D-myo-inositol-5P
2.7.1.149
3.1.3.67
Phosphatidylinositol-3,4,5P3
1-Phosphatidyl-1D-myo-inositol 3,5P2
2.7.1.150
1-Phosphatidyl-1D-myo-inositol-4,5P2
3.1.4.11
2.7.1.153
3.1.3.66
1-Phosphatidyl-1D-myo-inositol-3P
3.1.3.36
2.7.1.68
1-Phosphatidyl-1D-myo-inositol 3,4P2
1,2-Diacylglycerol
2.7.1.137
3.1.3.64
2.7.1.154
1D-myo-Inositol-1,4,5P3
1D-myo-Inositol-4P
2.7.1.67
2.7.1.127
3.1.3.56
3.1.3.57
3.1.3.25
1D-myo-Inositol-1,4P2
1-Phosphatidyl-1D-myo-inositol-4P
1D-myo-Inositol-1,3,4,5P4
3.1.3.62
2.7.8.11
1D-myo-Inositol-1,3,4P3
1D-myo-Inositol 3P
3.1.3.25
3.1.4.3
Phosphatidyl-1D-myo-inositol
2.7.1.151
3.1.3.56
3.1.3.57
3.1.3.66
3.1.3.25
2.7.1.64
4.6.1.13
1D-myo-Inositol-3,4P2
1D-myo-Inositol-1P
2.7.1.159
1D-myo-Inositol
5.5.1.4
2.7.1.159
1.1.1.18
3.1.3.64
Glycerophospholipid metabolism
1D-myo-Inositol 1,4,5,6P4
1D-myo-Inositol-1,3,4,6P4
D-Glucose-6P
scyllo-Inosose
1D-myo-Inositol-1,3P2
2.7.1.140
1D-3-O-Methyl-myo-inositol
2.1.1.39
4.2.1.44
5-Deoxy glucuronic acid
2-Deoxy-5-keto-D-gluconic acid
2-Deoxy-5-keto-D-gluconic acid-6P
2.7.1.151
1D-myo-Inositol-1,3,4,5,6P5
3.7.1.-
5.3.1.-
2.7.1.92
1D-1-O-Methyl-myo-inositol
2.1.1.40
2.7.1.158
2.7.1.134
3,5/4-Trihydroxy-cyclohexa-1,2-dione
4.1.2.29
myo-Inositol-P6
D-Glucuronate
1.13.99.1
Acetyl-CoA
1.2.1.18
1D-myo-Inositol-3,4,5,6P4
Malonic semialdehyde
3.1.3.26
3.1.3.8
Glycolysis / Gluconeogenesis
5.3.1.1
Inositol-1,2,3,4,5P5
Inositol 1,2,4,5,6P5
Pentose and glucuronate interconversions
Glyceraldehyde-3P
Dihydroxyacetone phosphate
00562 10/7/11
(c) Kanehisa Laboratories

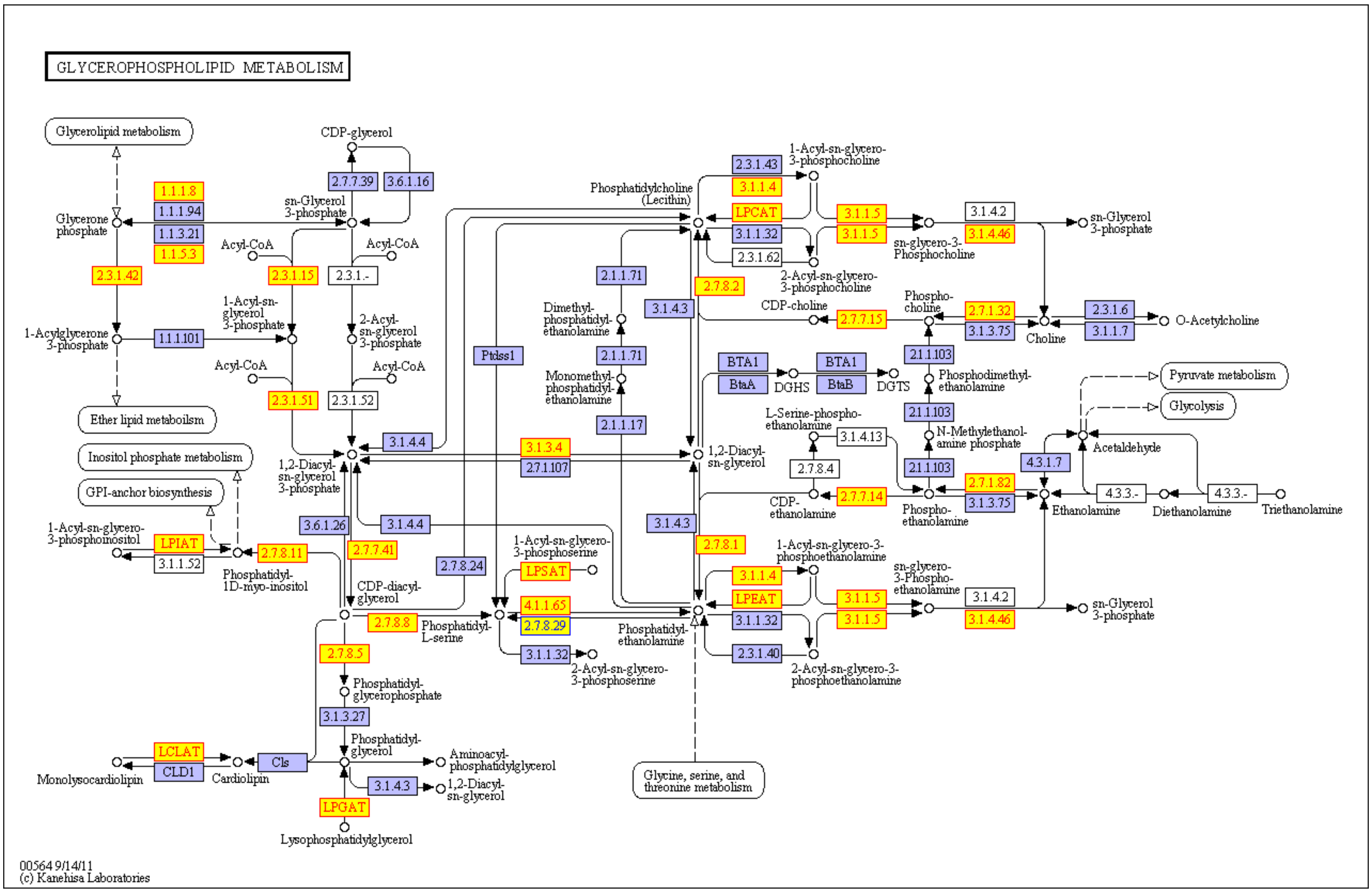

GLYCEROPHOSPHOLIPID METABOLISM
Glycerolipid metabolism
CDP-glycerol
Glycerone phosphate
sn-Glycerol 3-phosphate
1-Acylglycerone 3-phosphate
1-Acyl-sn-glycerol 3-phosphate
2-Acyl-sn-glycerol 3-phosphate
Acyl-CoA
Ether lipid metabolism
Inositol phosphate metabolism
GPI-anchor biosynthesis
1,2-Diacyl-sn-glycerol 3-phosphate
1-Acyl-sn-glycero-3-phosphoinositol
Phosphatidyl-1D-myo-inositol
CDP-diacylglycerol
Phosphatidyl-L-serine
Phosphatidylglycerophosphate
Phosphatidylglycerol
Aminoacylphosphatidylglycerol
1,2-Diacyl-sn-glycerol
Lysophosphatidylglycerol
Monolysocardiolipin
Cardiolipin
Phosphatidylcholine (Lecithin)
1-Acyl-sn-glycero-3-phosphocholine
2-Acyl-sn-glycero-3-phosphocholine
sn-glycero-3-Phosphocholine
Dimethyl-phosphatidyl-ethanolamine
Monomethyl-phosphatidyl-ethanolamine
CDP-choline
Phosphocholine
Choline
O-Acetylcholine
DGHS
DGTS
Phosphodimethyl-ethanolamine
Pyruvate metabolism
Glycolysis
L-Serine-phospho-ethanolamine
N-Methylethanolamine phosphate
Acetaldehyde
CDP-ethanolamine
Phosphoethanolamine
Ethanolamine
Diethanolamine
Triethanolamine
1-Acyl-sn-glycero-3-phosphoserine
2-Acyl-sn-glycero-3-phosphoserine
Phosphatidylethanolamine
1-Acyl-sn-glycero-3-phosphoethanolamine
2-Acyl-sn-glycero-3-phosphoethanolamine
sn-glycero-3-Phosphoethanolamine
Glycine, serine, and threonine metabolism
1.1.1.8
1.1.1.94
1.1.3.21
1.1.5.3
2.3.1.42
1.1.1.101
2.7.7.39
3.6.1.16
2.3.1.15
2.3.1.-
2.3.1.51
2.3.1.52
3.1.4.4
3.6.1.26
LPIAT
3.1.1.52
2.7.8.11
2.7.7.41
2.7.8.24
3.1.3.4
2.7.1.107
2.7.8.8
2.7.8.5
3.1.3.27
LCLAT
CLD1
Cls
3.1.4.3
LPGAT
Ptdss1
LPSAT
4.1.1.65
2.7.8.29
3.1.1.32
2.1.1.71
2.1.1.17
2.3.1.43
3.1.1.4
LPCAT
2.3.1.62
2.7.8.2
3.1.1.5
3.1.4.2
3.1.4.46
2.7.7.15
2.7.1.32
3.1.3.75
2.3.1.6
3.1.1.7
BTA1
BtaA
BtaB
2.1.1.103
3.1.4.13
2.7.8.4
2.7.7.14
2.7.1.82
4.3.1.7
4.3.3.-
2.7.8.1
LPEAT
2.3.1.40
00564 9/14/11
(c) Kanehisa Laboratories

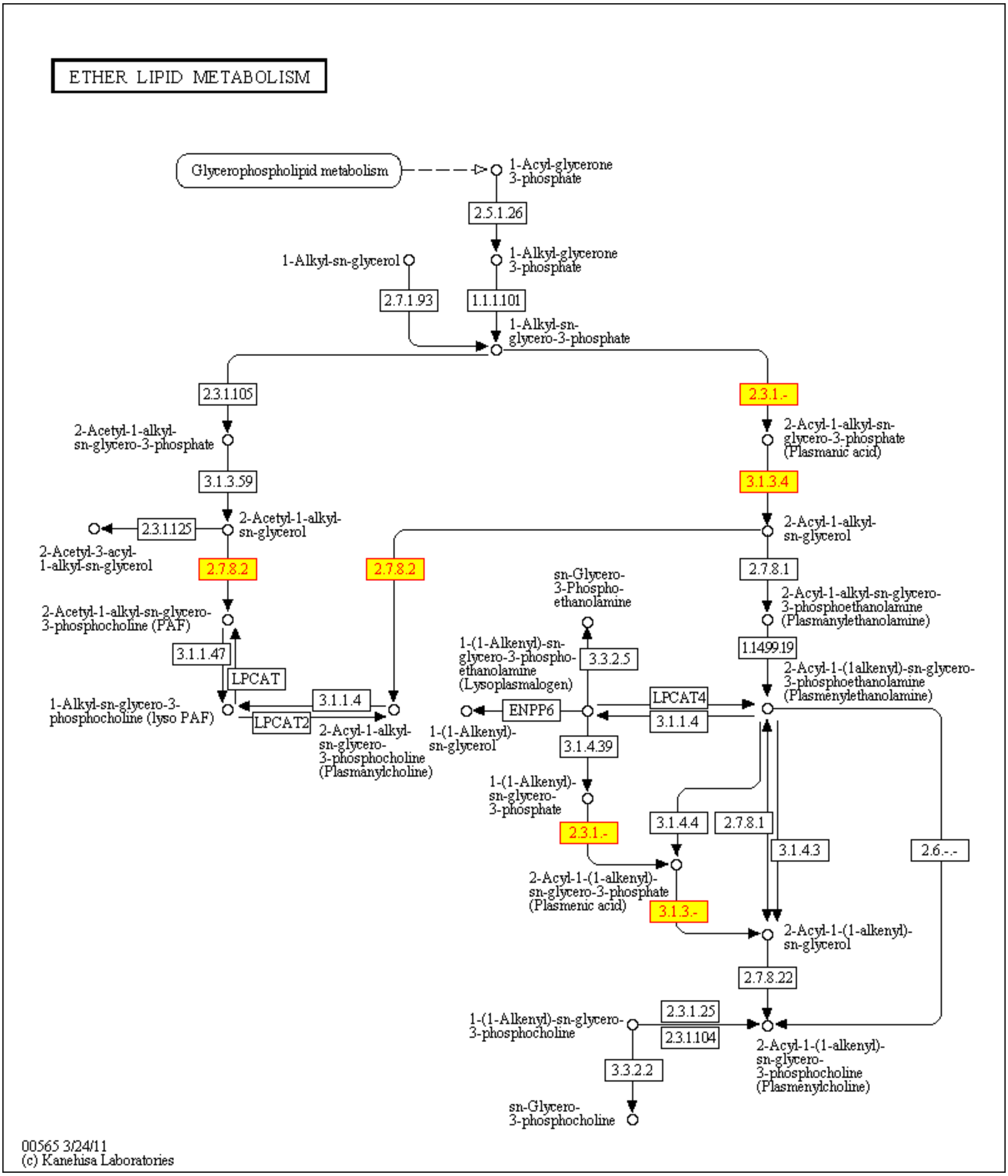

ETHER LIPID METABOLISM
Glycerophospholipid metabolism
1-Acyl-glycerone 3-phosphate
2.5.1.26
1-Alkyl-glycerone 3-phosphate
1-Alkyl-sn-glycerol
2.7.1.93
1.1.1.101
1-Alkyl-sn-glycero-3-phosphate
2.3.1.105
2.3.1.-
2-Acetyl-1-alkyl-sn-glycero-3-phosphate
2-Acyl-1-alkyl-sn-glycero-3-phosphate (Plasmanic acid)
3.1.3.59
3.1.3.4
2.3.1.125
2-Acetyl-1-alkyl-sn-glycerol
2-Acyl-1-alkyl-sn-glycerol
2-Acetyl-3-acyl-1-alkyl-sn-glycerol
2.7.8.2
2.7.8.2
2.7.8.1
sn-Glycero-3-Phospho-ethanolamine
2-Acyl-1-alkyl-sn-glycero-3-phosphoethanolamine (Plasmanylethanolamine)
2-Acetyl-1-alkyl-sn-glycero-3-phosphocholine (PAF)
1-(1-Alkenyl)-sn-glycero-3-phospho-ethanolamine (Lysoplasmalogen)
3.3.2.5
1.14.99.19
3.1.1.47
LPCAT
2-Acyl-1-(1alkenyl)-sn-glycero-3-phosphoethanolamine (Plasmenylethanolamine)
3.1.1.4
LPCAT4
1-Alkyl-sn-glycero-3-phosphocholine (lyso PAF)
ENPP6
3.1.1.4
LPCAT2
2-Acyl-1-alkyl-sn-glycero-3-phosphocholine (Plasmanylcholine)
1-(1-Alkenyl)-sn-glycerol
3.1.4.39
1-(1-Alkenyl)-sn-glycero-3-phosphate
3.1.4.4
2.7.8.1
2.3.1.-
3.1.4.3
2.6.-.-
2-Acyl-1-(1-alkenyl)-sn-glycero-3-phosphate (Plasmenic acid)
3.1.3.-
2-Acyl-1-(1-alkenyl)-sn-glycerol
2.7.8.22
2.3.1.25
1-(1-Alkenyl)-sn-glycero-3-phosphocholine
2.3.1.104
2-Acyl-1-(1-alkenyl)-sn-glycero-3-phosphocholine (Plasmenylcholine)
3.3.2.2
sn-Glycero-3-phosphocholine
00565 3/24/11
(c) Kanehisa Laboratories

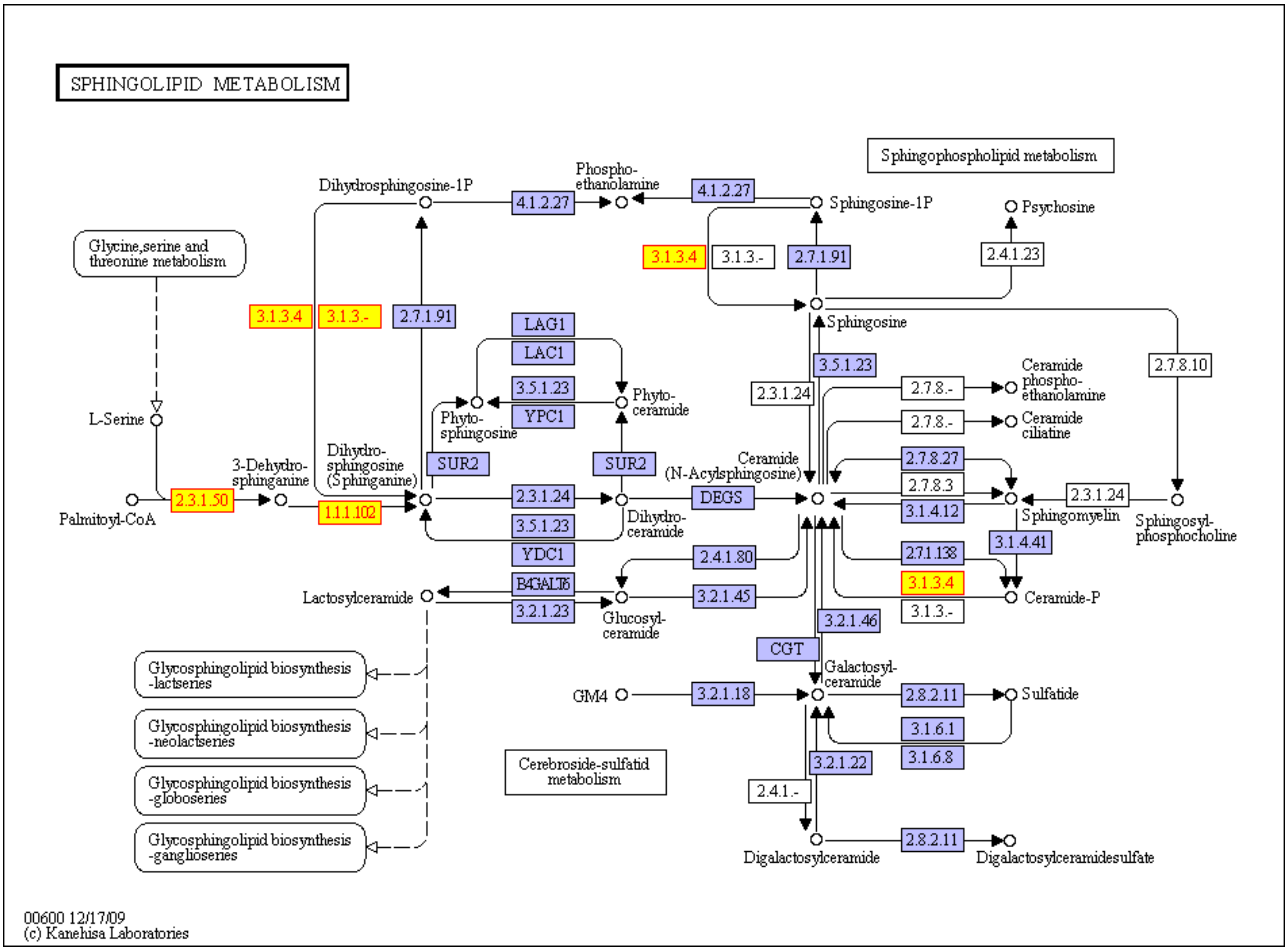
SPHINGOLIPID METABOLISM
Sphingophospholipid metabolism
Dihydrosphingosine-1P
4.1.2.27
Phospho-ethanolamine
4.1.2.27
Sphingosine-1P
Psychosine
Glycine,serine and threonine metabolism
3.1.3.4
3.1.3.-
2.7.1.91
2.4.1.23
3.1.3.4
3.1.3.-
2.7.1.91
Sphingosine
LAG1
LAC1
3.5.1.23
2.7.8.10
3.5.1.23
2.3.1.24
2.7.8.-
Ceramide phospho-ethanolamine
L-Serine
YPC1
Phyto-ceramide
2.7.8.-
Ceramide ciliatine
Phyto-sphingosine
Dihydro-sphingosine (Sphinganine)
3-Dehydro-sphinganine
SUR2
SUR2
Ceramide (N-Acylsphingosine)
2.7.8.27
2.7.8.3
2.3.1.24
Palmitoyl-CoA
2.3.1.50
1.1.1.102
2.3.1.24
DEGS
3.1.4.12
Sphingomyelin
Sphingosyl-phosphocholine
3.5.1.23
Dihydro-ceramide
3.1.4.41
YDC1
2.4.1.80
2.7.1.138
Lactosylceramide
B4GALT6
3.2.1.45
3.1.3.4
Ceramide-P
3.2.1.23
Glucosyl-ceramide
3.2.1.46
3.1.3.-
CGT
Glycosphingolipid biosynthesis -lactseries
Galactosyl-ceramide
GM4
3.2.1.18
2.8.2.11
Sulfatide
Glycosphingolipid biosynthesis -neolactseries
3.1.6.1
Cerebroside-sulfatid metabolism
3.2.1.22
3.1.6.8
Glycosphingolipid biosynthesis -globoseries
2.4.1.-
Glycosphingolipid biosynthesis -ganglioseries
Digalactosylceramide
2.8.2.11
Digalactosylceramidesulfate
00600 12/17/09
(c) Kanehisa Laboratories

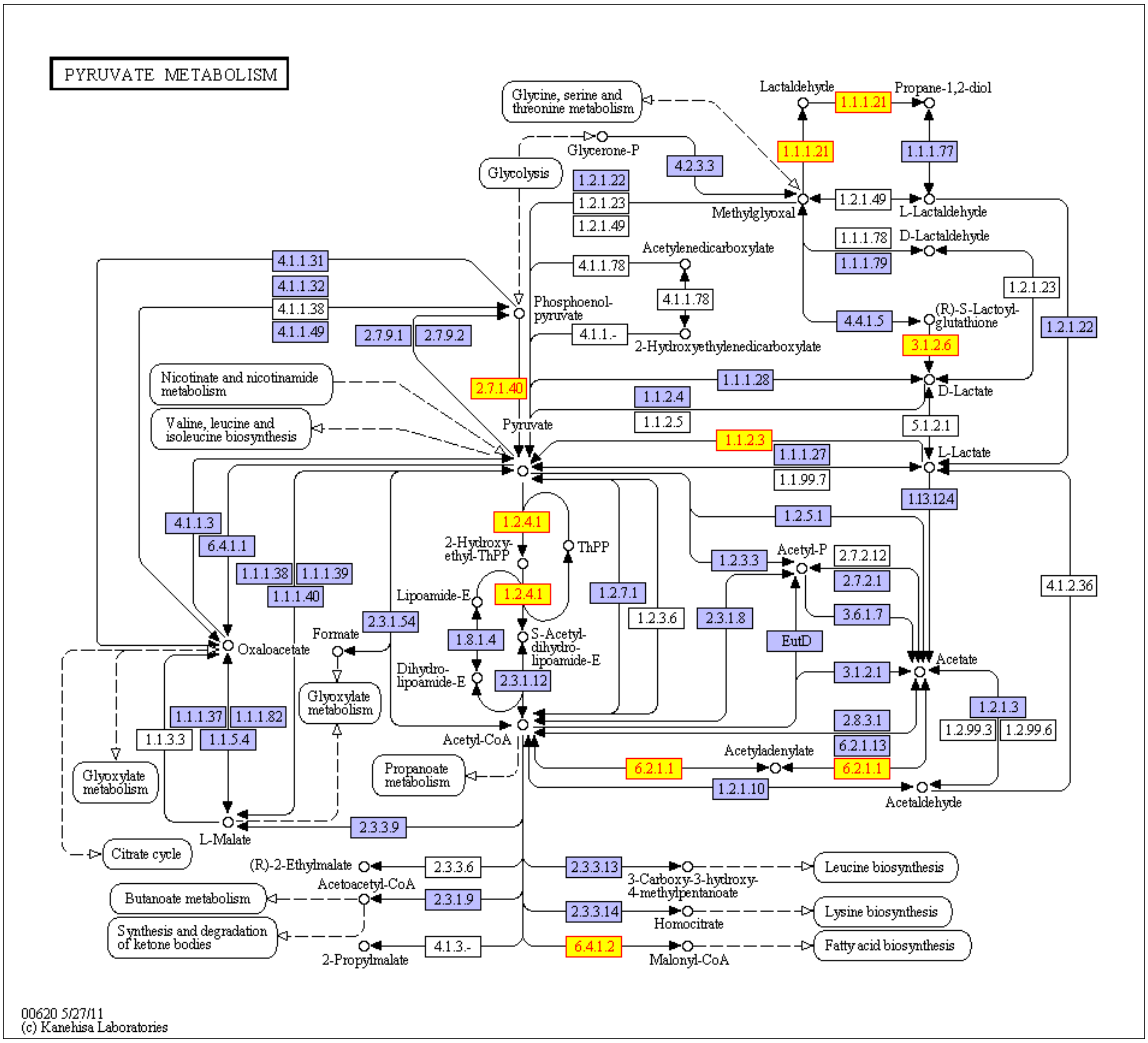
PYRUVATE METABOLISM
Glycine, serine and threonine metabolism
Lactaldehyde
1.1.1.21
Propane-1,2-diol
Glycerone-P
Glycolysis
4.2.3.3
1.1.1.21
1.1.1.77
1.2.1.22
1.2.1.23
1.2.1.49
Methylglyoxal
1.2.1.49
L-Lactaldehyde
1.1.1.78
D-Lactaldehyde
1.1.1.79
4.1.1.31
4.1.1.32
4.1.1.38
4.1.1.49
4.1.1.78
Acetylenedicarboxylate
4.1.1.78
Phosphoenol-pyruvate
4.1.1.-
2-Hydroxyethylenedicarboxylate
1.2.1.23
4.4.1.5
(R)-S-Lactoyl-glutathione
1.2.1.22
3.1.2.6
2.7.9.1
2.7.9.2
Nicotinate and nicotinamide metabolism
Valine, leucine and isoleucine biosynthesis
2.7.1.40
1.1.1.28
D-Lactate
1.1.2.4
1.1.2.5
5.1.2.1
Pyruvate
1.1.2.3
1.1.1.27
L-Lactate
1.1.99.7
1.13.124
4.1.1.3
6.4.1.1
1.2.4.1
2-Hydroxy-ethyl-ThPP
ThPP
1.2.5.1
1.1.1.38
1.1.1.39
1.1.1.40
1.2.3.3
Acetyl-P
2.7.2.12
2.7.2.1
4.1.2.36
Lipoamide-E
1.2.4.1
1.2.7.1
1.2.3.6
2.3.1.8
3.6.1.7
2.3.1.54
Formate
S-Acetyl-dihydro-lipoamide-E
EutD
Oxaloacetate
1.8.1.4
Acetate
Dihydro-lipoamide-E
2.3.1.12
3.1.2.1
Glyoxylate metabolism
1.2.1.3
1.2.99.3
1.2.99.6
1.1.1.37
1.1.1.82
1.1.3.3
1.1.5.4
2.8.3.1
Acetyl-CoA
6.2.1.13
Glyoxylate metabolism
Propanoate metabolism
Acetyladenylate
6.2.1.1
6.2.1.1
1.2.1.10
Acetaldehyde
L-Malate
2.3.3.9
Citrate cycle
(R)-2-Ethylmalate
2.3.3.6
2.3.3.13
3-Carboxy-3-hydroxy-4-methylpentanoate
Leucine biosynthesis
Butanoate metabolism
Acetoacetyl-CoA
2.3.1.9
2.3.3.14
Homocitrate
Lysine biosynthesis
Synthesis and degradation of ketone bodies
2-Propylmalate
4.1.3.-
6.4.1.2
Malonyl-CoA
Fatty acid biosynthesis
00620 5/27/11
(c) Kanehisa Laboratories

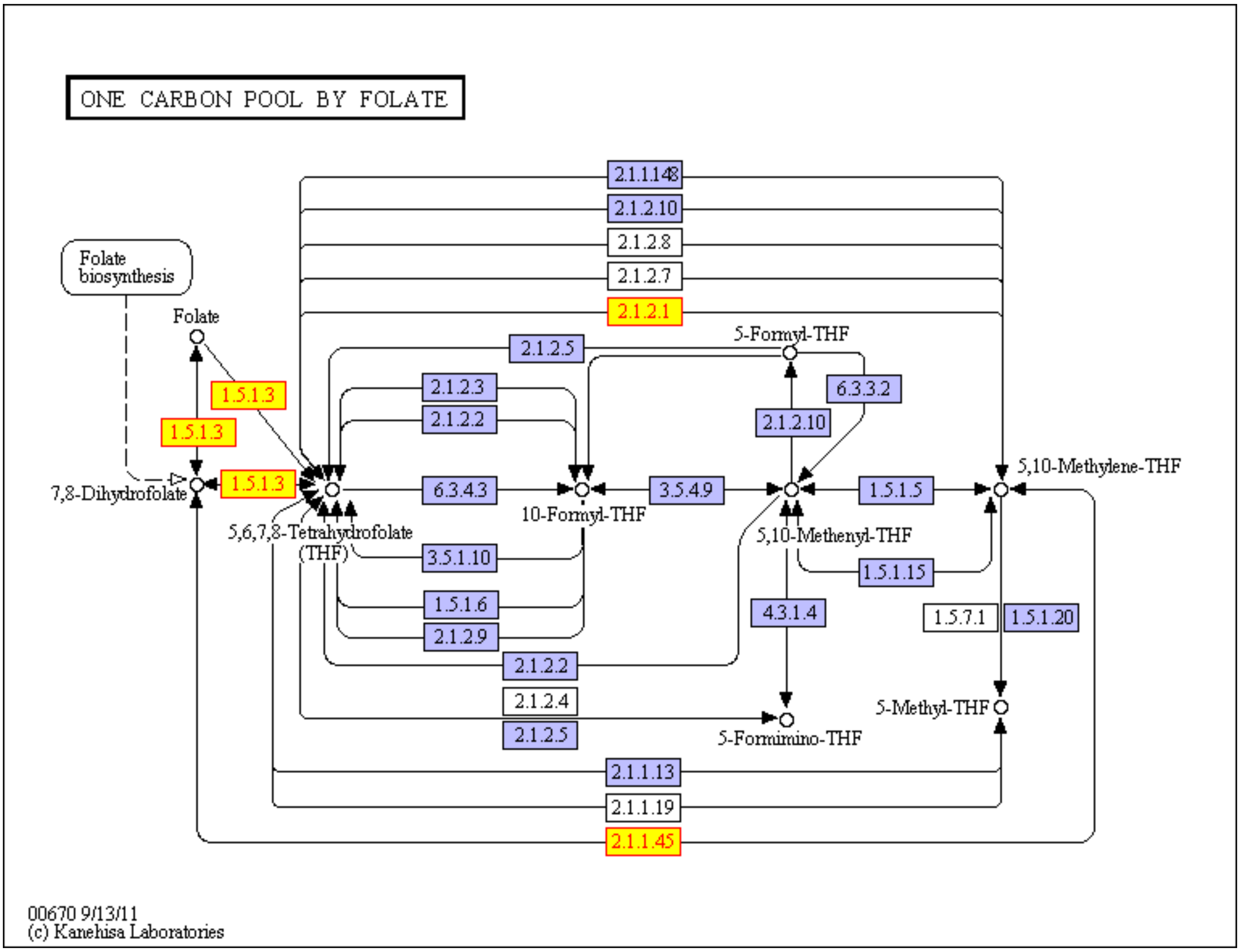

ONE CARBON POOL BY FOLATE
2.1.1.148
2.1.2.10
2.1.2.8
2.1.2.7
2.1.2.1
Folate biosynthesis
Folate
1.5.1.3
1.5.1.3
1.5.1.3
7,8-Dihydrofolate
2.1.2.5
5-Formyl-THF
2.1.2.3
2.1.2.2
6.3.3.2
2.1.2.10
6.3.4.3
3.5.4.9
1.5.1.5
5,10-Methylene-THF
10-Formyl-THF
5,6,7,8-Tetrahydrofolate (THF)
5,10-Methenyl-THF
3.5.1.10
1.5.1.15
1.5.1.6
4.3.1.4
1.5.7.1
1.5.1.20
2.1.2.9
2.1.2.2
2.1.2.4
5-Methyl-THF
2.1.2.5
5-Formimino-THF
2.1.1.13
2.1.1.19
2.1.1.45
00670 9/13/11
(c) Kanehisa Laboratories

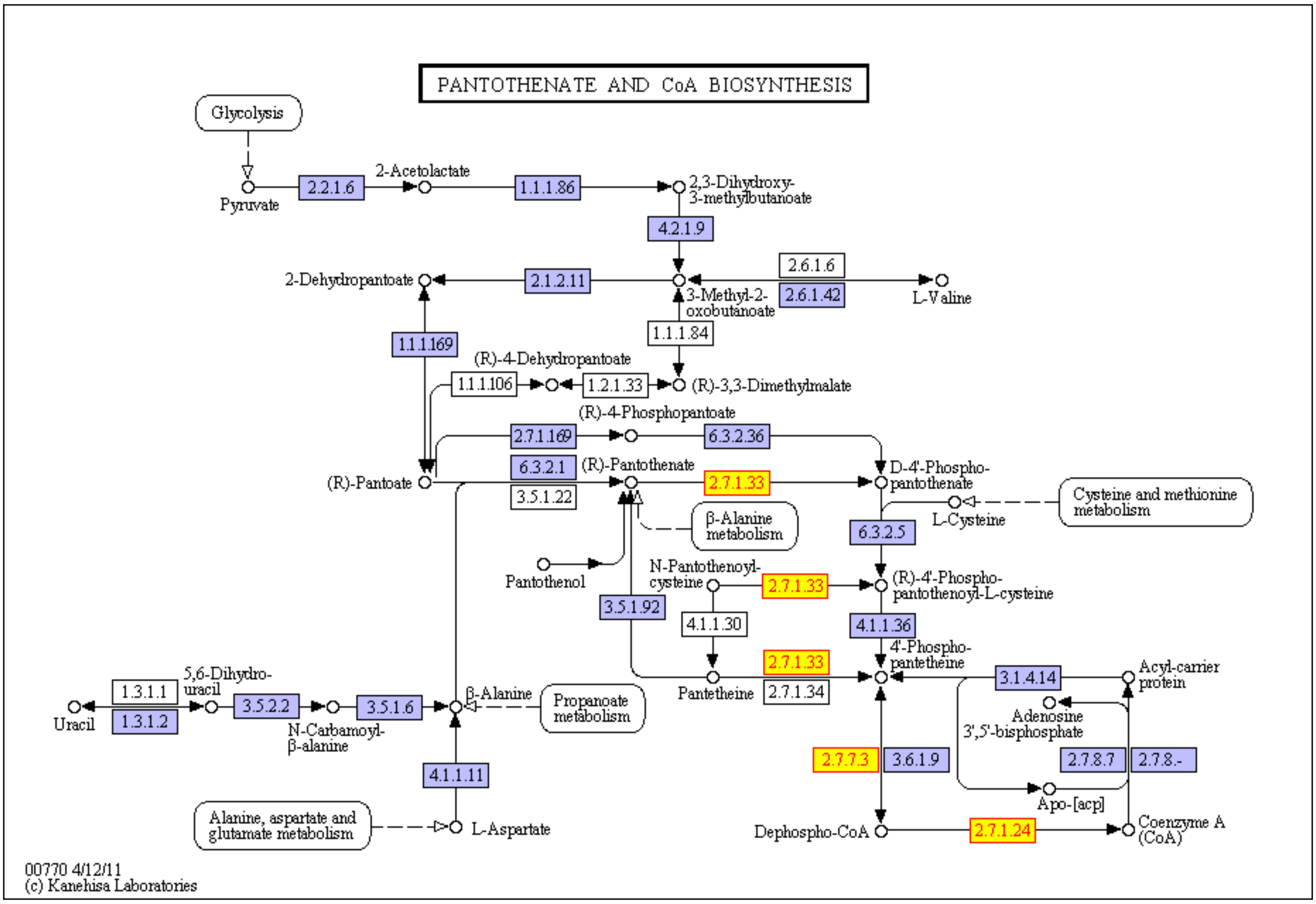
PANTOTHENATE AND CoA BIOSYNTHESIS
Glycolysis
Pyruvate
2.2.1.6
2-Acetolactate
1.1.1.86
2,3-Dihydroxy-3-methylbutanoate
4.2.1.9
2-Dehydropantoate
2.1.2.11
3-Methyl-2-oxobutanoate
2.6.1.6
2.6.1.42
L-Valine
1.1.1.169
1.1.1.84
(R)-4-Dehydropantoate
1.1.1.106
1.2.1.33
(R)-3,3-Dimethylmalate
(R)-4-Phosphopantoate
2.7.1.169
6.3.2.36
(R)-Pantoate
6.3.2.1
3.5.1.22
(R)-Pantothenate
2.7.1.33
D-4'-Phospho-pantothenate
Cysteine and methionine metabolism
L-Cysteine
β-Alanine metabolism
6.3.2.5
Pantothenol
N-Pantothenoyl-cysteine
2.7.1.33
(R)-4'-Phospho-pantothenoyl-L-cysteine
3.5.1.92
4.1.1.30
4.1.1.36
5,6-Dihydro-uracil
1.3.1.1
1.3.1.2
Uracil
3.5.2.2
N-Carbamoyl-β-alanine
3.5.1.6
β-Alanine
Propanoate metabolism
Pantetheine
2.7.1.33
2.7.1.34
4'-Phospho-pantetheine
3.1.4.14
Acyl-carrier protein
Adenosine 3',5'-bisphosphate
4.1.1.11
2.7.7.3
3.6.1.9
2.7.8.7
2.7.8.-
Apo-[acp]
Alanine, aspartate and glutamate metabolism
L-Aspartate
Dephospho-CoA
2.7.1.24
Coenzyme A (CoA)
00770 4/12/11
(c) Kanehisa Laboratories

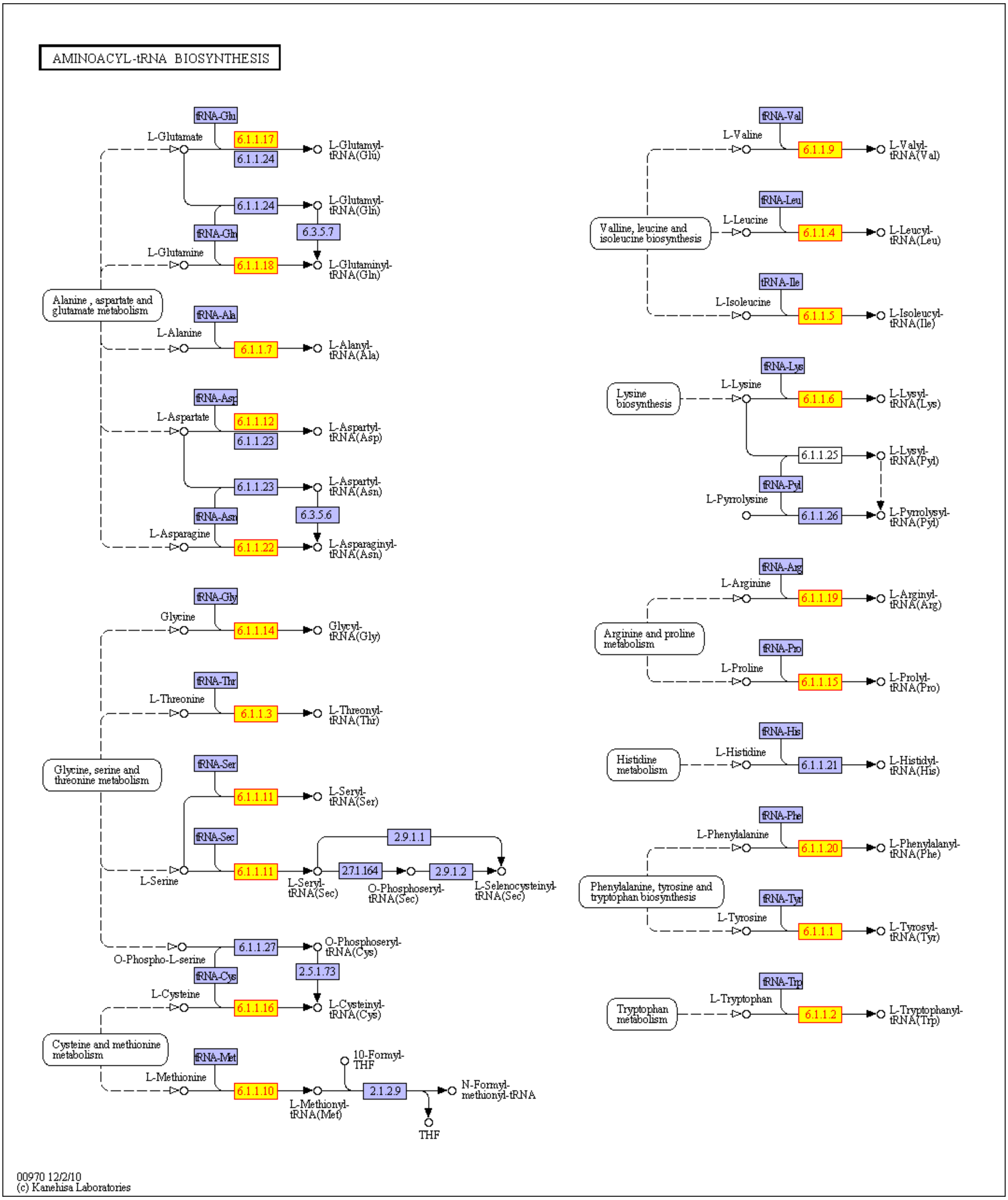

AMINOACYL-tRNA BIOSYNTHESIS
tRNA-Glu
L-Glutamate
6.1.1.17
6.1.1.24
L-Glutamyl-tRNA(Glu)
6.1.1.24
L-Glutamyl-tRNA(Gln)
6.3.5.7
tRNA-Gln
L-Glutamine
6.1.1.18
L-Glutaminyl-tRNA(Gln)
Alanine, aspartate and glutamate metabolism
tRNA-Ala
L-Alanine
6.1.1.7
L-Alanyl-tRNA(Ala)
tRNA-Asp
L-Aspartate
6.1.1.12
6.1.1.23
L-Aspartyl-tRNA(Asp)
6.1.1.23
L-Aspartyl-tRNA(Asn)
6.3.5.6
tRNA-Asn
L-Asparagine
6.1.1.22
L-Asparaginyl-tRNA(Asn)
tRNA-Gly
Glycine
6.1.1.14
Glycyl-tRNA(Gly)
tRNA-Thr
L-Threonine
6.1.1.3
L-Threonyl-tRNA(Thr)
Glycine, serine and threonine metabolism
tRNA-Ser
6.1.1.11
L-Seryl-tRNA(Ser)
tRNA-Sec
2.9.1.1
L-Serine
6.1.1.11
L-Seryl-tRNA(Sec)
2.7.1.164
O-Phosphoseryl-tRNA(Sec)
2.9.1.2
L-Selenocysteinyl-tRNA(Sec)
6.1.1.27
O-Phospho-L-serine
O-Phosphoseryl-tRNA(Cys)
tRNA-Cys
2.5.1.73
L-Cysteine
6.1.1.16
L-Cysteinyl-tRNA(Cys)
Cysteine and methionine metabolism
tRNA-Met
L-Methionine
6.1.1.10
10-Formyl-THF
2.1.2.9
N-Formyl-methionyl-tRNA
L-Methionyl-tRNA(Met)
THF
tRNA-Val
L-Valine
6.1.1.9
L-Valyl-tRNA(Val)
tRNA-Leu
Valine, leucine and isoleucine biosynthesis
L-Leucine
6.1.1.4
L-Leucyl-tRNA(Leu)
tRNA-Ile
L-Isoleucine
6.1.1.5
L-Isoleucyl-tRNA(Ile)
tRNA-Lys
Lysine biosynthesis
L-Lysine
6.1.1.6
L-Lysyl-tRNA(Lys)
6.1.1.25
L-Lysyl-tRNA(Pyl)
tRNA-Pyl
L-Pyrrolysine
6.1.1.26
L-Pyrrolysyl-tRNA(Pyl)
tRNA-Arg
L-Arginine
6.1.1.19
L-Arginyl-tRNA(Arg)
Arginine and proline metabolism
tRNA-Pro
L-Proline
6.1.1.15
L-Prolyl-tRNA(Pro)
tRNA-His
Histidine metabolism
L-Histidine
6.1.1.21
L-Histidyl-tRNA(His)
tRNA-Phe
L-Phenylalanine
6.1.1.20
L-Phenylalanyl-tRNA(Phe)
Phenylalanine, tyrosine and tryptophan biosynthesis
tRNA-Tyr
L-Tyrosine
6.1.1.1
L-Tyrosyl-tRNA(Tyr)
tRNA-Trp
Tryptophan metabolism
L-Tryptophan
6.1.1.2
L-Tryptophanyl-tRNA(Trp)
00970 12/2/10
(c) Kanehisa Laboratories

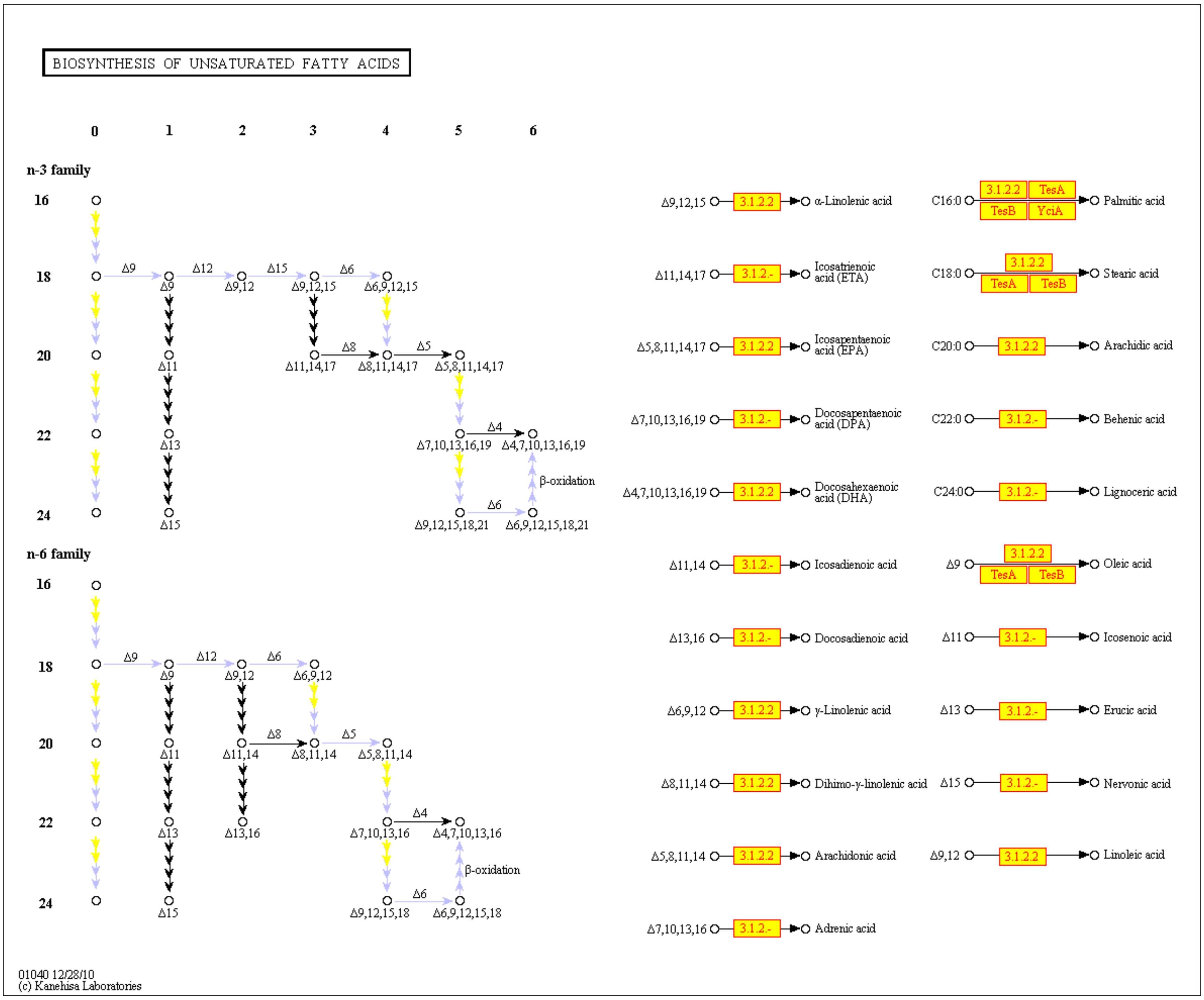
BIOSYNTHESIS OF UNSATURATED FATTY ACIDS
0
1
2
3
4
5
6
n-3 family
16
18
20
22
24
Δ9
Δ12
Δ15
Δ6
Δ9
Δ9,12
Δ9,12,15
Δ6,9,12,15
Δ8
Δ5
Δ11
Δ11,14,17
Δ8,11,14,17
Δ5,8,11,14,17
Δ4
Δ13
Δ7,10,13,16,19
Δ4,7,10,13,16,19
β-oxidation
Δ6
Δ15
Δ9,12,15,18,21
Δ6,9,12,15,18,21
n-6 family
16
18
20
22
24
Δ9
Δ12
Δ6
Δ9
Δ9,12
Δ6,9,12
Δ8
Δ5
Δ11
Δ11,14
Δ8,11,14
Δ5,8,11,14
Δ4
Δ13
Δ13,16
Δ7,10,13,16
Δ4,7,10,13,16
β-oxidation
Δ6
Δ15
Δ9,12,15,18
Δ6,9,12,15,18
Δ9,12,15 3.1.2.2 α-Linolenic acid
Δ11,14,17 3.1.2.- Icosatrienoic acid (ETA)
Δ5,8,11,14,17 3.1.2.2 Icosapentaenoic acid (EPA)
Δ7,10,13,16,19 3.1.2.- Docosapentaenoic acid (DPA)
Δ4,7,10,13,16,19 3.1.2.2 Docosahexaenoic acid (DHA)
Δ11,14 3.1.2.- Icosadienoic acid
Δ13,16 3.1.2.- Docosadienoic acid
Δ6,9,12 3.1.2.2 γ-Linolenic acid
Δ8,11,14 3.1.2.2 Dihimo-γ-linolenic acid
Δ5,8,11,14 3.1.2.2 Arachidonic acid
Δ7,10,13,16 3.1.2.- Adrenic acid
C16:0 3.1.2.2 TesA TesB YciA Palmitic acid
C18:0 3.1.2.2 TesA TesB Stearic acid
C20:0 3.1.2.2 Arachidic acid
C22:0 3.1.2.- Behenic acid
C24:0 3.1.2.- Lignoceric acid
Δ9 3.1.2.2 TesA TesB Oleic acid
Δ11 3.1.2.- Icosenoic acid
Δ13 3.1.2.- Erucic acid
Δ15 3.1.2.- Nervonic acid
Δ9,12 3.1.2.2 Linoleic acid
01040 12/28/10
(c) Kanehisa Laboratories

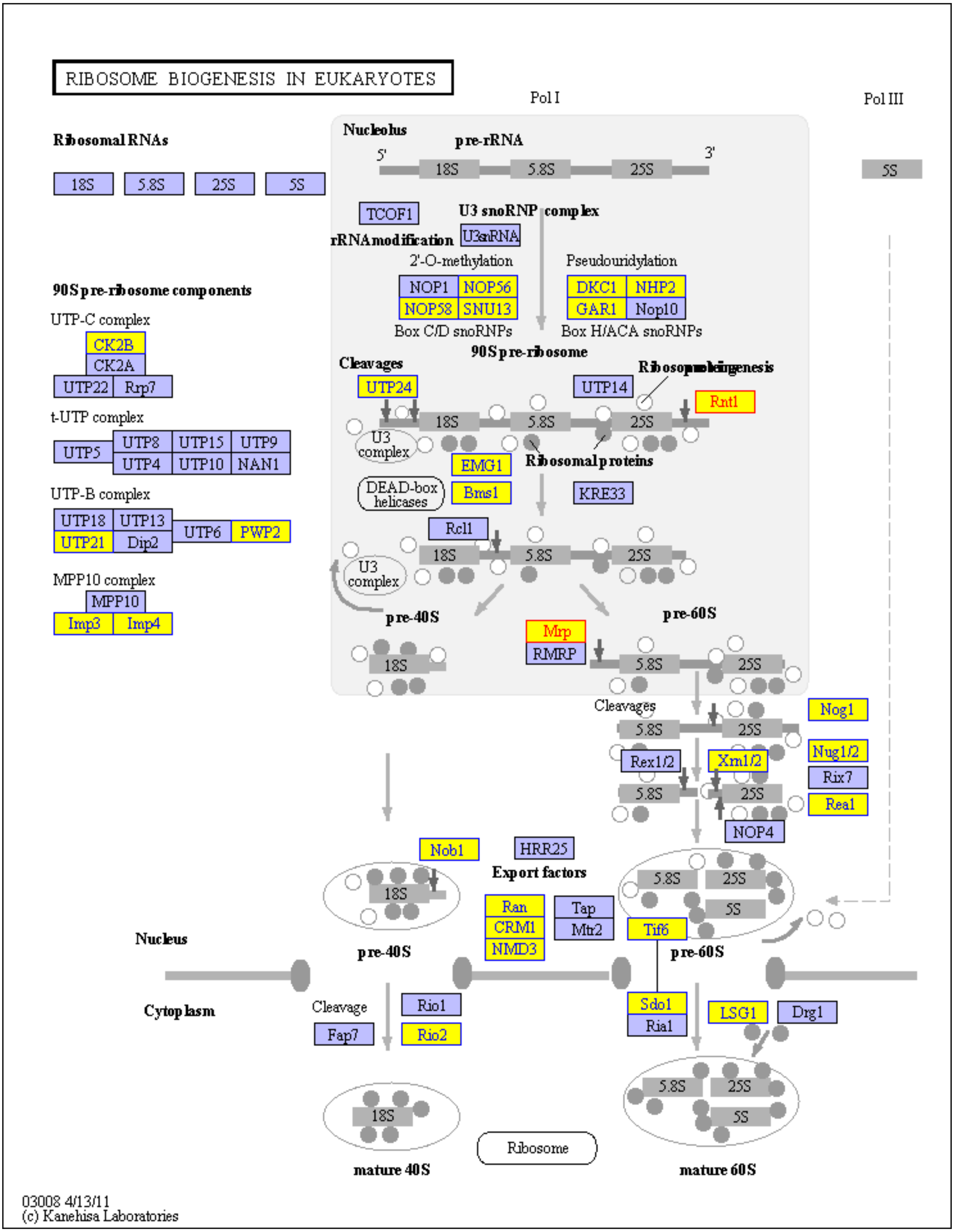
RIBOSOME BIOGENESIS IN EUKARYOTES
Pol I
Pol III
Ribosomal RNAs
18S
5.8S
25S
5S
Nucleolus
pre-rRNA
5'
3'
TCOF1
U3 snoRNP complex
U3snRNA
rRNA modification
2'-O-methylation
Pseudouridylation
NOP1
NOP56
NOP58
SNU13
Box C/D snoRNPs
DKC1
NHP2
GAR1
Nop10
Box H/ACA snoRNPs
90S pre-ribosome components
UTP-C complex
CK2B
CK2A
UTP22
Rrp7
t-UTP complex
UTP5
UTP8
UTP15
UTP9
UTP4
UTP10
NAN1
UTP-B complex
UTP18
UTP13
UTP21
Dip2
UTP6
PWP2
MPP10 complex
MPP10
Imp3
Imp4
90S pre-ribosome
Cleavages
UTP24
UTP14
Rnt1
U3 complex
EMG1
Ribosomal proteins
DEAD-box helicases
Bms1
KRE33
Rcl1
pre-40S
pre-60S
Mrp
RMRP
Nog1
Nug1/2
Rex1/2
Xrn1/2
Rix7
Rea1
NOP4
Nob1
HRR25
Export factors
Ran
CRM1
NMD3
Tap
Mtr2
Tif6
Nucleus
Cytoplasm
Cleavage
Rio1
Fap7
Rio2
Sdo1
Rial
LSG1
Drg1
mature 40S
mature 60S
Ribosome
03008 4/13/11
(c) Kanehisa Laboratories

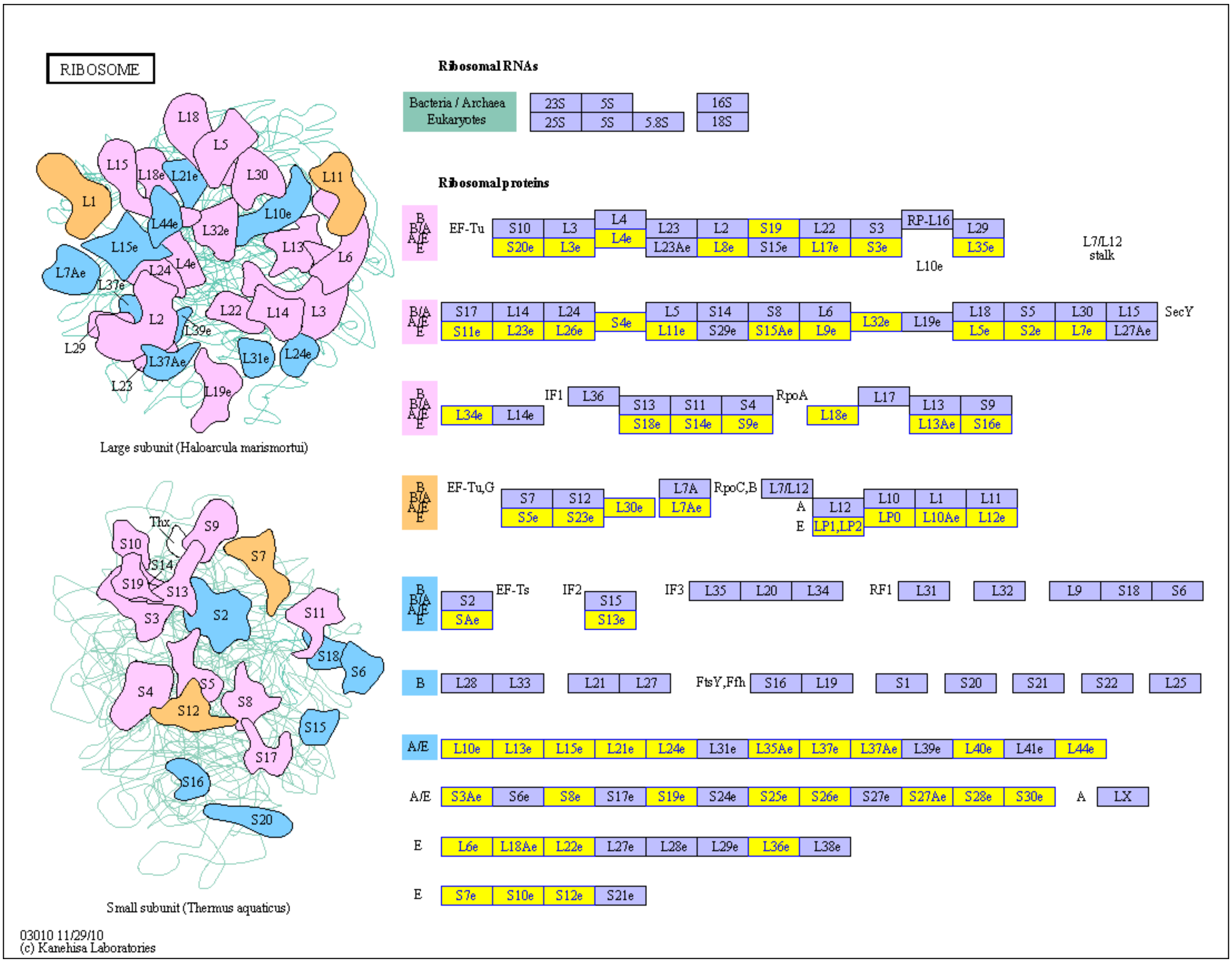

RIBOSOME
Ribosomal RNAs
Bacteria / Archaea
Eukaryotes
23S 5S 16S
25S 5S 5.8S 18S
Ribosomal proteins
B B/A A/E E
EF-Tu
S10 L3 L4 L23 L2 S19 L22 S3 RP-L16 L29
S20e L3e L4e L23Ae L8e S15e L17e S3e L35e
L10e
L7/L12 stalk
S17 L14 L24 S4e L5 S14 S8 L6 L32e L19e L18 S5 L30 L15 SecY
S11e L23e L26e L11e S29e S15Ae L9e L5e S2e L7e L27Ae
IF1 L36 S13 S11 S4 RpoA L17 L13 S9
L34e L14e S18e S14e S9e L18e L13Ae S16e
EF-Tu,G S7 S12 L30e L7A RpoC,B L7/L12 L12 L10 L1 L11
S5e S23e L7Ae LP1,LP2 LP0 L10Ae L12e
A
E
EF-Ts IF2 IF3 RF1
S2 S15 L35 L20 L34 L31 L32 L9 S18 S6
SAe S13e
L28 L33 L21 L27 FtsY,Ffh S16 L19 S1 S20 S21 S22 L25
A/E
L10e L13e L15e L21e L24e L31e L35Ae L37e L37Ae L39e L40e L41e L44e
S3Ae S6e S8e S17e S19e S24e S25e S26e S27e S27Ae S28e S30e
A LX
L6e L18Ae L22e L27e L28e L29e L36e L38e
S7e S10e S12e S21e
Large subunit (Haloarcula marismortui)
Small subunit (Thermus aquaticus)
03010 11/29/10
(c) Kanehisa Laboratories

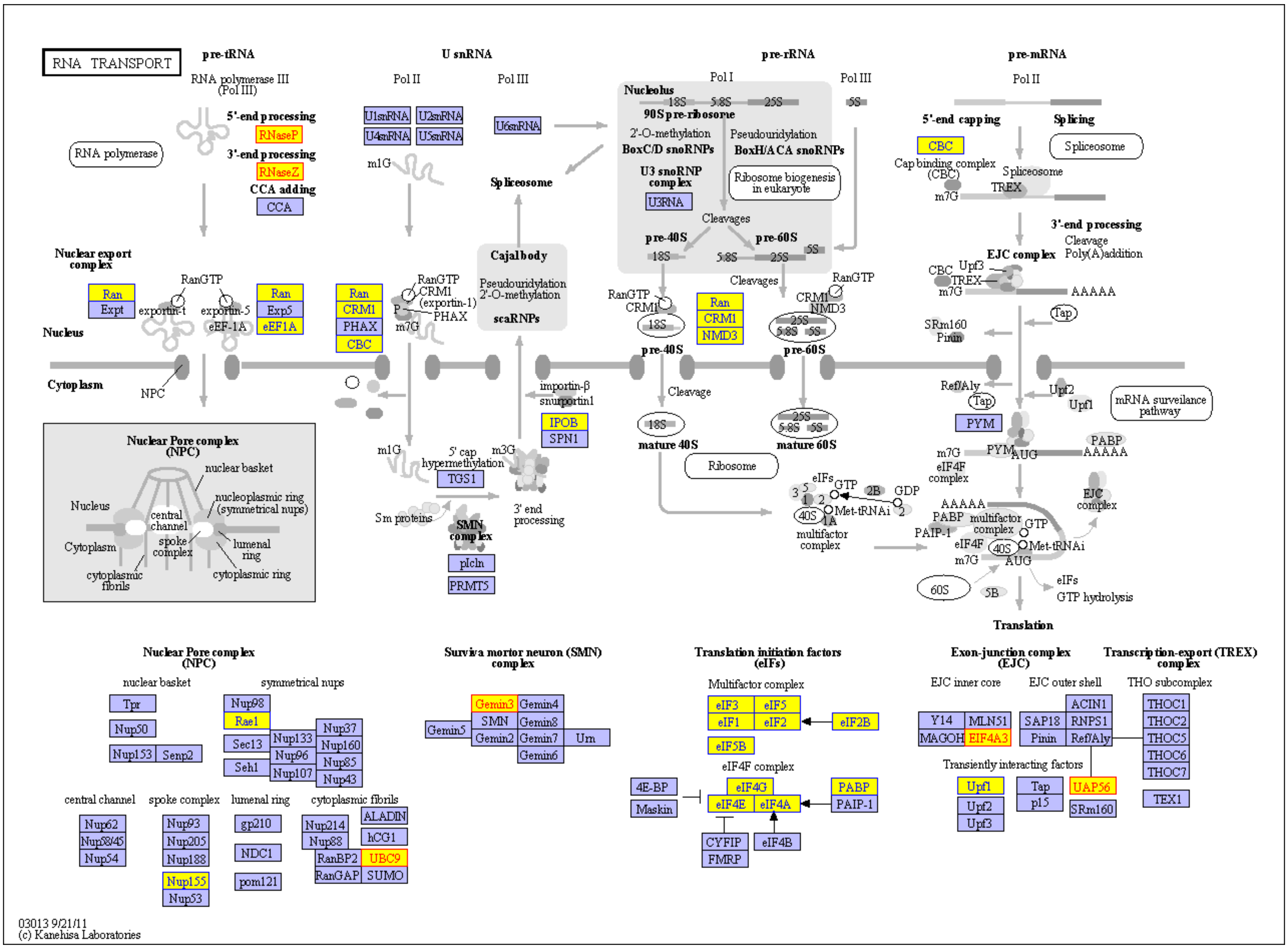

RNA TRANSPORT
pre-tRNA
U snRNA
pre-rRNA
pre-mRNA
Nuclear Pore complex (NPC)
Survival motor neuron (SMN) complex
Translation initiation factors (eIFs)
Exon-junction complex (EJC)
Transcription-export (TREX) complex
Nucleus
Cytoplasm
Nucleolus
Spliceosome
Cajal body
Translation
Ribosome
mRNA surveilance pathway
RNaseP
RNaseZ
CCA
Ran
CRM1
PHAX
CBC
IPOB
SPN1
TGS1
Gemin3
Gemin4
SMN
Gemin8
Gemin2
Gemin7
Gemin6
Gemin5
Unrip
NMD3
eIF3
eIF5
eIF1
eIF2
eIF2B
eIF5B
4E-BP
Maskin
eIF4G
eIF4E
eIF4A
PABP
PAIP-1
CYFIP
FMRP
eIF4B
Y14
MLN51
MAGOH
EIF4A3
ACIN1
SAP18
RNPS1
Pinin
Ref/Aly
Upf1
Upf2
Upf3
Tap
p15
UAP56
SRm160
THOC1
THOC2
THOC5
THOC6
THOC7
TEX1
Tpr
Nup50
Nup153
Senp2
Nup98
Rae1
Sec13
Seh1
Nup133
Nup160
Nup96
Nup107
Nup37
Nup85
Nup43
Nup62
Nup58/45
Nup54
Nup93
Nup205
Nup188
Nup155
Nup53
gp210
NDC1
pom121
Nup214
Nup88
ALADIN
hCG1
RanBP2
UBC9
RanGAP
SUMO
03013 9/21/11
(c) Kanehisa Laboratories

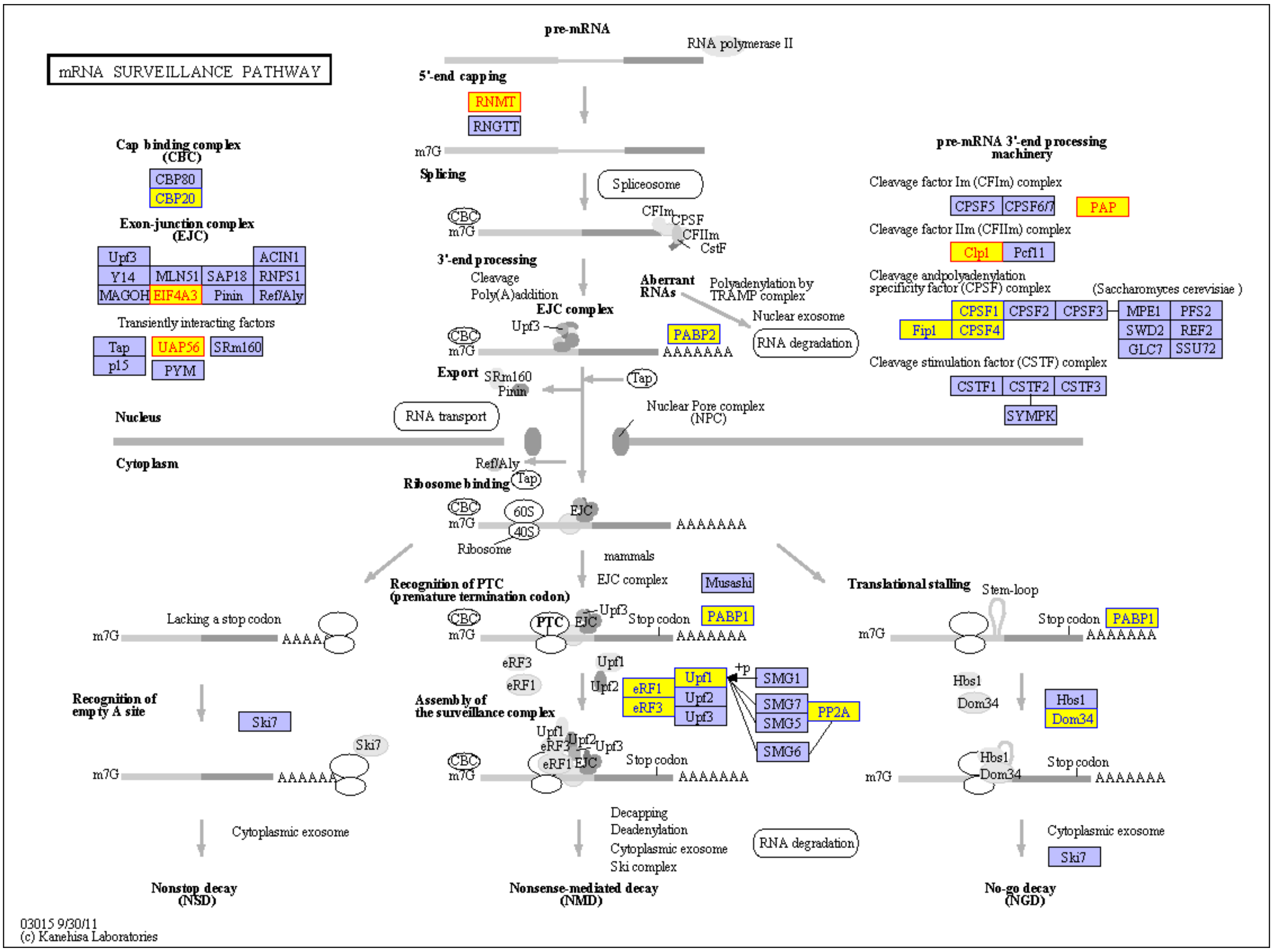
mRNA SURVEILLANCE PATHWAY
pre-mRNA
RNA polymerase II
5'-end capping
RNMT
RNGTT
m7G
Splicing
Spliceosome
Cap binding complex (CBC)
CBP80
CBP20
Exon-junction complex (EJC)
Upf3
ACIN1
Y14
MLN51
SAP18
RNPS1
MAGOH
EIF4A3
Pinin
Ref/Aly
Transiently interacting factors
Tap
UAP56
SRm160
p15
PYM
CBC
CFIm
CPSF
CFIIm
CstF
3'-end processing
Cleavage
Poly(A)addition
EJC complex
Upf3
PABP2
AAAAAAA
Aberrant RNAs
Polyadenylation by TRAMP complex
Nuclear exosome
RNA degradation
Export
SRm160
Pinin
Tap
pre-mRNA 3'-end processing machinery
Cleavage factor Im (CFIm) complex
CPSF5
CPSF6/7
PAP
Cleavage factor IIm (CFIIm) complex
Clp1
Pcf11
Cleavage and polyadenylation specificity factor (CPSF) complex
(Saccharomyces cerevisiae)
CPSF1
CPSF2
CPSF3
MPE1
PFS2
Fip1
CPSF4
SWD2
REF2
GLC7
SSU72
Cleavage stimulation factor (CSTF) complex
CSTF1
CSTF2
CSTF3
SYMPK
Nucleus
RNA transport
Nuclear Pore complex (NPC)
Cytoplasm
Ref/Aly
Ribosome binding
Tap
60S
40S
EJC
Ribosome
AAAAAAA
mammals
EJC complex
Musashi
Recognition of PTC (premature termination codon)
Translational stalling
Stem-loop
Lacking a stop codon
AAAA
PTC
Upf3
Stop codon
PABP1
Stop codon
PABP1
eRF3
Upf1
eRF1
Upf2
+p
eRF1
Upf1
SMG1
eRF3
Upf2
SMG7
PP2A
Upf3
SMG5
SMG6
Hbs1
Dom34
Hbs1
Dom34
Recognition of empty A site
Ski7
Assembly of the surveillance complex
Ski7
AAAAAA
Upf1
eRF3
Upf2
Upf3
eRF1
Stop codon
Hbs1
Dom34
Stop codon
Cytoplasmic exosome
Decapping
Deadenylation
Cytoplasmic exosome
Ski complex
RNA degradation
Cytoplasmic exosome
Ski7
Nonstop decay (NSD)
Nonsense-mediated decay (NMD)
No-go decay (NGD)
03015 9/30/11
(c) Kanehisa Laboratories

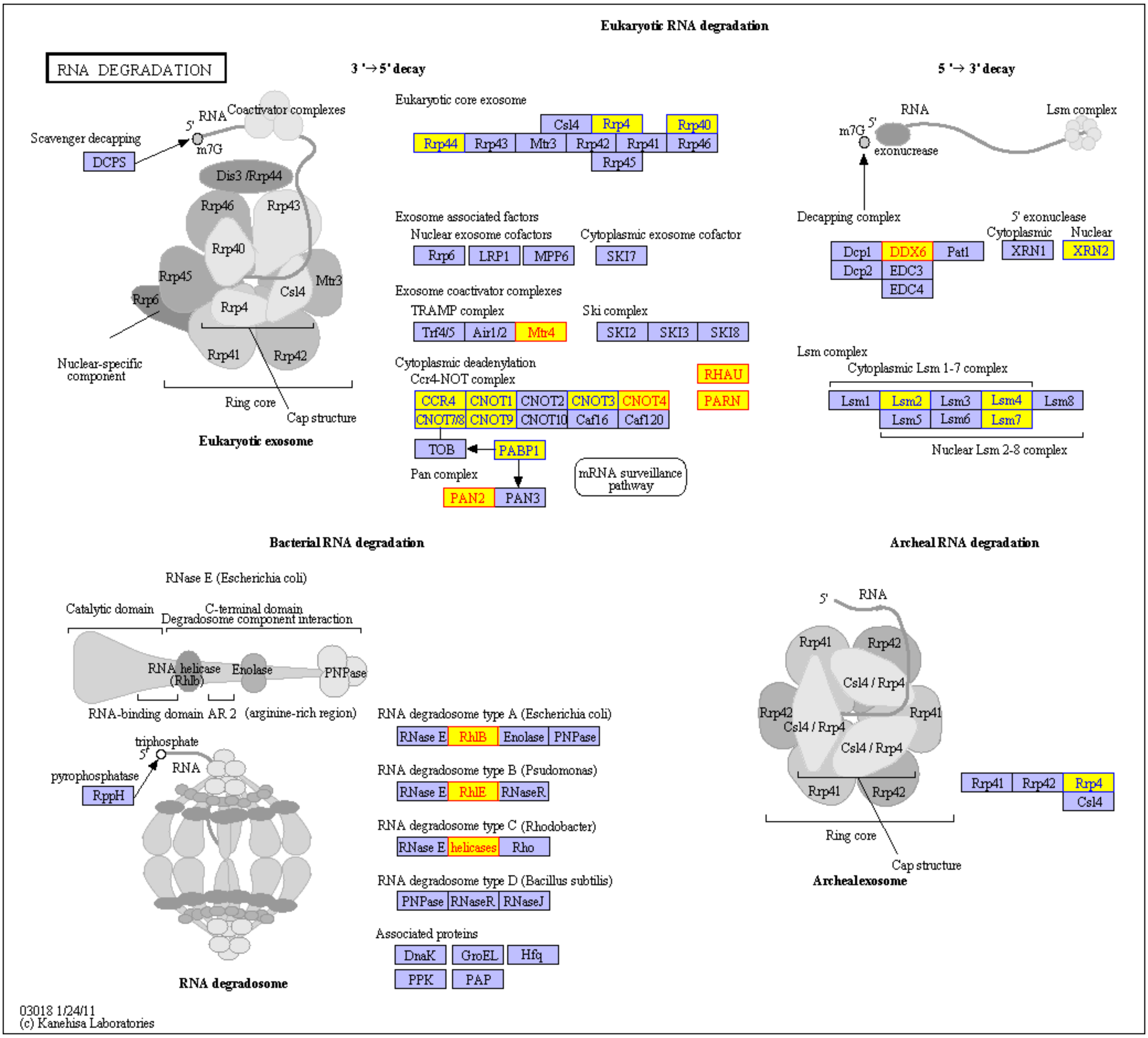
Eukaryotic RNA degradation
RNA DEGRADATION
3 '→ 5' decay
5 '→ 3' decay
Coactivator complexes
RNA
5'
Scavenger decapping
m7G
DCPS
Dis3 /Rrp44
Rrp46
Rrp43
Rrp40
Rrp45
Mtr3
Csl4
Rrp6
Rrp4
Nuclear-specific component
Rrp41
Rrp42
Ring core
Cap structure
Eukaryotic exosome
Eukaryotic core exosome
Csl4
Rrp4
Rrp40
Rrp44
Rrp43
Mtr3
Rrp42
Rrp41
Rrp46
Rrp45
Exosome associated factors
Nuclear exosome cofactors
Cytoplasmic exosome cofactor
Rrp6
LRP1
MPP6
SKI7
Exosome coactivator complexes
TRAMP complex
Ski complex
Trf4/5
Air1/2
Mtr4
SKI2
SKI3
SKI8
Cytoplasmic deadenylation
Ccr4-NOT complex
RHAU
PARN
CCR4
CNOT1
CNOT2
CNOT3
CNOT4
CNOT7/8
CNOT9
CNOT10
Caf16
Caf120
TOB
PABP1
Pan complex
mRNA surveillance pathway
PAN2
PAN3
RNA
Lsm complex
m7G
5'
exonucrease
Decapping complex
5' exonuclease
Cytoplasmic
Nuclear
Dcp1
DDX6
Pat1
XRN1
XRN2
Dcp2
EDC3
EDC4
Lsm complex
Cytoplasmic Lsm 1-7 complex
Lsm1
Lsm2
Lsm3
Lsm4
Lsm8
Lsm5
Lsm6
Lsm7
Nuclear Lsm 2-8 complex
Bacterial RNA degradation
Archeal RNA degradation
RNase E (Escherichia coli)
Catalytic domain
C-terminal domain
Degradosome component interaction
RNA helicase (Rhlb)
Enolase
PNPase
RNA-binding domain AR 2 (arginine-rich region)
triphosphate
5'
pyrophosphatase
RNA
RppH
RNA degradosome
RNA degradosome type A (Escherichia coli)
RNase E
RhlB
Enolase
PNPase
RNA degradosome type B (Psudomonas)
RNase E
RhlE
RNaseR
RNA degradosome type C (Rhodobacter)
RNase E
helicases
Rho
RNA degradosome type D (Bacillus subtilis)
PNPase
RNaseR
RNaseJ
Associated proteins
DnaK
GroEL
Hfq
PPK
PAP
5'
RNA
Rrp41
Rrp42
Csl4 / Rrp4
Rrp42
Rrp41
Csl4 / Rrp4
Csl4 / Rrp4
Rrp41
Rrp42
Ring core
Cap structure
Archealexosome
Rrp41
Rrp42
Rrp4
Csl4
03018 1/24/11
(c) Kanehisa Laboratories

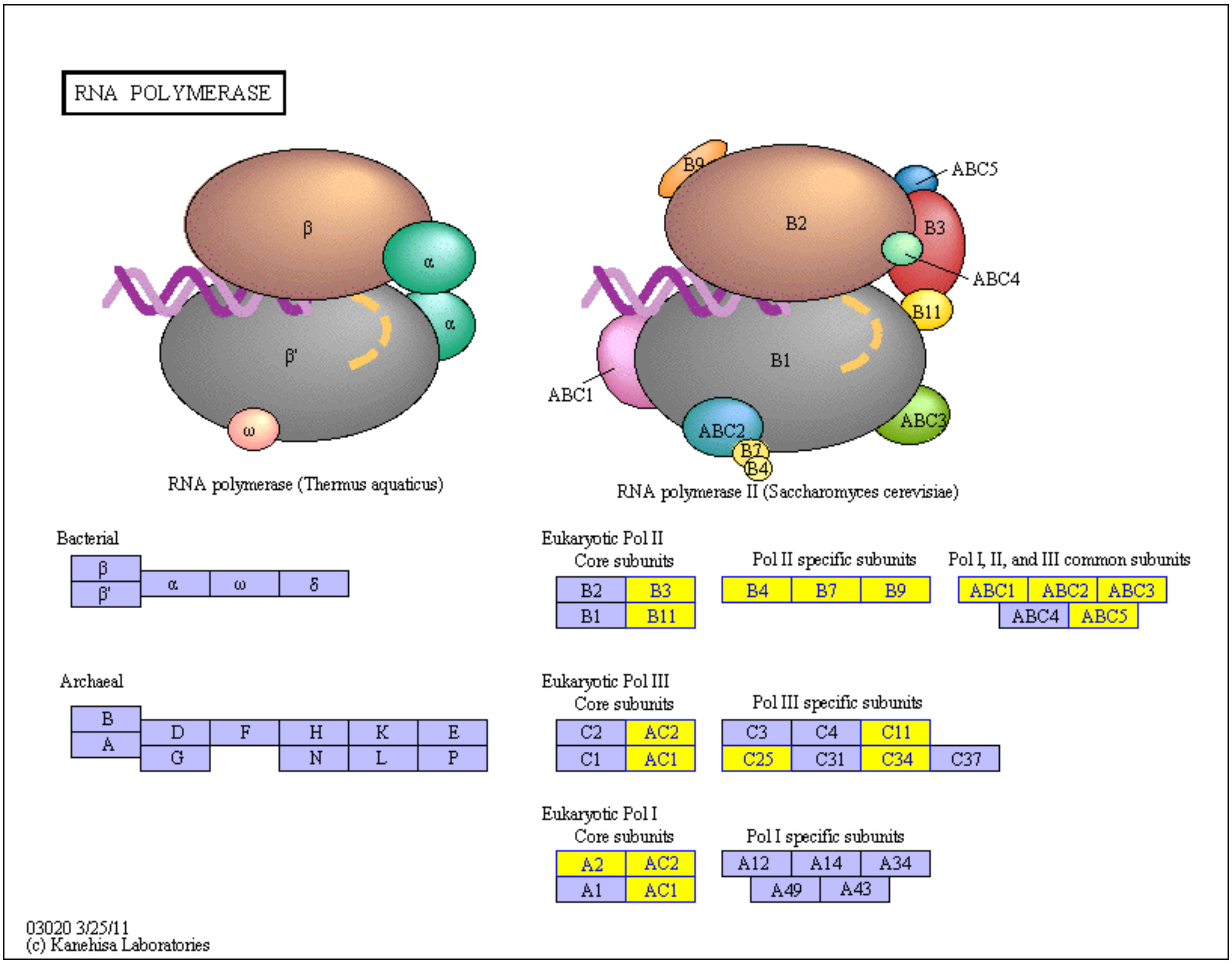
RNA POLYMERASE
β
α
α
β'
ω
RNA polymerase (Thermus aquaticus)
B9
ABC5
B2
B3
ABC4
B11
B1
ABC1
ABC2
ABC3
B7
B4
RNA polymerase II (Saccharomyces cerevisiae)
Bacterial
β
β'
α
ω
δ
Archaeal
B
A
D
G
F
H
N
K
L
E
P
Eukaryotic Pol II
Core subunits
B2
B3
B1
B11
Pol II specific subunits
B4
B7
B9
Pol I, II, and III common subunits
ABC1
ABC2
ABC3
ABC4
ABC5
Eukaryotic Pol III
Core subunits
C2
AC2
C1
AC1
Pol III specific subunits
C3
C4
C11
C25
C31
C34
C37
Eukaryotic Pol I
Core subunits
A2
AC2
A1
AC1
Pol I specific subunits
A12
A14
A34
A49
A43
03020 3/25/11
(c) Kanehisa Laboratories

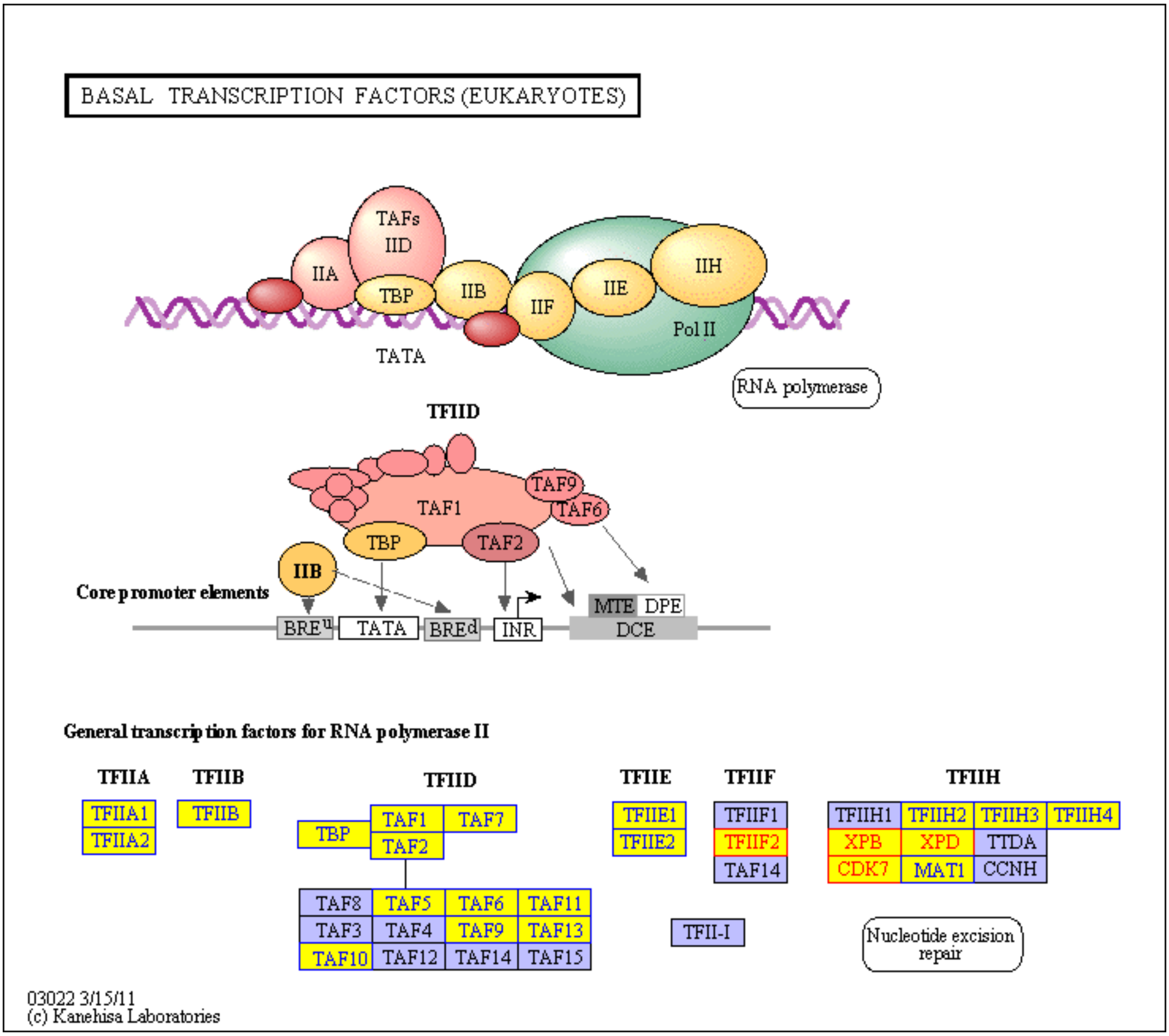
BASAL TRANSCRIPTION FACTORS (EUKARYOTES)
TAFs IID
IIA
TBP
IIB
IIF
IIE
IIH
Pol II
TATA
RNA polymerase
TFIID
TAF9
TAF1
TAF6
TBP
TAF2
IIB
Core promoter elements
BREu
TATA
BREd
INR
MTE
DPE
DCE
General transcription factors for RNA polymerase II
TFIIA
TFIIA1
TFIIA2
TFIIB
TFIIB
TFIID
TBP
TAF1
TAF7
TAF2
TAF8
TAF5
TAF6
TAF11
TAF3
TAF4
TAF9
TAF13
TAF10
TAF12
TAF14
TAF15
TFIIE
TFIIE1
TFIIE2
TFIIF
TFIIF1
TFIIF2
TAF14
TFII-I
TFIIH
TFIIH1
TFIIH2
TFIIH3
TFIIH4
XPB
XPD
TTDA
CDK7
MAT1
CCNH
Nucleotide excision repair
03022 3/15/11
(c) Kanehisa Laboratories

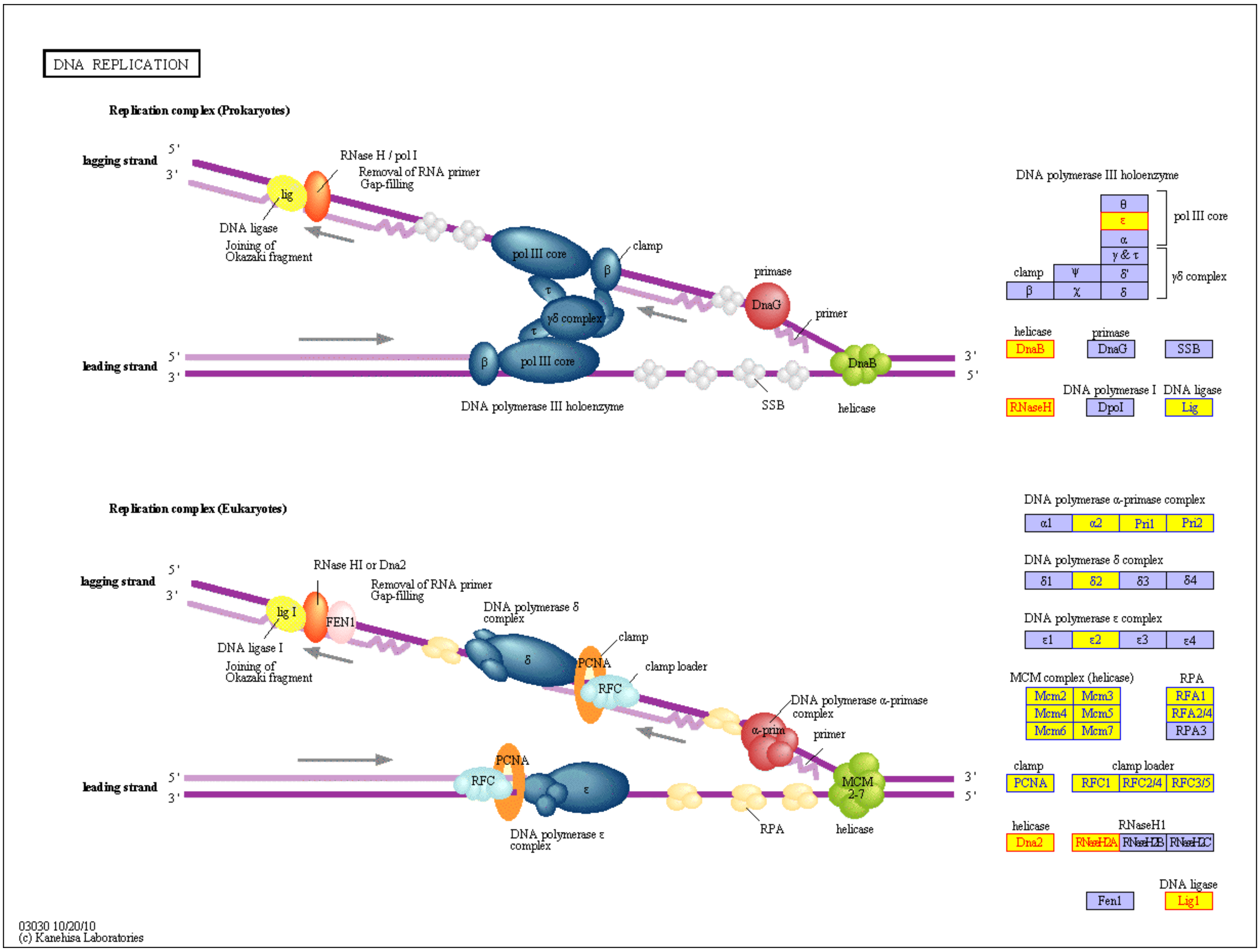

DNA REPLICATION
Replication complex (Prokaryotes)
lagging strand
leading strand
5'
3'
RNase H / pol I
Removal of RNA primer
Gap-filling
lig
DNA ligase
Joining of
Okazaki fragment
pol III core
clamp
β
τ
γδ complex
primase
DnaG
primer
DnaB
DNA polymerase III holoenzyme
SSB
helicase
DNA polymerase III holoenzyme
θ
ε
α
γ & τ
δ'
δ
ψ
χ
pol III core
γδ complex
helicase
primase
DnaB
SSB
DNA polymerase I
DNA ligase
RNaseH
DpoI
Lig
Replication complex (Eukaryotes)
RNase HI or Dna2
Removal of RNA primer
Gap-filling
lig I
FEN1
DNA ligase I
DNA polymerase δ
complex
PCNA
RFC
clamp loader
DNA polymerase α-primase
complex
α-prim
MCM
2-7
ε
DNA polymerase ε
complex
RPA
DNA polymerase α-primase complex
α1
α2
Pri1
Pri2
DNA polymerase δ complex
δ1
δ2
δ3
δ4
DNA polymerase ε complex
ε1
ε2
ε3
ε4
MCM complex (helicase)
Mcm2
Mcm3
Mcm4
Mcm5
Mcm6
Mcm7
RFA1
RFA2/4
RPA3
RFC1
RFC2/4
RFC3/5
Dna2
RNaseH1
RNaseH2A
RNaseH2B
RNaseH2C
Fen1
Lig1
03030 10/20/10
(c) Kanehisa Laboratories

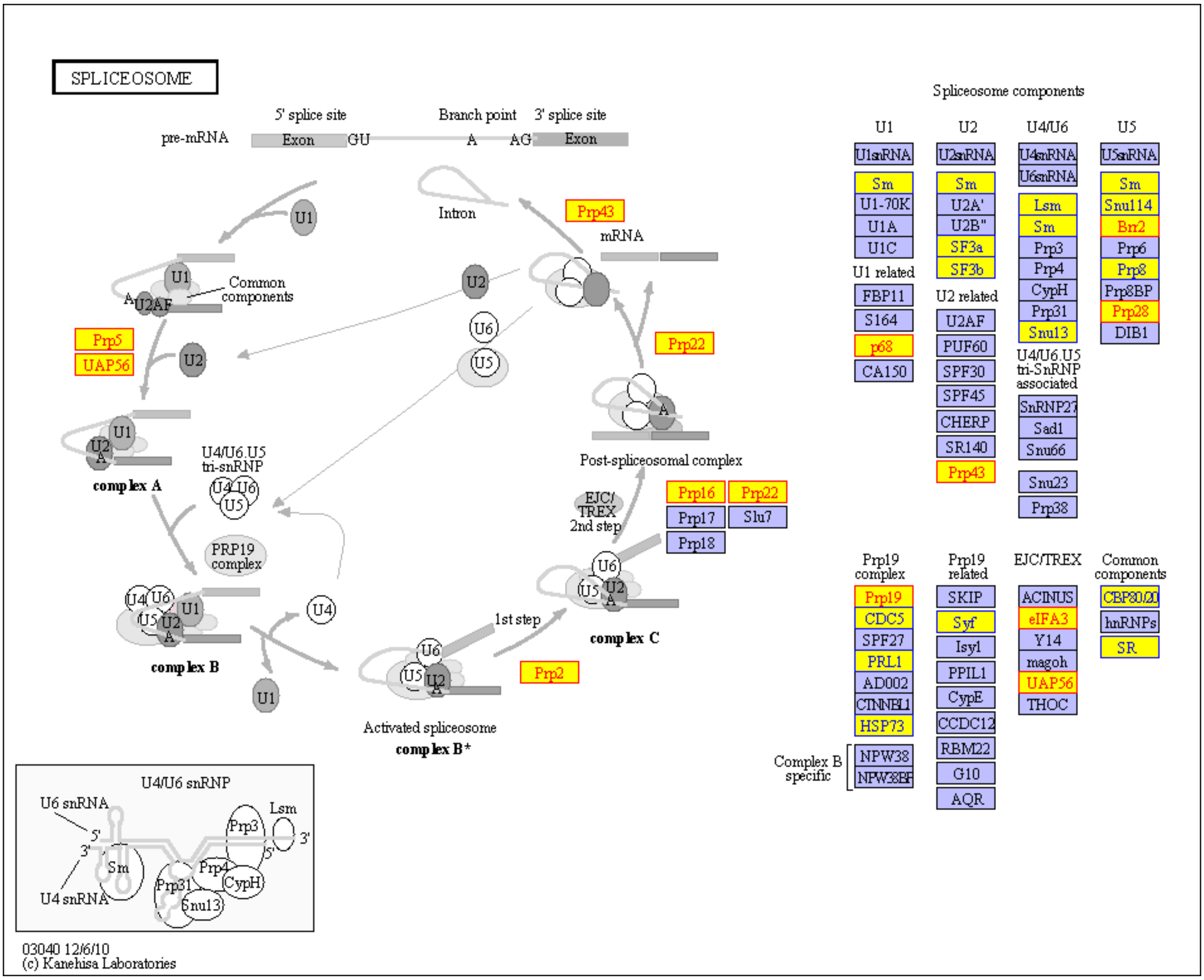
SPLICEOSOME
pre-mRNA
5' splice site
Branch point
3' splice site
Exon
GU
A
AG
Exon
Intron
mRNA
Prp43
Common components
Prp5
UAP56
Prp22
complex A
U4/U6.U5 tri-snRNP
PRP19 complex
Post-spliceosomal complex
EJC/ TREX
2nd step
Prp16
Prp22
Prp17
Slu7
Prp18
complex B
1st step
complex C
Prp2
Activated spliceosome
complex B*
U4/U6 snRNP
U6 snRNA
U4 snRNA
Lsm
Prp3
Sm
Prp4
CypH
Prp31
Snu13
Spliceosome components
U1
U2
U4/U6
U5
U1snRNA
Sm
U1-70K
U1A
U1C
U1 related
FBP11
S164
p68
CA150
U2snRNA
Sm
U2A'
U2B"
SF3a
SF3b
U2 related
U2AF
PUF60
SPF30
SPF45
CHERP
SR140
Prp43
U4snRNA
U6snRNA
Lsm
Sm
Prp3
Prp4
CypH
Prp31
Snu13
U4/U6.U5 tri-SnRNP associated
SnRNP27
Sad1
Snu66
Snu23
Prp38
U5snRNA
Sm
Snu114
Brr2
Prp6
Prp8
Prp8BP
Prp28
DIB1
Prp19 complex
Prp19
CDC5
SPF27
PRL1
AD002
CTNNBL1
HSP73
Complex B specific
NPW38
NPW38BP
Prp19 related
SKIP
Syf
Isy1
PPIL1
CypE
CCDC12
RBM22
G10
AQR
EJC/TREX
ACINUS
eIFA3
Y14
magoh
UAP56
THOC
Common components
CBP80/20
hnRNPs
SR
03040 12/6/10
(c) Kanehisa Laboratories

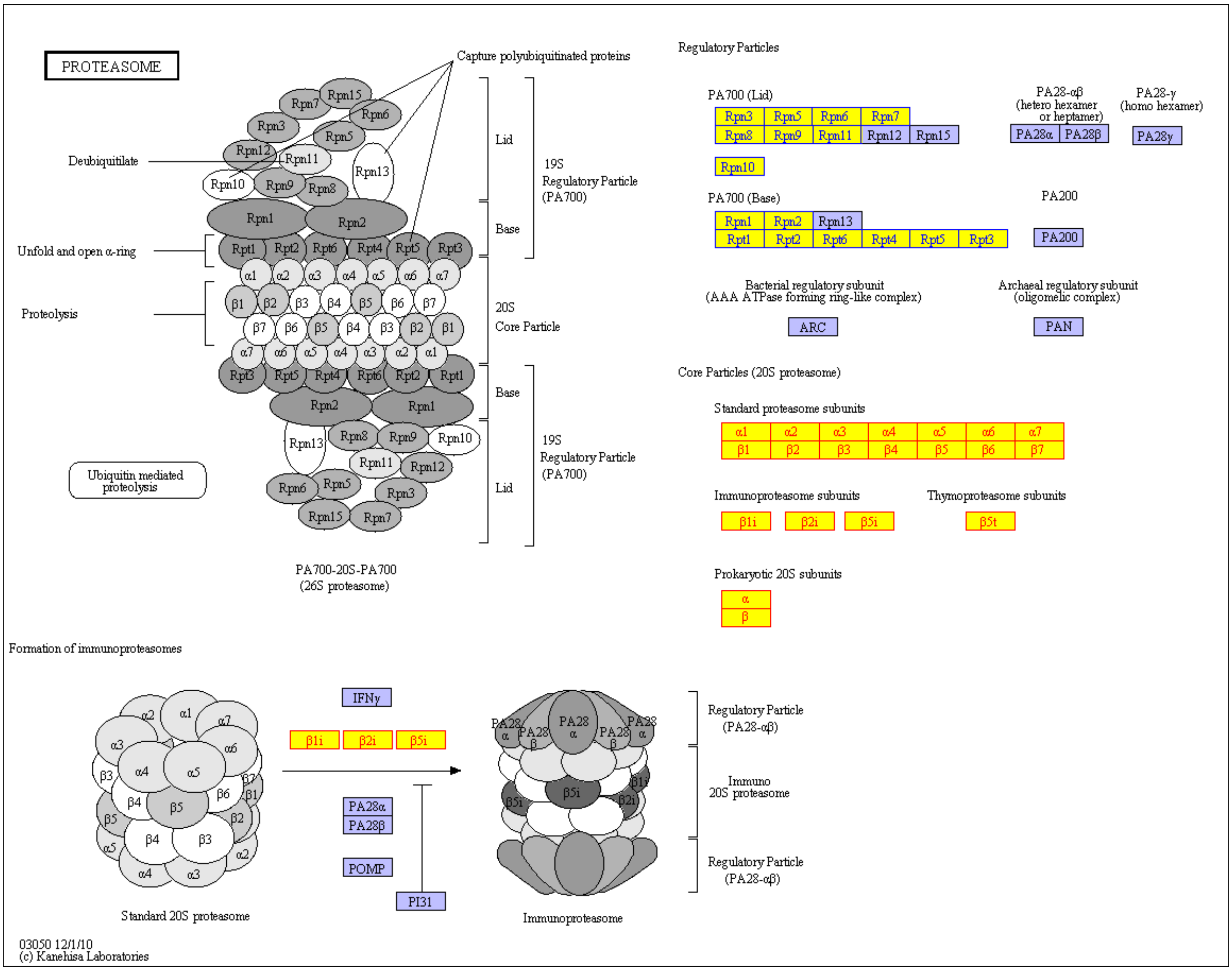
PROTEASOME
Capture polyubiquitinated proteins
Deubiquitilate
Unfold and open α-ring
Proteolysis
Lid
Base
19S
Regulatory Particle
(PA700)
20S
Core Particle
Ubiquitin mediated proteolysis
PA700-20S-PA700
(26S proteasome)
Regulatory Particles
PA700 (Lid)
Rpn3
Rpn5
Rpn6
Rpn7
Rpn8
Rpn9
Rpn11
Rpn12
Rpn15
Rpn10
PA700 (Base)
Rpn1
Rpn2
Rpn13
Rpt1
Rpt2
Rpt6
Rpt4
Rpt5
Rpt3
PA28-αβ
(hetero hexamer or heptamer)
PA28-γ
(homo hexamer)
PA28α
PA28β
PA28γ
PA200
Bacterial regulatory subunit
(AAA ATPase forming ring-like complex)
ARC
Archaeal regulatory subunit
(oligomeric complex)
PAN
Core Particles (20S proteasome)
Standard proteasome subunits
α1 α2 α3 α4 α5 α6 α7
β1 β2 β3 β4 β5 β6 β7
Immunoproteasome subunits
β1i β2i β5i
Thymoproteasome subunits
β5t
Prokaryotic 20S subunits
α
β
Formation of immunoproteasomes
IFNγ
β1i β2i β5i
PA28α
PA28β
POMP
PI31
Standard 20S proteasome
Immunoproteasome
Regulatory Particle
(PA28-αβ)
Immuno
20S proteasome
Regulatory Particle
(PA28-αβ)
03050 12/1/10
(c) Kanehisa Laboratories

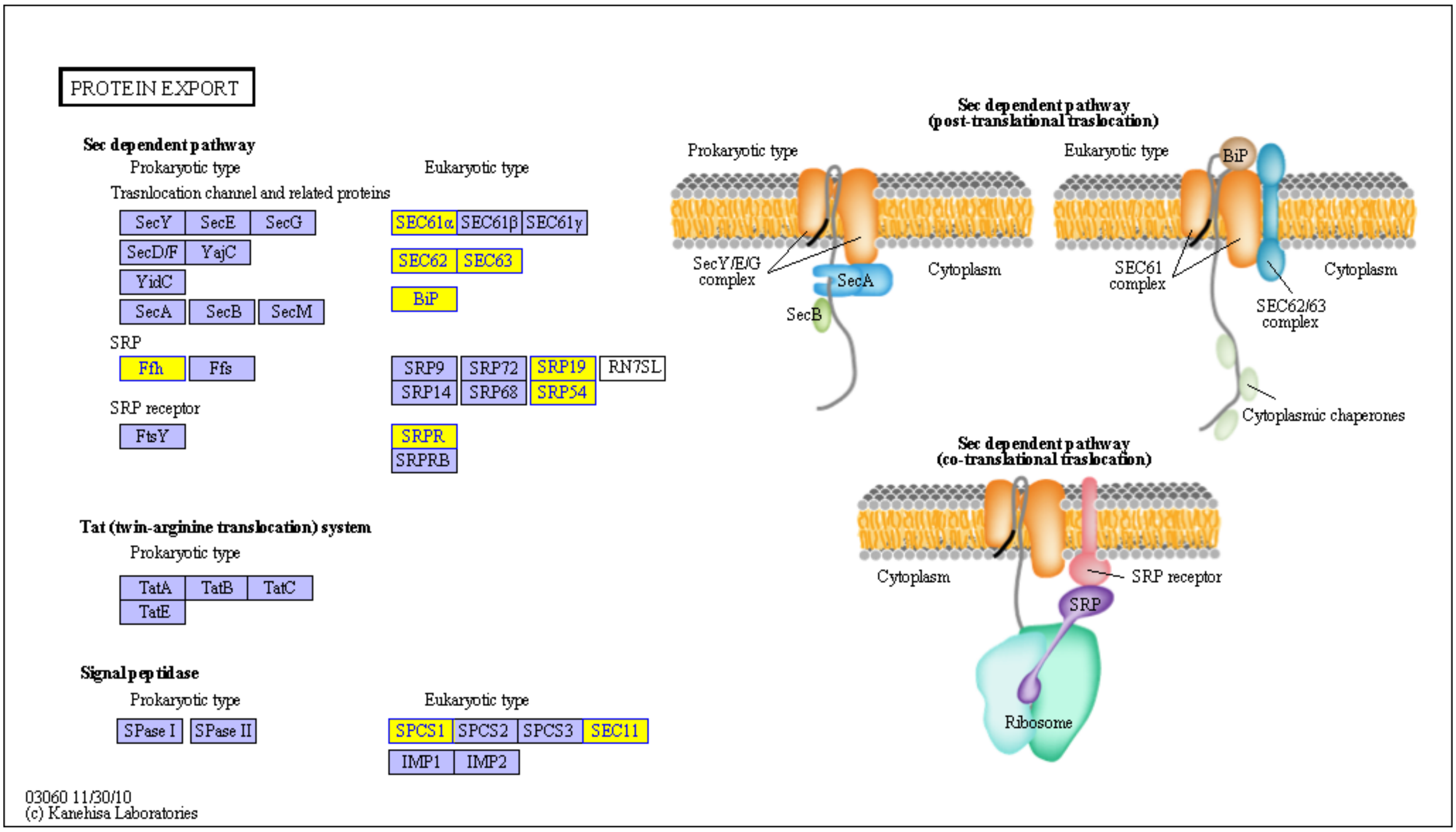
PROTEIN EXPORT
Sec dependent pathway
Prokaryotic type
Eukaryotic type
Trasnlocation channel and related proteins
SecY
SecE
SecG
SecD/F
YajC
YidC
SecA
SecB
SecM
SEC61α
SEC61β
SEC61γ
SEC62
SEC63
BiP
SRP
Ffh
Ffs
SRP9
SRP72
SRP19
RN7SL
SRP14
SRP68
SRP54
SRP receptor
FtsY
SRPR
SRPRB
Tat (twin-arginine translocation) system
Prokaryotic type
TatA
TatB
TatC
TatE
Signal peptidase
Prokaryotic type
Eukaryotic type
SPase I
SPase II
SPCS1
SPCS2
SPCS3
SEC11
IMP1
IMP2
Sec dependent pathway
(post-translational traslocation)
Prokaryotic type
Eukaryotic type
BiP
SecY/E/G
complex
SecA
SecB
Cytoplasm
SEC61
complex
SEC62/63
complex
Cytoplasm
Cytoplasmic chaperones
Sec dependent pathway
(co-translational traslocation)
Cytoplasm
SRP receptor
SRP
Ribosome
03060 11/30/10
(c) Kanehisa Laboratories

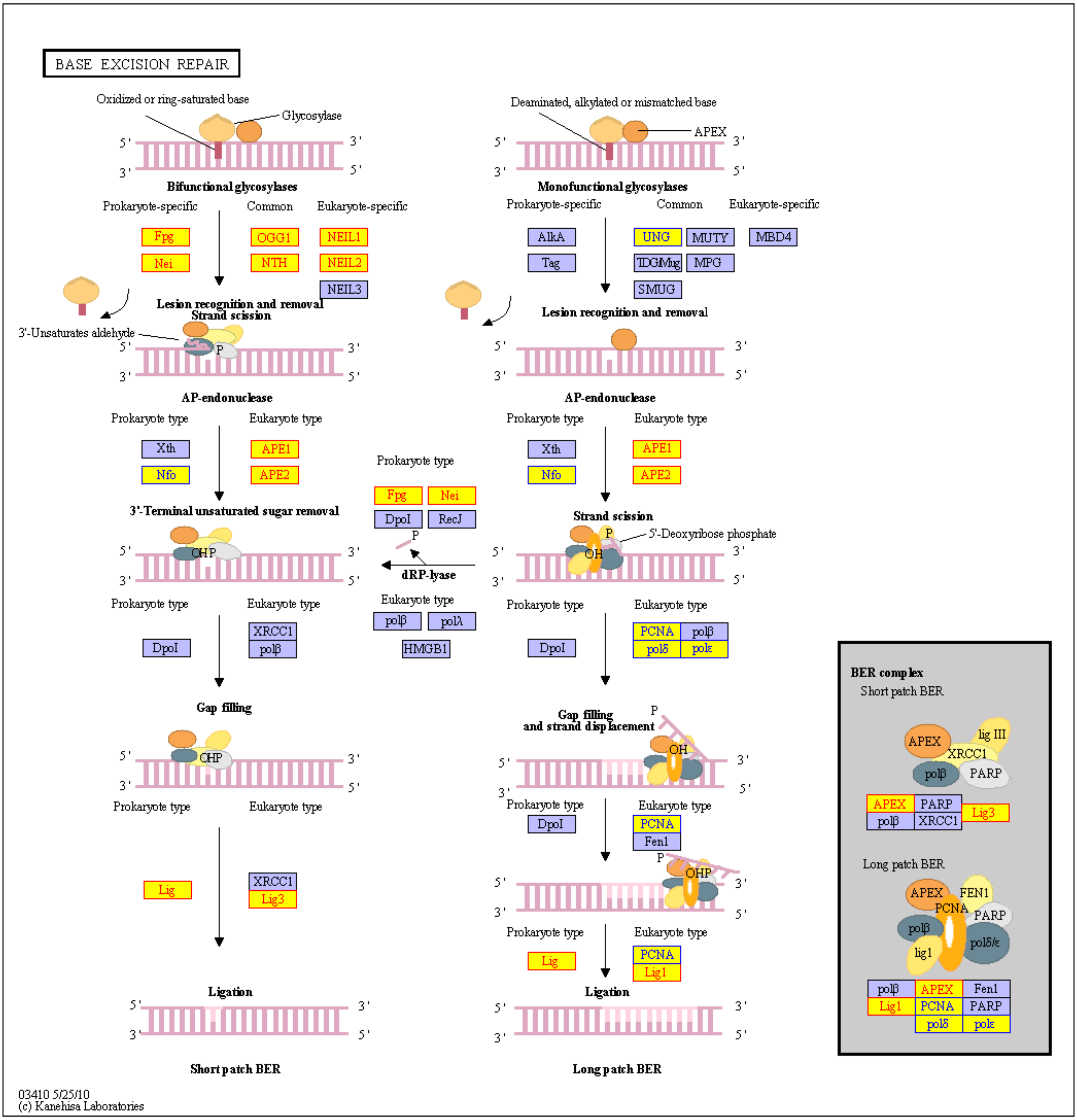
BASE EXCISION REPAIR
Oxidized or ring-saturated base
Glycosylase
Bifunctional glycosylases
Prokaryote-specific
Common
Eukaryote-specific
Fpg
Nei
OGG1
NTH
NEIL1
NEIL2
NEIL3
Lesion recognition and removal
Strand scission
3'-Unsaturates aldehyde
AP-endonuclease
Prokaryote type
Eukaryote type
Xth
Nfo
APE1
APE2
3'-Terminal unsaturated sugar removal
DpoI
RecJ
dRP-lyase
polβ
polλ
HMGB1
XRCC1
Gap filling
Lig
Lig3
Ligation
Short patch BER
Deaminated, alkylated or mismatched base
APEX
Monofunctional glycosylases
AlkA
Tag
UNG
MUTY
MBD4
TDG/Mug
MPG
SMUG
Strand scission
5'-Deoxyribose phosphate
PCNA
polδ
polε
Gap filling and strand displacement
Fen1
Lig1
Long patch BER
BER complex
Short patch BER
lig III
PARP
Long patch BER
FEN1
lig1
polδ/ε
03410 5/25/10
(c) Kanehisa Laboratories

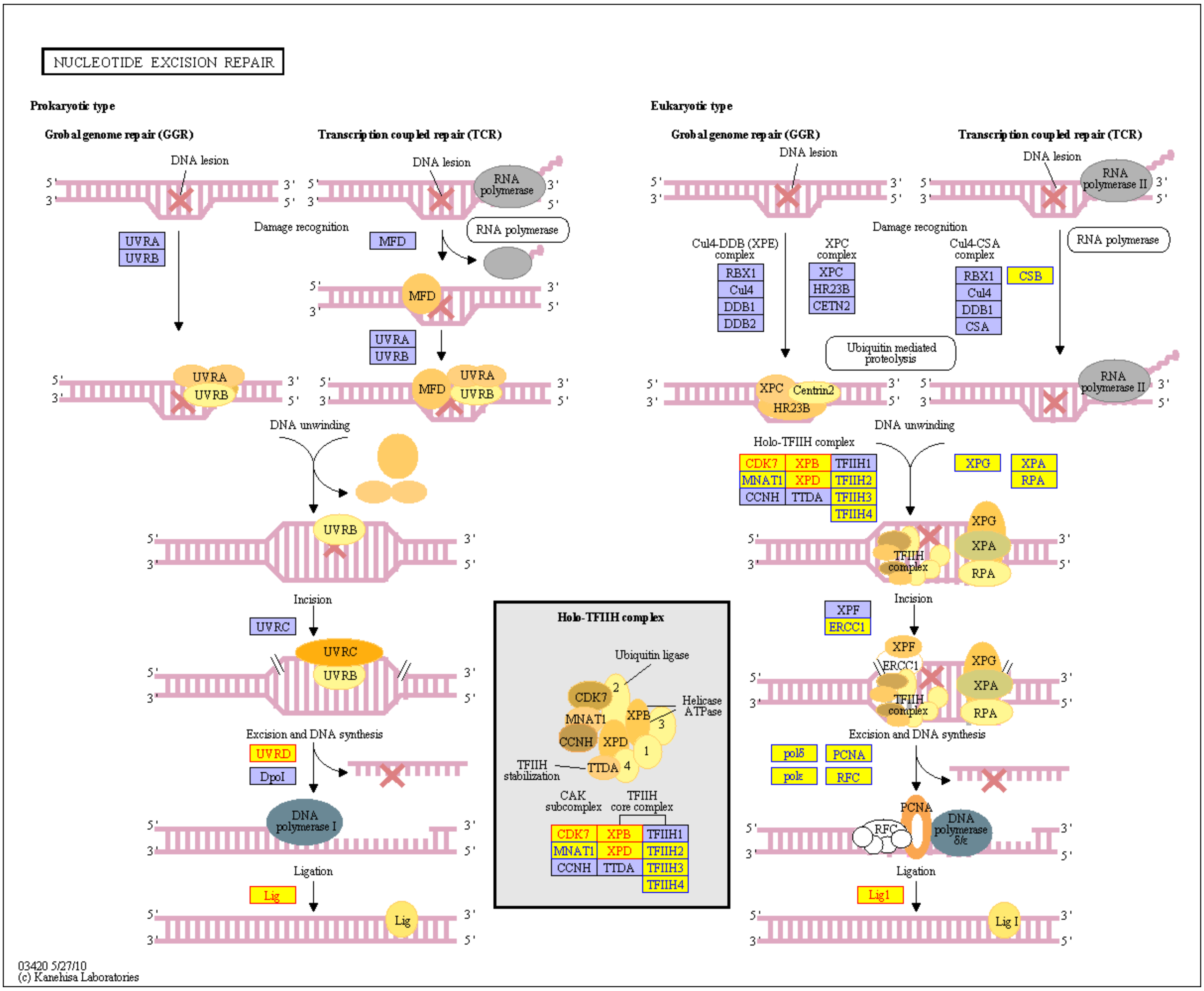

NUCLEOTIDE EXCISION REPAIR
Prokaryotic type
Grobal genome repair (GGR)
Transcription coupled repair (TCR)
DNA lesion
RNA polymerase
Damage recognition
UVRA
UVRB
MFD
DNA unwinding
Incision
UVRC
Excision and DNA synthesis
UVRD
DpoI
DNA polymerase I
Ligation
Lig
Eukaryotic type
Cul4-DDB (XPE) complex
RBX1
Cul4
DDB1
DDB2
XPC complex
XPC
HR23B
CETN2
Cul4-CSA complex
CSA
CSB
RNA polymerase II
Ubiquitin mediated proteolysis
Centrin2
Holo-TFIIH complex
CDK7
XPB
TFIIH1
MNAT1
XPD
TFIIH2
CCNH
TTDA
TFIIH3
TFIIH4
XPG
XPA
RPA
TFIIH complex
XPF
ERCC1
polδ
polε
PCNA
RFC
DNA polymerase δ/ε
Lig1
Lig I
Ubiquitin ligase
Helicase ATPase
TFIIH stabilization
CAK subcomplex
TFIIH core complex
03420 5/27/10
(c) Kanehisa Laboratories

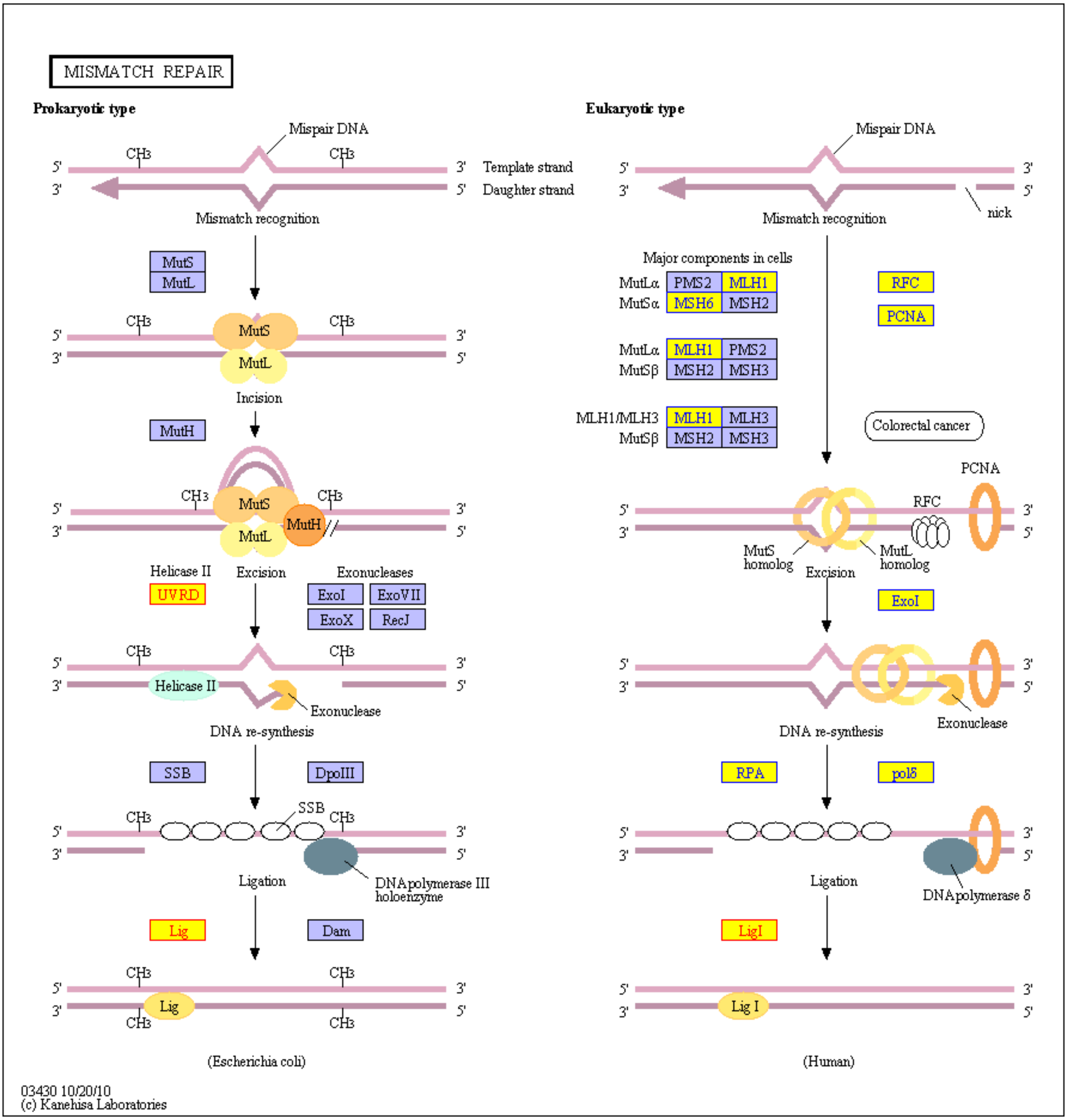
MISMATCH REPAIR
Prokaryotic type
Mispair DNA
CH3
5'
3'
Template strand
Daughter strand
Mismatch recognition
MutS
MutL
Incision
MutH
Helicase II
UVRD
Excision
Exonucleases
ExoI
ExoVII
ExoX
RecJ
Exonuclease
DNA re-synthesis
SSB
DpoIII
DNApolymerase III holoenzyme
Ligation
Lig
Dam
(Escherichia coli)
Eukaryotic type
Mispair DNA
nick
Mismatch recognition
Major components in cells
MutLα
MutSα
PMS2
MLH1
MSH6
MSH2
MutLα
MutSβ
MLH1
PMS2
MSH2
MSH3
MLH1/MLH3
MutSβ
MLH1
MLH3
MSH2
MSH3
RFC
PCNA
Colorectal cancer
PCNA
RFC
MutS homolog
MutL homolog
Excision
ExoI
Exonuclease
DNA re-synthesis
RPA
polδ
DNApolymerase δ
Ligation
LigI
Lig I
(Human)
03430 10/20/10
(c) Kanehisa Laboratories

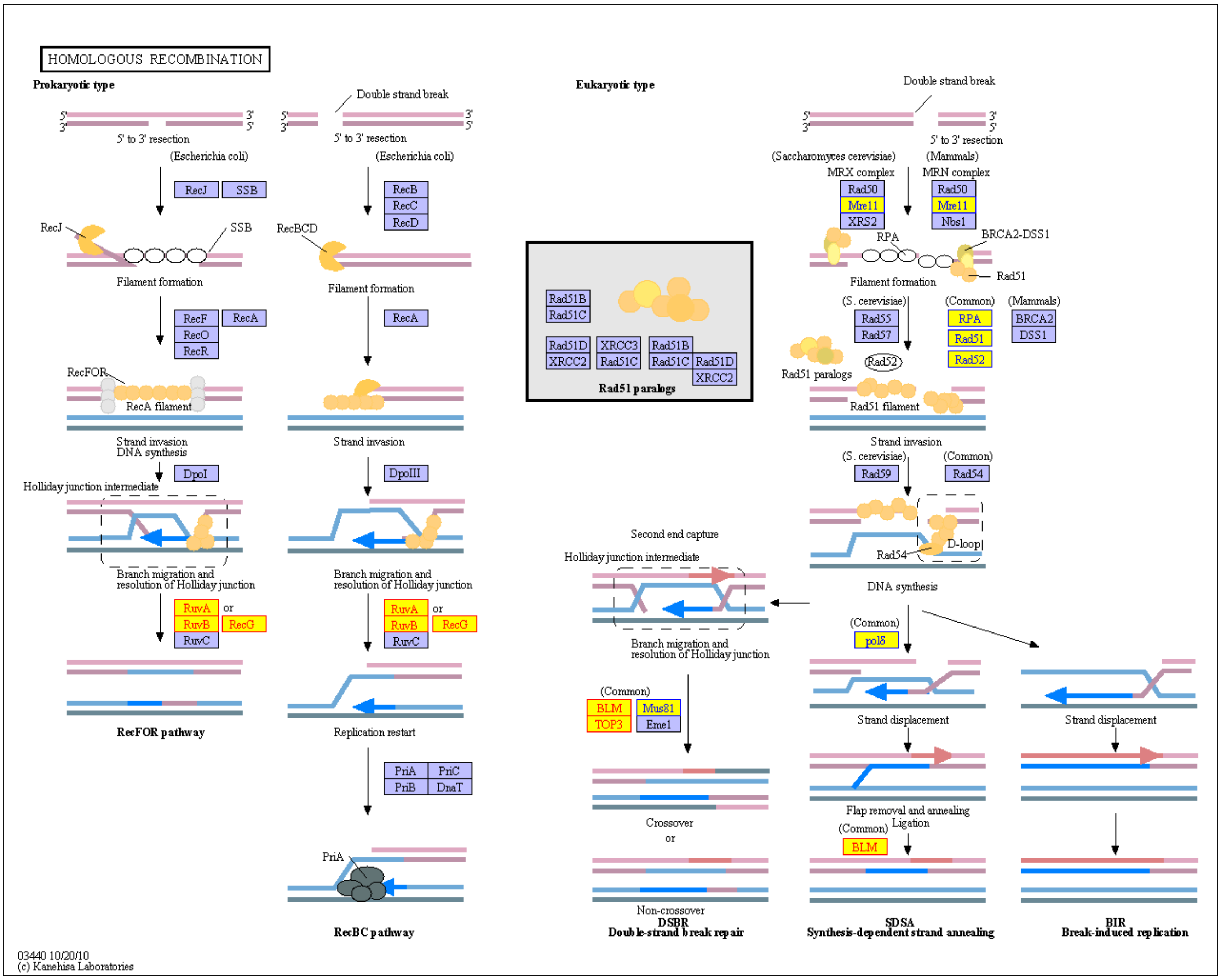
HOMOLOGOUS RECOMBINATION
Prokaryotic type
Eukaryotic type
Double strand break
5' to 3' resection
(Escherichia coli)
RecJ
SSB
RecB
RecC
RecD
RecBCD
Filament formation
RecF
RecO
RecR
RecA
RecFOR
RecA filament
Strand invasion
DNA synthesis
DpoI
DpoIII
Holliday junction intermediate
Branch migration and resolution of Holliday junction
RuvA
RuvB
RuvC
RecG
or
RecFOR pathway
Replication restart
PriA
PriB
PriC
DnaT
RecBC pathway
Rad51B
Rad51C
Rad51D
XRCC2
XRCC3
Rad51 paralogs
(Saccharomyces cerevisiae)
(Mammals)
MRX complex
MRN complex
Rad50
Mre11
XRS2
Nbs1
RPA
BRCA2-DSS1
Rad51
(S. cerevisiae)
(Common)
Rad55
Rad57
Rad52
BRCA2
DSS1
Rad51 filament
Rad59
Rad54
D-loop
Second end capture
DNA synthesis
pol δ
BLM
TOP3
Mus81
Eme1
Crossover
Non-crossover
Strand displacement
Flap removal and annealing
Ligation
DSBR
Double-strand break repair
SDSA
Synthesis-dependent strand annealing
BIR
Break-induced replication
03440 10/20/10
(c) Kanehisa Laboratories

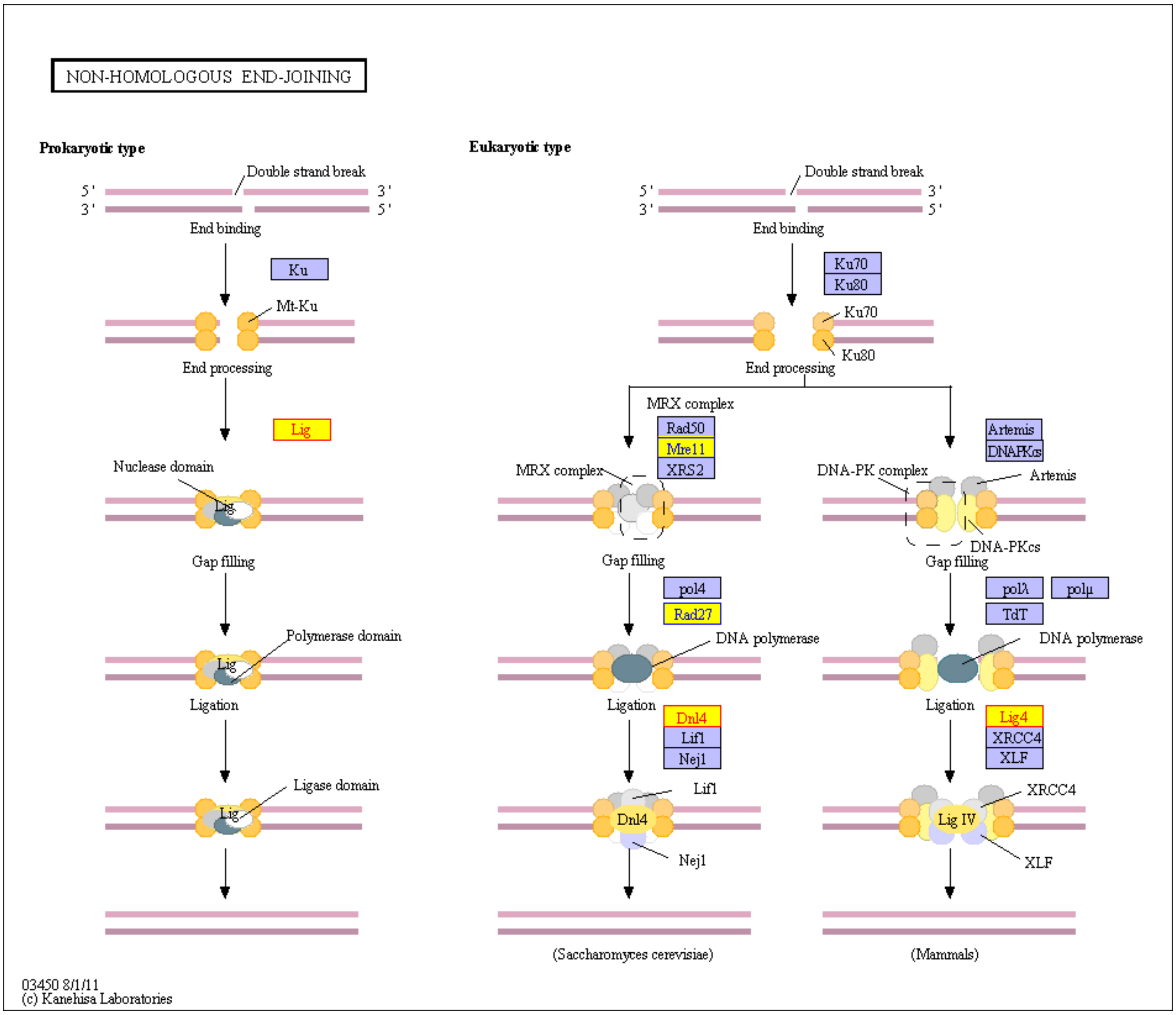
NON-HOMOLOGOUS END-JOINING
Prokaryotic type
Double strand break
5'
3'
3'
5'
End binding
Ku
Mt-Ku
End processing
Lig
Nuclease domain
Lig
Gap filling
Polymerase domain
Lig
Ligation
Ligase domain
Lig
Eukaryotic type
Double strand break
5'
3'
3'
5'
End binding
Ku70
Ku80
Ku70
Ku80
End processing
MRX complex
Rad50
Mre11
XRS2
MRX complex
Artemis
DNAPKcs
DNA-PK complex
Artemis
DNA-PKcs
Gap filling
pol4
Rad27
DNA polymerase
Gap filling
polλ
polμ
TdT
DNA polymerase
Ligation
Dnl4
Lif1
Nej1
Lif1
Dnl4
Nej1
Ligation
Lig4
XRCC4
XLF
XRCC4
Lig IV
XLF
(Saccharomyces cerevisiae)
(Mammals)
03450 8/1/11
(c) Kanehisa Laboratories

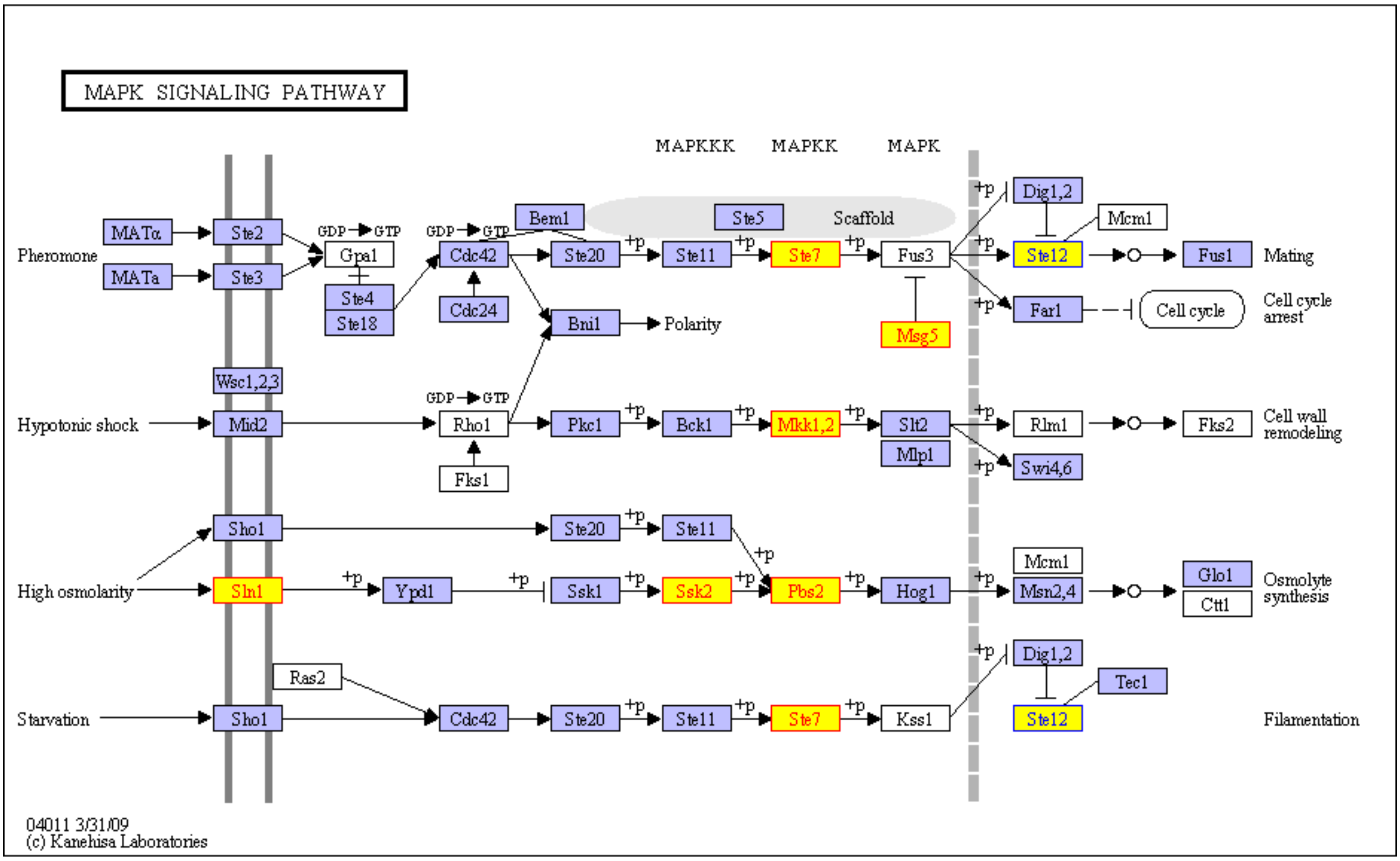
MAPK SIGNALING PATHWAY
MAPKKK
MAPKK
MAPK
Pheromone
MATα
MATa
Ste2
Ste3
GDP
GTP
Gpa1
Ste4
Ste18
Cdc42
Cdc24
Bem1
Ste20
Ste5
Scaffold
Ste11
Ste7
Fus3
Msg5
Bni1
Polarity
Dig1,2
Mcm1
Ste12
Fus1
Mating
Far1
Cell cycle
Cell cycle arrest
Hypotonic shock
Wsc1,2,3
Mid2
Rho1
Fks1
Pkc1
Bck1
Mkk1,2
Slt2
Mlp1
Rlm1
Swi4,6
Fks2
Cell wall remodeling
High osmolarity
Sho1
Sln1
Ypd1
Ssk1
Ssk2
Pbs2
Hog1
Msn2,4
Glo1
Ctt1
Osmolyte synthesis
Starvation
Ras2
Kss1
Tec1
Filamentation
+p
04011 3/31/09
(c) Kanehisa Laboratories

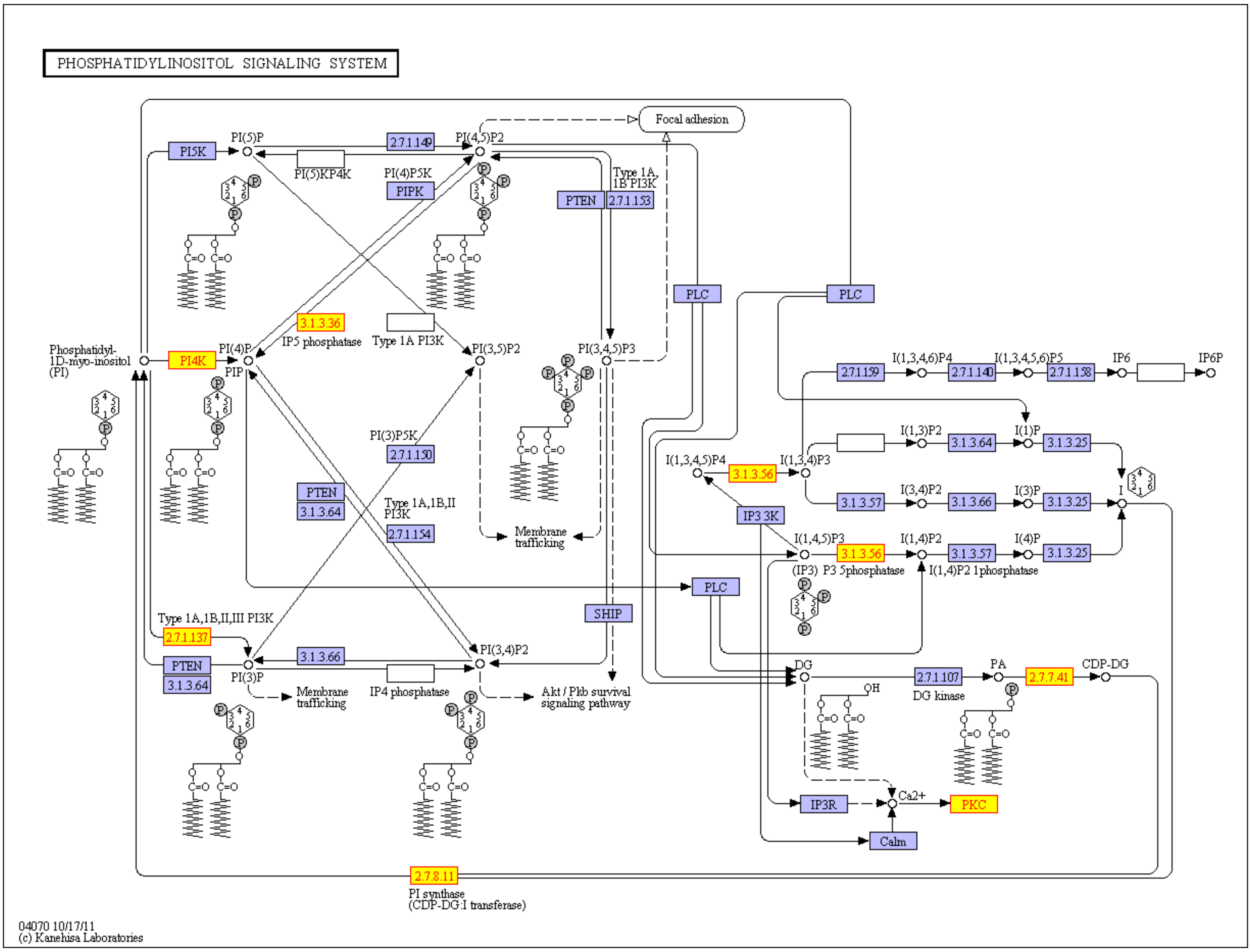
PHOSPHATIDYLINOSITOL SIGNALING SYSTEM
PI5K
PI(5)P
2.7.1.149
PI(4,5)P2
Focal adhesion
PI(5)P4K
PI(4)P5K
PIPK
Type 1A, 1B PI3K
PTEN
2.7.1.153
PLC
3.1.3.36
IP5 phosphatase
Type 1A PI3K
Phosphatidyl-1D-myo-inositol (PI)
PI4K
PI(4)P
PIP
PI(3,5)P2
PI(3,4,5)P3
I(1,3,4,6)P4
I(1,3,4,5,6)P5
IP6
IP6P
2.7.1.159
2.7.1.140
2.7.1.158
I(1,3)P2
I(1)P
3.1.3.64
3.1.3.25
PI(3)P5K
2.7.1.150
I(1,3,4,5)P4
3.1.3.56
I(1,3,4)P3
I(3,4)P2
I(3)P
I
3.1.3.57
3.1.3.66
PTEN
3.1.3.64
Type 1A,1B,II PI3K
2.7.1.154
Membrane trafficking
IP3 3K
I(1,4,5)P3
(IP3) P3 5phosphatase
I(1,4)P2
I(4)P
I(1,4)P2 1phosphatase
SHIP
Type 1A,1B,II,III PI3K
2.7.1.137
PI(3,4)P2
PTEN
3.1.3.64
PI(3)P
3.1.3.66
IP4 phosphatase
Akt / Pkb survival signaling pathway
DG
2.7.1.107
DG kinase
PA
2.7.7.41
CDP-DG
OH
IP3R
Ca2+
PKC
Calm
2.7.8.11
PI synthase (CDP-DG:I transferase)
04070 10/17/11
(c) Kanehisa Laboratories

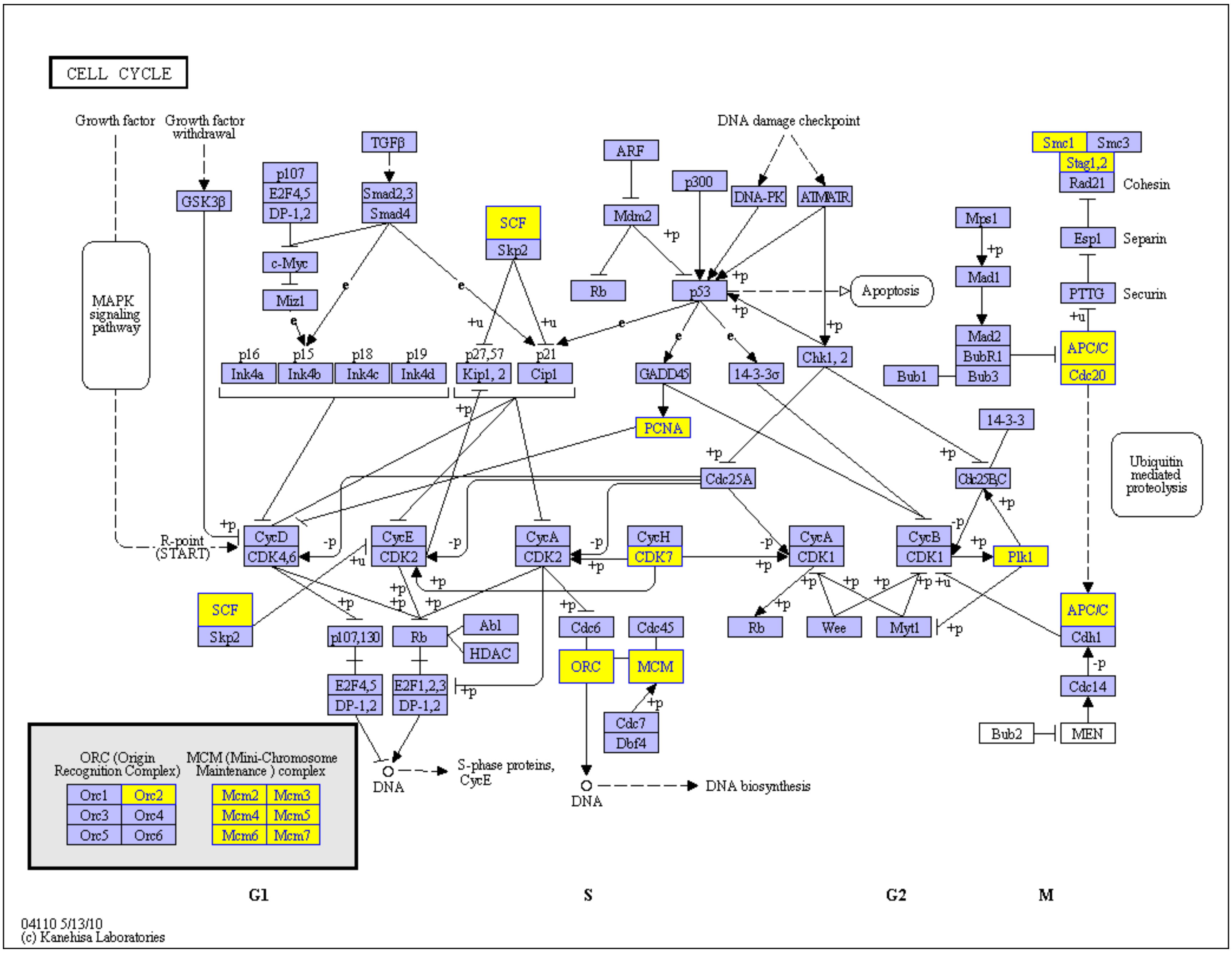
CELL CYCLE
Growth factor
Growth factor withdrawal
MAPK signaling pathway
GSK3β
p107
E2F4,5
DP-1,2
TGFβ
Smad2,3
Smad4
c-Myc
Miz1
SCF
Skp2
ARF
Mdm2
Rb
p300
DNA damage checkpoint
DNA-PK
ATM/ATR
p53
Apoptosis
p16
p15
p18
p19
p27,57
p21
Ink4a
Ink4b
Ink4c
Ink4d
Kip1, 2
Cip1
GADD45
14-3-3σ
Chk1, 2
PCNA
Cdc25A
R-point (START)
CycD
CDK4,6
CycE
CDK2
CycA
CDK2
CycH
CDK7
CycA
CDK1
CycB
CDK1
SCF
Skp2
p107,130
E2F4,5
DP-1,2
Rb
Abl
HDAC
E2F1,2,3
DP-1,2
Cdc6
Cdc45
ORC
MCM
Cdc7
Dbf4
DNA
S-phase proteins, CycE
DNA
DNA biosynthesis
Rb
Wee
Myt1
Smc1
Smc3
Stag1,2
Rad21
Cohesin
Esp1
Separin
PTTG
Securin
Mps1
Mad1
Mad2
BubR1
Bub3
Bub1
APC/C
Cdc20
14-3-3
Cdc25B,C
Plk1
Ubiquitin mediated proteolysis
APC/C
Cdh1
Cdc14
Bub2
MEN
ORC (Origin Recognition Complex)
Orc1
Orc2
Orc3
Orc4
Orc5
Orc6
MCM (Mini-Chromosome Maintenance) complex
Mcm2
Mcm3
Mcm4
Mcm5
Mcm6
Mcm7
G1
S
G2
M
04110 5/13/10
(c) Kanehisa Laboratories

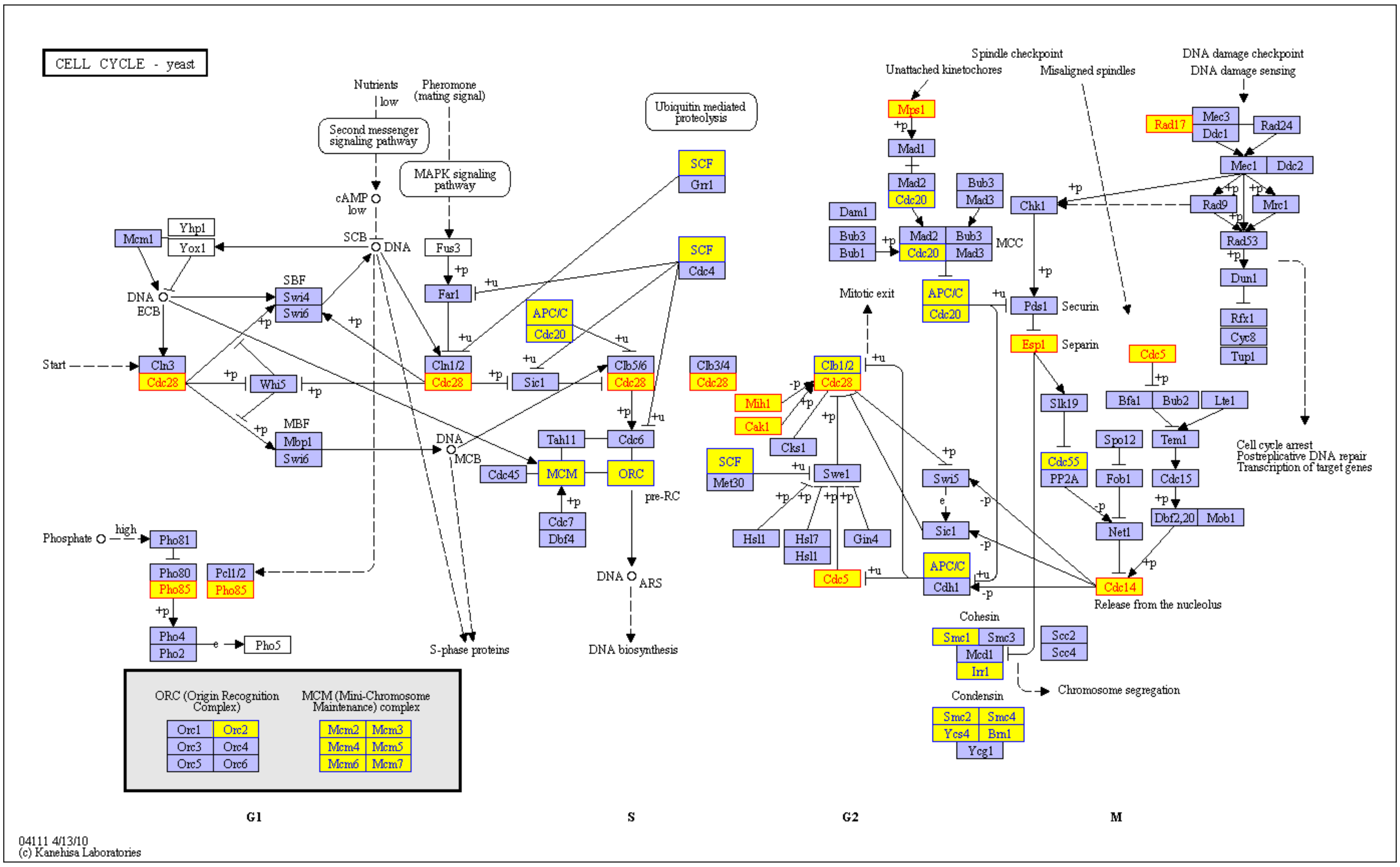

CELL CYCLE - yeast
Nutrients
low
Pheromone (mating signal)
Second messenger signaling pathway
MAPK signaling pathway
Ubiquitin mediated proteolysis
cAMP low
SCB
DNA
Mcm1
Yhp1
Yox1
Fus3
Far1
SCF
Grr1
Cdc4
APC/C
Cdc20
DNA ECB
SBF
Swi4
Swi6
Start
Cln3
Cdc28
Whi5
Cln1/2
Sic1
Clb5/6
Clb3/4
MBF
Mbp1
DNA MCB
Tah11
Cdc6
Cdc45
MCM
ORC
Cdc7
Dbf4
pre-RC
DNA ARS
DNA biosynthesis
S-phase proteins
Phosphate
high
Pho81
Pho80
Pho85
Pcl1/2
Pho4
Pho2
Pho5
ORC (Origin Recognition Complex)
Orc1
Orc2
Orc3
Orc4
Orc5
Orc6
MCM (Mini-Chromosome Maintenance) complex
Mcm2
Mcm3
Mcm4
Mcm5
Mcm6
Mcm7
Spindle checkpoint
Unattached kinetochores
Misaligned spindles
DNA damage checkpoint
DNA damage sensing
Mps1
Mad1
Mad2
Bub3
Mad3
Dam1
Bub1
MCC
Rad17
Mec3
Ddc1
Rad24
Mec1
Ddc2
Chk1
Rad9
Mrc1
Rad53
Dun1
Rfx1
Cyc8
Tup1
Mitotic exit
Pds1
Securin
Esp1
Separin
Cdc5
Mih1
Cak1
Clb1/2
Cks1
Met30
Swe1
Hsl1
Hsl7
Gin4
Swi5
Slk19
Bfa1
Bub2
Lte1
Spo12
Tem1
Cdc55
PP2A
Fob1
Cdc15
Net1
Dbf2,20
Mob1
Cdh1
Cdc14
Release from the nucleolus
Cell cycle arrest
Postreplicative DNA repair
Transcription of target genes
Cohesin
Smc1
Smc3
Mcd1
Irr1
Scc2
Scc4
Chromosome segregation
Condensin
Smc2
Smc4
Ycs4
Brn1
Ycg1
G1
S
G2
M
04111 4/13/10
(c) Kanehisa Laboratories

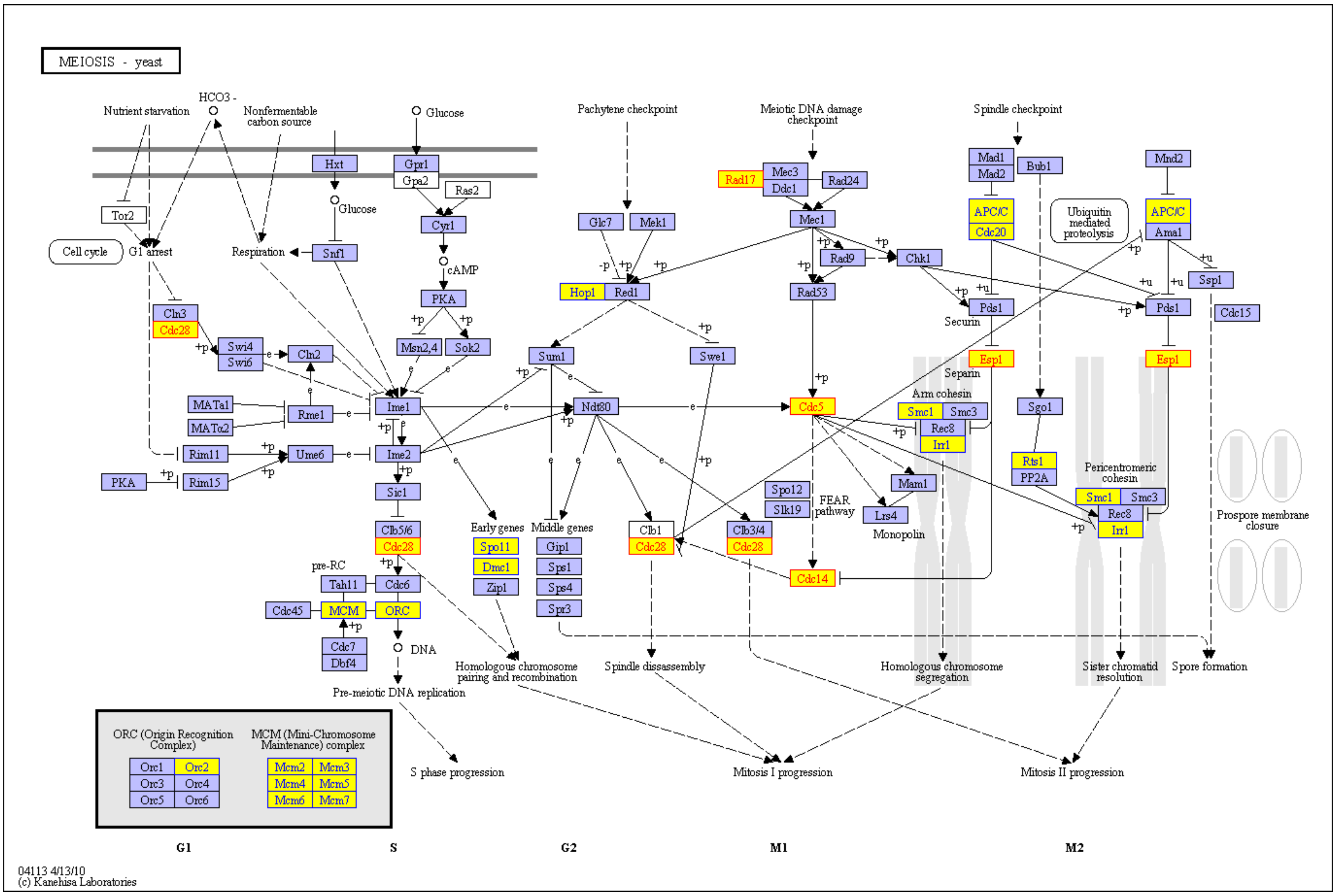
MEIOSIS - yeast
Nutrient starvation
HCO3 -
Nonfermentable carbon source
Glucose
Pachytene checkpoint
Meiotic DNA damage checkpoint
Spindle checkpoint
Hxt
Gpr1
Gpa2
Ras2
Tor2
Cell cycle
G1 arrest
Respiration
Snf1
Cyr1
cAMP
PKA
Msn2,4
Sok2
Cln3
Cdc28
Swi4
Swi6
Cln2
MATa1
MATα2
Rme1
Ime1
Rim11
Ume6
Ime2
PKA
Rim15
Sic1
Clb5/6
Cdc28
pre-RC
Tah11
Cdc6
Cdc45
MCM
ORC
Cdc7
Dbf4
DNA
Pre-meiotic DNA replication
S phase progression
Glc7
Mek1
Hop1
Red1
Sum1
Ndt80
Swe1
Early genes
Spo11
Dmc1
Zip1
Middle genes
Gip1
Sps1
Sps4
Spr3
Clb1
Cdc28
Clb3/4
Cdc28
Homologous chromosome pairing and recombination
Spindle dissassembly
Mitosis I progression
Rad17
Mec3
Ddc1
Rad24
Mec1
Rad9
Chk1
Rad53
Cdc5
Spo12
Slk19
FEAR pathway
Cdc14
Mam1
Lrs4
Monopolin
Homologous chromosome segregation
Mad1
Mad2
Bub1
APC/C
Cdc20
Ubiquitin mediated proteolysis
Pds1
Securin
Esp1
Separin
Arm cohesin
Smc1
Smc3
Rec8
Irr1
Sgo1
Rts1
PP2A
Mnd2
APC/C
Ama1
Ssp1
Pds1
Cdc15
Esp1
Pericentromeric cohesin
Smc1
Smc3
Rec8
Irr1
Prospore membrane closure
Sister chromatid resolution
Spore formation
Mitosis II progression
ORC (Origin Recognition Complex)
Orc1
Orc2
Orc3
Orc4
Orc5
Orc6
MCM (Mini-Chromosome Maintenance) complex
Mcm2
Mcm3
Mcm4
Mcm5
Mcm6
Mcm7
G1
S
G2
M1
M2
04113 4/13/10
(c) Kanehisa Laboratories

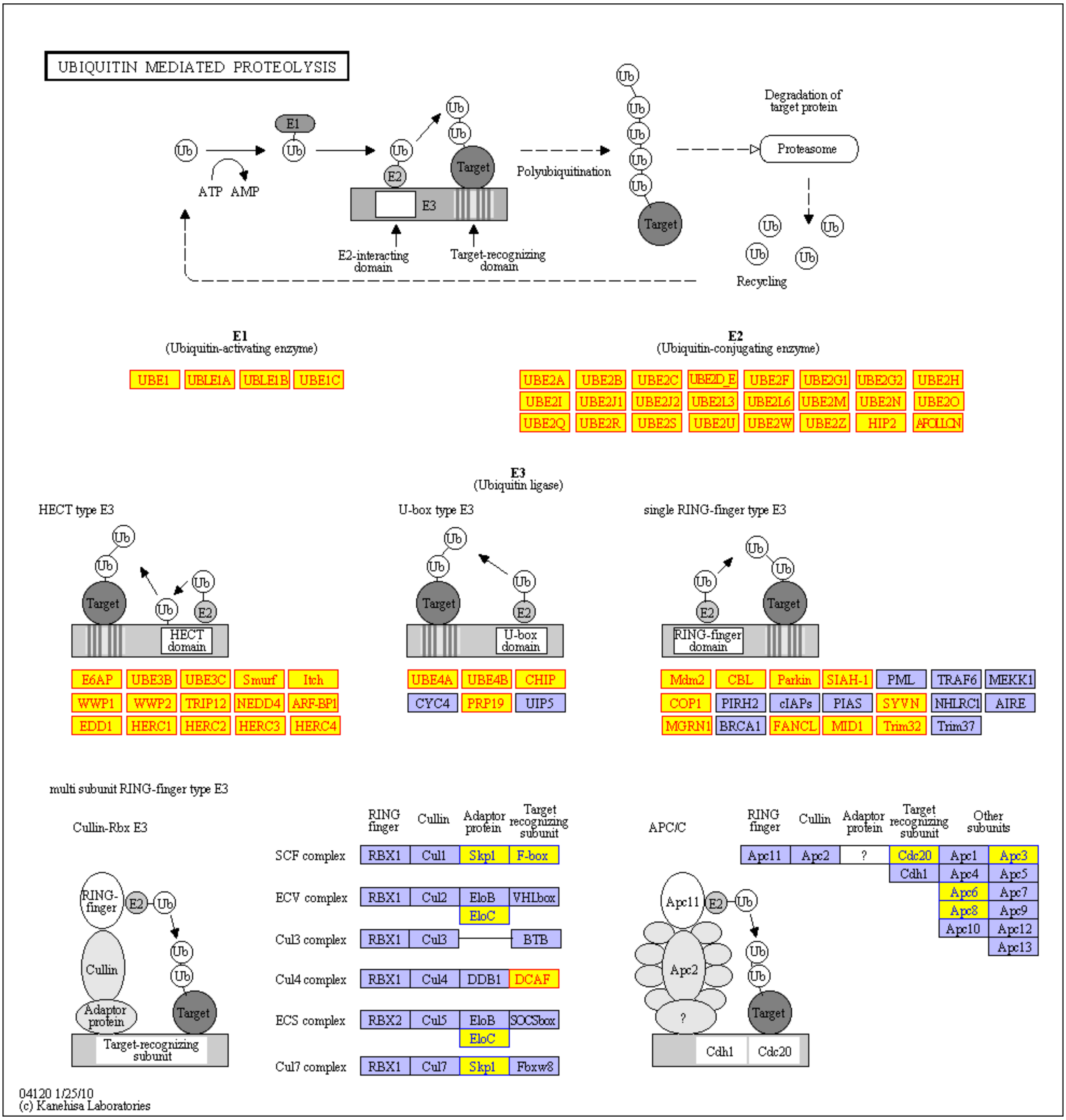
UBIQUITIN MEDIATED PROTEOLYSIS
Ub
E1
ATP AMP
E2
E3
Target
E2-interacting domain
Target-recognizing domain
Polyubiquitination
Degradation of target protein
Proteasome
Recycling
E1
(Ubiquitin-activating enzyme)
UBE1 UBLE1A UBLE1B UBE1C
E2
(Ubiquitin-conjugating enzyme)
UBE2A UBE2B UBE2C UBE2D_E UBE2F UBE2G1 UBE2G2 UBE2H
UBE2I UBE2J1 UBE2J2 UBE2L3 UBE2L6 UBE2M UBE2N UBE2O
UBE2Q UBE2R UBE2S UBE2U UBE2W UBE2Z HIP2 APOLLON
E3
(Ubiquitin ligase)
HECT type E3
HECT domain
E6AP UBE3B UBE3C Smurf Itch
WWP1 WWP2 TRIP12 NEDD4 ARF-BP1
EDD1 HERC1 HERC2 HERC3 HERC4
U-box type E3
U-box domain
UBE4A UBE4B CHIP
CYC4 PRP19 UIP5
single RING-finger type E3
RING-finger domain
Mdm2 CBL Parkin SIAH-1 PML TRAF6 MEKK1
COP1 PIRH2 cIAPs PIAS SYVN NHLRC1 AIRE
MGRN1 BRCA1 FANCL MID1 Trim32 Trim37
multi subunit RING-finger type E3
Cullin-Rbx E3
RING-finger
Cullin
Adaptor protein
Target-recognizing subunit
RING finger
Cullin
Adaptor protein
Target recognizing subunit
SCF complex RBX1 Cul1 Skp1 F-box
ECV complex RBX1 Cul2 EloB VHLbox
EloC
Cul3 complex RBX1 Cul3 BTB
Cul4 complex RBX1 Cul4 DDB1 DCAF
ECS complex RBX2 Cul5 EloB SOCSbox
EloC
Cul7 complex RBX1 Cul7 Skp1 Fbxw8
APC/C
RING finger
Cullin
Adaptor protein
Target recognizing subunit
Other subunits
Apc11 Apc2 ? Cdc20 Apc1 Apc3
Cdh1 Apc4 Apc5
Apc6 Apc7
Apc8 Apc9
Apc10 Apc12
Apc13
Apc11
Apc2
?
Cdh1 Cdc20
04120 1/25/10
(c) Kanehisa Laboratories

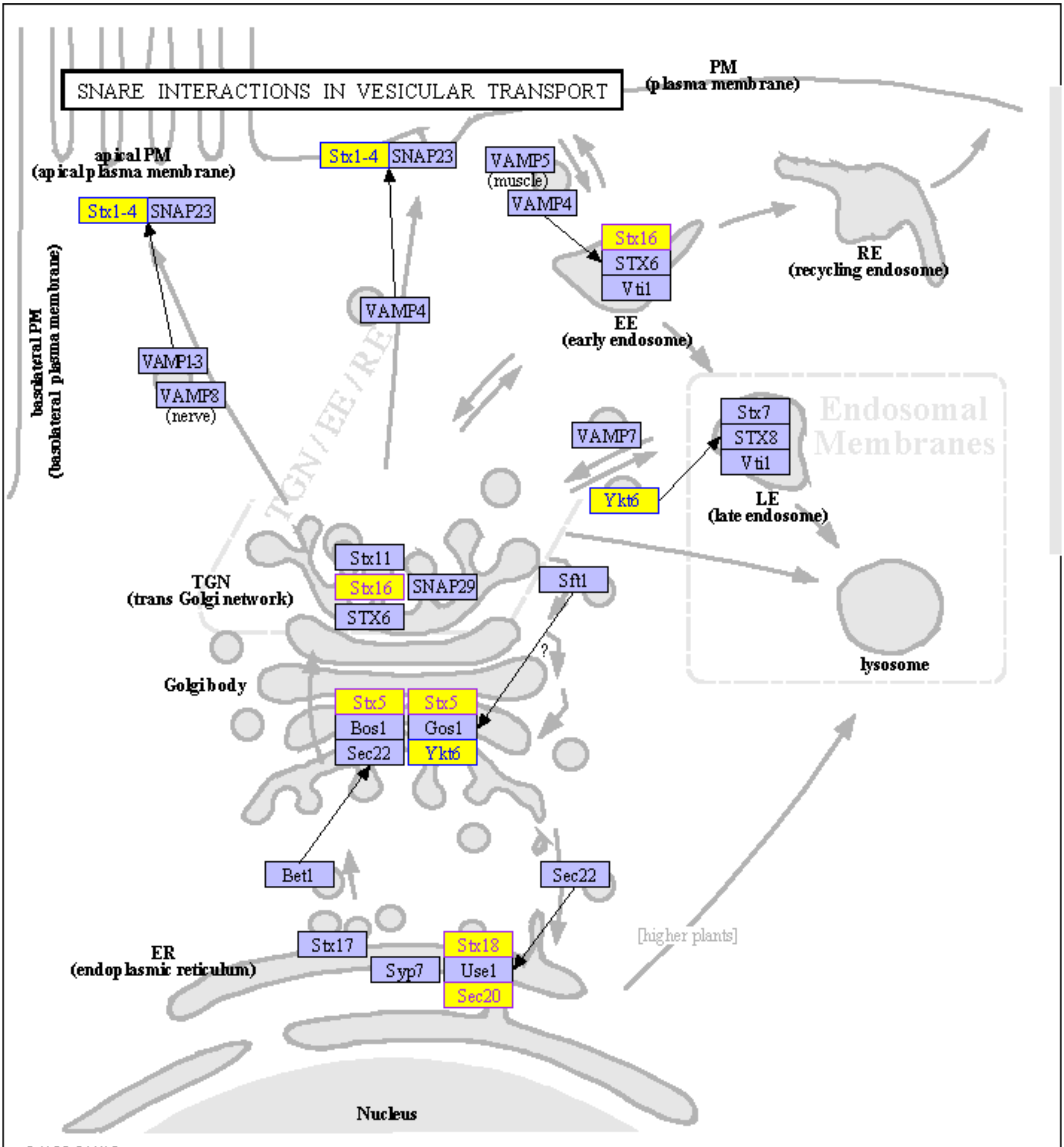

04130 3/4/10

(c) Kanehisa Laboratories

PROTEIN PROCESSING AND TRANSPORT AT THE ENDOPLASMIC RETICULUM

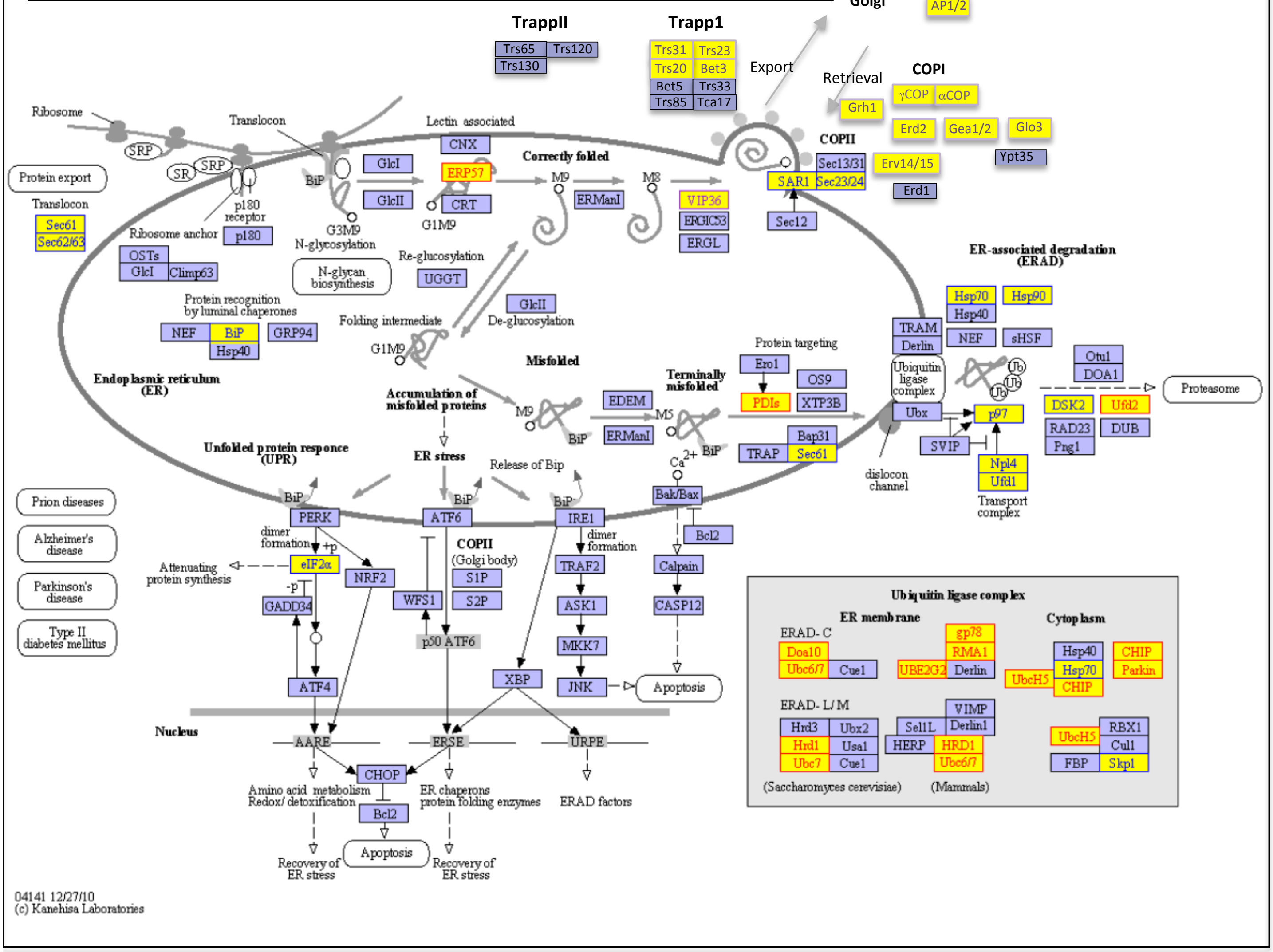

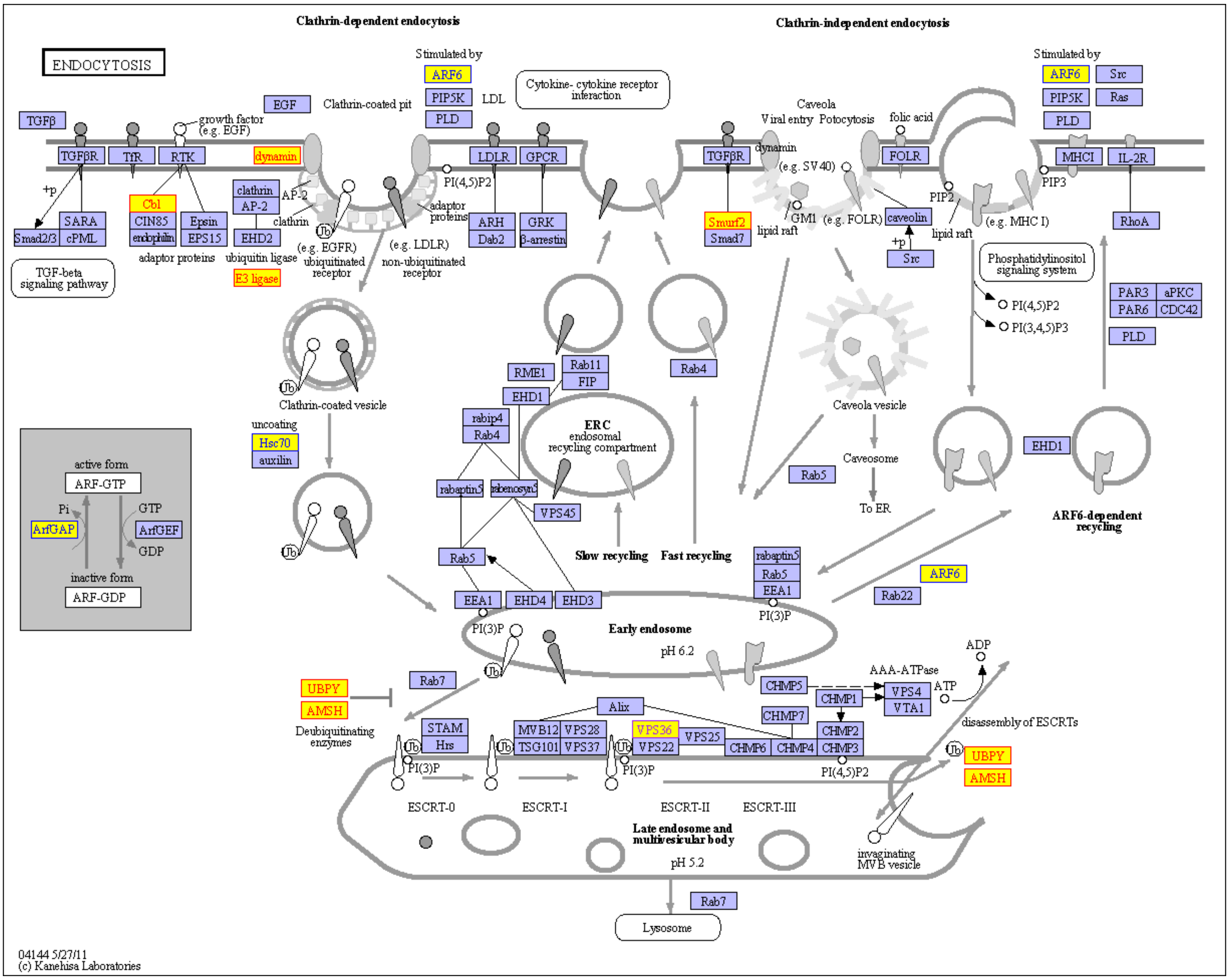

ENDOCYTOSIS
Clathrin-dependent endocytosis
Clathrin-independent endocytosis
Stimulated by
ARF6
PIP5K
PLD
LDL
Cytokine- cytokine receptor interaction
Caveola
Viral entry
Potocytosis
folic acid
Src
Ras
TGFβ
growth factor (e.g. EGF)
EGF
Clathrin-coated pit
TGFβR
TfR
RTK
dynamin
LDLR
GPCR
MHCI
IL-2R
FOLR
(e.g. SV40)
PI(4,5)P2
PIP2
PIP3
+p
Cbl
clathrin
AP-2
SARA
cPML
Smad2/3
CIN85
endophilin
Epsin
EPS15
EHD2
adaptor proteins
ubiquitin ligase
E3 ligase
(e.g. EGFR) ubiquitinated receptor
(e.g. LDLR) non-ubiquitinated receptor
ARH
Dab2
GRK
β-arrestin
Smurf2
Smad7
lipid raft
GM1
(e.g. FOLR)
caveolin
(e.g. MHC I)
RhoA
TGF-beta signaling pathway
Phosphatidylinositol signaling system
PI(4,5)P2
PI(3,4,5)P3
PAR3
aPKC
PAR6
CDC42
PLD
Clathrin-coated vesicle
uncoating
Hsc70
auxilin
RME1
Rab11
FIP
EHD1
rabip4
Rab4
ERC endosomal recycling compartment
rabaptin5
rabenosyn5
VPS45
Rab5
Rab4
Caveola vesicle
Caveosome
To ER
EHD1
ARF6-dependent recycling
active form
ARF-GTP
Pi
GTP
ArfGAP
ArfGEF
GDP
inactive form
ARF-GDP
Slow recycling
Fast recycling
EEA1
EHD4
EHD3
PI(3)P
Early endosome
pH 6.2
Rab22
UBPY
AMSH
Deubiquitinating enzymes
Rab7
Alix
CHMP5
CHMP1
VPS4
VTA1
AAA-ATPase
ADP
ATP
CHMP7
CHMP2
CHMP3
CHMP6
CHMP4
STAM
Hrs
MVB12
VPS28
TSG101
VPS37
VPS36
VPS22
VPS25
disassembly of ESCRTs
ESCRT-0
ESCRT-I
ESCRT-II
ESCRT-III
Late endosome and multivesicular body
pH 5.2
invaginating MVB vesicle
Lysosome
Ub
04144 5/27/11
(c) Kanehisa Laboratories

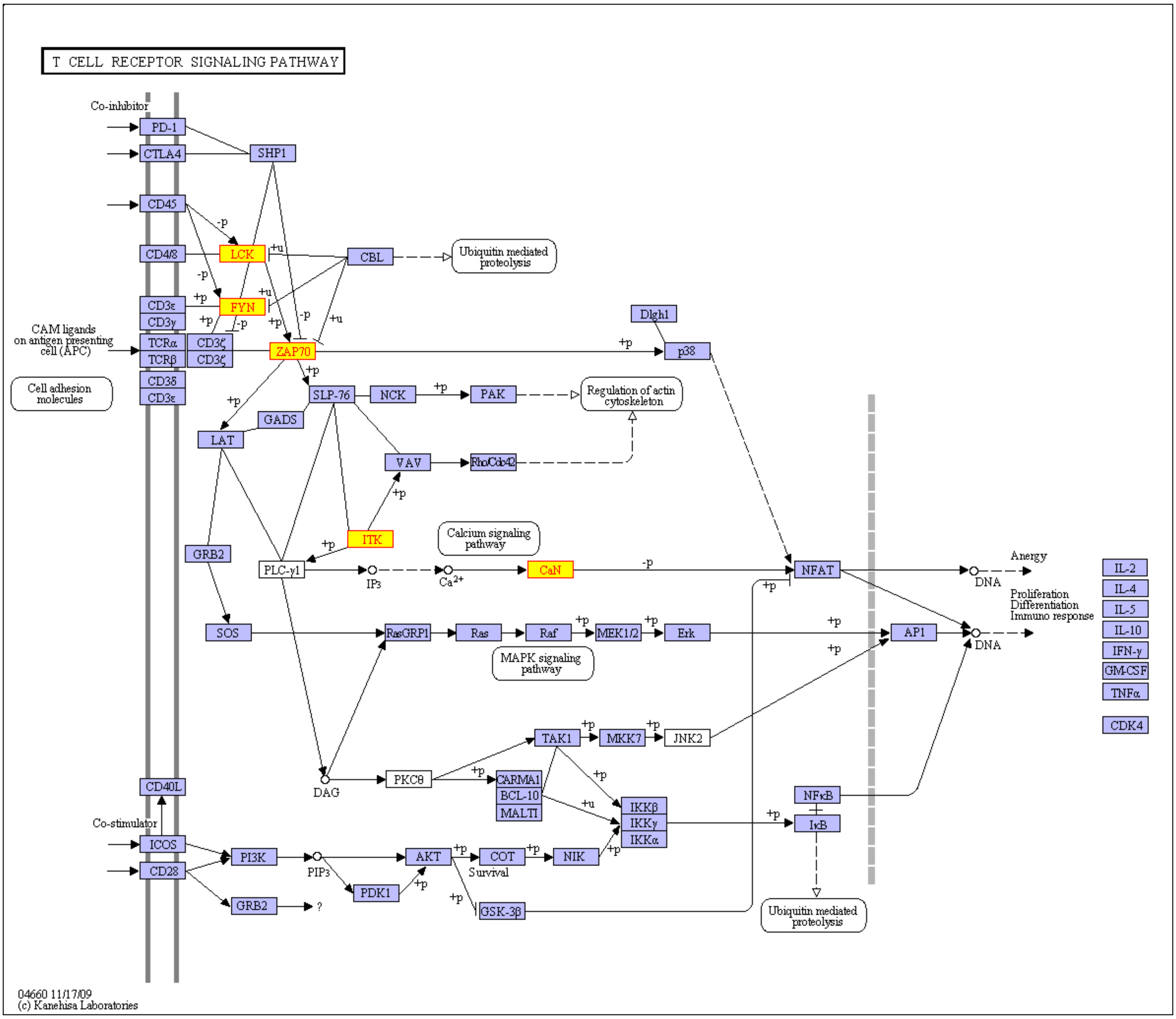

T CELL RECEPTOR SIGNALING PATHWAY
Co-inhibitor
PD-1
CTLA4
SHP1
CD45
CD4/8
LCK
CBL
Ubiquitin mediated proteolysis
CD3ε
CD3γ
FYN
CAM ligands on antigen presenting cell (APC)
TCRα
TCRβ
CD3ζ
CD3ζ
ZAP70
Dlgh1
p38
Cell adhesion molecules
CD3δ
CD3ε
SLP-76
NCK
PAK
Regulation of actin cytoskeleton
GADS
LAT
VAV
Rho/Cdc42
ITK
Calcium signaling pathway
GRB2
PLC-γ1
IP3
Ca2+
CaN
NFAT
DNA
Anergy
Proliferation
Differentiation
Immuno response
SOS
RasGRP1
Ras
Raf
MEK1/2
Erk
AP1
MAPK signaling pathway
TAK1
MKK7
JNK2
DAG
PKCθ
CARMA1
BCL-10
MALT1
IKKβ
IKKγ
IKKα
NFκB
IκB
CD40L
Co-stimulator
ICOS
CD28
PI3K
PIP3
AKT
COT
NIK
Survival
PDK1
GSK-3β
GRB2
Ubiquitin mediated proteolysis
IL-2
IL-4
IL-5
IL-10
IFN-γ
GM-CSF
TNFα
CDK4
04660 11/17/09
(c) Kanehisa Laboratories
