# Supplementary material for: The Genome of the Obligate Intracellular Parasite Trachipleistophora hominis: New Insights into Microsporidian Genome Dynamics and Reductive Evolution
Source: PLoS Pathog. 2012 Oct 25;8(10):e1002979. doi: 10.1371/journal.ppat.1002979 (PMC3486916; doi:10.1371/journal.ppat.1002979)
Supplement: Figure S2 — Sequences of putative introns in selected T. hominis genes. Predicted 5′ and 3′ splice sites (5′SS and 3′SS, respectively), the branch point (pbA) as well as the trinucleotide threshold area (3 nt) are indicated. The relaxed regular expression GTA[AG]GT[ATGC]+?TAATT[ATGC]{0,4}AG was used to identify putative T. hominis introns, following a comparison of T. hominis genes with intron-containing genes from N. ceranae and E. cuniculi (See also Tables S2 and S3). Scavenger is an mRNA decapping enzyme. (PDF) [file ppat.1002979.s002.pdf]

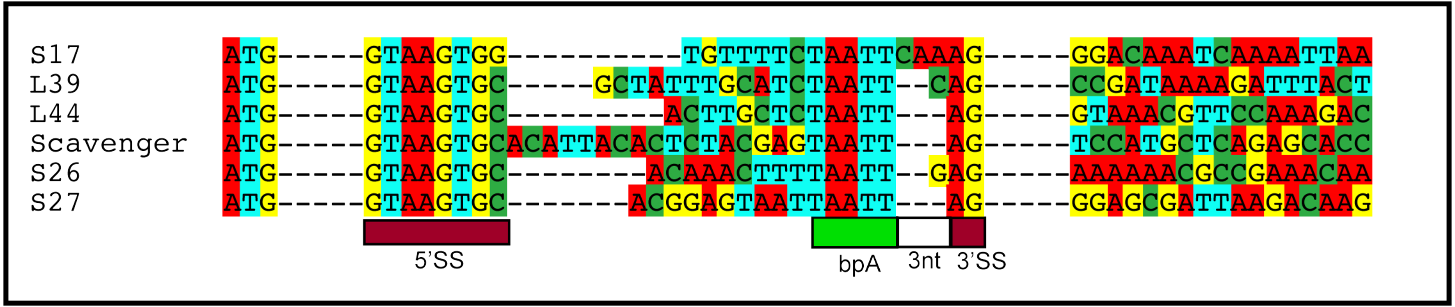
S17 ATG-----GTAAGTGG----------TGTTTTCTAATTCAAAG-----GGACAAATCAAAATTAA
L39 ATG-----GTAAGTGC-----GCTATTTGCATCTAATT--CAG-----CCGATAAAAGATTTACT
L44 ATG-----GTAAGTGC---------ACTTGCTCTAATT---AG-----GTAAACGTTCCAAAGAC
Scavenger ATG-----GTAAGTGCACATTACACTCTACGAGTAATT---AG-----TCCATGCTCAGAGCACC
S26 ATG-----GTAAGTGC--------ACAAACTTTTAATT--GAG-----AAAAAACGCCGAAACAA
S27 ATG-----GTAAGTGC--------ACGGAGTAATTAATT---AG-----GGAGCGATTAAGACAAG
5'SS
bpA
3nt
3'SS
