# Supplementary material for: The Genome of the Obligate Intracellular Parasite Trachipleistophora hominis: New Insights into Microsporidian Genome Dynamics and Reductive Evolution
Source: PLoS Pathog. 2012 Oct 25;8(10):e1002979. doi: 10.1371/journal.ppat.1002979 (PMC3486916; doi:10.1371/journal.ppat.1002979)

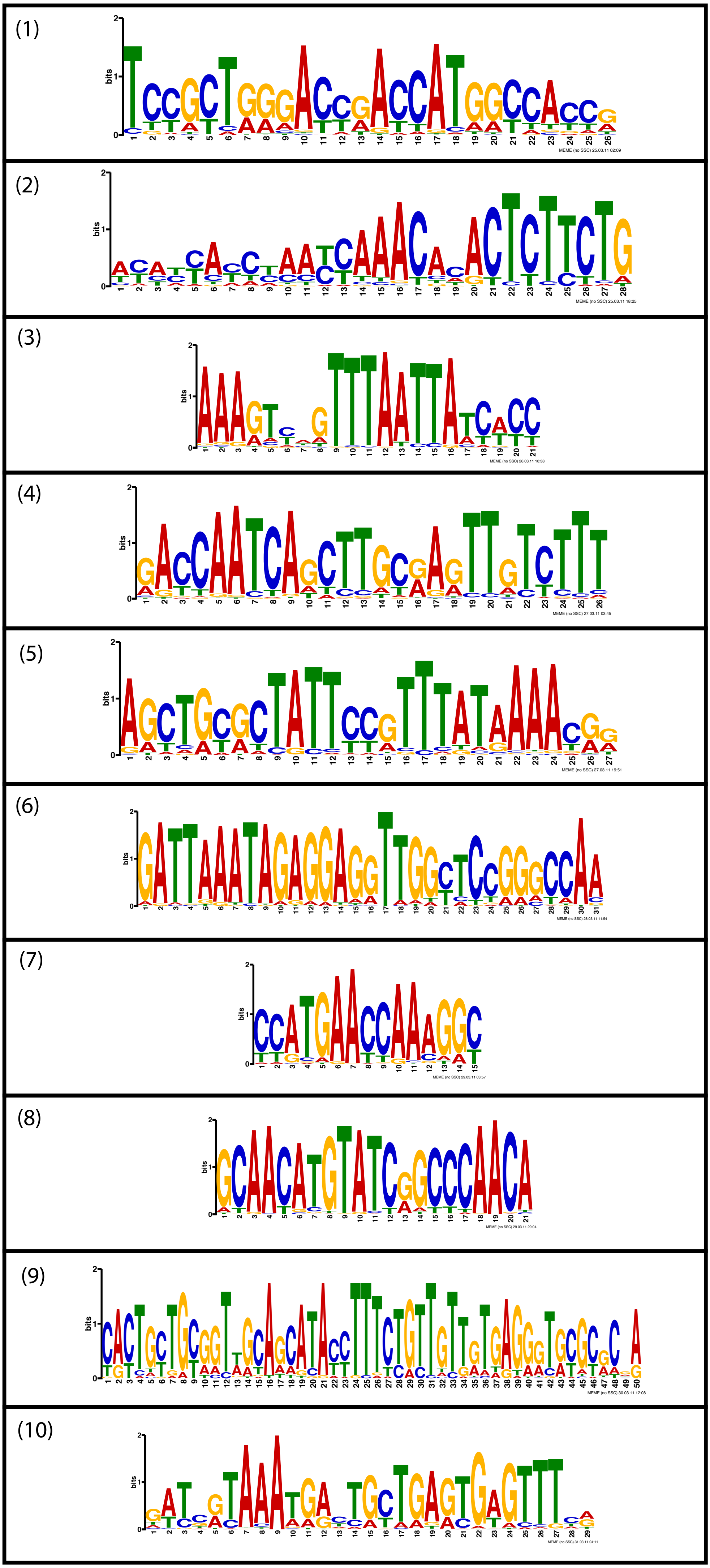
(1)
(2)
(3)
(4)
(5)
(6)
(7)
(8)
(9)
(10)

(11)

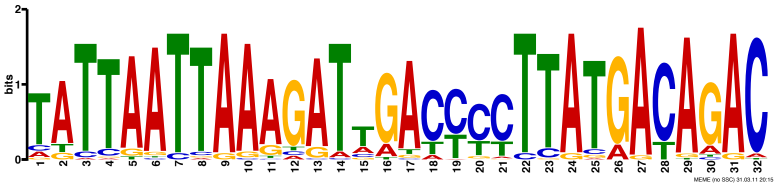

(12)

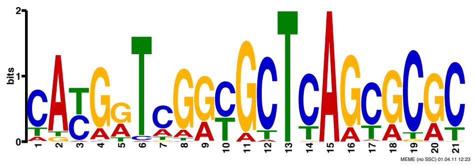

(13)

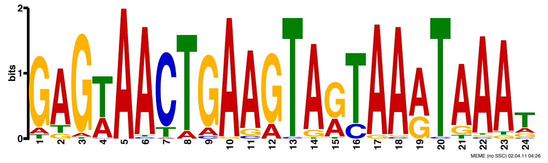

(14)

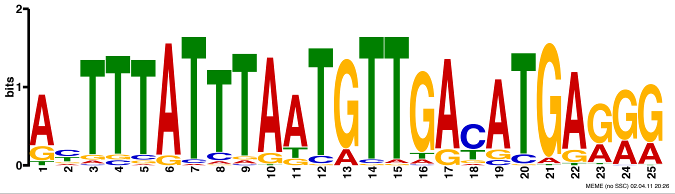

(15)

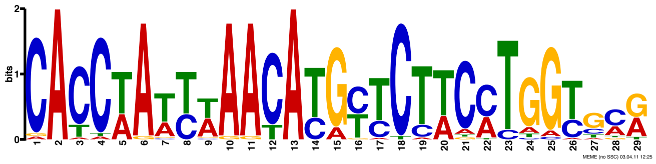

(16)

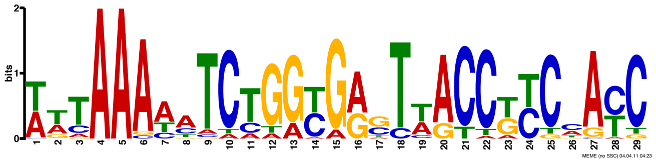

(17)

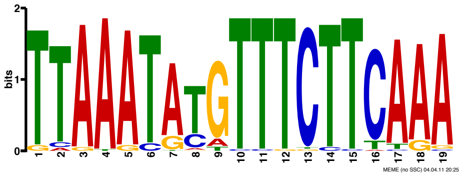

(18)

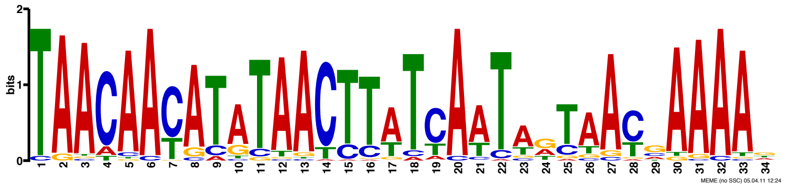

(19)

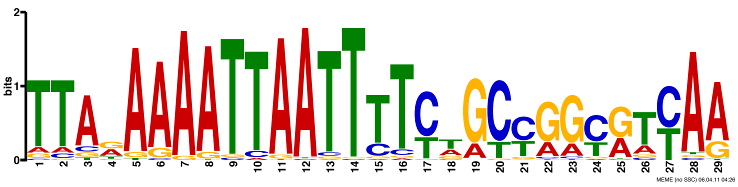

(20)

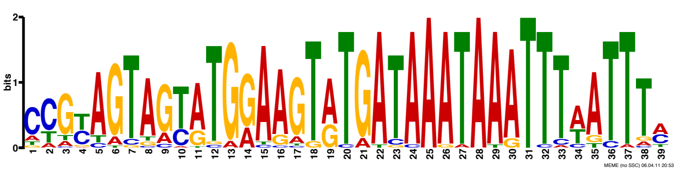

(21)

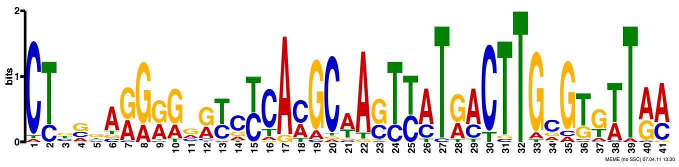

(22)

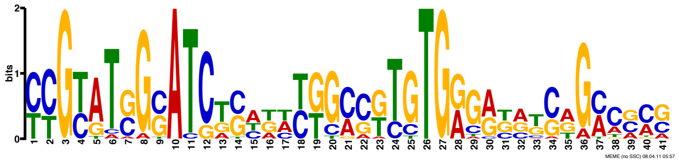

(23)

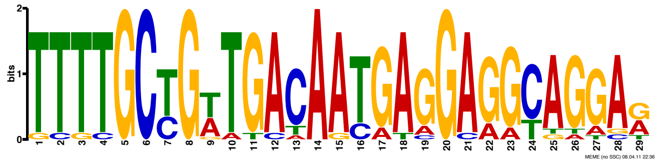

(24)

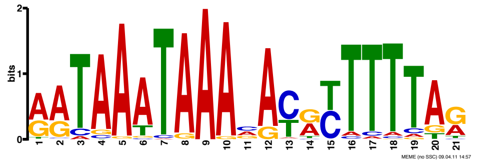

(25)

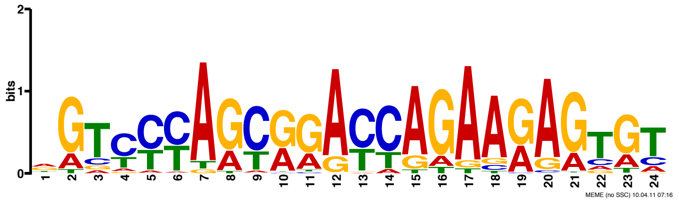

(26)

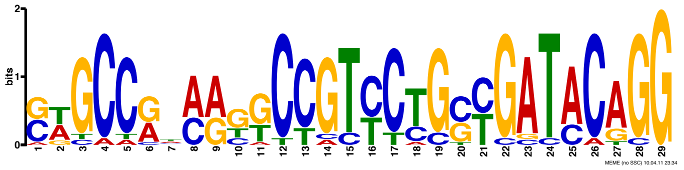

(27)

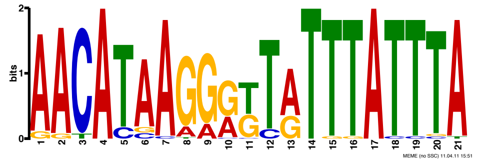

(28)

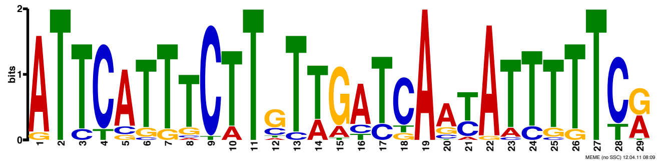

(29)

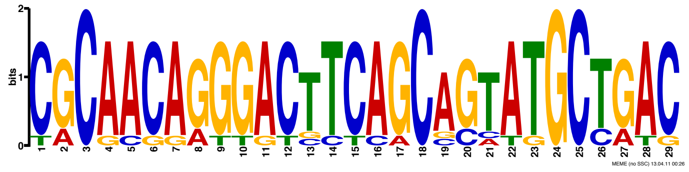

(30)

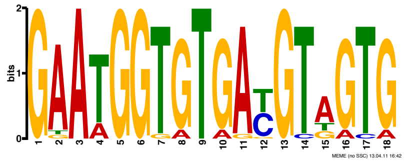

(31)

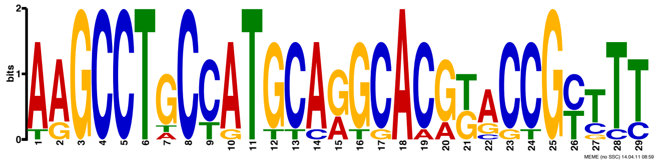

(32)

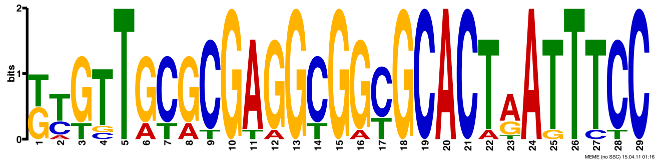

(33)

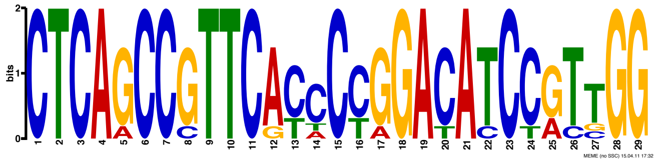

(34)

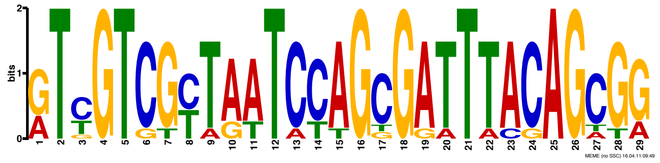

(35)

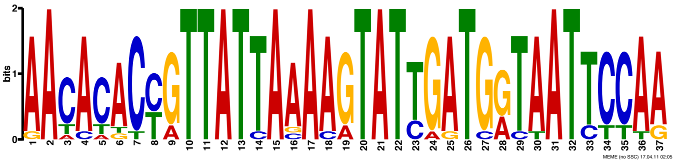

(36)

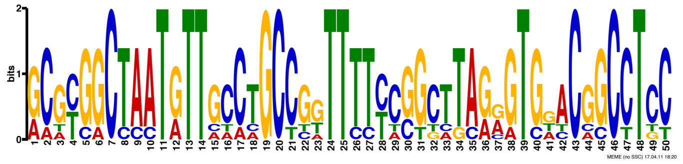

(37)

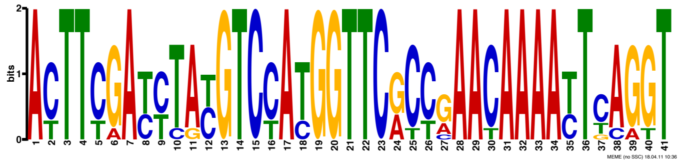

(38)

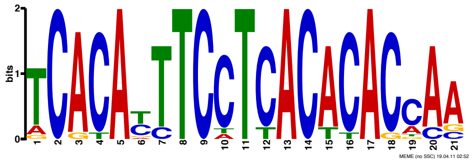

(39)

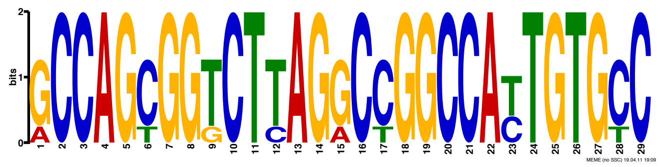

(40)

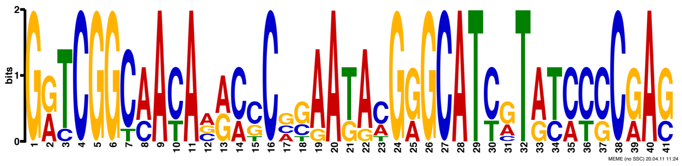

Supplement: Figure S3 — Identification of 40 potential regulatory motifs in the non-coding regions of the T. hominis genome. Details on the motifs and their similarity to known motifs are given in Tables S4 and S5. (PDF) [file ppat.1002979.s003.pdf]
