# Supplementary figures and images for: The Genome of the Obligate Intracellular Parasite Trachipleistophora hominis: New Insights into Microsporidian Genome Dynamics and Reductive Evolution
Source: PLoS Pathog. 2012 Oct 25;8(10):e1002979. doi: 10.1371/journal.ppat.1002979 (PMC3486916; doi:10.1371/journal.ppat.1002979)

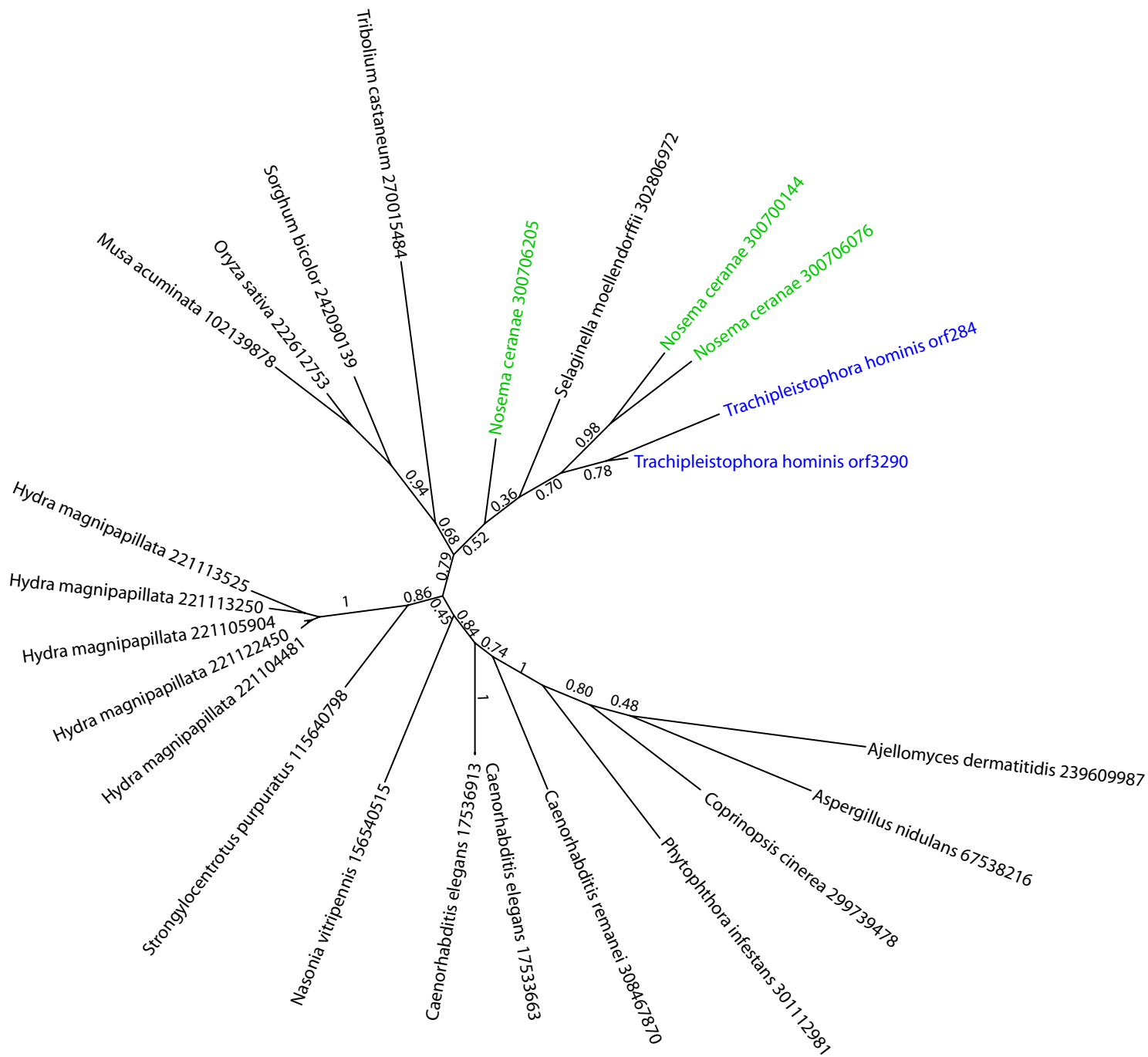

0.2

Supplement: Figure S4 — Relationships of helitron sequences from T. hominis and N. ceranae to those from other eukaryotes. The weakly supported tree favours a common origin for N. ceranae and T. hominis helitron elements, suggesting that helitrons were present in their common microsporidian ancestor, but does not confidently identify their closest relatives among other eukaryotic helitrons. The tree shown is from a Bayesian analysis of Dayhoff-recoded amino acid sequences performed using p4 and the node discrete compositional heterogeneity model ([123], NDCH) with two base composition vectors needed to fit the data; support values are Bayesian posterior probabilities. The tree was calculated over 2 million generations using the ‘auto-tune’ setting, and compositional fit was tested as previously described ([123]. The accession number (gi) or the T. hominis ORF locus tag are given for each sequence in the tree. (PDF) [file ppat.1002979.s004.pdf]

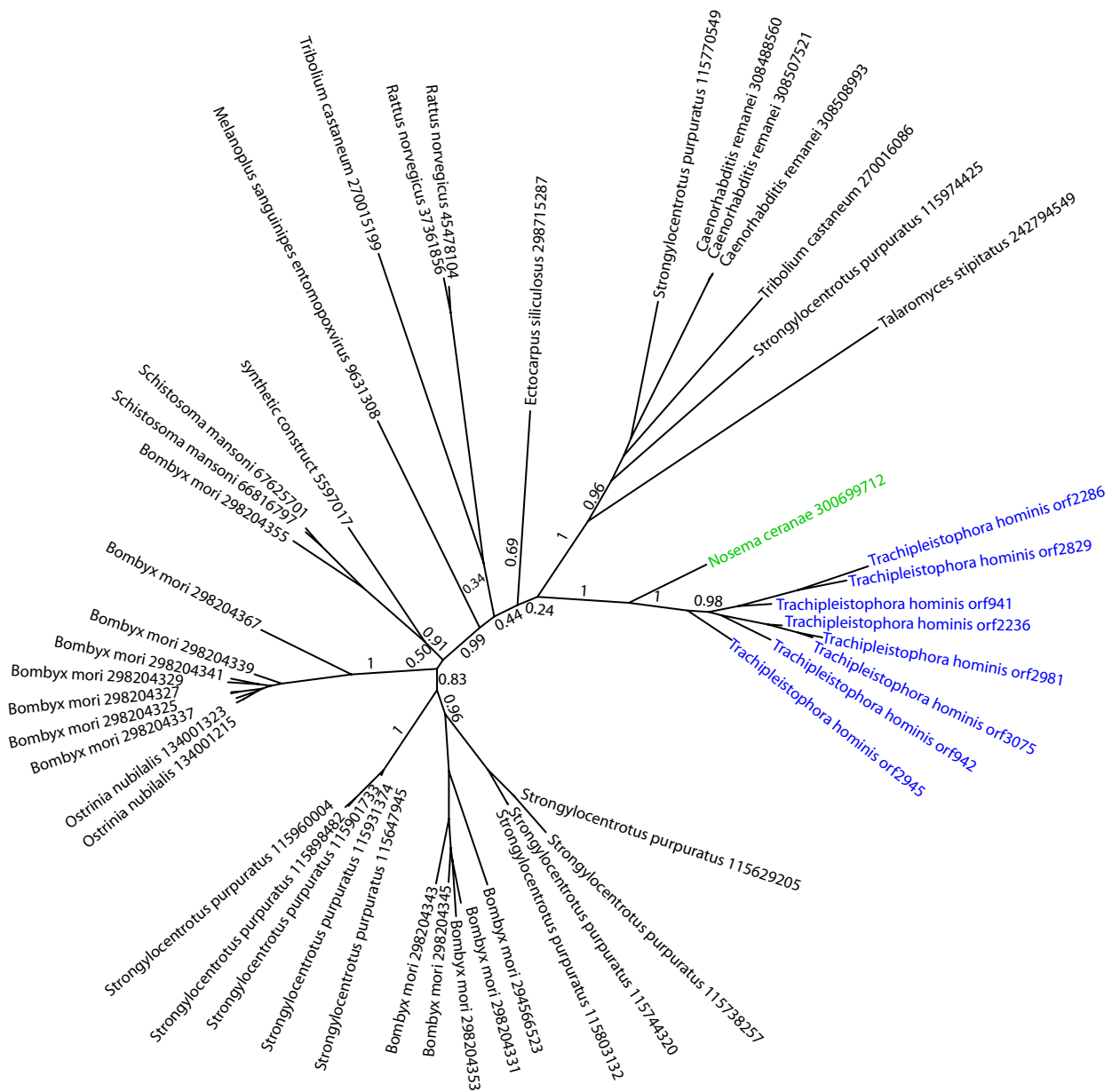

0.2

Supplement: Figure S5 — Relationships of non-LTR sequences from T. hominis and N. ceranae to those from other eukaryotes. The tree strongly supports the monophyly of N. ceranae and T. hominis non-LTR elements, suggesting that these elements were present in their common microsporidian ancestor. The topology also demonstrates that expansion of non-LTR elements has occurred in T. hominis. The tree shown is from a Bayesian analysis of Dayhoff-recoded amino acid sequences performed with p4 using the NDCH model with two base composition vectors. Further details of the analysis are given in the legend to Figure S4. The accession number (gi) or the T. hominis ORF locus tag are given for each sequence in the tree. (PDF) [file ppat.1002979.s005.pdf]

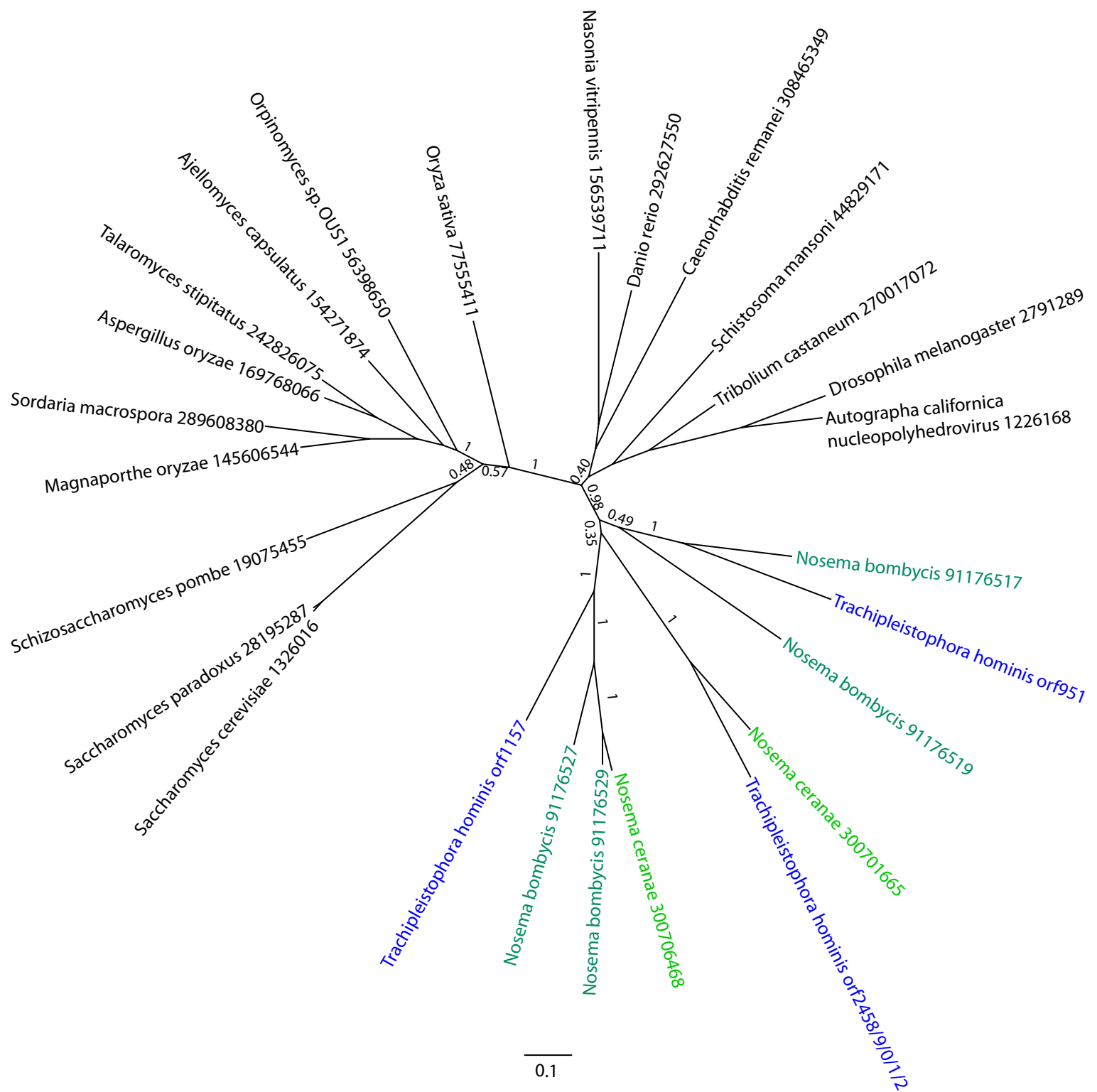

Supplement: Figure S6 — Relationships of microsporidian LTR sequences to those from other eukaryotes. The tree strongly supports the monophyly of N. ceranae, N. bombycis and T. hominis LTR elements, suggesting that these elements were present in their common microsporidian ancestor. The tree shown is from a Bayesian analysis of Dayhoff-recoded amino acid sequences performed with p4 using the NDCH model with two base composition vectors. Further details of the analysis are given in the legend to Figure S4. The accession number (gi) or the T. hominis ORF locus tag are given for each sequence in the tree. (PDF) [file ppat.1002979.s006.pdf]

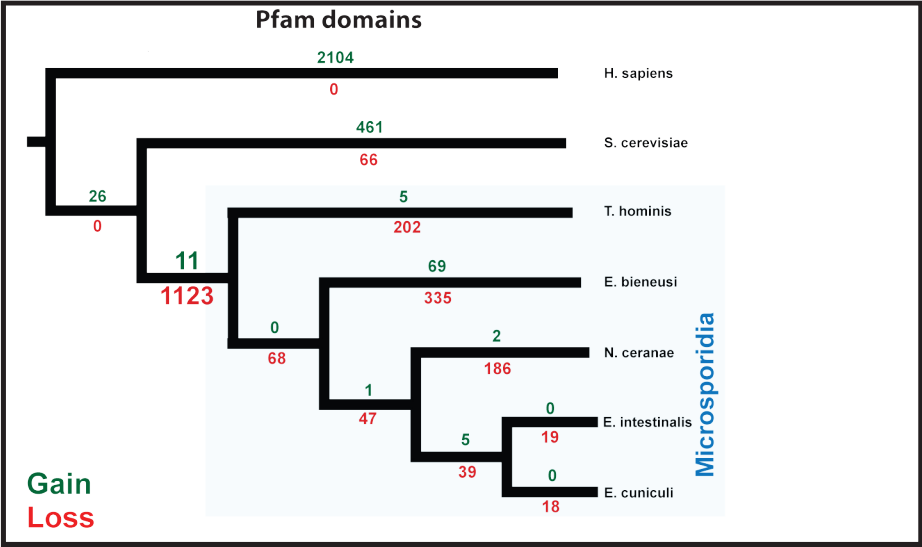

Supplement: Figure S8 — Gain and loss of Pfam domains during microsporidian evolution. Gain and loss of Pfam domains were plotted onto the cladogram using Dollo parsimony. The numbers indicated on the branches show the number of Pfam domains inferred to have been lost (red) or gained (green). (PDF) [file ppat.1002979.s008.pdf]

***S. cerevisiae***

5,886

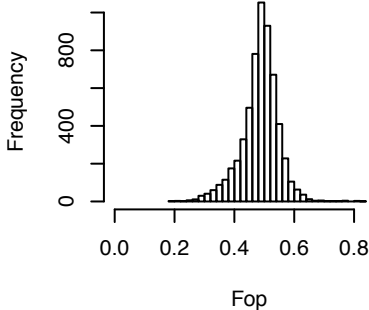

***T. hominis***

3,167

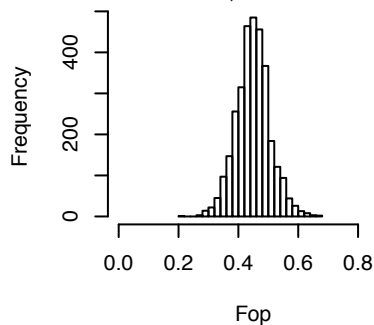

***N. ceranae***

2,060

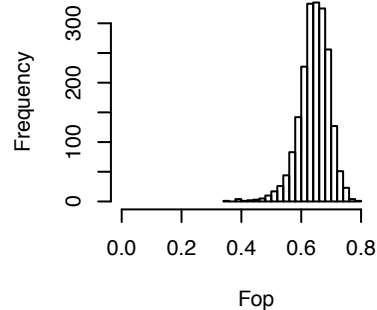

***E. cuniculi***

1,996

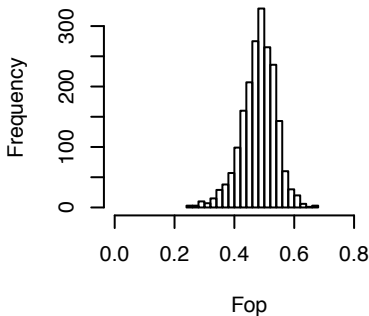

***E. intestinalis***

1,832

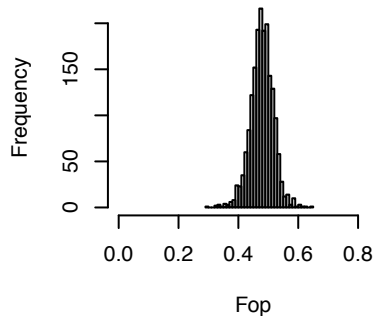

***E. bieneusi***

3,633

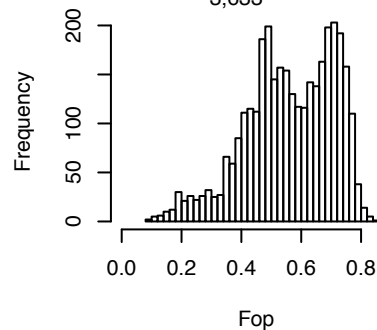

Supplement: Figure S9 — Synonymous codon usage in S. cerevisiae and the five sequenced microsporidian genomes. Per-gene codon usage was quantified using the FOP score (frequency of optimal codons) [39], which measures for each gene the proportion of amino acids encoded by the “optimal” (most frequent) codon, determined for the respective amino acid over the whole genome. Choice of synonymous codon usage is characteristic of a genome, often resulting in a unimodal distribution of the FOP score. The biased distribution for N. ceranae towards higher FOP scores reflects the reduction of codon usage variation in this genome as a function of very low G+C content (27.2%, vs. 40.3%+/−5.5% standard deviation for the other species [133]). The broad bimodal shape of the FOP distribution for E. bieneusi suggests heterogeneity of codon usage for which one possible explanation is contamination of the data (see main text for discussion). (PDF) [file ppat.1002979.s009.pdf]

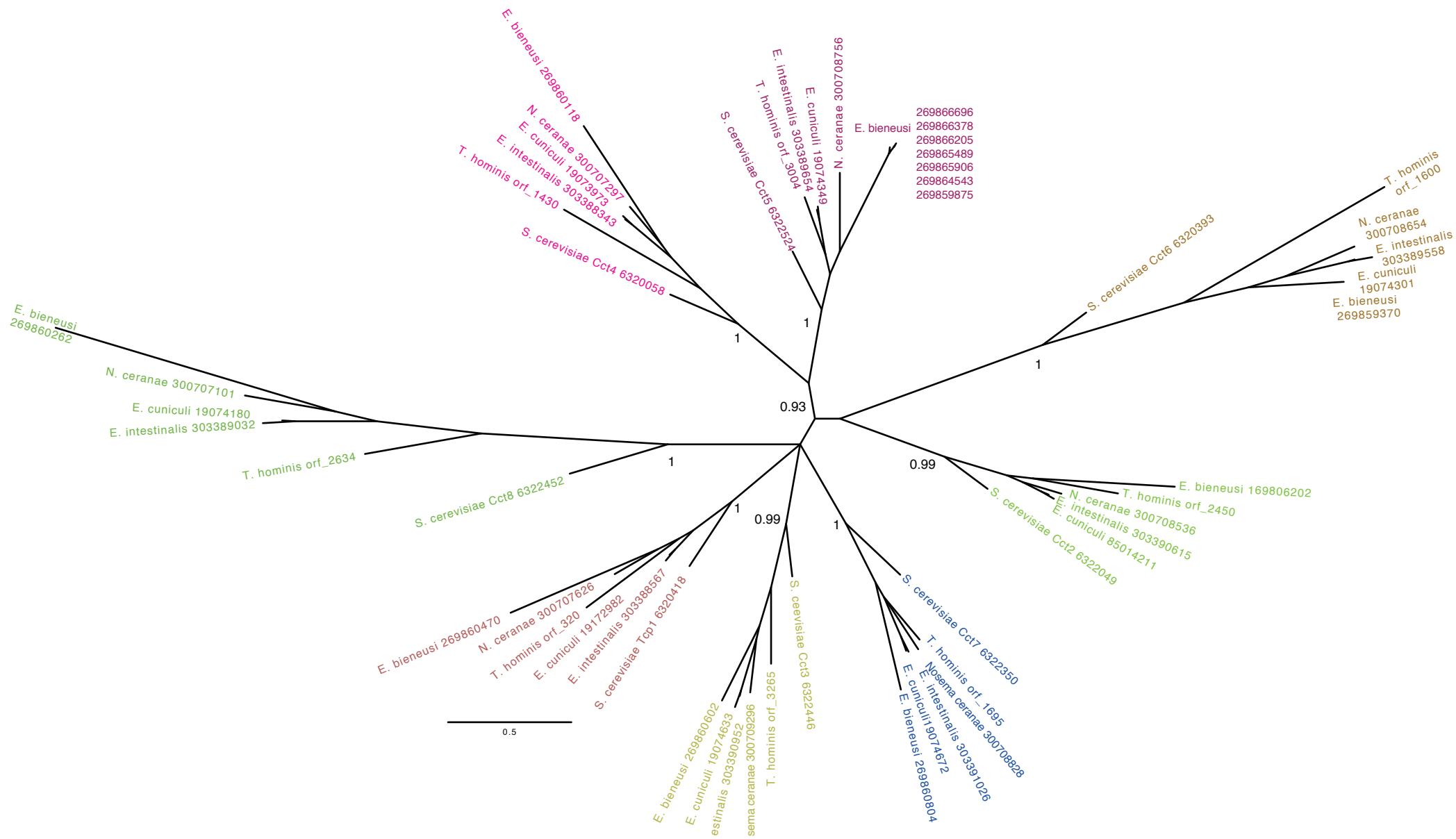

Supplement: Figure S10 — Phylogeny of TriC/CCT (chaperonin) genes in the microsporidia. All of the microsporidians have retained each of the eight TriC/CCT (cytosolic chaperonin) subunits. Support values are given as Bayesian posterior probabilities. The phylogeny was built using the CAT20 model in PhyloBayes. (PDF) [file ppat.1002979.s010.pdf]

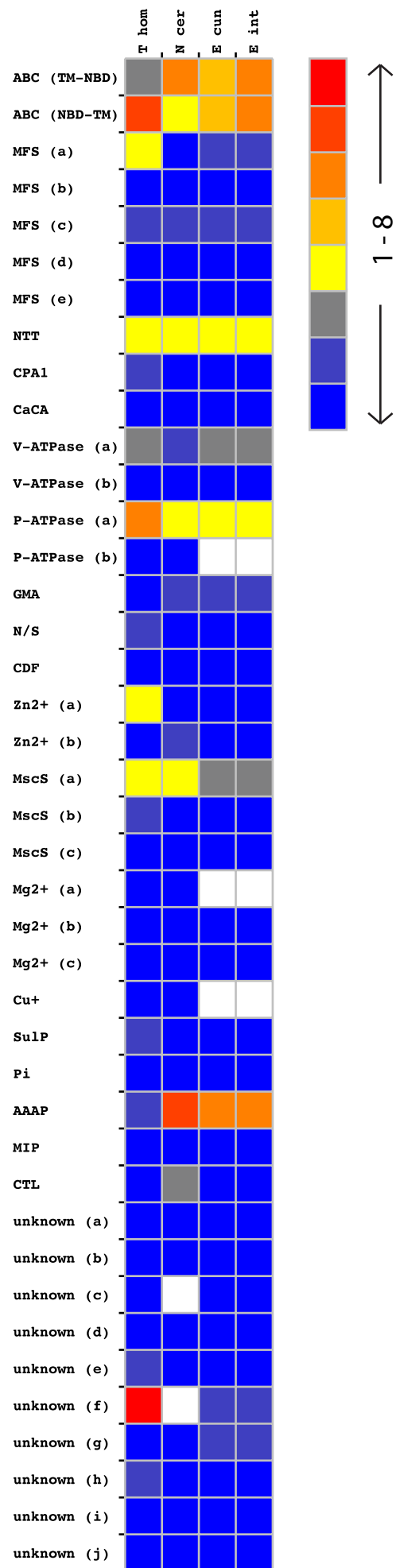

Supplement: Figure S11 — The number and types of different transport proteins in T. hominis and the other microsporidians investigated. The figure shows the different transporter protein families and the number of individual proteins in each family (MCL cluster) for each microsporidian genome investigated. The key for the abbreviated transporter names is provided in Table S14, where details of the manual annotation are also described. The number of proteins within each cluster for each species is indicated by a colour ranging from 1 protein (blue) to 8 proteins (red). An empty box indicates that no family member was detected. (PDF) [file ppat.1002979.s011.pdf]

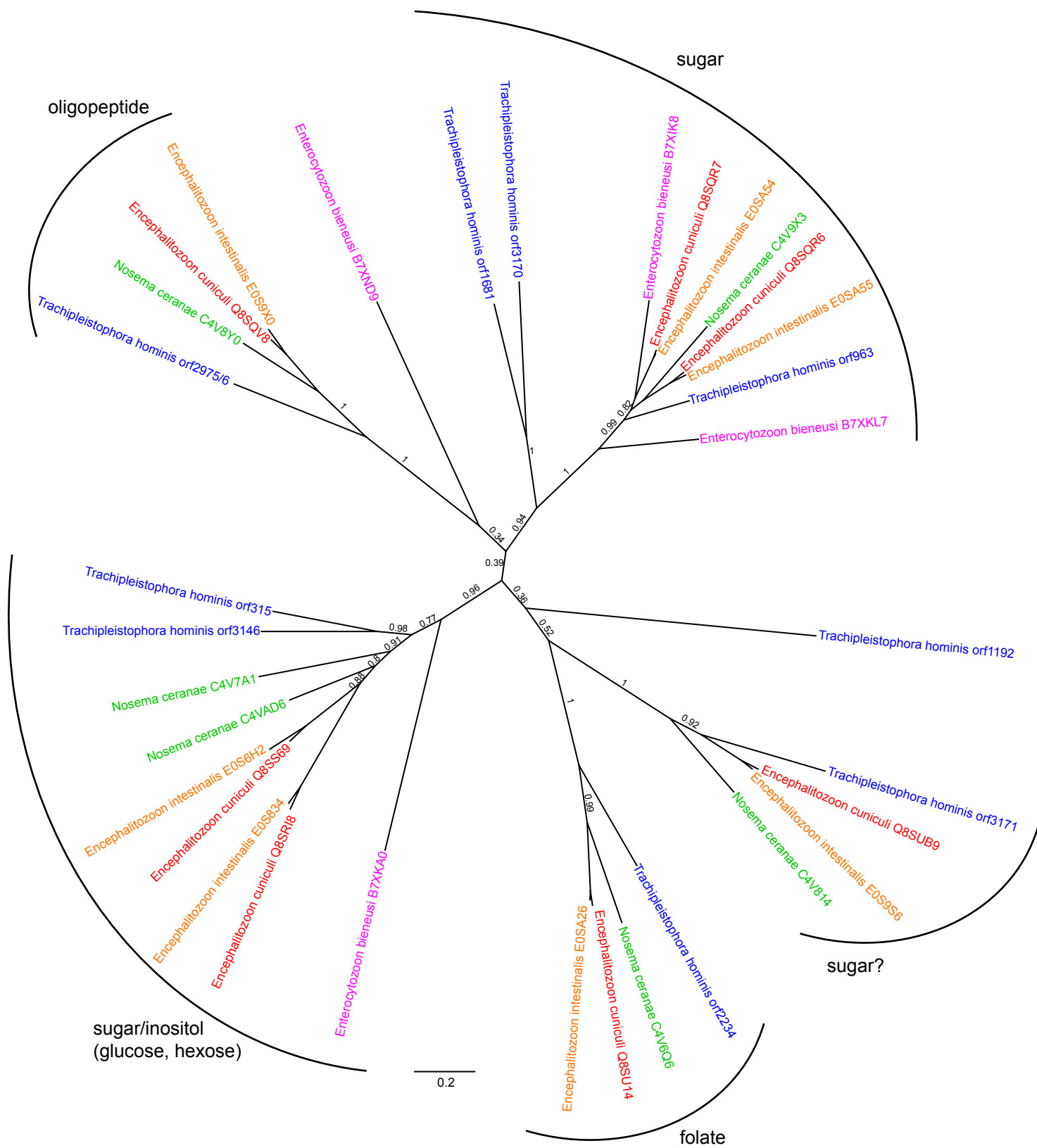

Supplement: Figure S12 — Phylogenetic analysis of microsporidian MFS transporters. Each species has retained at least one member of each MFS subgroup, suggesting that MFS transporters are functionally important for microsporidians. The tree was calculated with p4 on a Dayhoff-recoded dataset using two base composition vectors and were calculated for 2 million generations as described in Figure S4, support values are given as Bayesian posterior probabilities. Putative substrates are indicated; details of the evidence supporting these inferences are given in Table S14. (PDF) [file ppat.1002979.s012.pdf]

A

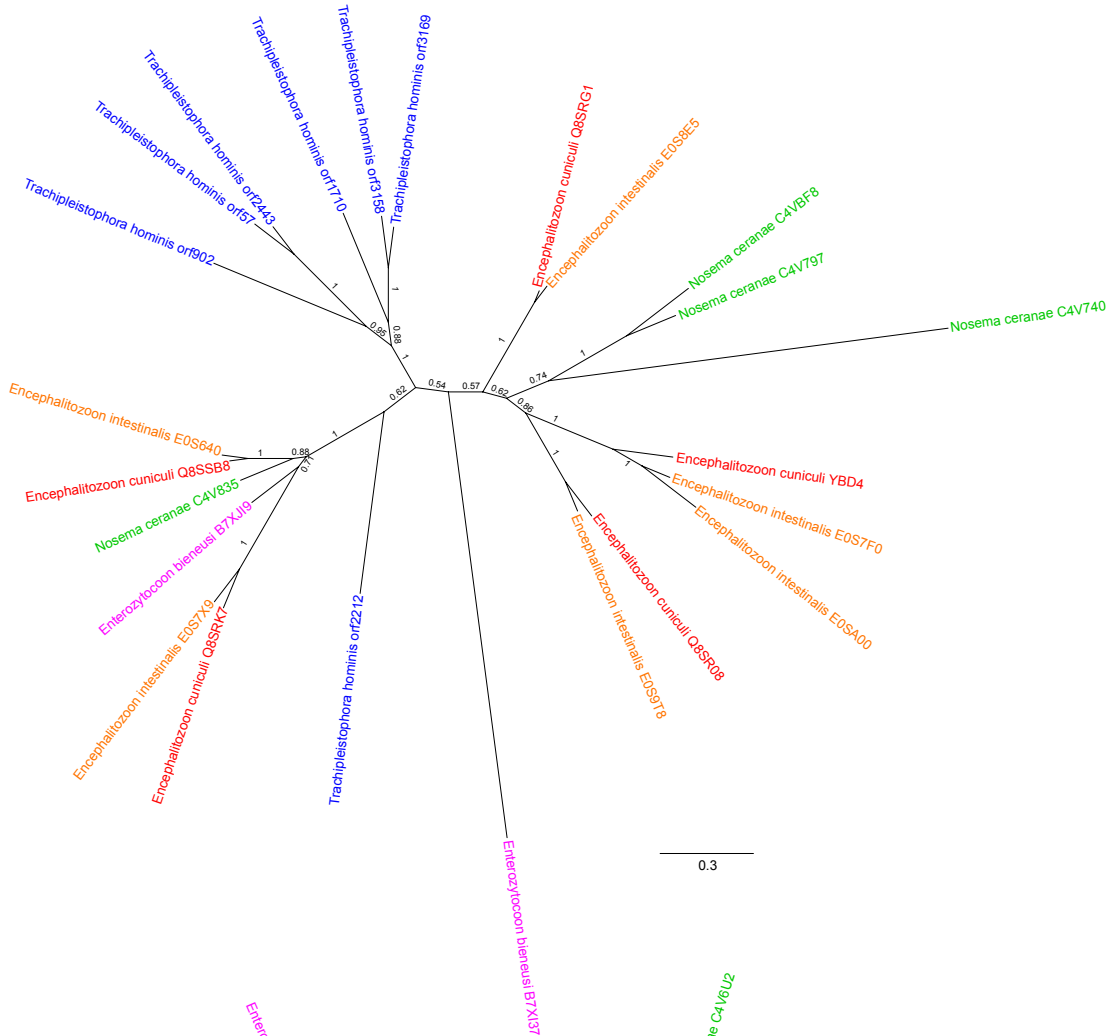

B

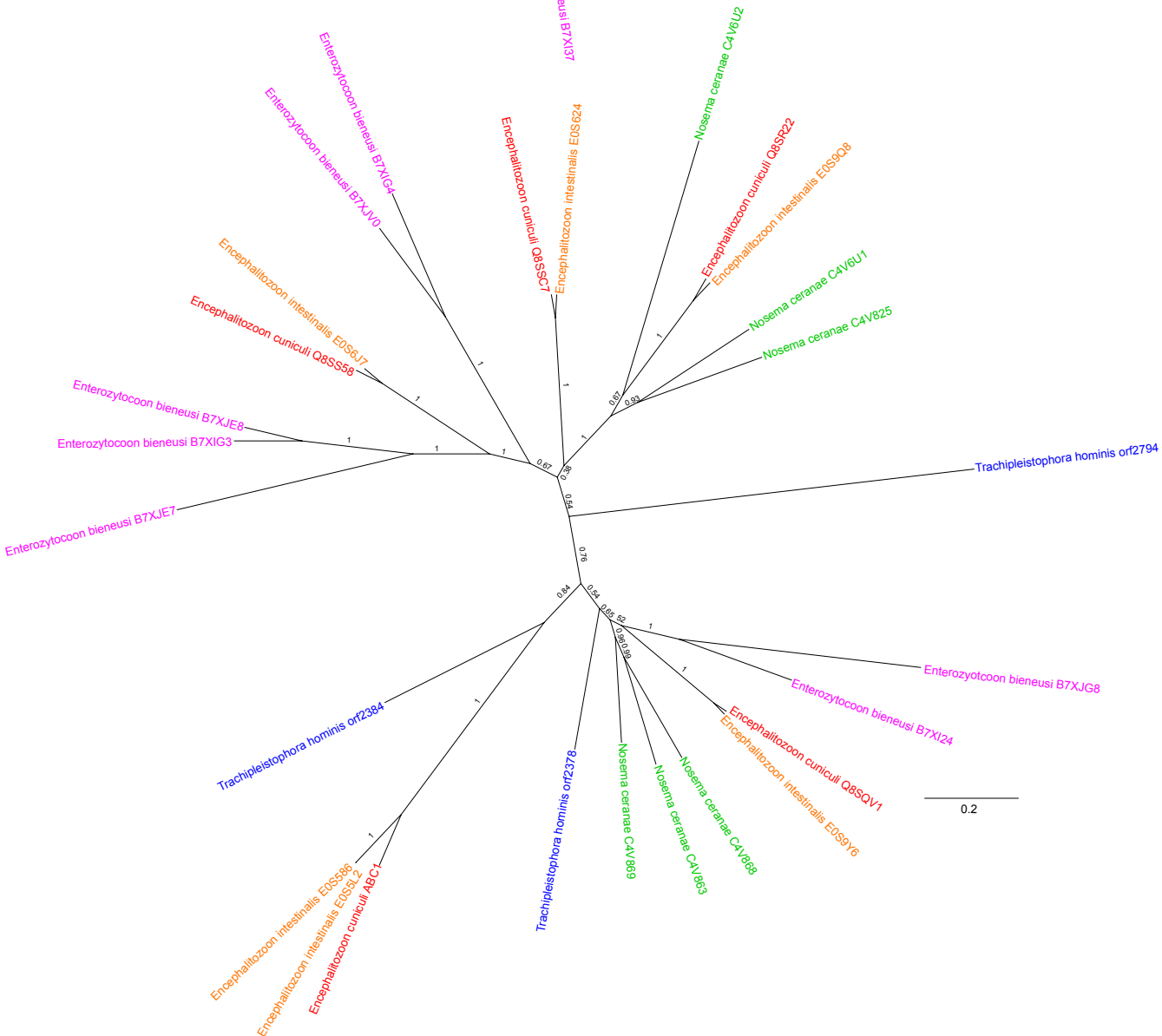

Supplement: Figure S13 — Phylogenetic analysis of microsporidian ABC transporters. To increase the number of positions available for phylogenetic analyses, the transporters were split into two groups based on their different domain organisations (A) N-terminus - TMD - nucleotide binding domain (NBD) - C-terminus and (B) N-terminus - NBD - TMD - C-terminus. These trees reveal lineage-specific duplications among microsporidians, with an apparent expansion of the transporters with topology (A) in T. hominis. The trees were calculated with p4 on a Dayhoff-recoded dataset using 7 base composition vectors and were calculated for 2 million generations as described in Figure S4, support values are given as Bayesian posterior probabilities. Details on similarities to other ABC transporters as well as their potential functions are given in Table S14. (PDF) [file ppat.1002979.s013.pdf]

**A**

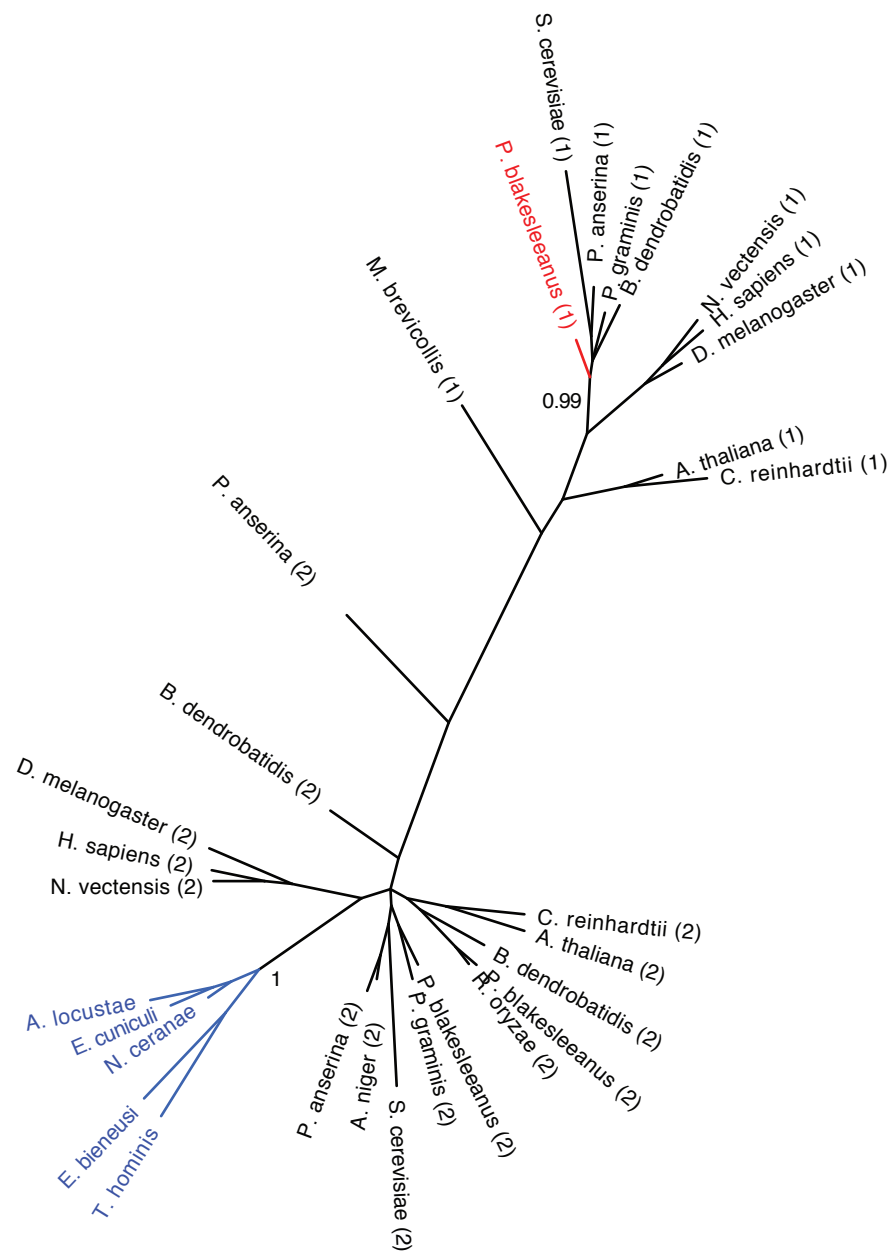

**B**

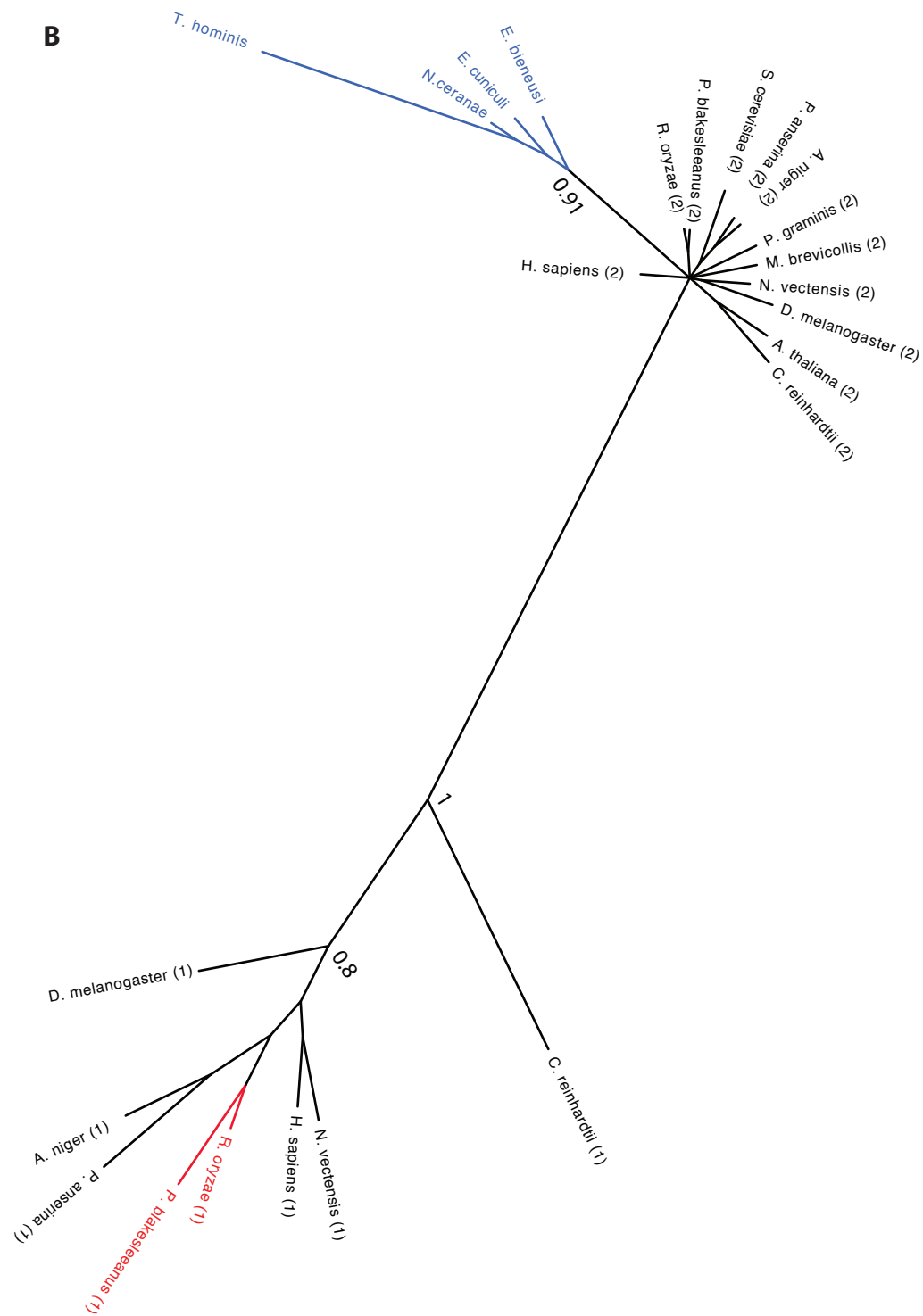

Supplement: Figure S15 — Phylogenies of the triosephosphate transporter (TPT) and the RNA helicase (Hel) genes that form part of the syntenic sex locus in Zygomycetes. Zygomycete sex locus sequences in red; microsporidian sequences in blue. The tree topologies for TPT (A) and Hel (B) suggest that the observed synteny between these sequences in zygomycete fungi, E. cuniculi and E. bienieusi is due to convergence. In the case of both genes, the zygomycete and microsporidian sequences are paralogs, related via a gene duplication that occurred early in eukaryotic evolution, confirming the analyses of Koestler and Ebersberger [74]. Sequences on either side of these duplications are denoted by (1) and (2). The phylogenies are consistent with the observation that these genes are not syntenic in T. hominis. The sequences are those used in Koestler and Ebersberger [74], with the addition of the respective T. hominis sequences. (PDF) [file ppat.1002979.s015.pdf]

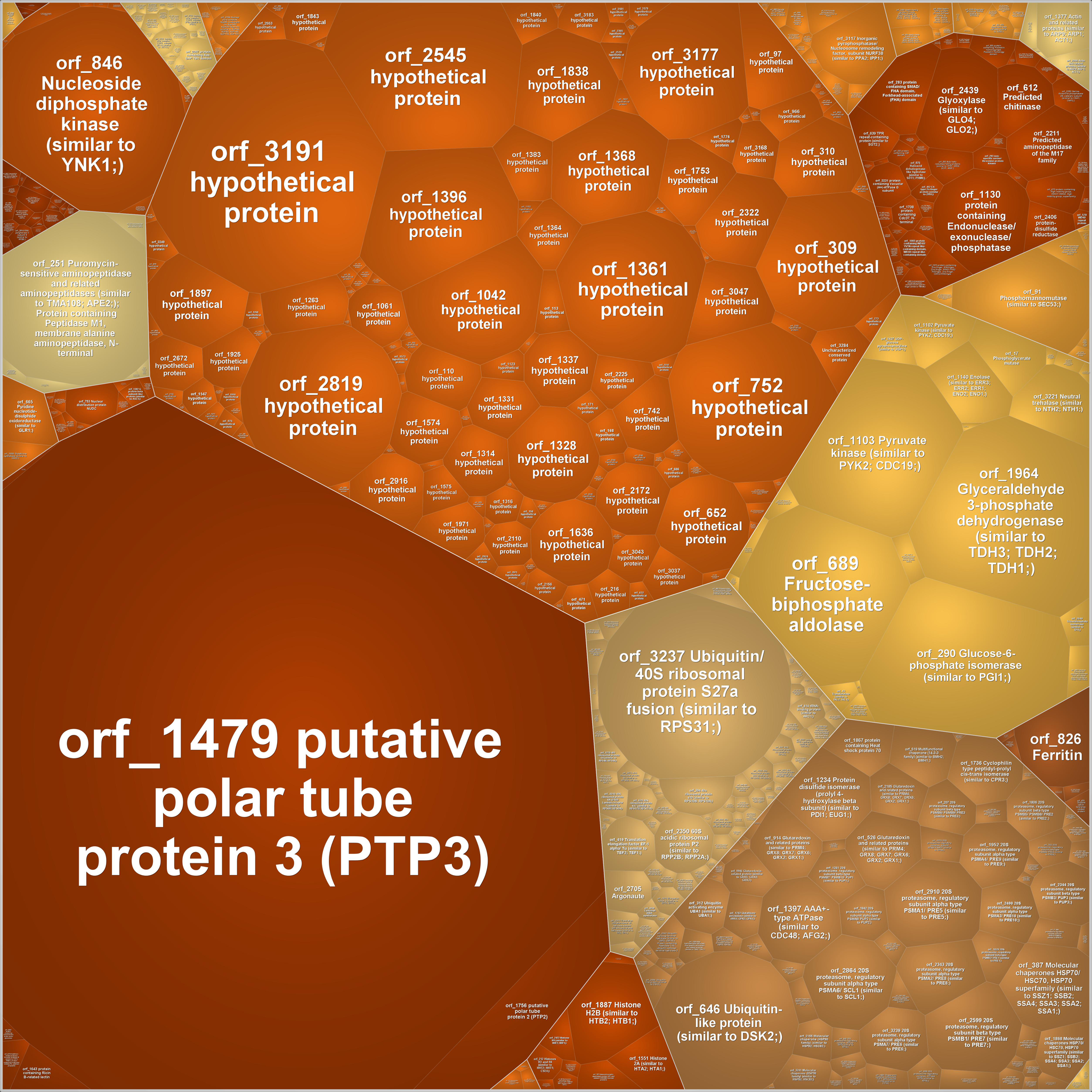

Supplement: Figure S16 — The same Voronoi treemap shown in Figure 7 showing the corresponding locus tags and protein annotation of the individual proteins identified. The area of individual cells is proportional to the semi-quantitative spectral counts of each individual protein. (TIF) [file ppat.1002979.s016.tif]
