# Supplementary material for: The Genome of the Obligate Intracellular Parasite Trachipleistophora hominis: New Insights into Microsporidian Genome Dynamics and Reductive Evolution
Source: PLoS Pathog. 2012 Oct 25;8(10):e1002979. doi: 10.1371/journal.ppat.1002979 (PMC3486916; doi:10.1371/journal.ppat.1002979)

A

```
T. hominis               -----------------------------------------MHVHPQDRNVIRII-------------------------
S. hominis SK119         --------------------------------------------------MARIW-------------------------
S. hominis C80           --------------------------------------------------MARIW-------------------------
P. donghaensis           -----------------------------------------MNDQPKRKKVSTYR-------------------------
S. pseudintermedius      --------------------------------------------------MKRFW-------------------------
S. aureus                ------------------------------------------------MKNTKNW-------------------------
A. thaliana              -------MILGRWVSFSCGNTPVTNCSERRRHTEFRRLSSASTCRPSLICSCLKSKS-SQETTQIEQLGNGEGFSVLASE
G. max                   MLSVLVCPSHELATPFQCLHFPNTDYSL--KCSPKLQLGSTPSQRNPFKCFCNRKEHVTPFSQ--------GFSELQEDD
O. tauri                 ------------------MRVTHSRAQIRPRQLNFHYQSSHARARRSSTRFTRSS-------------------------

T. hominis               -------SVALLTFIL--------LGVIKGV---LLIQESIGKKINTIFMI-LEDFSLY--SCSFLVYFLYNNMLKTDF-
S. hominis SK119         --------VAILTLIIYALAQFLPLVLAQTPI--FSRLSGMALARAGVYTQ-VILFILA---AILIIFFHFKIKNPTNLE
S. hominis C80           --------VAILTLIIYALAQFLPLVLAQTPI--FSRLSGMALARAGVYTQ-VILFILA---AILIIFFHFKIKNPTNLE
P. donghaensis           -PKSKGKKTPLYVLLIFIAAQLSPILFISPTMS-YFQGQGMDRQAAGIATSGWLIFLTMGIGFLLTLIIVSRDKRFFDIW
S. pseudintermedius      --------VSLLTVVLYILAQISPYIARAVGW--IQTGNQAQLLRQSIAVQ-FTAFIIV---AILIIILQIFVKNKLNFE
S. aureus                -------YESLFSGVYDGLKISFPLVLIYLIAY-IHDSLSFNPSINVIRWS-FGVIVST-----LIFFLIFKFKFF----
A. thaliana              IPWEDDNIWSTFALYMFSLHIPLSFGGLSIVAN-ILHRQVLDPQTQVLSLV-VLQMVELAGTVLLLRTTAKPQCKSINFL
G. max                   SPWESGNVWSNLALYLFTLHIPFSFGGLSVVAL-FNGQPVLDPQTEALSLL-TIQILEFGGALVLLEYIAKPQYKFSNFS
O. tauri                 -----GERVDANVLNA-SAKFAYAWVTLGYVAPSALASGRLGETSDVEYLAIVALTCET-----IKAYATYRVVNDSNDD

T. hominis               ---------KKTWLYKYR---LCAIICFDIVLSQYFVFIGNL-KIPLIANNGQSD---------SASVGDVAKLARIFTL
S. hominis SK119         QEHKESKRYIIPWA----------ILGFFIV-MLYQVIVGVI-NIWIFGQPQQSP----NTQRIMAVAKQL--PIFIILI
S. hominis C80           QEHKESKRYIIPWA----------ILGFFIV-MLYQVIVGVI-NIWIFGQPQQSP----NTQRIMAVAKQL--PIFIILI
P. donghaensis           KGKKSSLLMSIVWG----------FLGFLLL-LIGQSIAALI-EMNFLGIEPGSE----NTASLVSIAEVV--PLAIVSI
S. pseudintermedius      LGPKEKKRYVLPYI----------LVGLVIV-FIAQMVVNLI-SVQLFGANPASE----NTLRIMEIARQM--PIFIVLI
S. aureus                ---------AKDWKLTFTNKSFLIVIGSSIL---INLIGYFQNVFDLYKESKNQK----IIEEKLQNNQDHL-HFYFVDM
A. thaliana              KGN-NETREGRNWV-------VGSALGLGCL-VGFIFVTSLV-ADQLFGPKAVHE----SELEKIMVSGEVARSGCFALY
G. max                   RKNK--LLSNRNWF-------LSSAVGFGFL-VLLIFLTSLL-ADRLFGSKPVNN----PILKDMLLNSDISRLSCVLAY
O. tauri                 ----VLRDAVEGWASGRA---VLDGVAFGAVACVCARVVDGA-SAMLFGASDGIDKIDTAALATSADGGALATSAAAIAA

                                 EEXXXR                                                  FXXXH
T. hominis               VIFGPFLEEIIFRR----FLYQLVKGDRRLTKAIRNSEWRN------IFCLVILITINSTLFALLHYDEHYVHFFPWPFL
S. hominis SK119         SVVGPILEEYVFRK----VFFG------ELYDRIKGNRIIA------FL---IASTVSSLLFALAHN--DIKFILI----
S. hominis C80           SVVGPILEEYVFRK----VFFG------ELYDRIKGNRIIA------FL---IASTVSSLLFALAHN--DIKFILI----
P. donghaensis           VLFGPVLEELVFRR----VLFG------SLNQTTN------------FF---FATAVSALTFALIHF--DFTHLLL----
S. pseudintermedius      AIVGPLLEEFVFRK----VLFG------ELYHAIKANQWVK------FT---IATTISSAVFAVVHM--DFSHFLA----
S. aureus                VIVAPIYEEIIFRGIIYFYFIK------AIEKLFSQKSWEINARYSVIMCNGMFIMTSSLLFAYLHAWDTYIEAIP----
A. thaliana              CVVAPILEEIVYRR----FLLT------SLASRME---WW------------KALVISSGVFAAGHF--SGEDFVQ----
G. max                   CIVTPLLEEVVYRG----FLLT------SLFSTLE---WQ------------QAVAISSVVFSAIHF--SGENFLQ----
O. tauri                 CVVAPALEEFFFRG----FLYD------DLITRSGSNA--------------LAVTISSMVFAVAHF--SPRDVPS----
                                  ▲

                                                  H
T. hominis               YFACGGVNLCLACELCDNIVSAVFMHIFFNFFVL-------VKAIVTA---------------------------
S. hominis SK119         YFGMGMI-LSLAYTLTKRISVPILIHIFQNGFVV-------VMQFFLGDSL-NHLKQQTQFIIHLCN--------
S. hominis C80           YFGMGMI-LSLAYTLTKRISVPILIHIFQNGFVV-------VMQFFLGDSF-NHLKQQTQFIIHLCN--------
P. donghaensis           YFTTGLI-LAFLYQKTKSIVTPIIAHILLNSYVM-------VIQLNMDKIM-EFQKQLENLQ-------------
S. pseudintermedius      YFVMGII-FSAFYIYTKRLSVSIGIHMAQNGLVA-------LIQLMMPEKMIEDAVKQTQFILLLTSHWMSFFLS
S. aureus                YLSMGII-LSAVVVISKNIINSILIHMINNFFAFMYVETKEIFSLLIALIAVAIIMCLLELKRKRIS--------
A. thaliana              LFGIGCG-LGLCYSWSGNLASSVLVHSLYNALTL-------LFS-------------------------------
G. max                   LFIIGCV-LGCSYCWSGNLSSSIAIHSLYNALTL-------VITYFY----------------------------
O. tauri                 LATCGVV-FAFAKSTPSGLPAAVVAHAVFNASVL-------VERALAS---------------------------
```

B

Trachipleistophora hominis
orf3051

Staphylococcus hominis
ZP_04061034.1

Arabidopsis thaliana
NP_565483.1

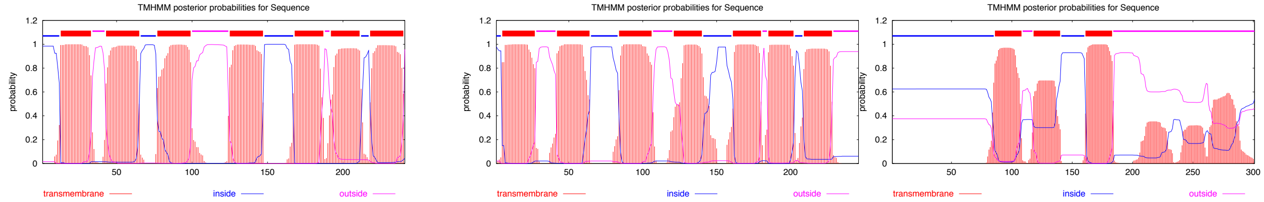

Supplement: Figure S14 — An endomembrane metalloprotease (CAAX-like peptidase) of the M79 family in T. hominis was potentially acquired by lateral gene transfer. (A) Protein alignment of the potential LGT orf_3051 with its closest BLAST hits. The three functional motifs for M79 peptidases are highlighted in green. Further details for the sequences displayed are given in Table S17. (B) The predicted transmembrane profile obtained using TMHMM [96] for the T. hominis protein is much more similar to the Staphylococcus hominis protein than to the eukaryotic sequence from Arabidopsis thaliana (Table S17). (PDF) [file ppat.1002979.s014.pdf]
