# Supplementary material for: The Genome of the Obligate Intracellular Parasite Trachipleistophora hominis: New Insights into Microsporidian Genome Dynamics and Reductive Evolution
Source: PLoS Pathog. 2012 Oct 25;8(10):e1002979. doi: 10.1371/journal.ppat.1002979 (PMC3486916; doi:10.1371/journal.ppat.1002979)
Supplement: Figure S7 — Phylogenetic analysis provides evidence for LGT of a piggyBac transposon between an ant and T. hominis. The tree suggests that LGT of a piggyBac transposon has occurred between the ancestors of the ant Harpegnathos saltator and T. hominis. The tree shown is from a Bayesian analysis of Dayhoff-recoded amino acid sequences performed with p4 using the NDCH model with an additional base composition vector. Further details of the analysis are given in the legend to Figure S4. The accession number (gi) or the T. hominis ORF locus tag are given for each sequence in the tree. (PDF) [file ppat.1002979.s007.pdf]

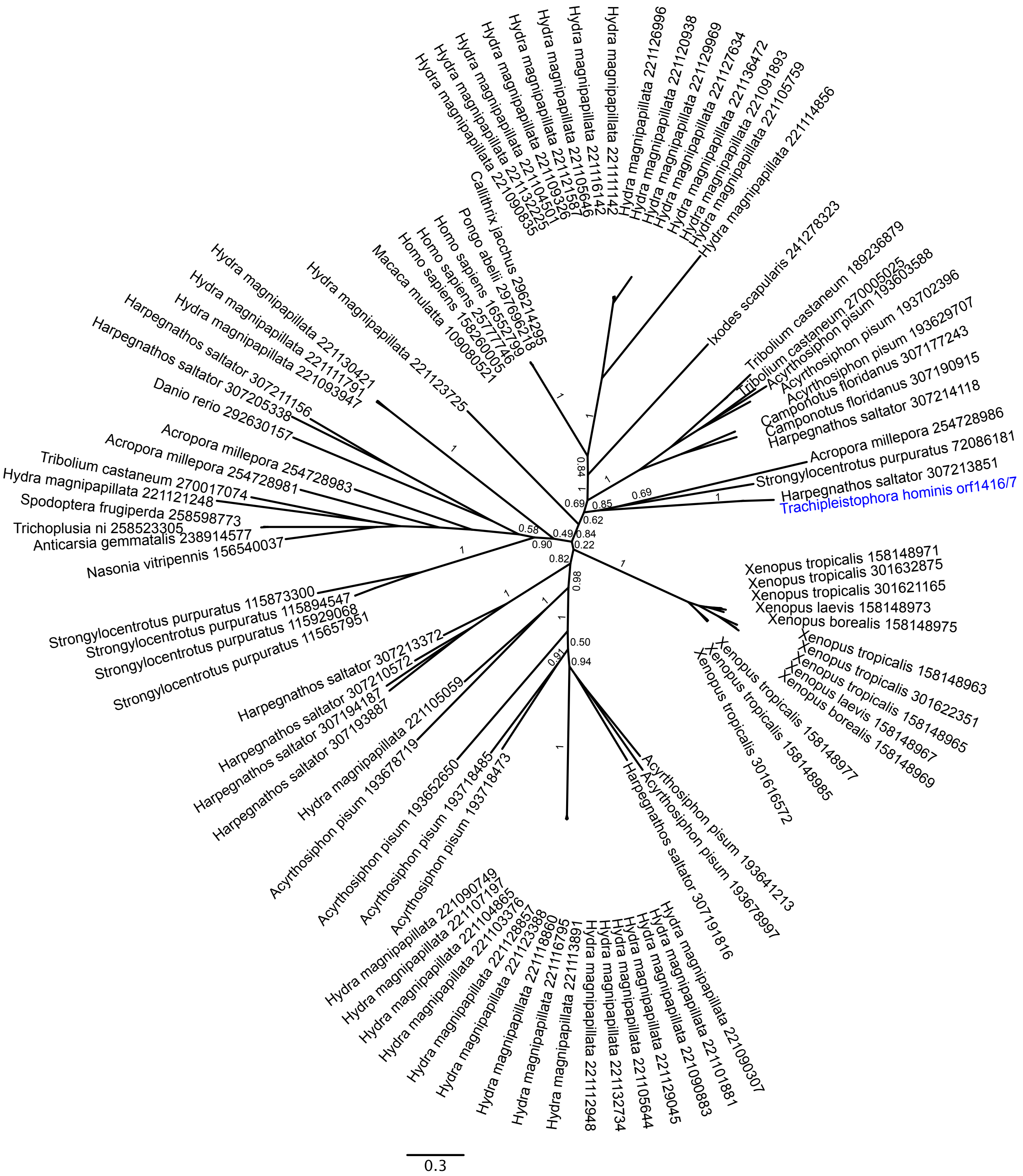

Hydra magnipapillata 221090835
Hydra magnipapillata 221132225
Hydra magnipapillata 221104501
Hydra magnipapillata 221109326
Hydra magnipapillata 221121587
Hydra magnipapillata 221105646
Hydra magnipapillata 221116142
Hydra magnipapillata 221111142
Hydra magnipapillata 221126996
Hydra magnipapillata 221120938
Hydra magnipapillata 221129969
Hydra magnipapillata 221127634
Hydra magnipapillata 221136472
Hydra magnipapillata 221091893
Hydra magnipapillata 221105759
Hydra magnipapillata 221114856
Ixodes scapularis 241278323
Callithrix jacchus 296214295
Pongo abelii 297696218
Homo sapiens 165527799
Homo sapiens 257777746
Homo sapiens 158260005
Macaca mulatta 109080521
Hydra magnipapillata 221123725
Hydra magnipapillata 221130421
Hydra magnipapillata 221111791
Hydra magnipapillata 221093947
Harpegnathos saltator 307211156
Harpegnathos saltator 307205338
Danio rerio 292630157
Acropora millepora 254728983
Acropora millepora 254728981
Tribolium castaneum 270017074
Hydra magnipapillata 221121248
Spodoptera frugiperda 258598773
Trichoplusia ni 258523305
Anticarsia gemmatalis 238914577
Nasonia vitripennis 156540037
Tribolium castaneum 189236879
Tribolium castaneum 270005025
Acyrthosiphon pisum 193603588
Acyrthosiphon pisum 193702396
Acyrthosiphon pisum 193629707
Camponotus floridanus 307177243
Camponotus floridanus 307190915
Harpegnathos saltator 307214118
Acropora millepora 254728986
Strongylocentrotus purpuratus 72086181
Harpegnathos saltator 307213851
Trachipleistophora hominis orf1416/7
Xenopus tropicalis 158148971
Xenopus tropicalis 301632875
Xenopus tropicalis 301621165
Xenopus laevis 158148973
Xenopus borealis 158148975
Xenopus tropicalis 158148963
Xenopus tropicalis 301622351
Xenopus tropicalis 158148965
Xenopus laevis 158148967
Xenopus borealis 158148969
Xenopus tropicalis 158148977
Xenopus tropicalis 158148985
Xenopus tropicalis 301616572
Strongylocentrotus purpuratus 115873300
Strongylocentrotus purpuratus 115894547
Strongylocentrotus purpuratus 115929068
Strongylocentrotus purpuratus 115657951
Harpegnathos saltator 307213372
Harpegnathos saltator 307210572
Harpegnathos saltator 307194187
Harpegnathos saltator 307193887
Hydra magnipapillata 221105059
Acyrthosiphon pisum 193678719
Acyrthosiphon pisum 193652650
Acyrthosiphon pisum 193718485
Acyrthosiphon pisum 193718473
Acyrthosiphon pisum 193641213
Acyrthosiphon pisum 193678997
Harpegnathos saltator 307191816
Hydra magnipapillata 221090749
Hydra magnipapillata 221107197
Hydra magnipapillata 221104865
Hydra magnipapillata 221103376
Hydra magnipapillata 221128857
Hydra magnipapillata 221123388
Hydra magnipapillata 221118860
Hydra magnipapillata 221116795
Hydra magnipapillata 221113891
Hydra magnipapillata 221112948
Hydra magnipapillata 221132734
Hydra magnipapillata 221105644
Hydra magnipapillata 221129045
Hydra magnipapillata 221090883
Hydra magnipapillata 221101881
Hydra magnipapillata 221090307
0.84
0.69
0.85
0.69
0.62
0.58
0.49
0.84
0.90
0.22
0.82
0.98
0.50
0.91
0.94
0.3
